# The relative contribution of DNA methylation and genetic variants on protein biomarkers for human diseases

**DOI:** 10.1371/journal.pgen.1007005

**Published:** 2017-09-15

**Authors:** Muhammad Ahsan, Weronica E. Ek, Mathias Rask-Andersen, Torgny Karlsson, Allan Lind-Thomsen, Stefan Enroth, Ulf Gyllensten, Åsa Johansson

**Affiliations:** Department of Immunology, Genetics and Pathology, Science for Life Laboratory, Uppsala University, Uppsala, Sweden; Albert Einstein College of Medicine, UNITED STATES

## Abstract

Associations between epigenetic alterations and disease status have been identified for many diseases. However, there is no strong evidence that epigenetic alterations are directly causal for disease pathogenesis. In this study, we combined SNP and DNA methylation data with measurements of protein biomarkers for cancer, inflammation or cardiovascular disease, to investigate the relative contribution of genetic and epigenetic variation on biomarker levels. A total of 121 protein biomarkers were measured and analyzed in relation to DNA methylation at 470,000 genomic positions and to over 10 million SNPs. We performed epigenome-wide association study (EWAS) and genome-wide association study (GWAS) analyses, and integrated biomarker, DNA methylation and SNP data using between 698 and 1033 samples depending on data availability for the different analyses. We identified 124 and 45 loci (Bonferroni adjusted *P* < 0.05) with effect sizes up to 0.22 standard units’ change per 1% change in DNA methylation levels and up to four standard units’ change per copy of the effective allele in the EWAS and GWAS respectively. Most GWAS loci were *cis*-regulatory whereas most EWAS loci were located in *trans*. Eleven EWAS loci were associated with multiple biomarkers, including one in *NLRC5* associated with CXCL11, CXCL9, IL-12, and IL-18 levels. All EWAS signals that overlapped with a GWAS locus were driven by underlying genetic variants and three EWAS signals were confounded by smoking. While some *cis*-regulatory SNPs for biomarkers appeared to have an effect also on DNA methylation levels, *cis*-regulatory SNPs for DNA methylation were not observed to affect biomarker levels. We present associations between protein biomarker and DNA methylation levels at numerous loci in the genome. The associations are likely to reflect the underlying pattern of genetic variants, specific environmental exposures, or represent secondary effects to the pathogenesis of disease.

## Author summary

Every human being is unique, each with an individual risk of developing disease. Variation between humans can be attributed to genetic variation or the environment to which we are exposed. However, phenotypic differences can also be due to the epigenetic pattern of our genes, which refers to chemical modification that affects the activity of our genes. Epigenetic factors have recently gained a lot of interest, and aberrant epigenetic patterns, have been linked to many diseases. In the present study, we have identified genetic and epigenetic factors that influence the activity of our genes, by way of comparing to the level of different proteins that our genes encode, proteins that have previously been associated with human diseases such as cardiovascular diseases or cancer. We could clearly see that the epigenetic pattern was associated with the expression of specific genes but that such associations were mainly caused by genetic variants, or environmental factors influencing both epigenetic and expression patterns. However, it appeared that some disease-associated proteins could have altering effects on the epigenetic pattern, then suggesting that some epigenetic alterations represent secondary effects to the pathogenesis of disease.

## Introduction

Over the past few years, Genome-Wide Association Studies (GWAS) have resulted in the identification of thousands of genetic variants that are associated with human traits and diseases. However, identified single nucleotide polymorphisms (SNPs) only explain a small fraction of the risk of disease, and most of the genetic contribution to human traits remains unidentified [1]. It has also been suggested that other molecular factors, such as epigenetic modifications and protein biomarkers are associated with disease development. While biomarkers are often considered being markers of disease, epigenetic factors have commonly been suggested to have a causal effect on disease development. Epigenome-wide association studies (EWAS) have therefore been performed aiming to identify associations between epigenetic modifications and disease traits and differential epigenetic patters have been identified for e.g. asthma, obesity and myocardial infraction [2–4]. However it is currently unclear whether the epigenetic variation is causal in the pathogenesis of disease. Epigenetic variation can be influenced by genetic variation [5], binding of transcription factors [6] or by external (environmental) factors such as smoking [7]. Whether associations between epigenetic factors and disease risk reflect disease pathogenesis, if the association are confounded by unknown environmental factors or if epigenetic variation is causal for disease development has still yet to be investigated.

Epigenetic modifications play an important role in regulation of gene expression [8] and in pathogenesis of human diseases [9]. This raises the possibility that epigenetic variation can be causal for disease development or can even to contribute to the heritability of human traits. Trans-generational heritability of epigenetic alterations, i.e. epigenetic changes that escape reprogramming during the formation of our gametes and during the embryogenesis [10] has not been demonstrated in humans. Intergenerational heritability on the other hand, has. A fetus is already exposed to a certain environment before birth, and epigenetic changes also occur during this time. An example is that newborns to mothers that have been smoking during pregnancy have epigenetic changes that reflect the increased activity of genes, including *AHRR* encoding the aryl-hydrocarbon receptor repressor, which are involved in the metabolism of toxic components from tobacco smoke [11]. Those changes in *AHRR* are similar to those observed among adult smokers [7]. However, the activity of these genes is not likely to cause an increased risk of smoke-associated disorders. Rather, the exposure to toxic compounds during embryogenesis could serve as the causal factor for increased disease risk as well as for variation in *AHRR* DNA methylation. However, there are also epigenetic alterations caused by exposure to different pathogenic environmental factors that might result in increased disease risk. For example, hypermethylation of tumor suppressor genes can lead to gene inactivation and subsequent cancer progression [12]. The pre-birth accumulation of such causal epigenetic alterations, can thereby also explain part of the heritability of human traits. In addition, it has been proposed that genetic variants that allow for more dynamic changes in gene expression (which is reflected by alterations in the epigenetic patterns themselves) further contribute to the heritability [13].

Similarly to epigenetic alterations, elevated levels of many proteins have been associated with various diseases and are therefore considered to be biomarkers for respective disease. Protein biomarkers are produced naturally in the body. Increased levels of a biomarker can reflect up-regulation of a gene due to disease pathogenesis or due to epigenetic factors. One could therefore believe that epigenetic alterations should give rise to changes in many downstream factors. However, it is also known that protein levels vary between individuals due to genetic variants and lifestyle factors [14,15]. It is therefore possible that genetic, environmental and/or lifestyle factors are the major causes of association between epigenetic alterations and disease related biomarker levels, as well as between epigenetic alterations and disease prevalence where the epigenetic alterations by themselves are not causing the disease.

In this study we have investigate the genetic and epigenetic contribution to variation in a large set of disease related biomarkers and addressed if epigenetic alterations by can be causal for variation in biomarker levels. We have used large scale genetic, epigenetic and proteomic data to describe the association between genetic and epigenetic factors and protein biomarkers for inflammation, cancer and cardiovascular diseases. We have used the highly sensitive and specific Proximity Extension Assay (PEA) [16] to measure the abundance of 144 established or potential protein biomarkers for inflammation, cancer and cardiovascular disease (S1 Table) [15,17] in plasma from 1,004 individuals from a cross-sectional population-based cohort from Sweden. We have then integrated data from over 10 million SNPs and DNA methylation levels at 470,000 CpG sites with the protein biomarker data. The specific objectives were to: 1) identify associations between genetic variants and biomarker levels, 2) identify associations between DNA methylation (one epigenetic mechanism) and biomarkers and measure to what extent these associations are caused by genetic variants, 3) investigate whether measured DNA methylation can explain parts of the variation in biomarker levels that are not explained by genetic variants, and 4) investigate whether genetic variants regulate DNA methylation levels also influences protein biomarker abundance or vice versa.

## Results

A total of 1033 participants with genotype data were included in this study. A subset of these participants had good quality measured biomarkers (N = 961) and DNA methylation (N = 729) data available, of which 698 had both biomarker and DNA methylation data available (S2 Table). The age of the participants ranged from 14 to 94 years with a median of 50.4 years, and 53% of the participants were females. The cohort is population-based with participants being invited independent on disease and health status, with a number of participants having (or having experienced) different diseases (S2 Table).

### GWAS for biomarker levels

**Fig 1:**
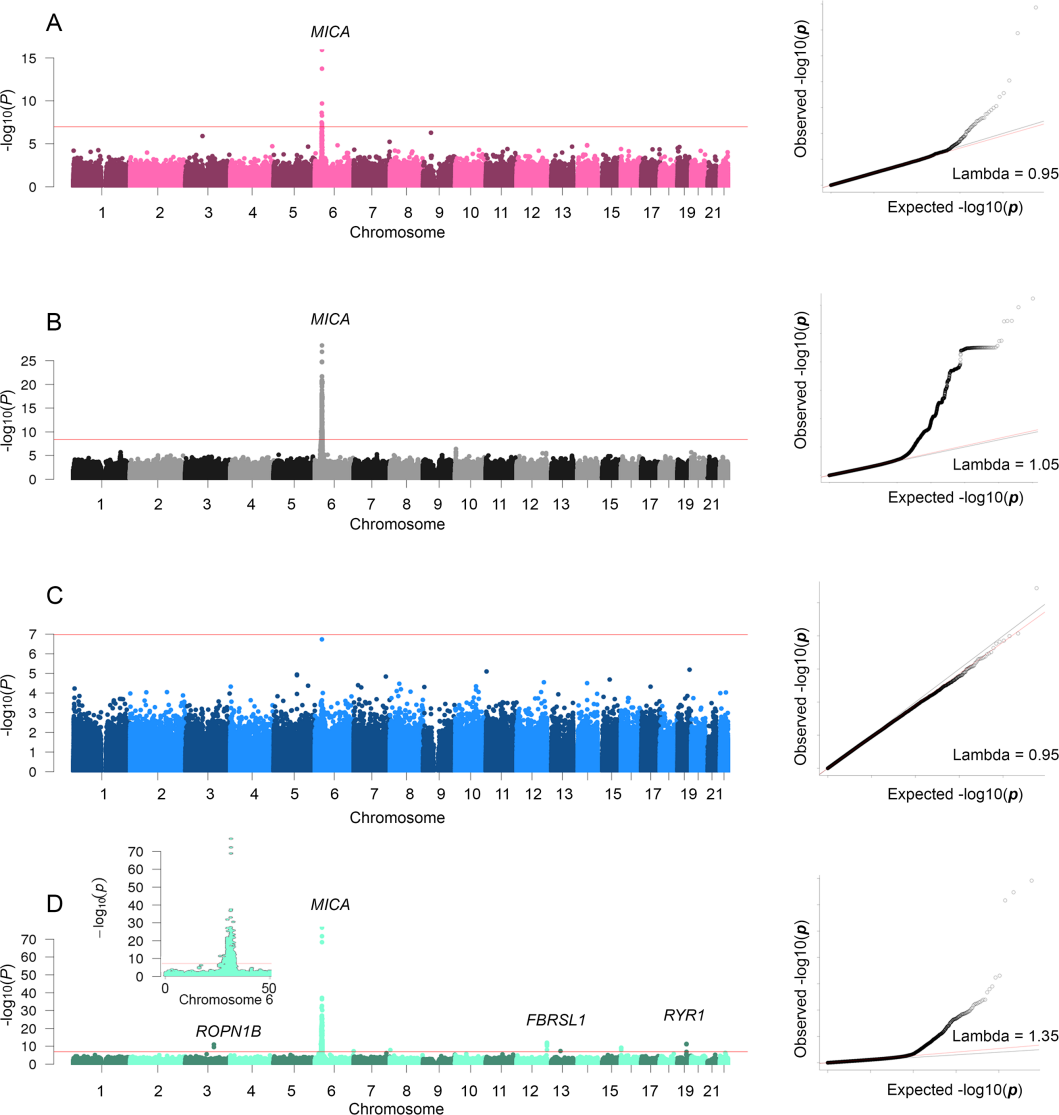
Manhattan and QQ plots for MIC-A. The red line in the Manhattan plots (left panel) indicates the threshold of significance (Bonferroni adjusted P-value = 0.05). The black line in the QQ plots (right panel) shows the 1:1 regression line and the red line in the QQ plots is the regression line illustrating the inflation of low P-values and the slope of the red line equals the inflation factor (Lambda). A) Results from the primary EWAS between MIC-A and DNA methylation. B) Results from the primary GWAS. C) Results from the EWAS adjusted for all independent SNPs (N = 3) identified in the GWAS. D) Association between MIC-A GS and DNA methylation levels across the genome, and a zoom-in on a region of chromosome 6.

In total, 121 of the 144 biomarkers passed quality control (QC) and were used in the analysis. Many biomarkers are closely related and their abundance level in plasma clustered accordingly (S1 Fig). We previously used a two-stage GWAS design to identify genetic variants associated with protein abundance levels [15,18]. In the current study we combined the material and used an updated reference panel for imputing genotypes not included on the genotyping arrays. Previously we have identified GWAS signals for 24 of the biomarkers [15,17]. Here we have increased this number by identifying 45 GWAS regions (hits) for 39 of the biomarkers (Bonferroni adjusted *P* < 0.05, adjusted for 11,901,634 SNPs tested) (Fig 1, S2 Fig and S3 Table). Using a more strict adjustment for multiple texting (*P* **<** 4.1e-10 - see method section), that also adjusts for the number of biomarkers include, resulted in 35 GWAS regions for 30 biomarkers. In the GWASes, the inflation of low P-values was limited, with lambda estimates ranging from 0.90 to 1.06 (Fig 1, S2 Fig and S1 Table). For a majority of these biomarkers (N = 24) the associations represented *cis*-regulatory SNPs that were located in direct proximity to the respective protein-encoding gene. For another five of the biomarkers, the associated SNP was located within one megabase from the gene that encodes the respective protein (S4 Table). Adjusting for the most significant SNP for each biomarker resulted in secondary (conditional) signals for five biomarkers (CCL24, MIC-A, MMP-7, MMP-10 and VEGFR-2, S5 Table) and tertiary signals for two biomarkers (CCL24 and MIC-A, S6 Table).

### EWAS for biomarker levels

**Fig 2:**
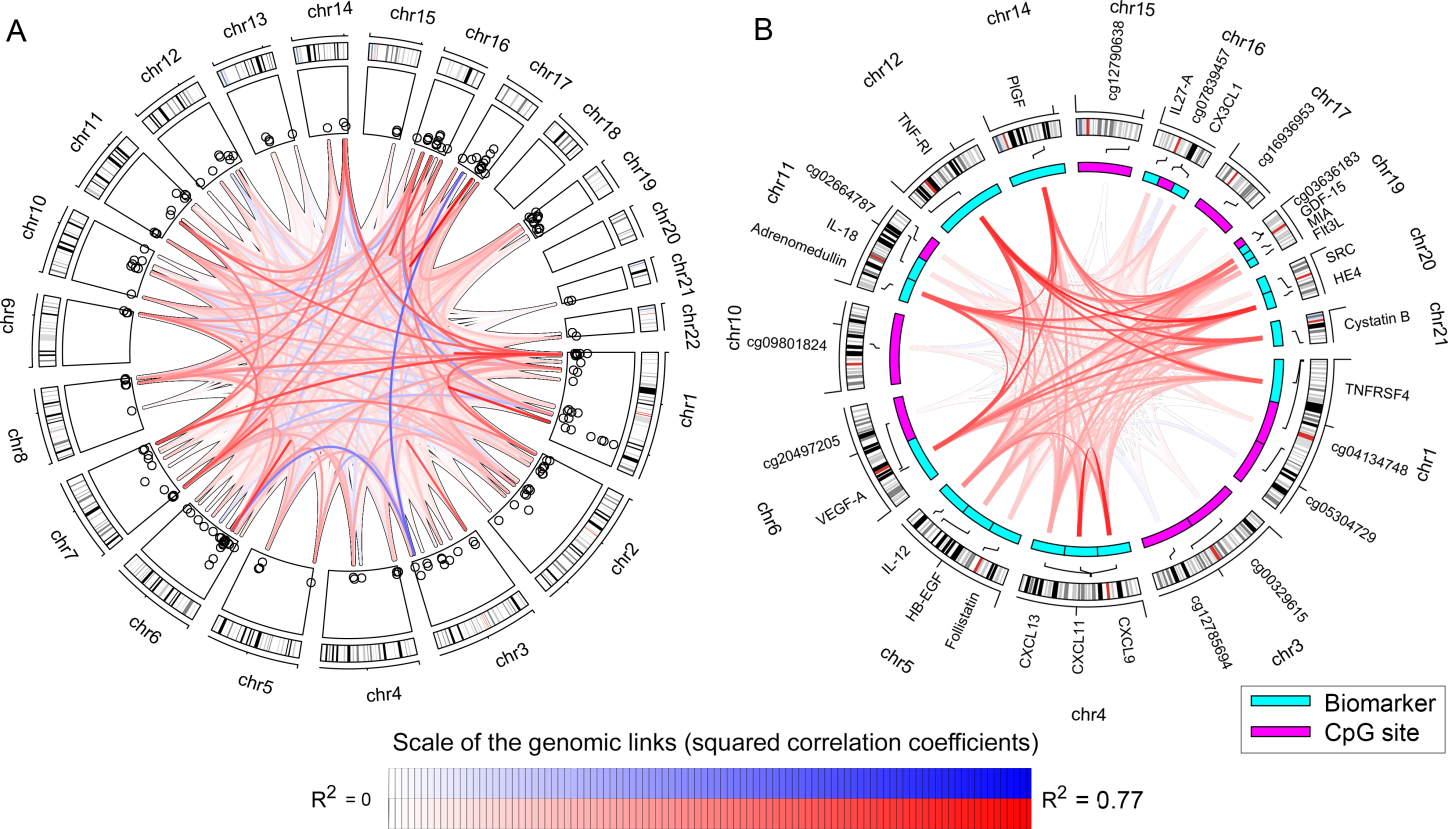
Correlation links between genomic regions. The links between the CpG sites represent squared correlation coefficients (R^2^) ranging from zero to 0.77 reflected by the intensity in the color with red representing positive correlations and blue negative correlation. A) Shows the correlation pattern between DNA methylation levels at different CpG sites across the autosomal chromosomes. Only CpG sites associated with any of the biomarkers are included. The outer circle shows the chromosome number, second circle shows the cytobands for each chromosome clock wisely directed. The third circle is an extract from the Manhattan plot the–log10(P-values), with the scale ranging from 7 to 24. B) Shows the correlation pattern between CpG sites that are associated with multiple biomarkers and the correlation pattern between these biomarkers. The second circle is the names of the CpG sites and the biomarkers included in theses analyses. Inside the cytoband-circle, the CpG cites (magenta) and the genes encoding respective biomarker (cyan) with lines to the genomic position are shown.

The EWAS revealed a total of 188 significant associations distributed over 131 chromosomal regions with the abundance levels of 44 biomarkers (Bonferroni adjusted *P* < 0.05, adjusted for 473,864 sites tested) (Fig 1, S3 Fig, S7 Table). The inflation of low P-values were somewhat higher in the EWAS compared to the GWAS, with lambda estimates ranging from 0.9 to 1.52 (Fig 1, S3 Fig and S1 Table). A subset of the probes that were used to measure the DNA methylation levels have previously been highlighted as potentially cross-hybridizing to other parts of the genome [19]. Removing such probes from the results reduced the number of chromosomal regions associated with biomarker levels from 131 to 124. For CCL24, CCL4, CHI3L1, Cystatin B, IL6R, MIC-A and RETN the signals mapped to a *cis*-located CpG site near the gene encoding the respective biomarker, similar to what was observed in the GWAS. Adjusting for the most significant CpG site from each association signal did not result in any significant genome-wide secondary signals. In contrast to the GWAS signals (which, for many biomarkers extended over several megabases) most of the EWAS signals were represented by one single CpG site. Only 20 regions consisted of multiple biomarker-associated CpG sites, including those associated with GDF-15 [20], Flt3L and CXCL9. Out of 57 CpG sites in these 20 regions, 28 sites represented partially independent effects (see the Method section) although only one met the threshold for genome-wide significance (S8 Table). We observed fairly high correlations between the methylation levels at some distantly located CpG sites (Fig 2A). Most correlations were positive with strong correlations between CpG sites located in the same chromosomal regions (e.g. on chromosome 1, 3, 6, 7, 16, and 17), representing the dependent effects already investigated (S8 Table). However, we also observe some correlations between trans-located CpG sites.

In total, 13 different CpG sites were associated with more than one biomarker (Table 1, Fig 2B). These sites were distributed over eleven genomic regions and all but one is located within or in close proximity to genes that have been implicated in cardiovascular disease, cancer or inflammation [21–32] (Table 1). One of the CpG sites, cg03636183, in *F2RL3* was associated with both IL-12 and WFDC2 levels, and another site, cg05575921, in *AHRR* was associated with IL-12 levels. By including smoking in our model, these associations (between DNA methylation and biomarkers) were no longer significant (*P* > 0.05), which reveals that tobacco smoking was confounding these associations. The P-values for none of the other associations were influenced by including smoking as a covariate in the analysis.

**Table 1:** CpG sites that are associated with multiple biomarkers, and previous associations between the CpG locus diseases.

CpG Name	Gene(s) annotation CpG Gene name	Disease related gene function	Biomarker	Pathogenic association of biomarker	Estimate ^b^	*P*
cg00329615	Immunoglobulin superfamily member 11 (*IGSF11)*	*IGSF11* stimulates cell growth and is frequently up regulated in intestinal gastric cancer [53]	IL-12	Inflammation, cancer	3.48	3.8x10^-8^
			MIA	Cancer	3.52	1.8x10^-8^
			TNFRSF4	Cancer	4.16	3.1 x10^-10^
cg02664787	Opioid binding protein/cell adhesion molecule-like (*OPCML)*	*OPCML* is involved in ovarian cancer and studies have shown epigenetic inactivation in ovarian tumors [29]	PlGF	Cardiovascular, cancer	-4.87	1.5 x10^-8^
			TNF-RI	Cardiovascular, cancer	-4.46	5.3 x10^-8^
cg03636183 ^c^	Coagulation factor II (Thrombin) receptor-like 2 (*F2RL3)*	*F2RL3* helps to sustain platelet aggregation during prolonged thrombin exposures [22] and DNA methylation at *F2RL3* has been shown to be a strong predictor of mortality [32].	WFDC2	Cancer	-3.41	3.1 x10^-8^
			IL-12^c^	Inflammation, cancer	4.36	9.9 x10^-10^
cg04134748	PR domain containing 16 (*PRDM16)*	*PRDM16* is associated to left ventricular noncompaction and cardiomyopathy [21]	Flt3L	Inflammation, cancer	3.96	5.3 x10^-12^
			SRC	Cardiovascular	-3.77	9.1 x10^-9^
cg05304729	Myeloid cell nuclear differentiation antigen (*MNDA*)	*MNDA*is identified as a marker for nodal marginal zone lymphoma [26]	CXCL11	Inflammation, cancer	-6.51	1.1 x10^-9^
			CXCL9	Inflammation, cancer	-5.36	2.5 x10^-8^
cg07839457 ^a^	NLR family, CARD domain containing 5 (*NLRC5*)	*NLRC5* is involved in immune defense [24]	CXCL11	Inflammation, cancer	-3.26	3.5 x10^-11^
			CXCL9	Inflammation, cancer	-2.84	1.2 x10^-10^
			IL-12	Inflammation, cancer	-2.62	2.9 x10^-8^
			IL-18	Cardiovascular, inflammation	-2.6	7.0 x10^-8^
cg09801824	DnaJ (Hsp40) homolog, subfamily B, member 12 *(DNAJB12)*, DNA-damage-inducible transcript 4 *(DDIT4)*	*VMP1* is associated with Lp-PLA_2_ which is associated with increased cardiovascular risk [23]. *VMP1* and *MIR21* are involved in cancer risk [28].	CXCL11	Inflammation, cancer	9.91	3.6 x10^-9^
			CXCL9	Inflammation, cancer	9.67	1.2 x10^-10^
cg12785694	Structural maintenance of chromosome 4 (*SMC4)* Intraflagellar transport 80 *(IFT80)*	*SMC4 is associated with cancer* [25].	CXCL11	Inflammation, cancer	-4.43	6.7 x10^-8^
			IL-12	Inflammation, cancer	-4.41	8.1 x10-^9^
cg12790638	Hyaluronan and proteoglycan link protein 3 *(HAPLN3)*, Milk fat globule-EGF factor 8 protein *(MFGE8)*	*HAPLN3 and MFGE8 are involved in various cancers* [30,31].	Adrenomedullin	Cardiovascular, cancer	3.95	7.2 x10^-8^
			Cystatin B	Cardiovascular, cancer	3.7	1.5 x10^-8^
			VEGF-A	Cardiovascular, cancer, inflammation	3.98	5.4 x10^-8^
cg16936953^a^	Vacuole membrane protein 1 (*VMP1*), Micro-RNA 21 (*MIR21*)	*VMP1* is associated with Lp-PLA_2_ which is associated with increased cardiovascular risk [23]. *VMP1* and *MIR21* are involved in cancer risk [28].	CXCL13	Cancer	-3.68	5.6 x10^-9^
			Follistatin	Cardiovascular, cancer	-3.55	1.0 x10^-7^
			GDF-15	Cardiovascular, cancer	-3.74	2.5 x10^-15^
			IL27-A	Cardiovascular	-3.04	8.9 x10^-9^
cg20497205	Opsin 5 *(OPN5)*		CX3CL1	Cardiovascular, inflammation	9.04	4.1 x10^-8^
			HB-EGF	Cardiovascular, cancer	8.19	4.1 x10^-8^

a. The *NLRC5* and *VMP1/MIR21* regions consist of multiple associated CpG sites. However, these CpG sites are not independent and therefore only the most significant CpG site is included in this table.

b. The estimates represent change in biomarker values (in standard deviations) per 100% change in DNA methylation level (from 0 to 100%)

### Integrated analyses and heritabilities

**Fig 3:**
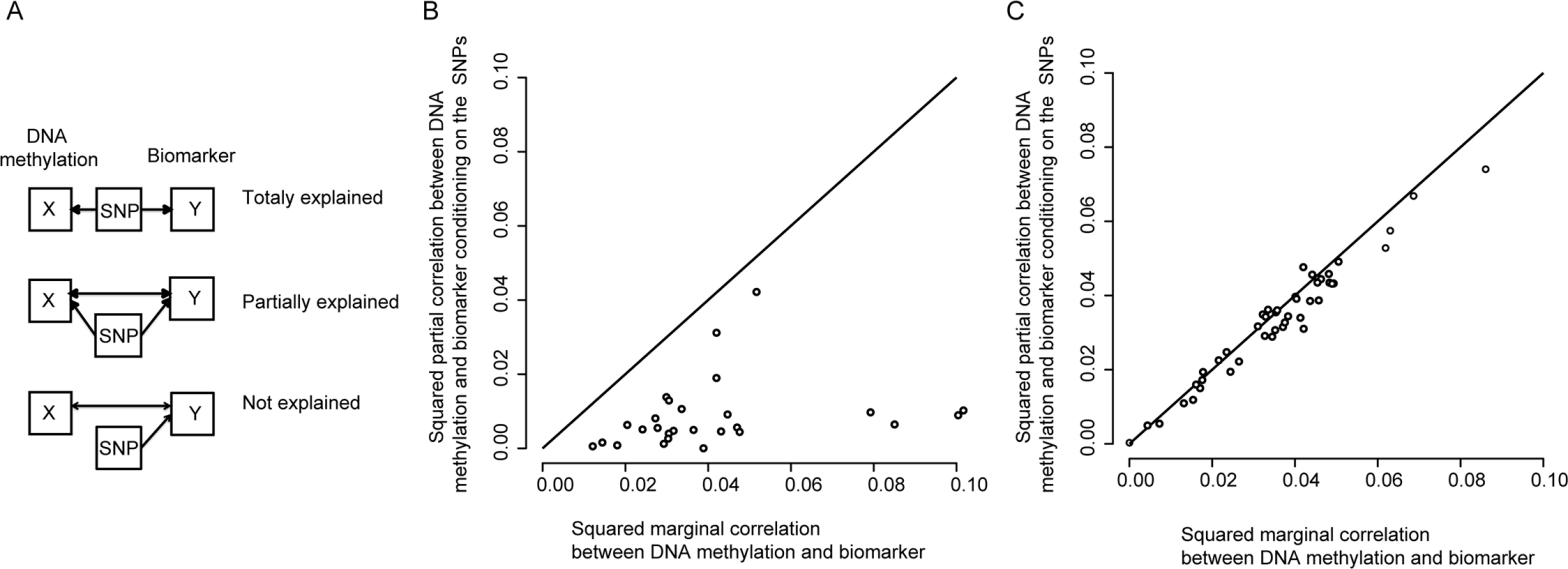
Association between DNA methylation and biomarkers explained by SNPs. A) Overview of possible scenarios. If the associations are fully explained (caused) by the SNPs, there will be no correlation between the DNA methylation and the biomarker after conditioning on the SNP. If the association is partially explained, there will be a correlation between the DNA methylation and the biomarker after conditioning on the SNPs, but the correlation will be weaker. If the SNP is not causing the association, the correlation between the DNA methylation and the biomarker will not be influenced when conditioning on the SNPs. B) and C) The squared marginal and partial (after conditioning on the SNPs) correlation coefficients between the DNA methylation and the biomarkers for pairs where a cis-regulatory SNP influencing the biomarker levels has been identified. In B) for pairs where the CpG site and the gene encoding the biomarker maps to the same locus, and in C) for pairs where CpG site and the gene encoding the biomarker map to different regions. For calculating the marginal and partial correlation coefficients, only individuals with DNA methylation, biomarker and SNP data available were included (N = 698).

Integrating the results of the EWAS and GWAS, 18 biomarkers (CCL19, CCL24, CCL4, CHI3L1, Cystatin B, Flt3L, IL-12, IL17RB, IL6RA, MIA, MIC-A, MMP-12, NEMO, RETN, ST2, TIM, TM, and TRAIL) showed significant associations with both SNPs and DNA methylation levels. For eleven of these biomarkers the GWAS and EWAS signals were located in the same region (within two megabases from each other). To determine if the EWAS and GWAS signals in the overlapping regions were independent, the SNPs for each biomarker (S9 Table) were used as covariates in the EWAS (Fig 1, S4 and S5 Figs and S10 Table). None of the 11 EWAS hits that were located in the same region with a GWAS hit remained genome-wide significantly after adjusting for the top SNPs (Fig 3B, Table 2). However, considering the number of test performed (15 tests for 11 biomarkers), two were still significant after adjusting for multiple testing. This suggests that most of the EWAS signals were partly driven by underlying genetic variation (Fig 3A and 3B). For seven biomarkers, where the EWAS hit was not located in the same region as the GWAS hit, the EWAS signals were mainly unaffected or even became more significant by adjusting for the top SNPs (Fig 3C, S10 Table). The latter is expected when adjusting for an important precision variable (in this case the SNP) that influences the variation in the outcome variable, since failure to adjust for precision variables will tend to attenuate association between the biomarker and the DNA methylation levels towards the null.

**Table 2:** CpG sites and SNPs in the same chromosomal region that are associated with the same biomarkers.

Biomarker	Chr	SNP name ^a^	SNP position	*P* (GWAS) N = 961 ^b^	CpG name(s) ^c^	CpG position	*P* (EWAS) N = 698 ^b^	*P*_*adj*_ (EWAS)^d^ N = 698 ^b^	*P* (CpG-SNPs)^e^ N = 729 ^b^
CCL19	6	rs2395201	32451897	5.9 x 10−^17^	cg26805579	32372962	1.8 x 10^−8^	0.038	1.5 x 10^−93^
CCL24	7	rs10755885	75463505	1.8 x 10−^39^	cg12943082	75419311	8.5 x 10^−13^	0.0057	3.6 x 10^−16^
		rs73359714	75447156	7.5 x 10−^37^					
		rs11465295	75442407	2.0 x 10−^12^					
CHI3L1	1	rs2153101	203168474	2.9 x 10−^41^	cg07423149	203156246	1.3 x 10^−24^	5.9 x 10^−5^	3.1 x 10^−75^
Cystatin B	21	rs35285321	45201832	7.2 x 10−^15^	cg09468832	45199094	1.5 x 10^−8^	0.12	7.8 x 10^−84^
					cg08529987	45206724	4.6 x 10^−9^	0.0024	6.6 x 10^−46^
IL6RA	1	rs12133641	154428283	3.0 x 10−^73^	cg21262032	154437693	1.2 x 10^−8^	0.18	8.9 x 10^−18^
MIA	19	rs3869574	41284915	2.4 x 10−^26^	cg09274963	41633902	1.1 x 10^−8^	0.0098	7.6 x 10^−45^
MIC-A	6	rs6938453	31377793	5.9 x 10−^29^	cg02884661	31382931	1.2 x 10^−16^	0.13	4.3 x 10^−31^
		rs52979004	31402666	4.2 x 10−^15^	cg04628742	29973221	2.4 x 10^−9^	0.032	6.3 x 10^−11^
		rs2516470	31407331	8.8 x 10−^10^	cg12001709	31466798	5.1 x 10^−9^	0.26	1.4 x 10^−13^
RETN	19	rs149552675	7624687	1.1 x 10−^10^	cg02346997	7733883,	3.1 x 10^−8^	1.6 x 10^−6^	2.5 x 10^−5^
RETN	19				cg22322184	7734203	9.1 x 10^−9^	1.9 x 10^−7^	0.0011
ST2	2	rs12712136	102936366	1.2 x 10−^46^	cg05295703	102895712	8.4 x 10^−9^	0.95	2.3 x 10^−49^

The table shows the results for the biomarkers with overlapping (with regards to chromosomal loci) signals from the EWAS and the GWAS, the association between biomarker and CpG sites when adjusted for the SNPs, and the association between CpG methylation and SNPs.

a. Only independent top SNPs are included

b. N–Sample size for each set of analyses.

c. Only independent top CpG sites are included

d. *P*-value between the CpG site and the biomarker after adjusting for all independent SNPs.

e. *P*-value for the association between the CpG site and all independent SNPs (combined)

We also estimated the heritability for each biomarker and tested whether the heritabilities were significantly different (see method section) when adjusting for associated GWAS or EWAS signals, which would indicate that the GWAS or EWAS hits could explain a significant part of the heritability (S11 Table). Many of the biomarkers, even the ones for which we did not find a GWAS hit, had high heritability estimates. For example: mAmP, TRANCE, GAL, VEGF-D and IL-18 all had high heritability estimates (>40% of the variation is due to heritable factors), but no significant SNPs were identified. This reflects the strict threshold of significance used in the GWAS resulting in a large number of false negative associations, which agrees with previous GWA studies [1]. For many of the GWAS-associated biomarkers, the heritability was still high after adjusting for the top GWAS SNP, including for biomarkers such as Ep-CAM, CD30-L, MMP-1, and CCL24 with residual heritabilities of over 50%. Only five biomarkers (IL6RA, CXCL10, CCL4, MIC-A, E-selectin, and CCL24) showed a significantly lower heritability after adjusting for the top GWAS hits. However, adjusting for the significant EWAS hits, on top of the GWAS SNPs, did not results in significantly lower heritability for any of the biomarkers.

### Mendelian Randomization to test for causal effects of increased biomarker levels

**Fig 4:**
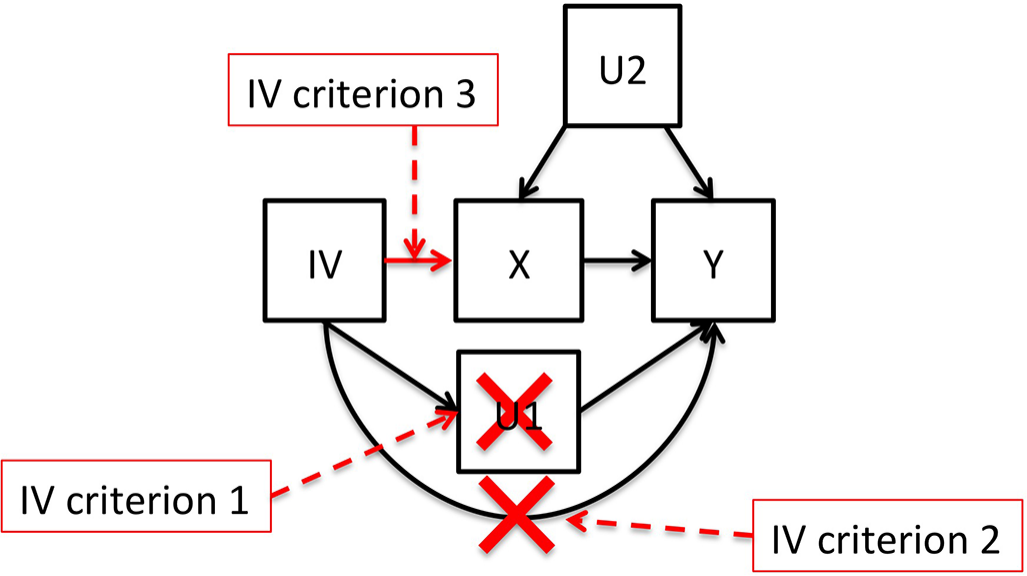
A directed acyclic graph of the direct effects and correlations between protein levels, genetic variants, DNA methylation and smoking. Single-headed arrows represent direct associations and double-headed arrows indicate correlations. Two of the correlations between DNA methylation and protein levels could be completely explained by the confounding by smoking that is influencing both DNA methylation and on protein levels. Similarly, 23 of the biomarker-DNA methylation correlations could be explained by genetic variants. The numbers in the figure represent the numbers that we found among the proteins and EWAS-significant CpG sites in our study.

We then used a Mendelian Randomization (MR) like approach to determine whether *cis*- regulatory methylation SNPs (meQTLs), SNPs associated with DNA methylation levels, were also influencing variation in biomarker levels, or if *cis*- regulatory biomarkers SNPs influenced the variation in DNA methylation (Fig 4). The basic concept in MR is that individuals have genetic variants that result in different genetic levels of e.g. DNA methylation or biomarker abundance [33]. To reduce the risk of pleiotropy only *cis*-regulatory SNPs (within one megabase from the gene encoding the biomarker) were considered for these analyses. The MR analyses was divided into five parts: 1) identify IVs for biomarker levels (*cis*-regulatory biomarkers SNPs), 2) test if the biomarker IVs are associated with DNA methylation levels, 3) identify IVs for DNA methylation levels (*cis*-meQTLs), 4) test if the DNA methylation IVs are associated with biomarker levels, and 5) use identified IVs in bi-directional MR analyses for a small subset of the biomarkers.

### Mendelian Randomization—Identification of IVs for biomarker levels

Genetic scores (GS), representing the increase (or decrease) in biomarker levels, were calculated for each individual (S12 Table) using SNPs as instrumental variables (IVs). For 29 of the biomarkers, we identified valid IVs according to the IV criterion 1 (see the Method section, Fig 4). Even though only one or a few SNPs (IVs) were included per biomarker, the strengths of the instruments were good. Some of the IVs explained over 40% of the variation in biomarker levels (S6 Fig) with F-values of the IVs ranging from 30 to 600 (S12 Table), which is far above the recommended minimum F-value of 10 that has been suggested for MR studies [34].

### Mendelian Randomization—Test if biomarker IVs are associated with DNA methylation levels

The IVs were tested for association with variation in DNA methylation across the genome. As expected, the IVs were associated with DNA methylation for all regions overlapping between the EWAS and GWAS. For these, we were not able to do any causal inference since the IV can be directly associated with the DNA methylation level as well as being associated through the biomarker level, and thereby fail the second IV criteria (Fig 4). However, many of the IVs were associated with DNA methylation at multiple loci in the genome (S7 Fig and S13 Table). For example, the IVs for MIC-A were not only associated with DNA methylation across a seven-megabase region around MHC on chromosome 6 (including *MICA*), but also with DNA methylation at multiple regions on other chromosomes (Fig 1D). This suggests that MIC-A levels might influence DNA methylation across the genome. For IL6RA, the IV was associated with methylation levels around *IL6R* (encoding IL6RA), but also with methylation levels at *HLA-DPA1* and *HLA-DPB1*, which are important genes for the immune system, indicating that IL6RA might influence the expression of these genes. Similarly, the IVs for 25 other biomarkers were associated (in trans) with DNA methylation patterns (S13 Table).

### Mendelian Randomization—Identification of IVs for DNA methylation levels

We then aimed to identify valid IVs for the DNA methylation levels at all EWAS-significant CpG sites. We first performed association studies for these CpG sites (S7 Table) to identify *cis*- meQTL, i.e. *cis*-regulatory SNPs that were associated with variation in DNA methylation level. Out of 169 unique CpG sites, 58 were associated (*P* < 0.5 x 10^−8^) with a *cis*-regulatory SNP located within one megabase from the CpG site (S14 Table). Of these, one SNP overlapped directly with the CpG site, and three were located close to the CpG site (< 50bp). However, most of the meQTL were located several thousand base pairs away from the CpG site.

### Mendelian Randomization—Test if DNA methylation IVs are associated with biomarker levels

**Fig 5:**
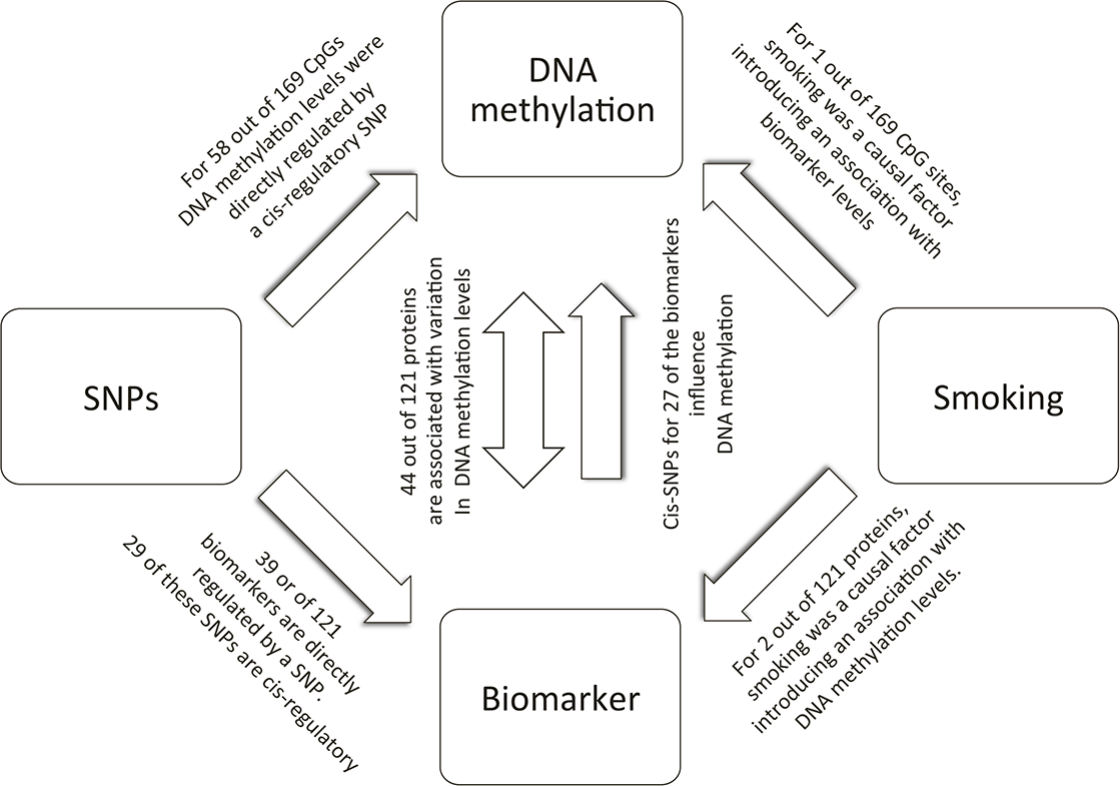
A directed acyclic graph of the causal inference. X and Y are two correlated phenotypes (the DNA methylation level at a CpG site and a biomarker level). The instrumental variable (IV) is the cis-regulatory genetic variant that directly influences X. If the IV is influencing Y through X in this way, there is a causal relation between X and Y. If the IV is not influencing Y through X, there might be an unknown confounding factor influencing both X and Y (U2), or there might be a reverse causation (Y influences X). To be a valid instrument three criteria must be fulfilled: IV criterion 1) the genetic variant must not be associated with an unknown confounding factor (U1) that influences Y, and IV criterion 2) the genetic variant must not be directly associated with Y, and IV criterion 3) the genetic variant should be associated with X.

To evaluate whether DNA methylation levels affect biomarkers directly, we used these meQTL as IVs, and tested for association with their respective CpG-associated biomarker. Not surprisingly, for associations that overlapped between EWAS and GWAS for the same biomarker, the *cis*-regulatory IVs identified were also associated with both DNA methylation and biomarker level. For these, the association between the DNA methylation and the biomarker was already shown be caused by the SNP to a large extent (Fig 5). Since these IVs are directly associated with both DNA methylation and biomarker levels, they are not valid as IVs in MR according to the second IV criteria (Fig 5). For the non-GWAS-overlapping EWAS hits, where we had valid IVs, there was no IV that was associated with biomarker levels after adjusting for the number of tests performed. Most of these IVs had high strength, with F-values ranging from 14 up to 400, with a median value of 81 (S14 Table). Therefore, these results do not support that variation in DNA methylation level itself directly affects the levels of these biomarkers.

### Bi-directional Mendelian Randomization

For biomarker-DNA methylation associations where we could identify IVs for both the biomarker and the DNA methylation levels, we performed bi-directional analyses. Bi-directional MR requires that the IVs for the two variables (biomarker and DNA methylation) are independent which discarded most of the associations from these analyses. Only for two of the associations, the IV for the biomarker and the DNA methylation levels were independent: rs10076557 (IL-12)—rs12526186 (cg03636183) with R^2^ = 0.001 (P = 0.34), and rs2279699 (MIA)—rs2515669 (cg21586223) with R^2^ = 0.0002 (P = 0.74). In either of the cases, we could not see a significant association between the biomarker IV the DNA methylation levels or the DNA methylation IV and the biomarker level (P>0.05). We can therefore not draw any conclusion with regards to causality based on this experiment.

## Discussion

We have investigated genetic and epigenetic determinants of protein biomarkers for cancer, inflammation or cardiovascular disease by integrating genome-wide DNA methylation and SNP data. The EWAS revealed 188 associations between protein abundance and DNA methylation, representing 131 loci of which 124 contained at least one probe that is not likely to cross-hybridize in the genome. In contrast, the GWAS revealed 5,951 associations between protein abundance and SNPs, representing 45 loci. The larger number of associations with SNPs partly reflects the larger number of SNPs analyzed (N = 11,901,634), compared to the number of CpG sites (N = 473,864) with the fraction of significant sites being similar in the GWAS and the EWAS (0.04% and 0.05% respectively). However, the larger extent of correlation (linkage disequilibrium [LD]) between SNPs compared to the extent between DNA methylation levels at CpG sites also results in a larger number of associated SNPs.

We found that many of the biomarkers are regulated by *cis*-located SNPs in proximity to the protein-encoding gene. However, the EWAS hits were more spread throughout the genome. DNA methylation at a number of CpG sites was associated with more than one biomarker. Most of these CpGs were located within or in close proximity to genes that are part of the same biological pathways as the biomarkers and most of these genes have previously been shown to be involved in cardiovascular diseases, cancer or inflammation (Table 1). Some of the biomarkers, such as GDF-15, Flt3L and CXCL9, were associated with multiple CpG sites located in different chromosomal regions, whereas most of the biomarkers were only associated with DNA methylation at one region, similar to the results from the GWAS. In contrast to the GWAS signals, which for many biomarkers extend over several megabases, most of the EWAS signals were localized to one CpG site. The large GWAS regions reflect the large amount of LD in the genome, caused by genetic variants co-segregating during meiosis due to physical proximity (or low recombination rates in between), but it also reflect the high density of SNPs analyzed. In contrast, the correlation pattern in DNA methylation reflects the underlying regulatory properties of a chromosomal region. Two CpG sites, located in close proximity to one another, can have a completely different methylation pattern if one is located within a transcription factor-binding site in a promoter region, while the other is located outside the promoter. However, two CpG sites located close to each other in the same regulatory region are more likely to have a high correlation between their DNA methylation levels. A total of 57 of our significant CpG sites clustered in 20 regions with more than one EWAS signal. Out of these 57 CpG sites, 28 sites represent partially independent effects.

Epigenetic changes have previously been proposed to affect the pathogenesis of complex diseases [35]. We therefore evaluated the causal relation between genetic variants, DNA methylation and biomarker levels. We could see that SNPs identified in the GWAS, accounted for a significant fraction of the heritability of some biomarkers. However, we could not get any evidence for that DNA methylation explains any part of the heritability. We further showed that cis-regulatory SNPs for some of the biomarkers had a direct effect on DNA methylation levels at different locations of the genome. However, we found no evidence that DNA methylation has any direct effect on biomarker levels. Some associations between DNA methylation and biomarker levels were driven by environmental factors. For example, CpG sites in *F2RL3* and *AHRR* were associated with both IL-12 and/or WFDC2 levels. We have previously shown that DNA methylation at these CpG sites, as well as IL-12 levels, is strongly influenced by tobacco smoking [7,15]. By including smoking in our model, these associations were no longer significant, which suggests that tobacco smoking was confounding these associations. Many of the EWAS hits were also driven by underlying *cis*-regulatory SNPs and the associations were no longer significant when adjusting for the GWAS-SNPs. Such confounding, caused by SNPs or environmental factors, seem to be a major driver of associations between DNA methylation and biomarker levels. It could therefore be considered very likely that many of the remaining EWAS hits reflect different unmeasured health or lifestyle-related factors, such as immune system disorders or infections. Taken together, our results indicate that DNA methylation has a limited direct effect on biomarkers of complex diseases, and rather reflect the underlying pattern of genetic variants, specific environmental exposures, or represent secondary effects of the pathogenesis of disease.

One limitation of the study is that DNA methylation was measured in white blood cells and it is possible that analysis of other tissues could have generated different results, in particular for biomarkers that are not primarily expressed in blood cells. Another limitation may be the use of causal interference where direct experiments, with individuals randomized into be exposed or not be exposed to a specific biomarker, would have been preferred. However, in order to perform a similar study using direct experiments, one would have to use a large population of experimental animals and design experiments separately for each of the biomarkers and for each of the CpG sites. Exposing animals to biomarkers, by direct injections, and measure the short-term epigenetic response would be possible. However, there is no high-throughput method available for *in situ* alterations in DNA methylation levels and thus the influence of DNA methylation changes on biomarker levels cannot be tested. Therefore, the best way to address the causality between biomarkers and CpG sites is to use well-established statistical methods for causal inference as done in our study. It is also worth pointing out that defining an IV as the most significant SNP from a GWAS and by using the same cohort for defining IVs, as performing the MR, can lead to overestimation of the regression coefficients and over-fitting. However this will not influence the *P*-values in the causal inference but possibly the estimate of the causal effect, which should thereby be interpreted with care. Another limitation of the study is that samples were stored for several years prior to epigenetic and proteomics analyses. While DNA methylation appears to be stable over time, proteins are more sensitive to storage time [36]. In all our analyses we used a dummy variable to separate the samples that had been stored for different amount of time (year of sampling), and we also performed normalization to account for batch effects, which should eliminate the risk of introducing false positive associations. However, it is possible that some proteins have been partly degraded during storage, which could influence the detection frequency, as well as resulting in weaker associations with biomarker levels compared to using samples with shorter storage time.

Another limitation with the study might be the strict adjustment for multiple testing in the EWAS where we have used Bonferroni adjustment for the number of CpG sites analyses, rather than FDR adjustment that is commonly used in DNA methylation studies. This might have resulted in slightly fewer significant findings, but the probability of false positive results is reduced. Most of the tests are not independent due to the correlation between biomarkers, the LD between SNPs and the correlation between DNA methylation levels at nearby CpGs. We have therefore adjusted for the total number of SNPs and CpG sites analyzed, but not for the number of biomarkers included in the analyses. However, all our genome-wide significant EWAS hits were also significant using FDR (Benjamini-Hochberg) adjustment for all CpG sites and all biomarkers. We also observed inflation of low P-values in the EWAS for some of the biomarkers. Such inflation is not unexpected and is not comparable to inflation in a GWAS. Association between biomarker levels and in DNA methylation levels can reflect underlying environmental factors leading to a change in both markers. For example, an inflammatory response will be associated with increased levels of some inflammatory biomarkers. This is mediated by activating the expression of the genes, which is tightly linked to the DNA methylation level. Therefore, we would expect co-variation between biomarkers and DNA methylation at many regions in the genome, giving rise to inflation of low p-values. Finally, the use of cis-regulatory genetic variants as IVs in MR might is not completely resistant to pleiotropy [37]. It is possible that the cis-regulatory variant influences the expression of several genes and that the causal path from the SNP to the response variable is not (only) through the biomarker/CpG site under investigation. One way to overcome the risk of pleiotropy in MR studies is the inclusion of many independent IVs. However, due to the strong cis-regulatory effects on biomarker and DNA methylation levels, in combination with the LD pattern of the genome, it is not possible to find independent IVs for the variables investigated.

## Conclusions

We have integrated genetic and DNA methylation data to study associations and causal relationship between DNA methylation and protein abundance. For 36% (44/121) of the studied protein biomarkers, the abundance level was associated with DNA methylation, but for 52% these biomarkers (23/44), the associations were explained by genetic variants (Fig 4). For a subset of biomarkers, the association with DNA methylation was confounded by environmental factors (e.g., smoking), but for the majority of the associations, no such relationship could be found. Interestingly, for 22% of the proteins (N = 27), the abundance level was likely to affect DNA methylation patterns at different genomic loci based on the fact that their respective IVs were associated with DNA methylation levels (Fig 4). However, the opposite direction (DNA methylation IVs influencing biomarker levels) was not observed. These results does not indicate that DNA methylation plays a major direct role in the regulation of these disease biomarkers but that many of these biomarkers instead might exert their effects by affecting DNA methylation and thereby the expression of other genes.

## Methods

### Ethics statement

The NSPHS study was approved by the local ethics committee at the Uppsala University in compliance with the Declaration of Helsinki (Regionala Etikprövningsnämnden, Uppsala Dnr 2005:325) and extension of the project period was approved 2016-03-19 (same Dnr). All participants gave their written informed consent to the study. In case the participant was not full age, a legal guardian signed additionally. The procedure which was used to obtain informed consent and the respective informed consent form has been recently discussed in the light of present ethical guidelines [38].

### Study cohort NSPHS

Northern Sweden Population Health Study (NSPHS), the study cohort used in this project, was a health survey of the population in the parishes of Karesuando (year 2006) and Soppero (year 2009), county of Norrbotten. NSPHS was initiated to study the medical consequences of lifestyle and genetics. These parishes had about 3,000 inhabitants who met the eligibility criteria in terms of age (>14 years), of which 1,069 individuals participated in the study. More information about the NSPHS has been published previously [14]. For each participant in the NSPHS, blood samples were taken and immediately frozen and stored at −70 C. Genomic DNA for methylation and SNP analyses was extracted from previously frozen peripheral blood leukocytes using a phenol:chloroform protocol.

### Genetic data

DNA samples from the NSPHS individuals were genotyped using the Illumina HumanExome-12v1 and either Illumina Infinium HumanHap300v2.0 or Illumina HumanOmniExpress-12v1 bead microarrays [14]. Analysis of genotype raw data and quality control (QC) were performed using GenABEL package [39]. A total of 556,432 and 979,343 genotypes passed the QC in the samples collected in 2006 and 2009 respectively, and were used to impute unassayed genetic variants. Genotype data were imputed with a pre-phasing approach implemented in SHAPEIT version 2.5 (r790)) [40] and IMPUTE2 (version 2.3.2) [41] in the two sub-cohorts separately, using the 1000 Genomes Phase 3 integrated variant set (released October 2014) as the reference panel. Quality control (QC) were performed using GTOOL (v0.7.5) [42]. First, IMPUTE’s ‘info’ score >0.3 were required in both sub-cohorts prior to merging. The merged data were filtered using a Bonferroni-corrected Hardy–Weinberg cutoff of 0.05, combined info score >0.3 and a minor-allele frequency (MAF) > 0.0001, corresponding to at least one chromosome in the whole material. Only autosomal SNPs were included in the analyses. After QC of imputed autosomal data 11,901,484 SNPs and a total of 1,033 individuals remained (S2 Table). All genomic positions have been reported according to GRCh37.

### DNA methylation data

Genomic DNA for 743 samples that had passed the SNP QC (all samples that was collected in 2006 and a random selection of samples from the 2009 collection) was bisulfite-converted using an EZ DNA methylation Kit (ZYMO research) according to the manufacturer's recommendations. The methylation status of the genomic DNA was then assessed using the Human Methylation450 BeadChip, (Illumina, San Diego, USA) according to the standard protocol. Analysis of the raw data was performed using minfi. Normalization was done using Subset-quantile Within Array Normalisation (SWAN). A marker detection *P—*value ≤ 1.38*10^−10^ (Bonferroni adjusted *P* -value = 0.05, adjusted for the number of individuals * the number of CpG sites analyzed) was applied, a Probe Call rate of >0.98, and an individual call rate of >0.98 was used. A total of eight control samples were included: two positive controls, two negative controls, two duplicated samples that originated from the same blood samples and two duplicated samples that came from the same individual but blood samples were taken with three years in between as has been described previously [7]. After removing control samples, and two genetic outliers, 729 samples with DNA methylation data, which had also passed the QC in the SNP genotyping, remained for downstream analyses (S2 Table). Only autosomal sites were included in the analyses, leaving 473,864 sites post-QC. To ensure that the results were not influenced by variation in blood cell fractions between samples, we estimated the fraction of CD8T-, CD4T-, NK- and B-cells, monocytes and granulocytes in the study samples. This was done using the R package minfi [43] that allows for estimating cell fractions in Illumina 450K methylation data from whole blood. This method is based on the methylation data published for flow-sorted cells [44] and algorithms derived from the study by Houseman *et al*. [45]. Annotation of CpG site proximity to genes was provided by Illumina but we also validated these by comparing chromosomal positions of CpG sites with positions of human genes using build 37. A fraction of the probes, which are used to measure the DNA methylation on the Illumina 450K chips, have previously been suggested to cross-hybridize to different genomic loci [46] and these have been flagged in Supplemental Tables.

### Biomarkers

Directly after blood samples were taken, plasma was frozen and stored in aliquots at -70°C until central analysis. Biomarkers were measured in plasma for 1,004 randomly selected individuals, all of which had passed the SNP QC, using the Olink Proseek Multiplex Oncology I and CVD I ^96x96^ kit and quantified by real-time PCR using the Fluidigm BioMark™ HD real-time PCR platform as described earlier [15–17,47]. In brief, two oligonucleotide-labeled antibodies probes are bound to each biomarker. If the two probes are in close proximity a PCR target sequence is formed by a proximity-dependent DNA polymerization, which is quantified using standard real-time PCR. For cost efficiency, most of the biomarkers that overlap between the Oncology and CVD panel were only measured once with exception for Cystatin B and HB-EGF that were measured twice due to their associations with both cancer and CVD. The biomarkers were measured in batches (plates) of 92 individuals and 4 internal controls on each plate and normalized as described previously [15–17,47]. Due to systematic differences between plates, the plate number was included as a covariate (a factor) in all statistical analyses. In addition, all measurements for a given biomarker on a given plate were excluded if there were too many (>90%) individuals below the detection limit on a given plate. Individuals were excluded if at least one internal control was flagged as an outlier, if they were outliers regarding all biomarker measurements, or if >75% of the biomarker measurements were below the detection limit. Finally, we removed all biomarkers with a post-QC detection rate below 20%, which is a reasonable cutoff since many biomarkers are only expected to be detected in individuals with a disease related condition. After all QC steps, 121 biomarkers and 961 individuals (S2 Table) remained for downstream analyses. All individual measurements that did not pass QC were set to missing, meaning also that all measurements below detection limit were removed prior to statistical analyses. The measured values for biomarkers were then rank transformed to normality (mean = 0 and standard deviation = 1), using the rntransform function in GenABEL [48] and adjusted for sex, age, body-mass index (bmi), year of collection and plate number (as a factor).

### Statistical analysis—GWAS for biomarker levels

Analyses were performed using GenABEL [48] or using ProbABEL [49], including 961 individuals with SNP and biomarker data. We used a polygenic model in GenABEL to adjust for relatedness among individuals by utilizing a genetic kinship matrix. The kinship matrix was estimated using the ibs function in GenABEL including genotyped autosomal SNPs (MAF > 0.05) that overlap between the two different genotyping chips. The inverse of the variance-covariance matrix, and the residuals from the polygenic models were exported as input in the GWA analyses. The GWA analyses was performed by palinear function in ProbABEL using the -mmscore option, with a dummy variable separating samples genotyped with two different genotyping arrays as the only covariate. All biomarkers were already adjusted for relevant covariates as part of the QC. In the GWAS we primarily used a P-value cutoff of 0.05 / the total number of SNPs tested. However, we also indicate which associations would pass a threshold if also adjusting for the number of biomarkers tested using a threshold of 5e-8 (the standard GWAS P-value cutoff) divided by the number of biomarkers analyzed (N = 121) resulting in a P-value cutoff of 4.1e-10. QQ-plots and Manhattan plots were produced using qqman [50].

### Statistical analysis—EWAS for biomarker levels

All statistical analysis and plots were carried out in R [51]. A total of 729 individuals passed QC for DNA methylation and out of these, 698 also had post-QC biomarker data available (S2 Table) and were included in the EWAS analyses. The DNA methylation levels (*aka*. beta-values) were adjusted for sex, age, batch and plate effects, year of sampling and cell fractions (CD8T-, CD4T-, NK- and B-cells, monocytes and granulocytes) using a linear model. Adjusted biomarkers were compared to adjusted methylation levels using the polygenic model implemented in the GenABEL package [48], which adjust for relatedness among individuals using a genetic kinship matrix. Biomarkers were used as response variables and methylation levels as explanatory variables. The estimates were extracted directly from the polygenic model, and *P*-values were estimated by comparing the polygenic model to a null model where the explanatory variable (DNA methylation) was removed. Independency between EWAS significant CpG sites that were located in the same region (within 2Mb from each other) and that were associated with the same biomarker was investigated. This was done by including the most significant CpG site as a covariate in the null model and compare the null model to an alternative model where the most significant and one additional CpG site from the region was included. The P-values for the additional CpG sites were retrieved from the chi2 distribution and chi2 values were calculated as the difference in twice the negative maximum log-likelihood between the alternative and the null models. If and additional significant CpG sites were detected (P < 0.05), this CpG site was also included in the null model and the procedure was repeated until no additional CpG sites within the region were significant. State of the art in EWAS studies is to use FDR adjustment for multiple testing. We have been somewhat stricter in adjusting for multiple testing and the results presented from the GWAS are the ones that have FDR Q-values < 0.05 when adjusting for all CpG sites and all biomarkers. However, we also applied a per biomarker Bonferroni threshold that takes into account the number of CpG sites tested for each biomarker, resulting in a somewhat more conservative threshold of P = 1.06e-07 compared to 1.97e-07.

### Statistic analyses—integrated analyses and heritability estimation

We also tested if significant DNA methylation–biomarker associations could be explained by (caused by) underlying SNPs for all biomarkers for which an EWAS and a GWAS hit was identified. We computed the squared marginal correlation coefficient between the biomarker (Y) and the DNA methylation level (X) and the squared partial correlation coefficient conditioning on the SNPs (adjusting for the same covariates and using the same transformation as above). In these analyses, only individuals with data for biomarkers, DNA methylation and SNPs were included (N = 698). The association was considered to be fully explained if X and Y was not correlated when conditioning on the SNPs (*P* > 0.05/number or tests performed), partially explained if X was correlated with Y after conditioning on the SNPs (*P* ≤ 0.05/number or tests performed), but the *P*-value did not meet the threshold for genome-wide significance used to identify the association in the first place (*P* > 0.05/473,864). If *P* ≤ 0.05/473,864 in the conditional analyses, the association was considered not to be explained by an SNP. Heritability estimates for the biomarkers were calculated using the polygenic function in GenABEL [39] using a maximum likelihood. Biomarkers were first adjusted for covariates and normalized as in previous analyses, and year of sampling was included as a covariate in the polygenic model. The polygenic function maximizes the likelihood of the data under polygenic model to test for the most likely estimate the heritability of a trait. To retrieve test statistics for the heritability estimates, a null model (with heritability set to zero) was compared to the alternative model (with the most likely heritability estimate). *P*-values were retrieved from the chi2 distribution and chi2 values were calculated as the difference in twice the negative maximum log-likelihood between the model when heritability is optimized and the model where heritability is fixed to a set value (e.g. zero to test if the heritability differs from zero). To test if a SNP or DNA methylation level can explain a significant part of the heritability, the null model (when heritability is optimized) was compared to the alternative model (when heritability is optimized with additional covariates in form of SNPs or DNA methylation levels).

### Statistic analyses—Mendelian Randomization

We used Mendelian Randomization (MR) to determine if DNA methylation was directly affecting variation in biomarker levels, or if biomarkers themselves were affecting the variation in DNA methylation, and to estimate the magnitude of causal effects. MR uses genetic variants associated with a phenotype (X) as an instrumental variable (IV) to test for causal effects of X on a second phenotype (Y) (Fig 5). To be a valid instrument three criteria must be fulfilled [52]: IV criterion 1) the genetic variant must not be associated with an unknown confounding factor (U1) that influences Y, and IV criterion 2) the genetic variant must not be directly associated with Y and IV criterion 3) the genetic variant should be directly associated with X. To fulfill the first criterion, only genetic variants that are *directly* associated with X were considered as IVs. These variants were selected as *cis*-regulatory genetic variants that directly regulate the expression of the protein biomarker or the DNA methylation level and therefore, the risk of pleiotropic effects, where the genetic variant is associated with an unknown confounding factor that influences Y, is minimized. Criterion 2 above implies that we cannot make any causal inference between biomarkers and CpG sites that are located within the same loci and regulated by the same genetic variants. If there is a causal relation identified between X and Y we can use MR to estimate the effect.

The causal inference was performed in two ways. Firstly, the DNA methylation level was set as the explanatory variable (X) and the biomarker level to the response variable (Y), using the most significant *cis*-regulatory meQTL as the IV for all EWAS significant CpG sites where such variant was identified. Secondly, the biomarker level was set as the causal variable (X) and the DNA Methylation level as the response variable (Y), then using the SNPs being *cis*-regulatory in relation to the gene encoding respective biomarker as the IVs. For DNA methylation levels only the most significant SNP for each CpG site was included as IV in the causal inference, but for biomarker levels all independent SNPs from the GWAS were used as IVs. The F-statistics was used to estimate the strength of the IVs [52] according to Eq 1, where *R*^*2*^ is the squared correlation coefficient between the IV and the explanatory phenotype (X in Fig 5), *n* is the sample size, and *k* the number of instruments. Since we were using mainly single SNPs as IVs, *k* was set to 1 for most tests, except for biomarkers with more that one independent GWAS hit.

$$F=\left(\frac{n-k-1}{k}\right)\left(\frac{R^{2}}{1-R^{2}}\right)$$ (Eq 1)

The causal inference was performed using the Two-Stage Least Squares (2SLS) regression analysis. As in the GWAS and EWAS analyses, we first regressed out all the variation due to the covariates, both for the explanatory variable (*X*) and the response variable (*Y*). We then considered the linear regression model:

$$Y=\beta_{0}+\beta_{1}X+\varepsilon$$ (Eq 2)

We assume that *ε* is normally distributed with zero mean. To obtain the estimated value $\hat{X}$ we regressed *X* on the instrumental variables *(IV*) for each *X*:

$$X=\gamma_{0}+\gamma_{1}{IV}_{1}+\gamma_{2}{IV}_{2}+\gamma_{3}{IV}_{3}+\nu$$ (Eq 3)

to estimate the regression coefficients *γ*_0_,*γ*_1_,*γ*_2_,*γ*_3_. Subsequently, we calculated the predicted values of *X* such that:

$$\hat{X}=GS={\hat{\gamma }}_{0}+{\hat{\gamma }}_{1}{IV}_{1}+{\hat{\gamma }}_{2}{IV}_{2}+{\hat{\gamma }}_{3}{IV}_{3}$$ (Eq 4)

derived from Eq 3, and pasted $\hat{X}$ into the original linear regression model (Eq 2):

$$Y=\beta_{0}+\beta_{1}\hat{X}+\omega ,$$ (Eq 5)

Here, *ω* is a composite error term that is uncorrelated with $\hat{X}$. For biomarkers with only one IV and DNA methylation levels, only one IV was included in Eqs 4 and 5. The predicted value of *X*, $\hat{X}$, commonly referred to as a genetic score (GS) in genetic studies, represents the genetically determined level of a biomarker, of DNA methylation level at a CpG site. A final test of the significance of *β*_1_ (the MR estimate) was performed, adjusting for relatedness among individuals using the polygenic model in GenABEL, as described above.

### Statistical analysis—cis-regulatory meQTL

For all CpG sites identified in the EWAS, we performed association analyses to identify *cis*-regulatory meQTL (SNPs influencing the DNA methylation level) and to estimate the $\hat{\gamma }$ values to be inserted into Eq 4. All SNPs within a one-megabase region flanking each CpG site were considered as being *cis*-regulatory and included in the analyses. DNA methylation is known to be highly heritable, mainly due to underlying SNPs. In such situation, with a more or less monogenetic phenotypes, the variance explained by the relatedness among individuals is very similar to the variance explained by one single SNP. Using the polygenic model (as in our previous analyses) will then remove all of the phenotypic variance that is due to that SNPs. Consequently, adjusting the methylation levels for relatedness was not desirable. Therefore, DNA methylation levels were adjusted for the same covariates as in the EWAS and analyzed using the linear model for association analyses implemented in the palinear function in ProbABEL [49]. Since different chromosomes were analyzed for each CpG site, resulting in a different number of tests performed, a general GWAS cut-off for statistical significance of 5 x 10^−8^ was used and a 0.01 minor allele frequency cut-off.

## Supporting information

S1 Fig Relationship tree that clusters similar biomarkers based upon expression values (A), and principal component plot for the biomarkers (B).
(PDF)

S2 Fig Manhattan plots and QQ plots for GWAS results for all analyzed biomarkers. (PDF)

S3 Fig Manhattan plots and QQ plots for EWAS results for all analyzed biomarkers. (PDF)

S4 Fig Manhattan plots for EWAS results when adjusted for independent GWAS SNPs. A total of 18 biomarkers with both EWAS and GWAS hits are included.
(PDF)

S5 Fig Manhattan plots for comparison of the results. Results from the different analyses are shown as: A) the primary EWAS, B) primary GWAS, C) EWAS adjusted for independent GWAS SNPs, and D) EWAS for GS, calculated from *cis*-regularity SNP, that influence biomarker levels.
(PDF)

S6 Fig Allelic effects on biomarker levels. The X-axis represents the weighted (by the regression coefficients) allelic effects by the top GWAS SNPs and the Y–axis is the measured biomarker level for each individual. For biomarkers with more than one independent SNP, all independent *cis*-SNPs are included, and N indicates the number of SNPs included. R-squared is the fraction of the variance in biomarker levels explained by all (N) variants. For biomarkers with N = 1, most individuals are in either of three clusters that correspond to individuals being homozygous for the minor or major allele or individuals being heterozygous. Dosage values for the imputed genotypes are used, and therefore, some individuals are located outside the three clusters. For biomarkers with more than one SNP included as IVs; more than three clusters are observed which agrees with the higher number of possible allele-combinations.
(PDF)

S7 Fig EWAS for genetic scores (GS). GS are calculated for biomarkers with *cis*-regulatory SNPs only.
(PDF)

S1 Table Summary of the analyzed biomarkers. (XLSX)

S2 Table Number of individuals in each omics-dataset (post-QC [pre-QC]) and the overlap between pairwise omics-datasets analyzed, basic characteristics, disease prevalence and co-morbidity. (XLSX)

S3 Table Primary GWAS signals. (XLSX)

S4 Table GWAS regions with significant hits. (XLSX)

S5 Table Secondary GWAS signals, i.e. signals after correcting for the most significant primary signal for each biomarker. (XLSX)

S6 Table Tertiary GWAS signals, i.e. signals after correcting for the most significant primary and secondary signal for each biomarker. (XLSX)

S7 Table List of 188 CpG-sites (N = 169 unique) significantly associated with different biomarkers (N = 44). (XLSX)

S8 Table Results from the tests of independent effects of CpG-sites in the same chromosomal region (within two megabases from each other) (XLSX)

S9 Table Biomarkers with significant EWAS and GWAS hits. (XLSX)

S10 Table EWAS significant CpG sites after adjusting for the GWAS SNPs. Only biomarkers with overlapping EWAS and GWAS signal are included.
(XLSX)

S11 Table Heritability estimates for biomarkers prior to, and after adjusting for associated SNPs and DNA methylation at associated CpG sites. (XLSX)

S12 Table Biomarkers and SNPs used in the Mendelian Randomization (MR). (XLSX)

S13 Table EWAS significant findings for genetically determined biomarker levels. (XLSX)

S14 Table CpG sites with a significant SNP influencing the methylation level (meQTL), and the association between the meQTL and its respective biomarker. (XLSX)
