# Supplementary material for: The relative contribution of DNA methylation and genetic variants on protein biomarkers for human diseases
Source: PLoS Genet. 2017 Sep 15;13(9):e1007005. doi: 10.1371/journal.pgen.1007005 (PMC5617224; doi:10.1371/journal.pgen.1007005)
Supplement: S1 Fig — Relationship tree that clusters similar biomarkers based upon expression values (A), and principal component plot for the biomarkers (B). (PDF) [file pgen.1007005.s001.pdf]

**S1 Fig.** A) Relationship tree, drawn for the biomarkers in this study, which clusters similar - biomarkers based upon expression values. B) Principal component plot for the biomarkers in the study.

A

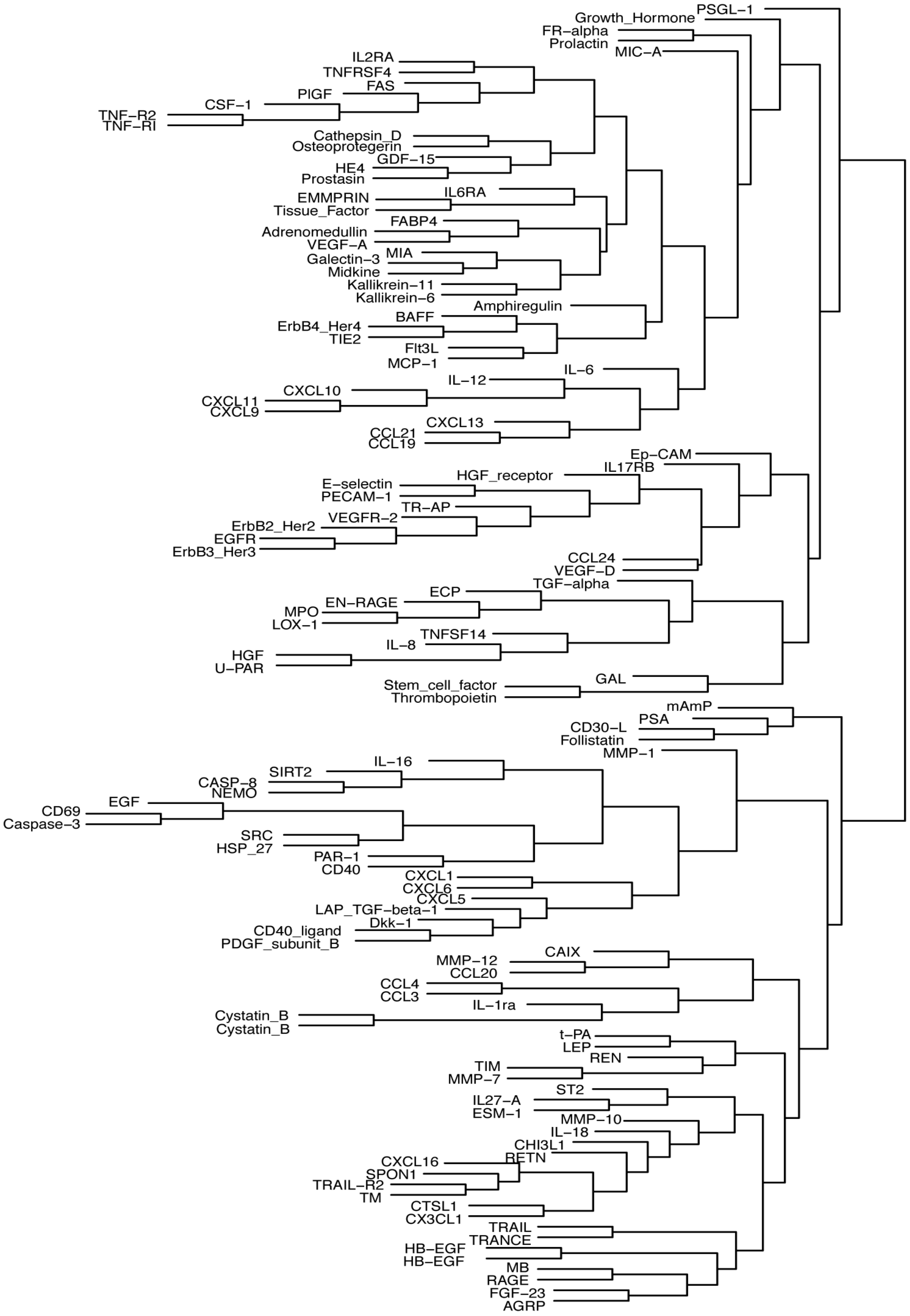

B

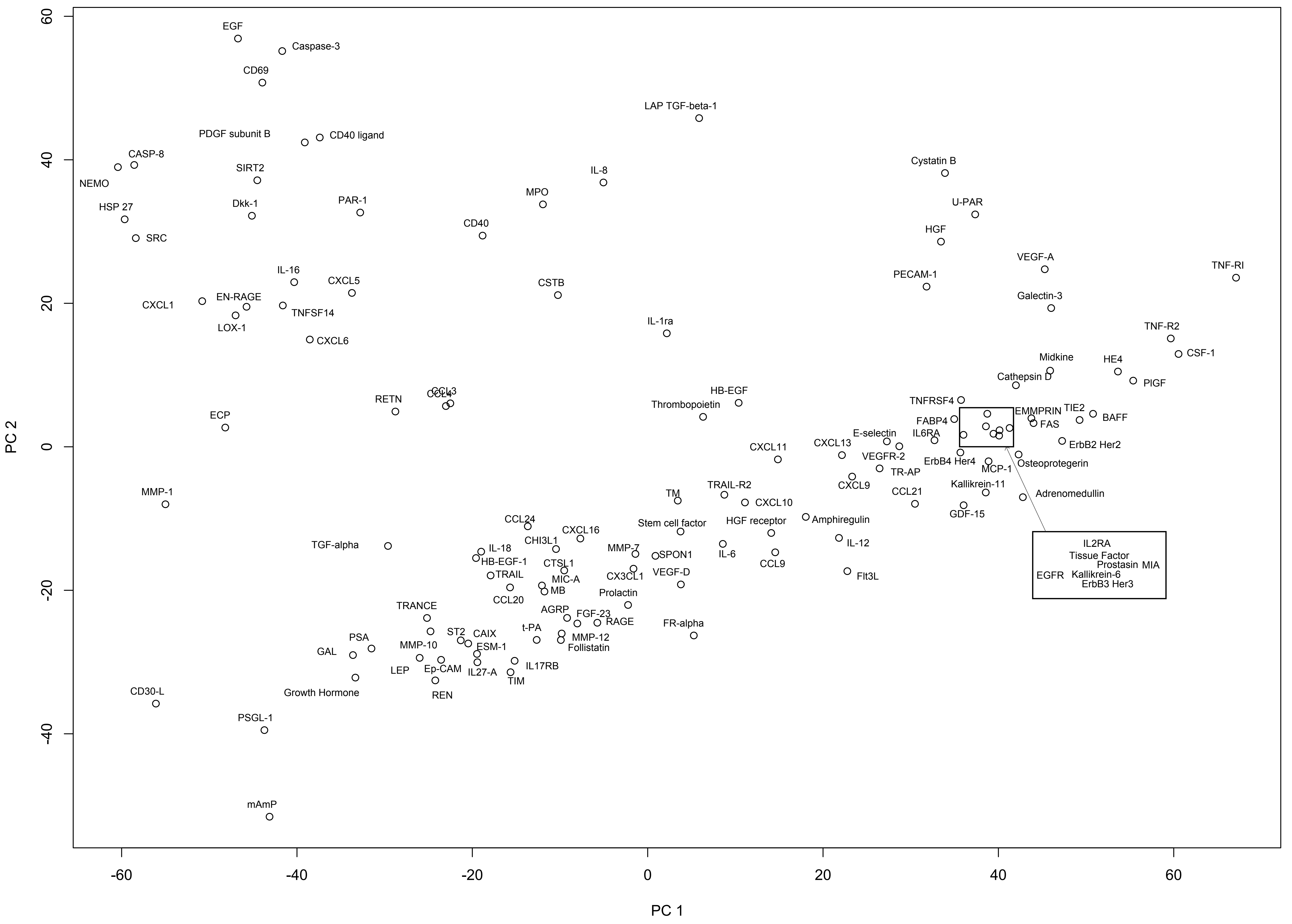

EGF
Caspase-3
CD69
LAP TGF-beta-1
PDGF subunit B
CD40 ligand
CASP-8
NEMO
SIRT2
IL-8
Cystatin B
HSP 27
Dkk-1
PAR-1
MPO
U-PAR
SRC
CD40
HGF
IL-16
CXCL5
CSTB
VEGF-A
PECAM-1
TNF-RI
CXCL1
EN-RAGE
TNFSF14
LOX-1
Galectin-3
IL-1ra
TNF-R2
CXCL6
CSF-1
Midkine
HE4
Cathepsin D
PlGF
RETN
CCL3
CCL4
HB-EGF
TNFRSF4
Thrombopoietin
ECP
EMMPRIN
TIE2
BAFF
FABP4
FAS
E-selectin
IL6RA
CXCL11
CXCL13
ErbB2 Her2
VEGFR-2
ErbB4 Her4
Osteoprotegerin
TR-AP
MCP-1
CXCL9
Kallikrein-11
MMP-1
TRAIL-R2
TM
CCL21
Adrenomedullin
CXCL10
GDF-15
CCL24
Amphiregulin
Stem cell factor
HGF receptor
CXCL16
TGF-alpha
CHI3L1
IL2RA
IL-18
MMP-7
IL-12
Tissue Factor
SPON1
IL-6
Prostasin
MIA
HB-EGF-1
CTSL1
CCL9
TRAIL
VEGF-D
EGFR
Kallikrein-6
CX3CL1
MIC-A
Flt3L
ErbB3 Her3
MB
CCL20
Prolactin
TRANCE
AGRP
FGF-23
RAGE
FR-alpha
PSA
ST2
CAIX
t-PA
MMP-12
MMP-10
ESM-1
GAL
Follistatin
LEP
Ep-CAM
IL17RB
IL27-A
TIM
Growth Hormone
REN
CD30-L
PSGL-1
mAmP
PC 2
PC 1
60
40
20
0
-20
-40
-60
