# Supplementary material for: The relative contribution of DNA methylation and genetic variants on protein biomarkers for human diseases
Source: PLoS Genet. 2017 Sep 15;13(9):e1007005. doi: 10.1371/journal.pgen.1007005 (PMC5617224; doi:10.1371/journal.pgen.1007005)
Supplement: S2 Fig — (PDF) [file pgen.1007005.s002.pdf]

Supplemental Fig S2*. Manhattan plots and QQ plots for GWAS results for all analyzed biomarkers.*

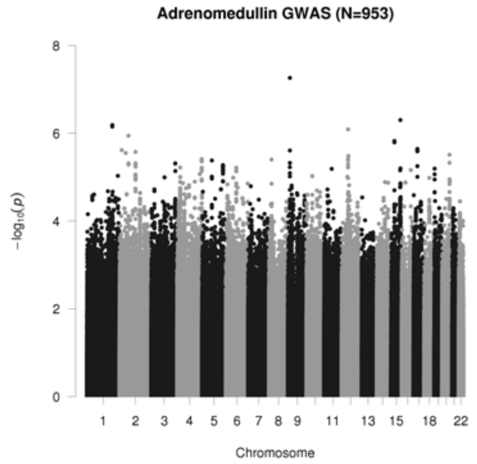

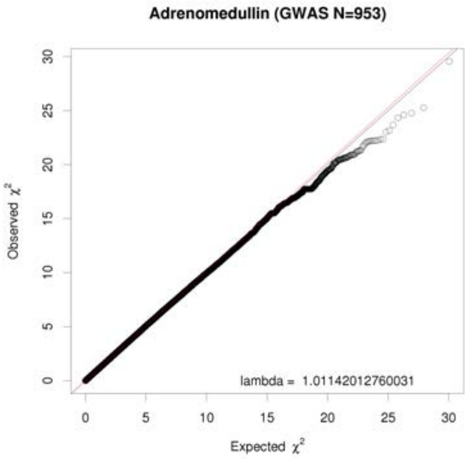

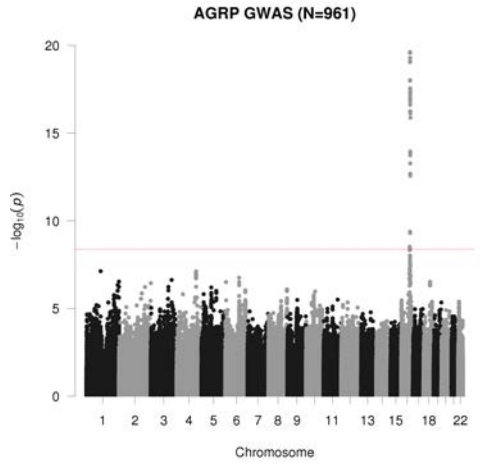

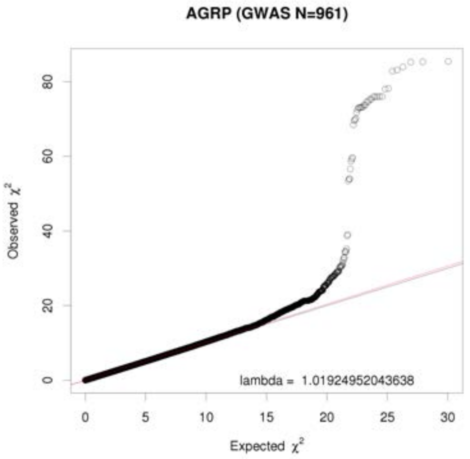

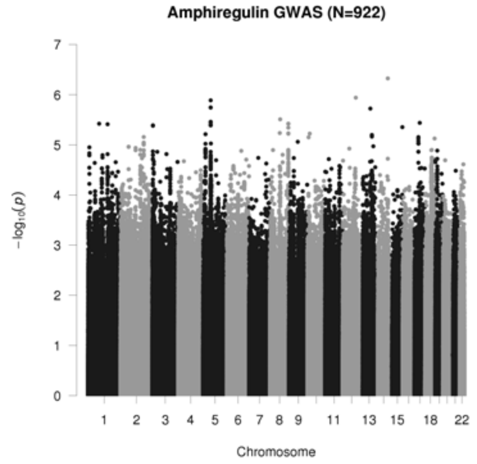

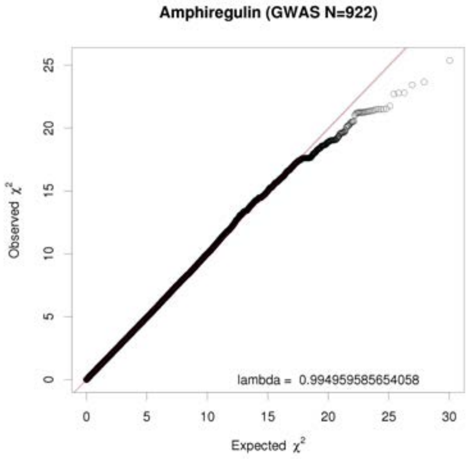

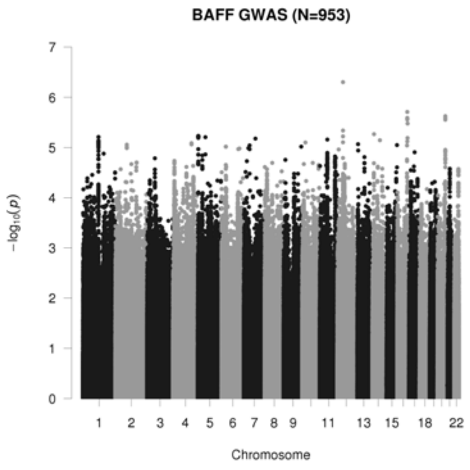
BAFF GWAS (N=953)
Chromosome
$-\log_{10}(p)$

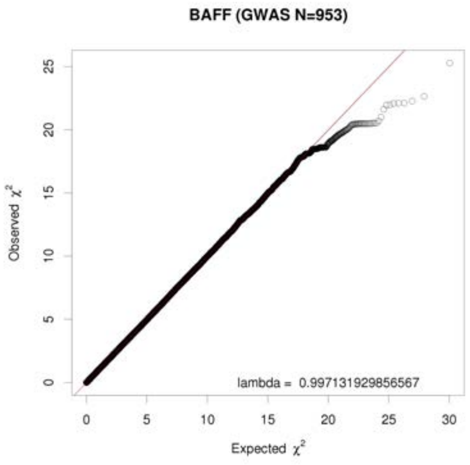
BAFF (GWAS N=953)
Observed χ^2
Expected χ^2
lambda = 0.997131929856567

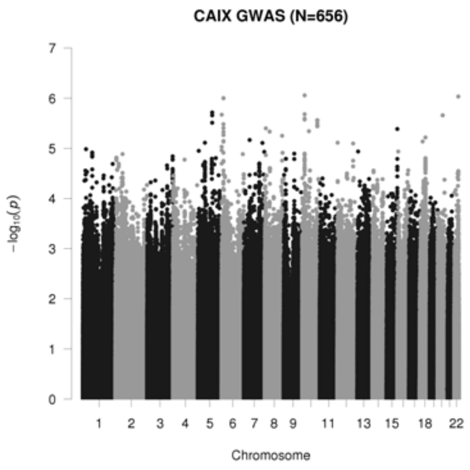
CAIX GWAS (N=656)
Chromosome
$-\log_{10}(p)$

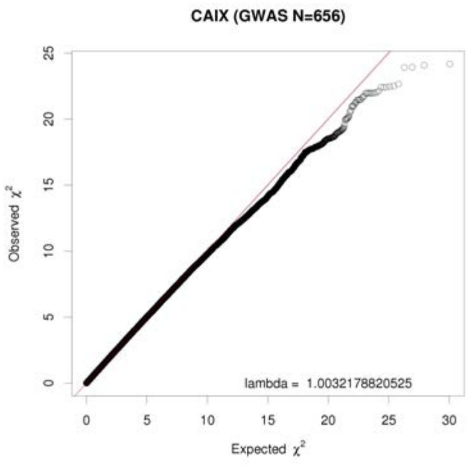
CAIX (GWAS N=656)
Observed χ^2
Expected χ^2
lambda = 1.0032178820525

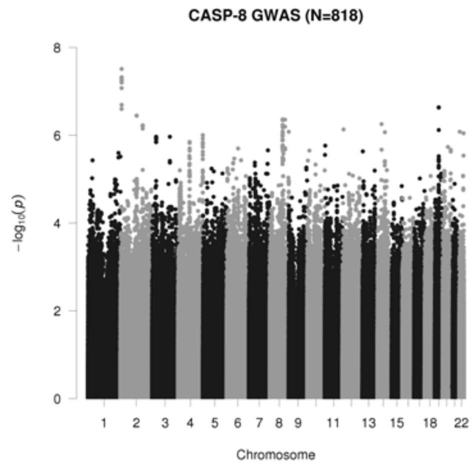
CASP-8 GWAS (N=818)
Chromosome
$-\log_{10}(p)$

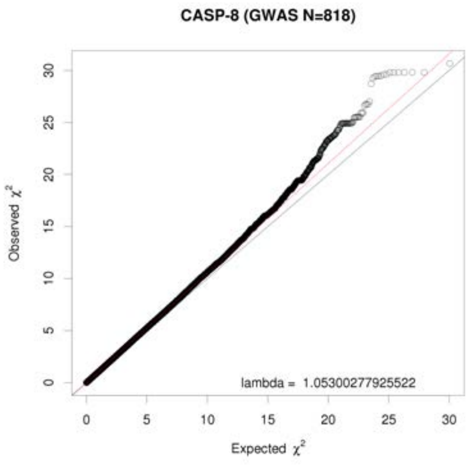
CASP-8 (GWAS N=818)
Observed χ^2
Expected χ^2
lambda = 1.05300277925522

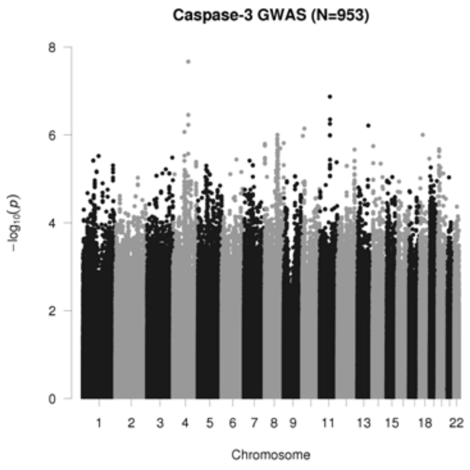
Caspase-3 GWAS (N=953)
$-\log_{10}(p)$
Chromosome

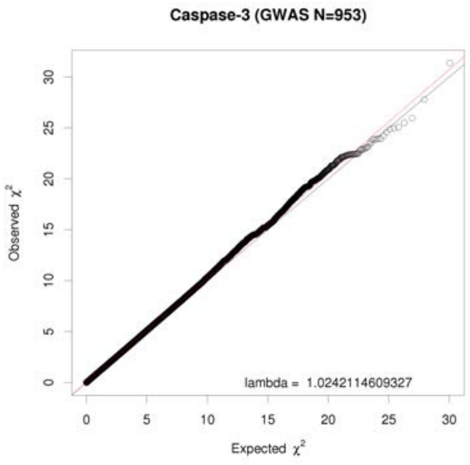
Caspase-3 (GWAS N=953)
Observed χ^2
Expected χ^2
lambda = 1.0242114609327

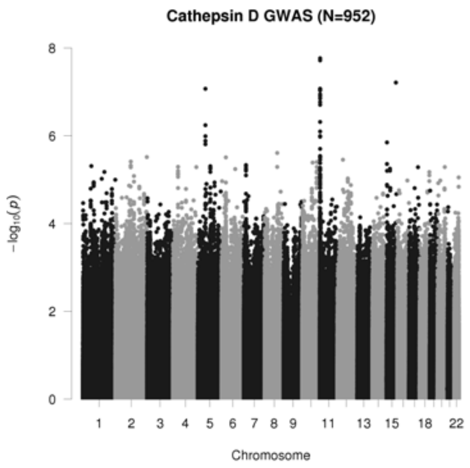
Cathepsin D GWAS (N=952)
$-\log_{10}(p)$
Chromosome

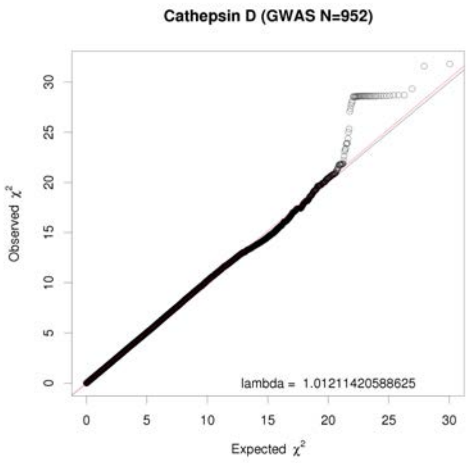
Cathepsin D (GWAS N=952)
Observed χ^2
Expected χ^2
lambda = 1.01211420588625

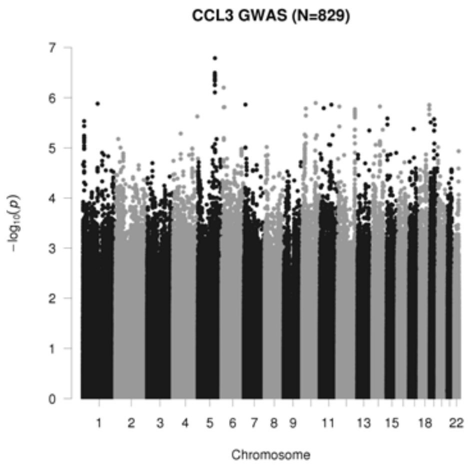
CCL3 GWAS (N=829)
$-\log_{10}(p)$
Chromosome

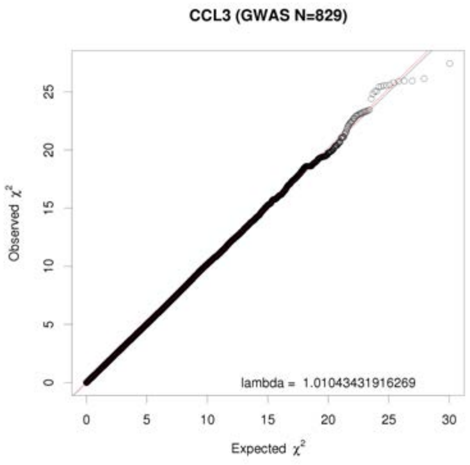
CCL3 (GWAS N=829)
Observed χ^2
Expected χ^2
lambda = 1.01043431916269

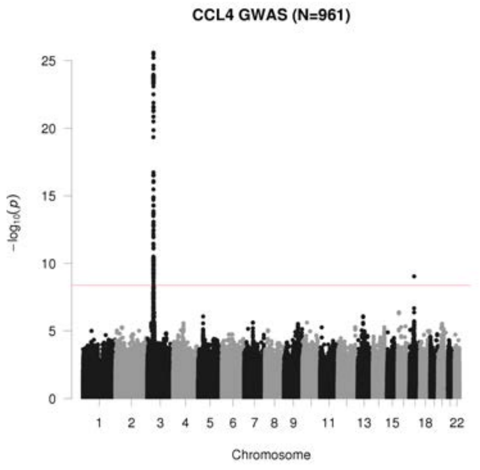
CCL4 GWAS (N=961)
$-\log_{10}(p)$
Chromosome

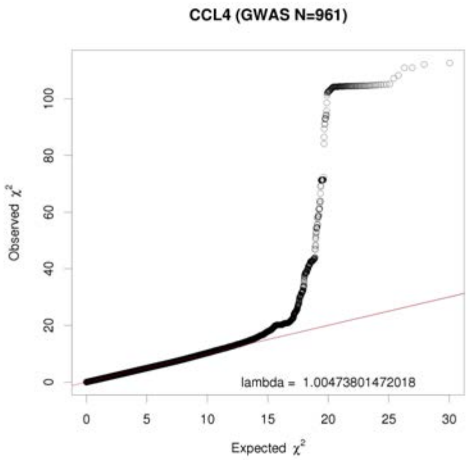
CCL4 (GWAS N=961)
Observed χ^2
Expected χ^2
lambda = 1.00473801472018

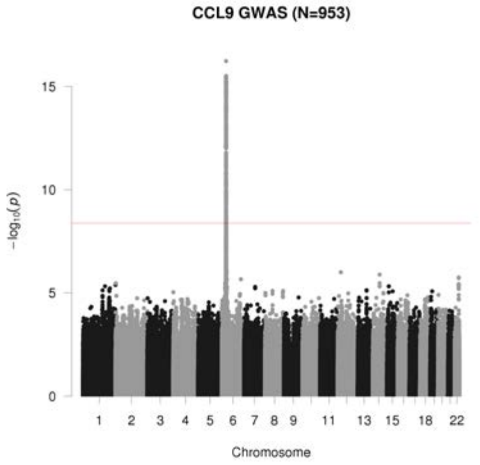
CCL9 GWAS (N=953)
$-\log_{10}(p)$
Chromosome

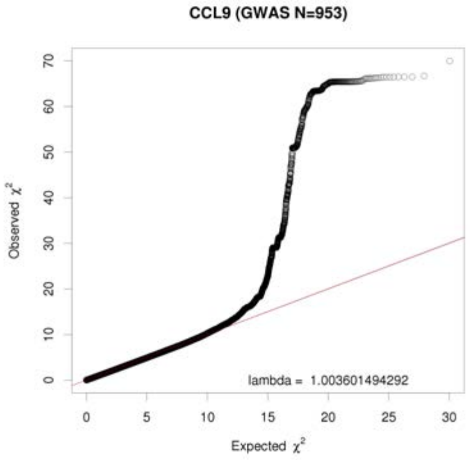
CCL9 (GWAS N=953)
Observed χ^2
Expected χ^2
lambda = 1.003601494292

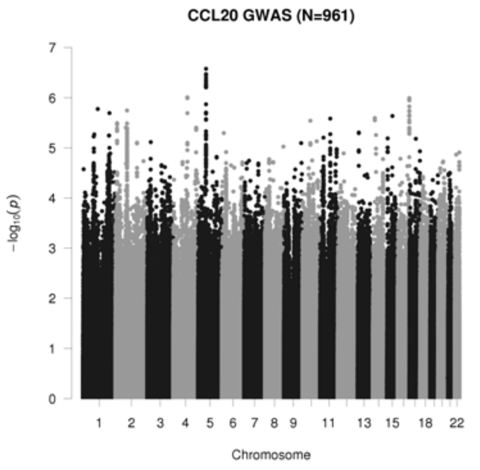
CCL20 GWAS (N=961)
$-\log_{10}(p)$
Chromosome

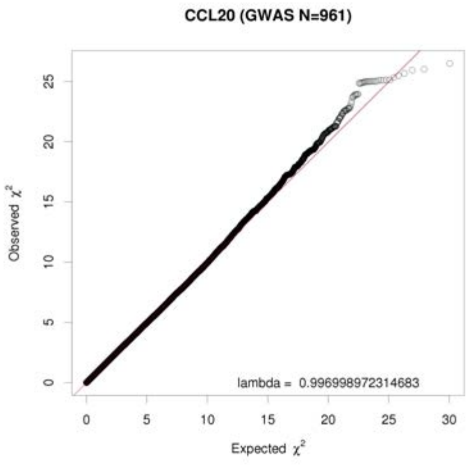
CCL20 (GWAS N=961)
Observed χ^2
Expected χ^2
lambda = 0.996998972314683

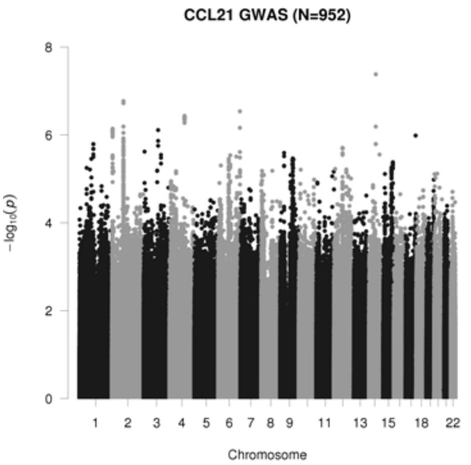
CCL21 GWAS (N=952)
$-\log_{10}(p)$
0 2 4 6 8
1 2 3 4 5 6 7 8 9 11 13 15 18 22
Chromosome

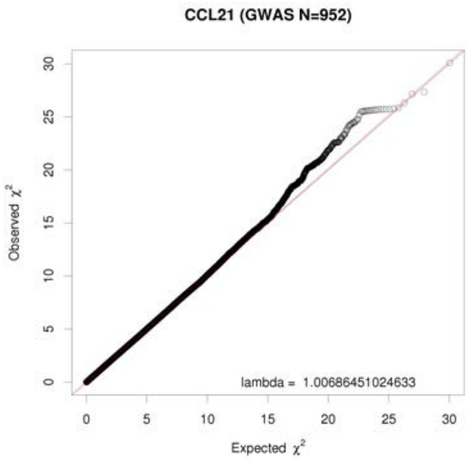
CCL21 (GWAS N=952)
Observed χ^2
Expected χ^2
0 5 10 15 20 25 30
lambda = 1.00686451024633

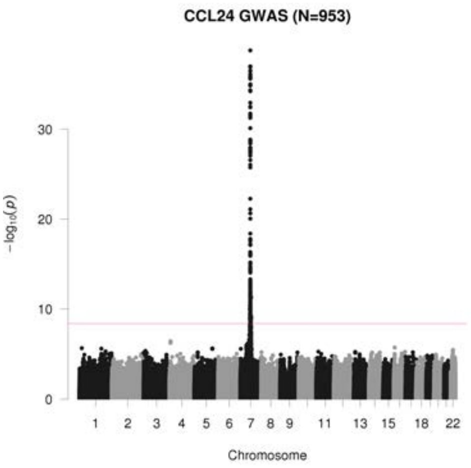
CCL24 GWAS (N=953)
$-\log_{10}(p)$
0 10 20 30
1 2 3 4 5 6 7 8 9 11 13 15 18 22
Chromosome

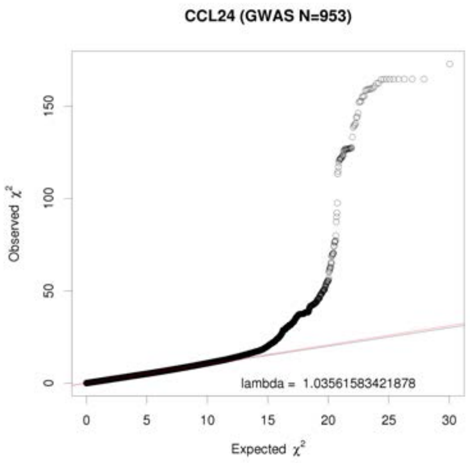
CCL24 (GWAS N=953)
Observed χ^2
0 50 100 150
Expected χ^2
0 5 10 15 20 25 30
lambda = 1.03561583421878

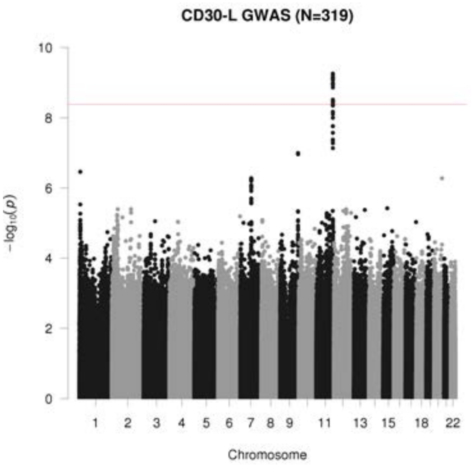
CD30-L GWAS (N=319)
$-\log_{10}(p)$
0 2 4 6 8 10
1 2 3 4 5 6 7 8 9 11 13 15 18 22
Chromosome

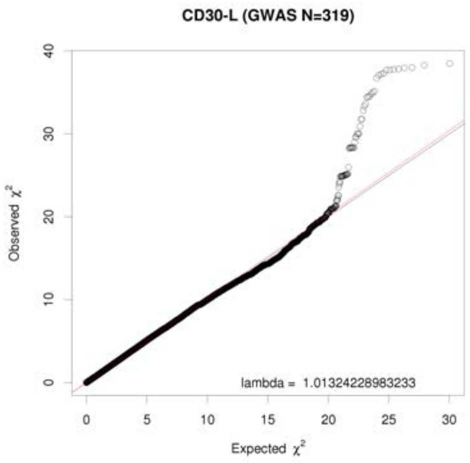
CD30-L (GWAS N=319)
Observed χ^2
0 10 20 30 40
Expected χ^2
0 5 10 15 20 25 30
lambda = 1.01324228983233

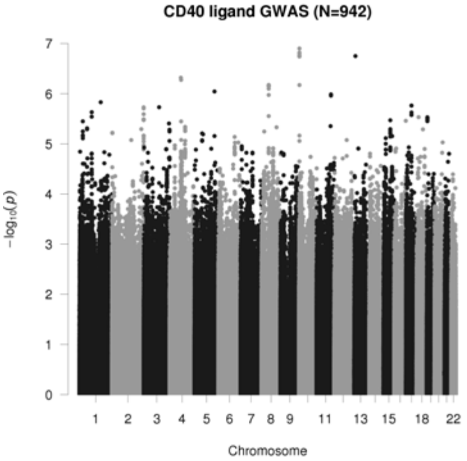
CD40 ligand GWAS (N=942)
$-\log_{10}(p)$
Chromosome

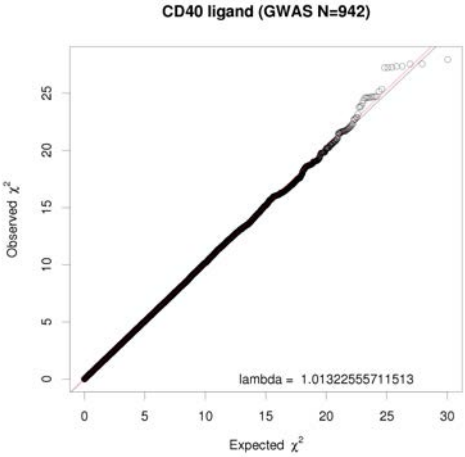
CD40 ligand (GWAS N=942)
Observed χ^2
Expected χ^2
lambda = 1.01322555711513

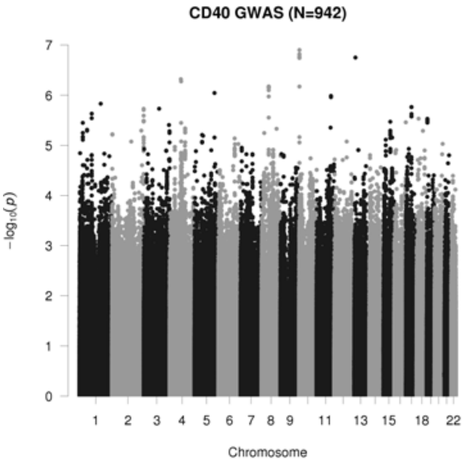
CD40 GWAS (N=942)
$-\log_{10}(p)$
Chromosome

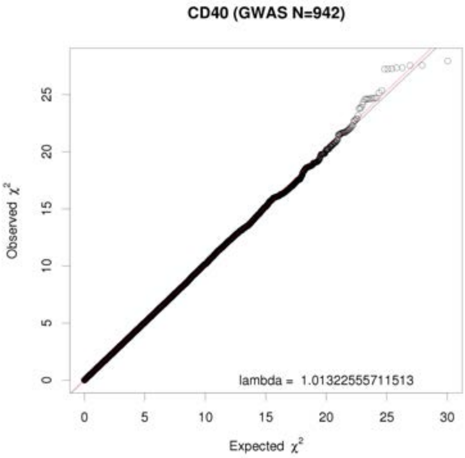
CD40 (GWAS N=942)
Observed χ^2
Expected χ^2
lambda = 1.01322555711513

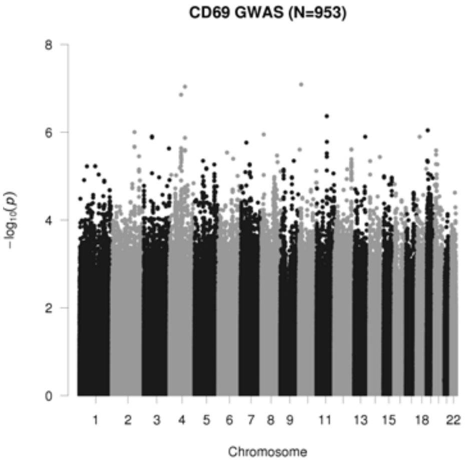
CD69 GWAS (N=953)
$-\log_{10}(p)$
Chromosome

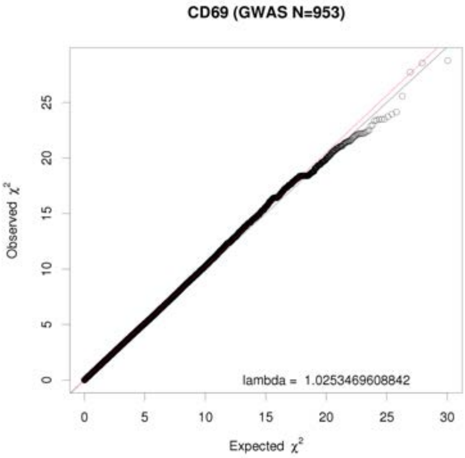
CD69 (GWAS N=953)
Observed χ^2
Expected χ^2
lambda = 1.0253469608842

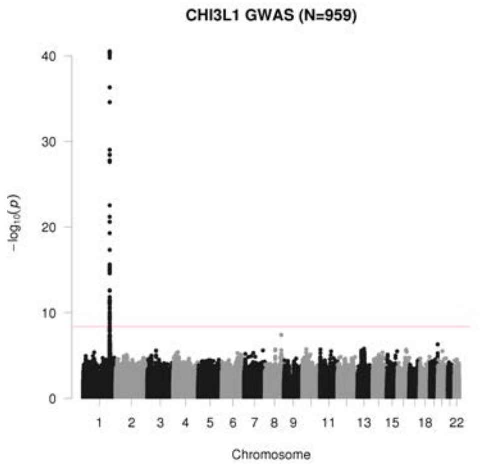
CHI3L1 GWAS (N=959)
$-\log_{10}(p)$
0 10 20 30 40
1 2 3 4 5 6 7 8 9 11 13 15 18 22
Chromosome

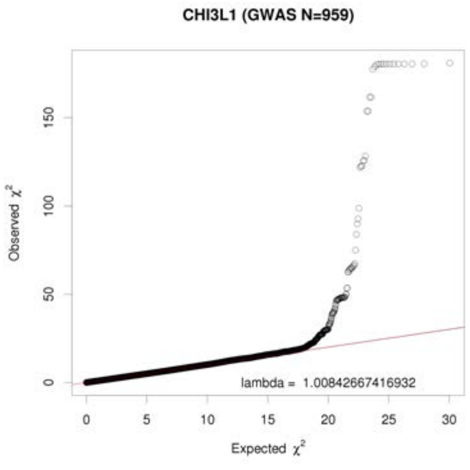
CHI3L1 (GWAS N=959)
Observed χ^2
0 50 100 150
lambda = 1.00842667416932
0 5 10 15 20 25 30
Expected χ^2

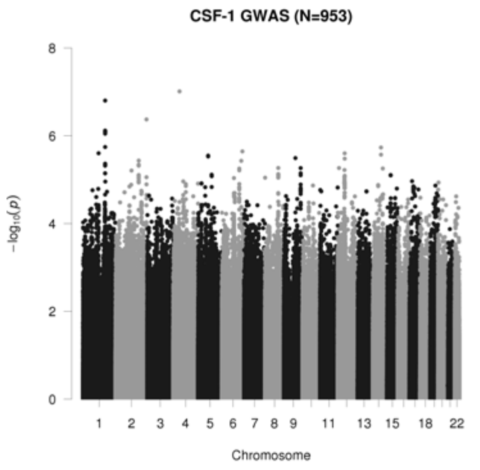
CSF-1 GWAS (N=953)
$-\log_{10}(p)$
0 2 4 6 8
1 2 3 4 5 6 7 8 9 11 13 15 18 22
Chromosome

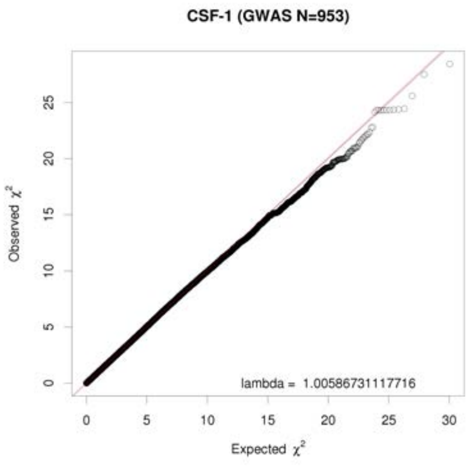
CSF-1 (GWAS N=953)
Observed χ^2
0 5 10 15 20 25
lambda = 1.00586731117716
0 5 10 15 20 25 30
Expected χ^2

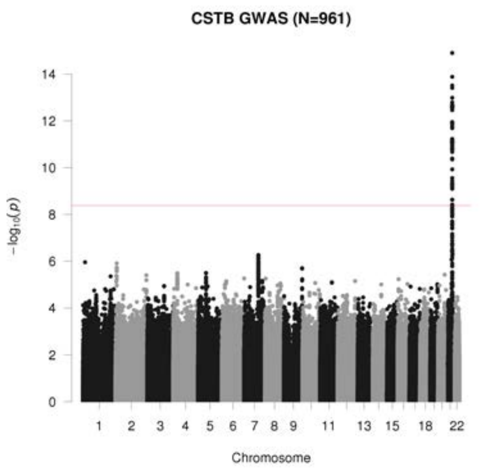
CSTB GWAS (N=961)
$-\log_{10}(p)$
0 2 4 6 8 10 12 14
1 2 3 4 5 6 7 8 9 11 13 15 18 22
Chromosome

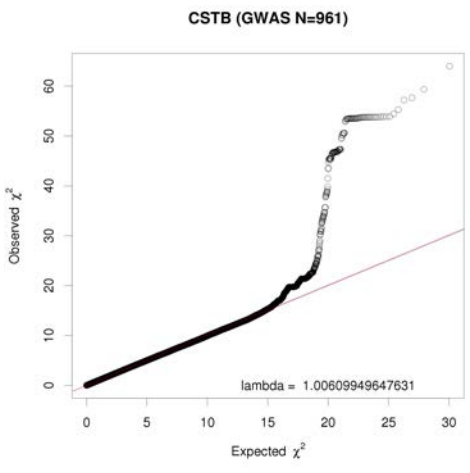
CSTB (GWAS N=961)
Observed χ^2
0 10 20 30 40 50 60
lambda = 1.00609949647631
0 5 10 15 20 25 30
Expected χ^2

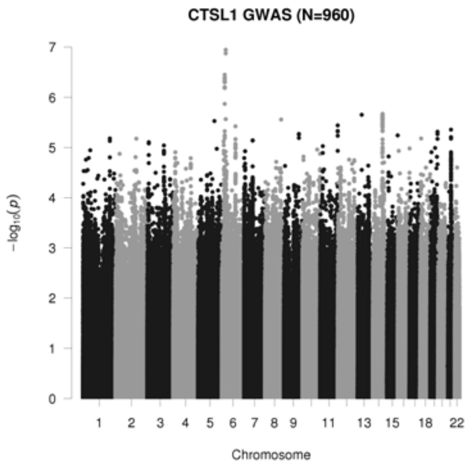
CTSL1 GWAS (N=960)
$-\log_{10}(p)$
Chromosome

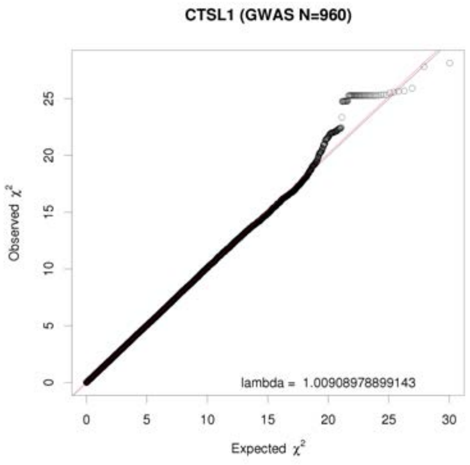
CTSL1 (GWAS N=960)
Observed χ^2
Expected χ^2
lambda = 1.00908978899143

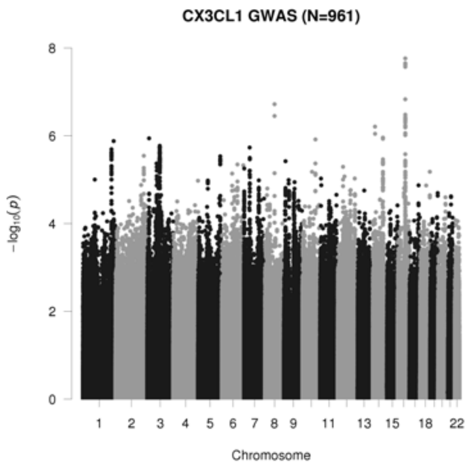
CX3CL1 GWAS (N=961)
$-\log_{10}(p)$
Chromosome

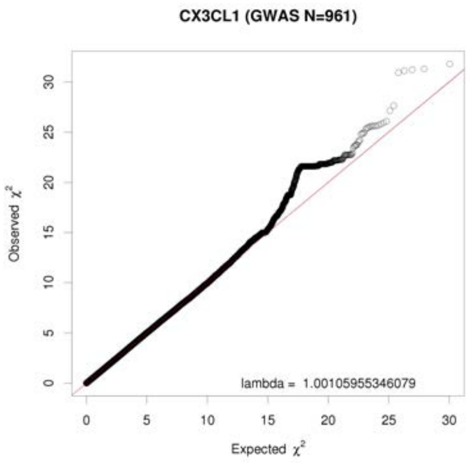
CX3CL1 (GWAS N=961)
Observed χ^2
Expected χ^2
lambda = 1.00105955346079

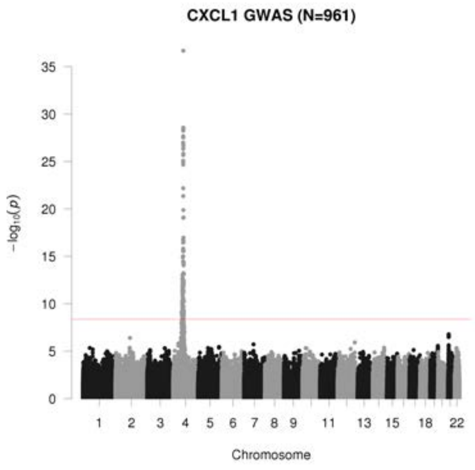
CXCL1 GWAS (N=961)
$-\log_{10}(p)$
Chromosome

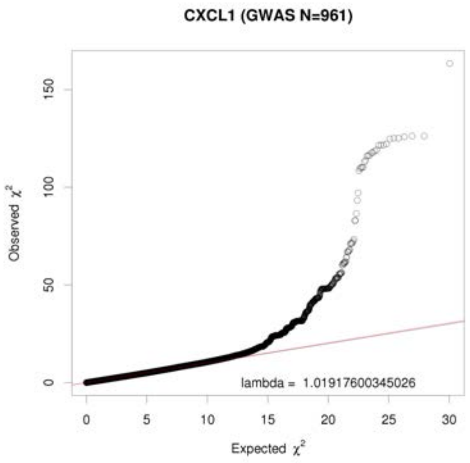
CXCL1 (GWAS N=961)
Observed χ^2
Expected χ^2
lambda = 1.01917600345026

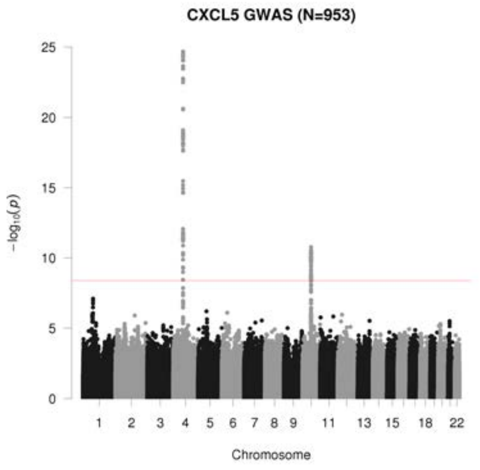
CXCL5 GWAS (N=953)
$-\log_{10}(p)$
Chromosome

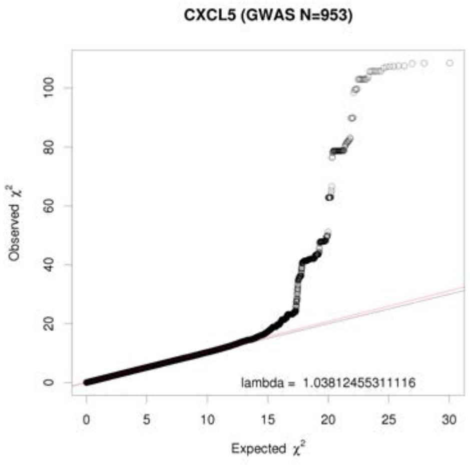
CXCL5 (GWAS N=953)
Observed χ^2
Expected χ^2
lambda = 1.03812455311116

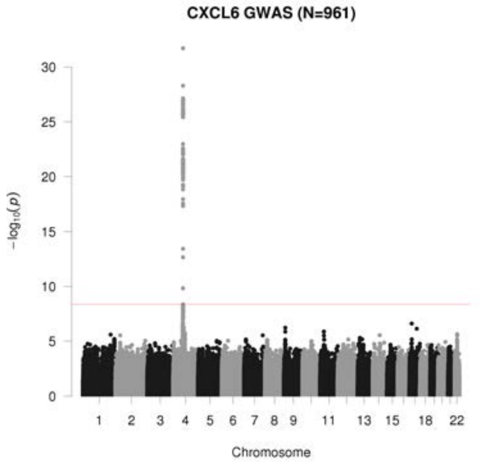
CXCL6 GWAS (N=961)
$-\log_{10}(p)$
Chromosome

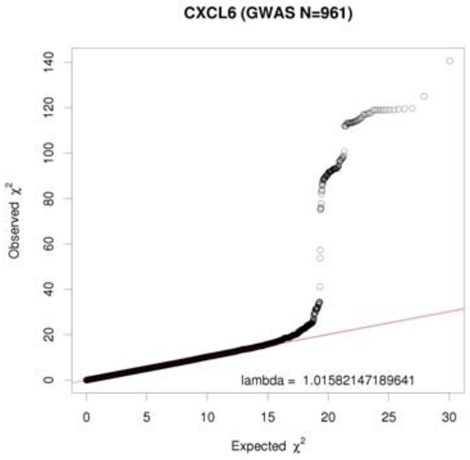
CXCL6 (GWAS N=961)
Observed χ^2
Expected χ^2
lambda = 1.01582147189641

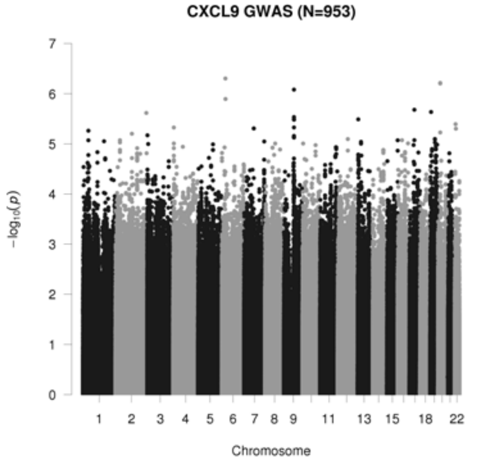
CXCL9 GWAS (N=953)
$-\log_{10}(p)$
Chromosome

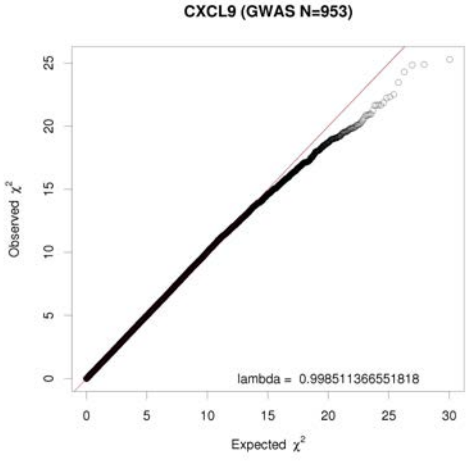
CXCL9 (GWAS N=953)
Observed χ^2
Expected χ^2
lambda = 0.998511366551818

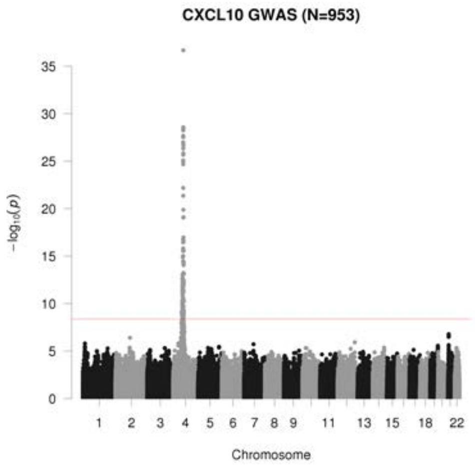
CXCL10 GWAS (N=953)
$-\log_{10}(p)$
Chromosome
1 2 3 4 5 6 7 8 9 11 13 15 18 22

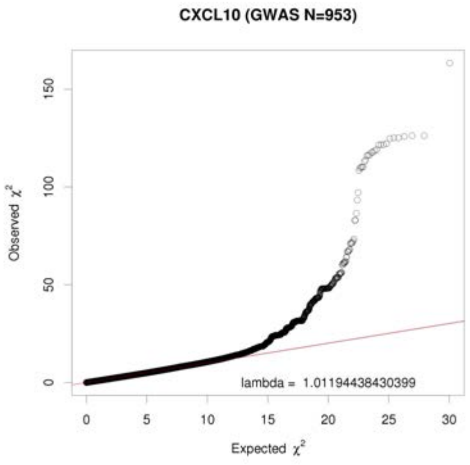
CXCL10 (GWAS N=953)
Observed χ^2
Expected χ^2
lambda = 1.01194438430399

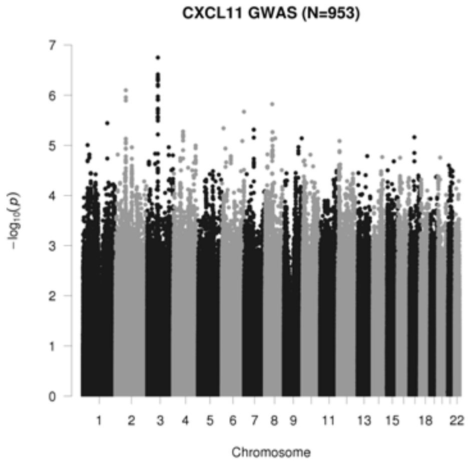
CXCL11 GWAS (N=953)
$-\log_{10}(p)$
Chromosome
1 2 3 4 5 6 7 8 9 11 13 15 18 22

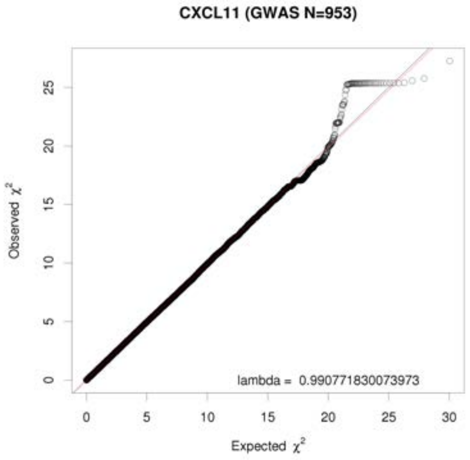
CXCL11 (GWAS N=953)
Observed χ^2
Expected χ^2
lambda = 0.990771830073973

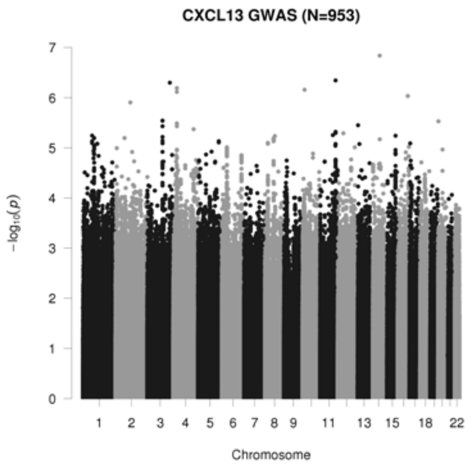
CXCL13 GWAS (N=953)
$-\log_{10}(p)$
Chromosome
1 2 3 4 5 6 7 8 9 11 13 15 18 22

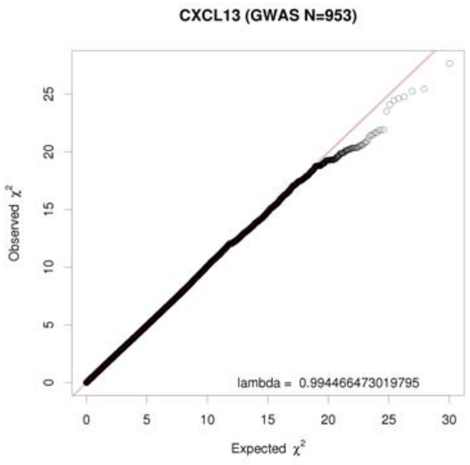
CXCL13 (GWAS N=953)
Observed χ^2
Expected χ^2
lambda = 0.994466473019795

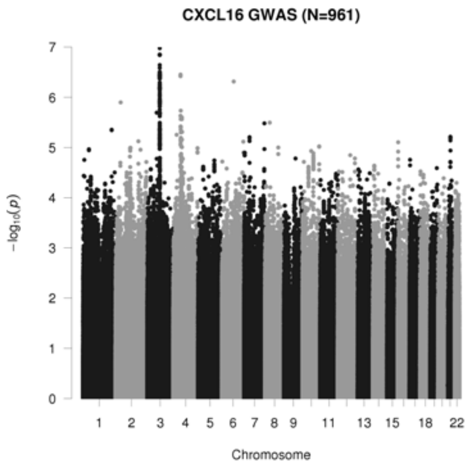
CXCL16 GWAS (N=961)
$-\log_{10}(p)$
Chromosome

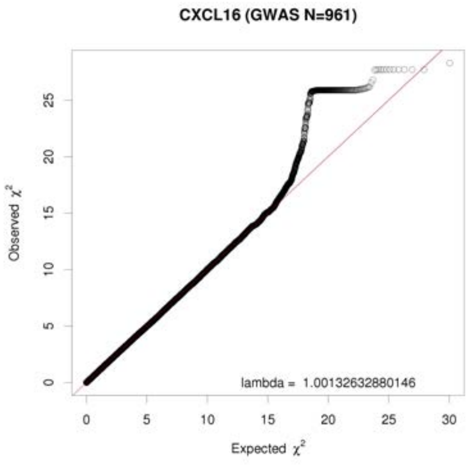
CXCL16 (GWAS N=961)
Observed χ^2
Expected χ^2
lambda = 1.00132632880146

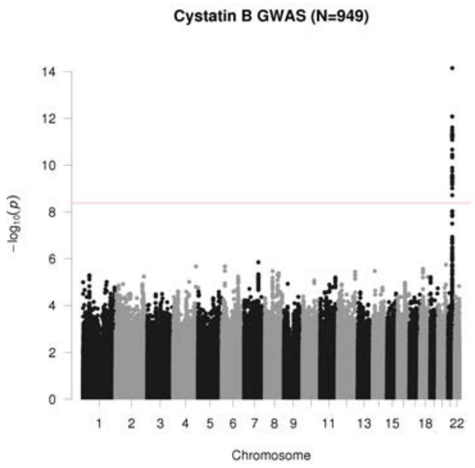
Cystatin B GWAS (N=949)
$-\log_{10}(p)$
Chromosome

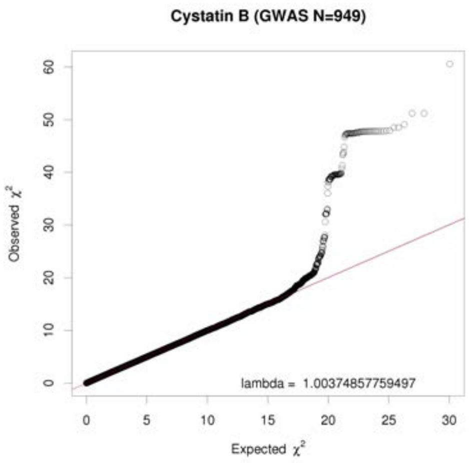
Cystatin B (GWAS N=949)
Observed χ^2
Expected χ^2
lambda = 1.00374857759497

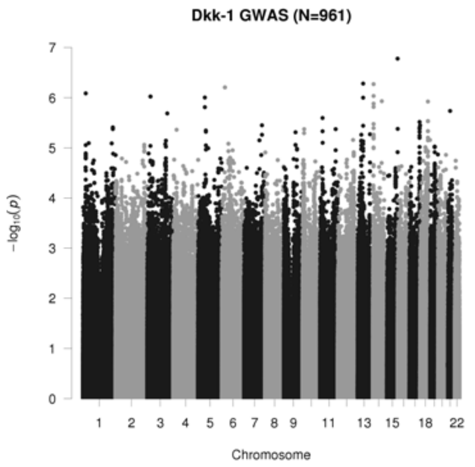
Dkk-1 GWAS (N=961)
$-\log_{10}(p)$
Chromosome

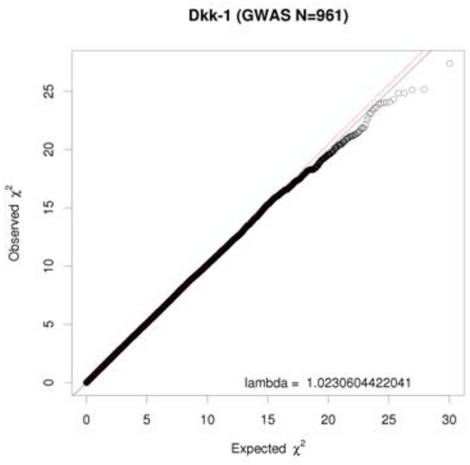
Dkk-1 (GWAS N=961)
Observed χ^2
Expected χ^2
lambda = 1.0230604422041

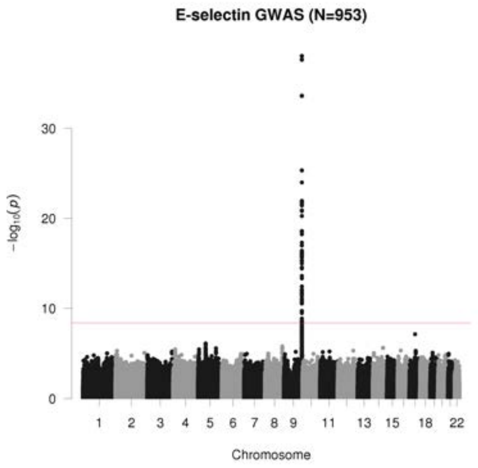
E-selectin GWAS (N=953)
$-\log_{10}(p)$
Chromosome

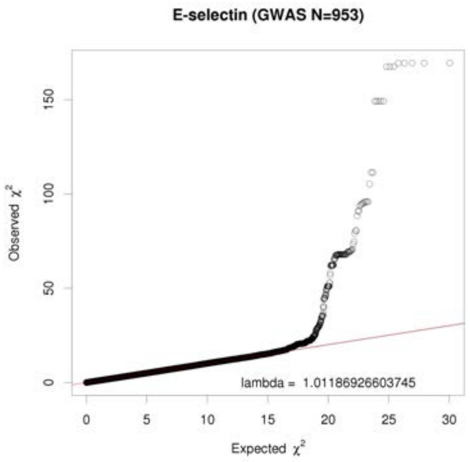
E-selectin (GWAS N=953)
Observed χ^2
Expected χ^2
lambda = 1.01186926603745

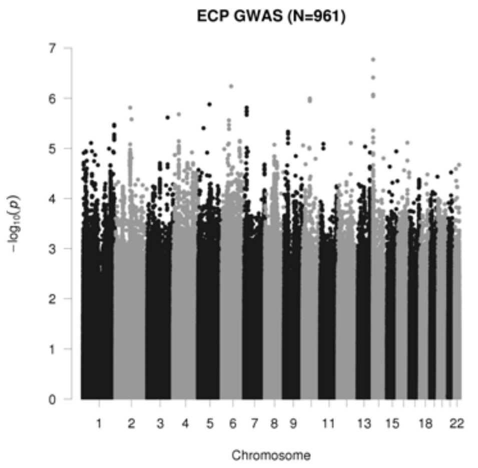
ECP GWAS (N=961)
$-\log_{10}(p)$
Chromosome

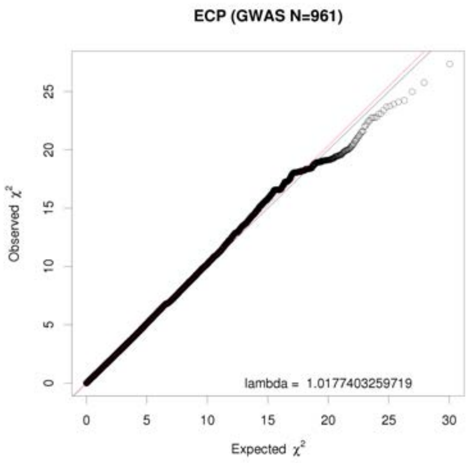
ECP (GWAS N=961)
Observed χ^2
Expected χ^2
lambda = 1.0177403259719

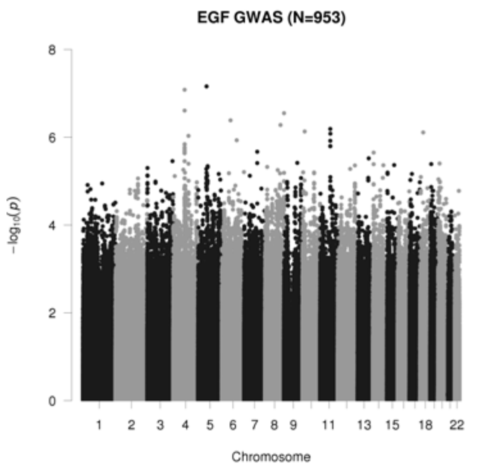
EGF GWAS (N=953)
$-\log_{10}(p)$
Chromosome

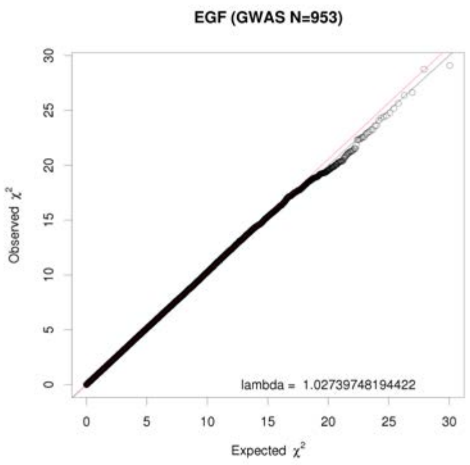
EGF (GWAS N=953)
Observed χ^2
Expected χ^2
lambda = 1.02739748194422

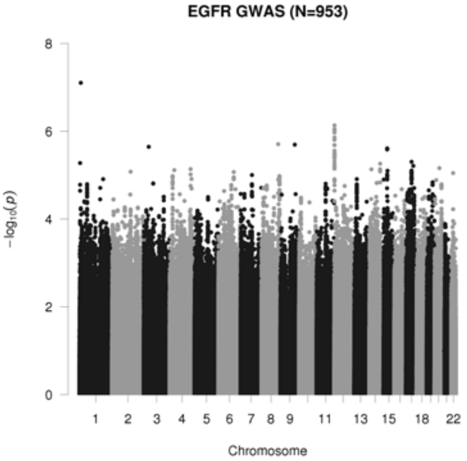
EGFR GWAS (N=953)
−log10(p)
Chromosome

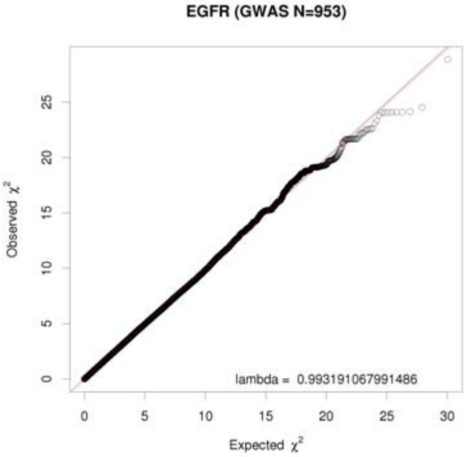
EGFR (GWAS N=953)
Observed χ2
Expected χ2
lambda = 0.993191067991486

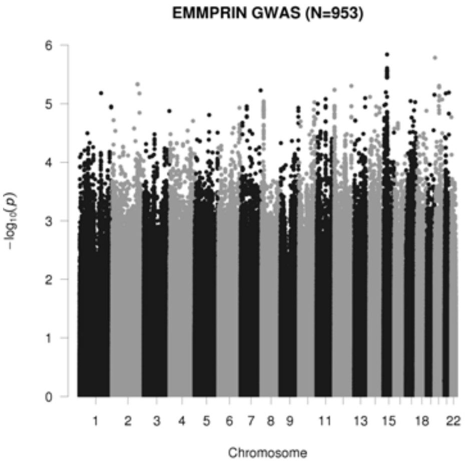
EMMPRIN GWAS (N=953)
−log10(p)
Chromosome

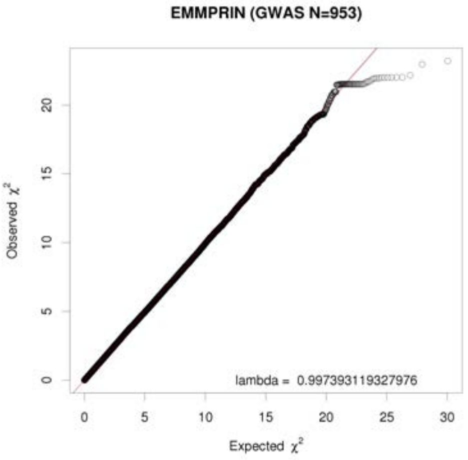
EMMPRIN (GWAS N=953)
Observed χ2
Expected χ2
lambda = 0.997393119327976

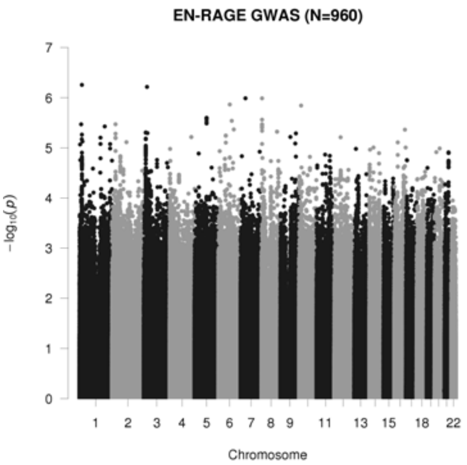
EN-RAGE GWAS (N=960)
−log10(p)
Chromosome

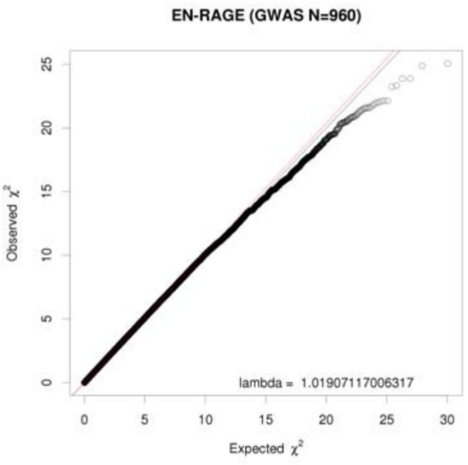
EN-RAGE (GWAS N=960)
Observed χ2
Expected χ2
lambda = 1.01907117006317

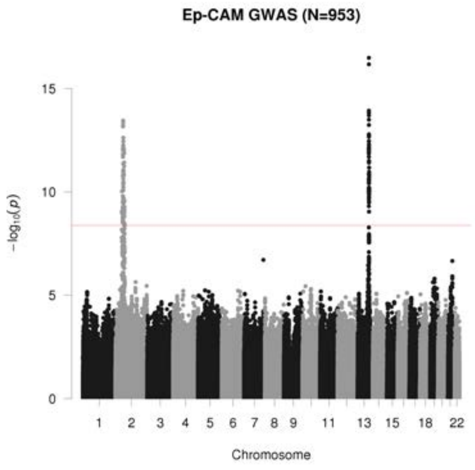
Ep-CAM GWAS (N=953)
$-\log_{10}(p)$
0
5
10
15
1 2 3 4 5 6 7 8 9 11 13 15 18 22
Chromosome

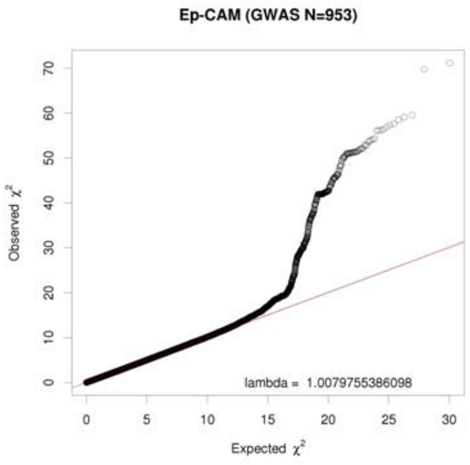
Ep-CAM (GWAS N=953)
Observed χ^2
0 10 20 30 40 50 60 70
lambda = 1.0079755386098
0 5 10 15 20 25 30
Expected χ^2

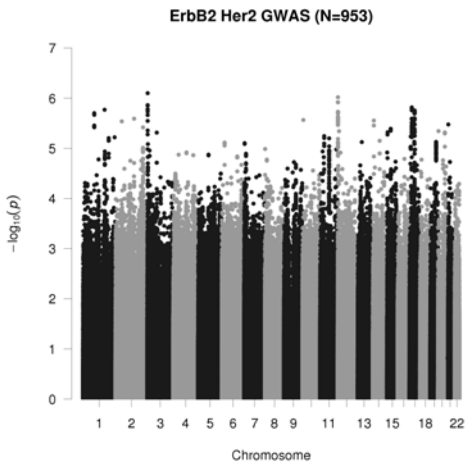
ErbB2 Her2 GWAS (N=953)
$-\log_{10}(p)$
0 1 2 3 4 5 6 7
1 2 3 4 5 6 7 8 9 11 13 15 18 22
Chromosome

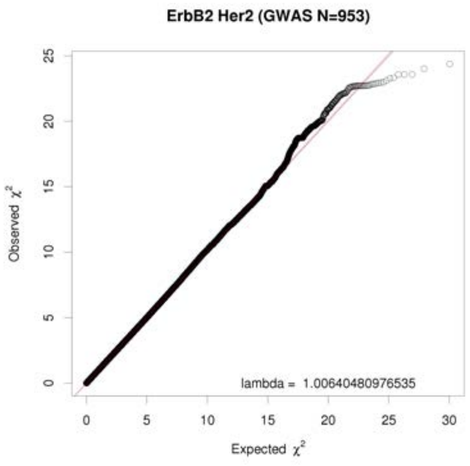
ErbB2 Her2 (GWAS N=953)
Observed χ^2
0 5 10 15 20 25
lambda = 1.00640480976535
0 5 10 15 20 25 30
Expected χ^2

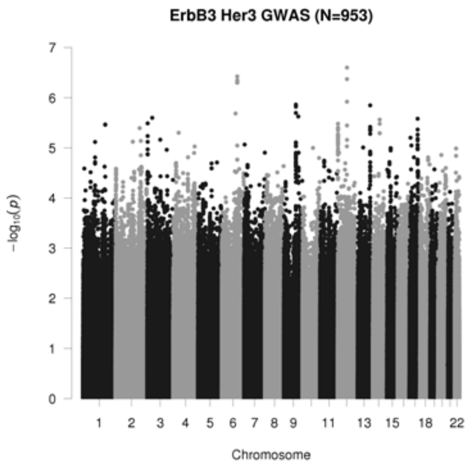
ErbB3 Her3 GWAS (N=953)
$-\log_{10}(p)$
0 1 2 3 4 5 6 7
1 2 3 4 5 6 7 8 9 11 13 15 18 22
Chromosome

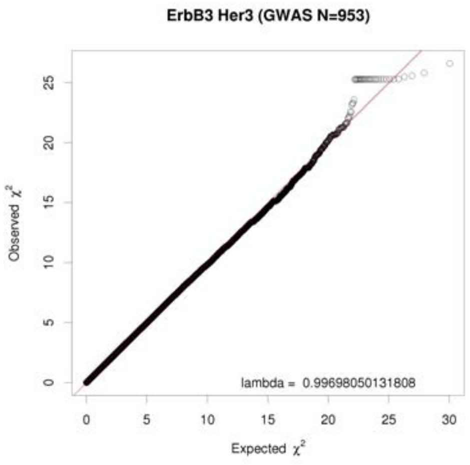
ErbB3 Her3 (GWAS N=953)
Observed χ^2
0 5 10 15 20 25
lambda = 0.99698050131808
0 5 10 15 20 25 30
Expected χ^2

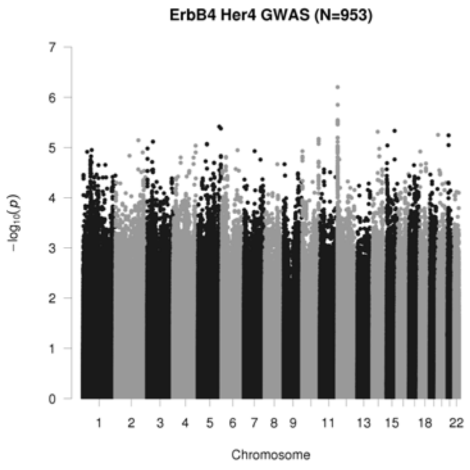
ErbB4 Her4 GWAS (N=953)
$-\log_{10}(p)$
Chromosome

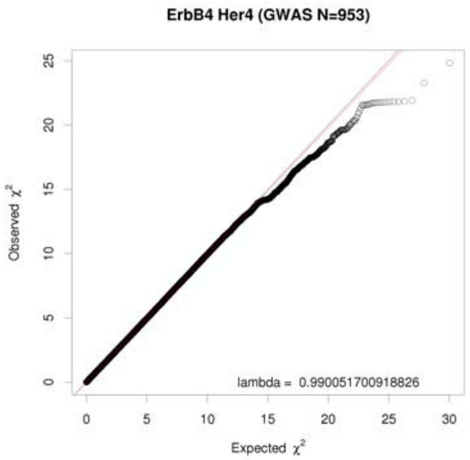
ErbB4 Her4 (GWAS N=953)
Observed χ^2
Expected χ^2
lambda = 0.990051700918826

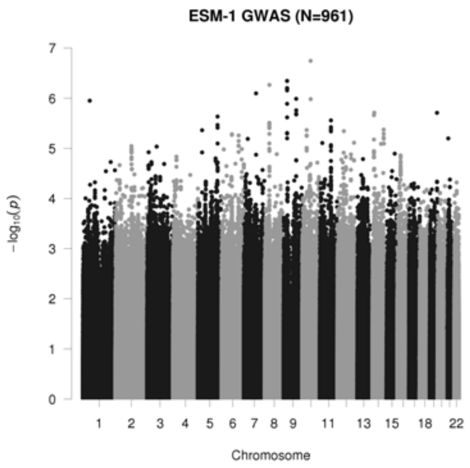
ESM-1 GWAS (N=961)
$-\log_{10}(p)$
Chromosome

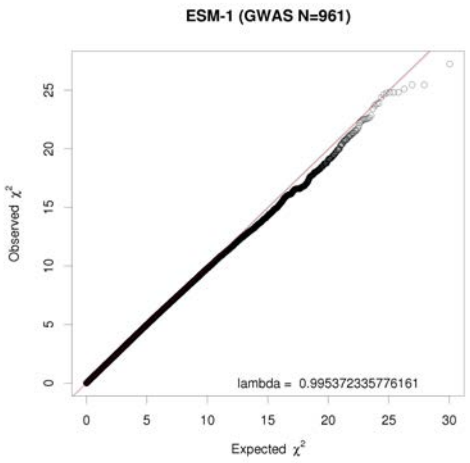
ESM-1 (GWAS N=961)
Observed χ^2
Expected χ^2
lambda = 0.995372335776161

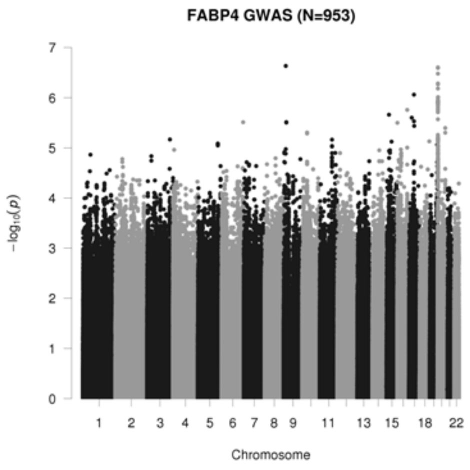
FABP4 GWAS (N=953)
$-\log_{10}(p)$
Chromosome

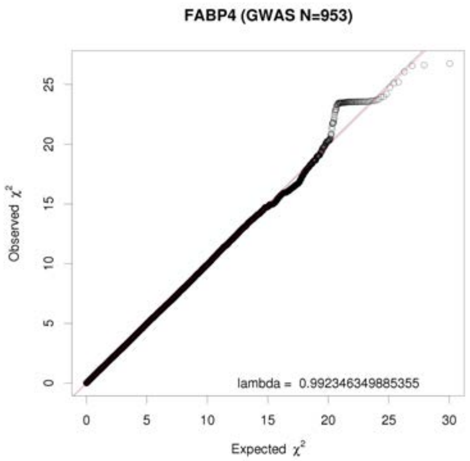
FABP4 (GWAS N=953)
Observed χ^2
Expected χ^2
lambda = 0.992346349885355

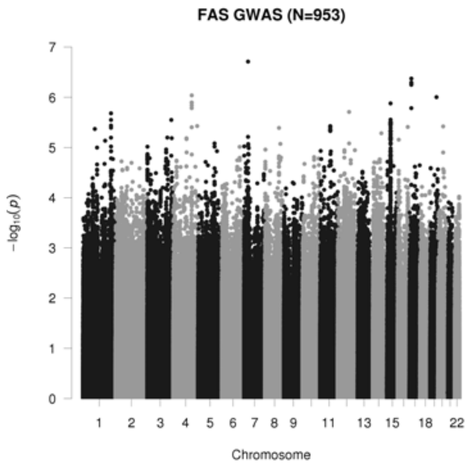
FAS GWAS (N=953)
$-\log_{10}(p)$
Chromosome

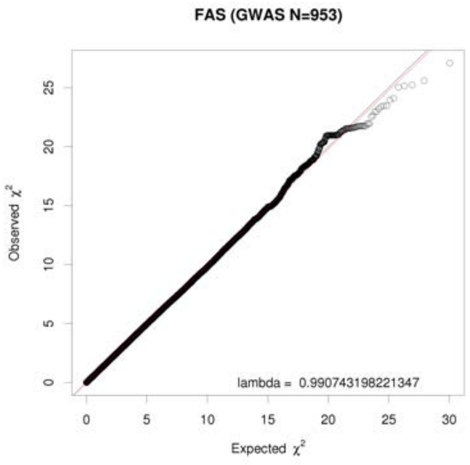
FAS (GWAS N=953)
Observed χ^2
Expected χ^2
lambda = 0.990743198221347

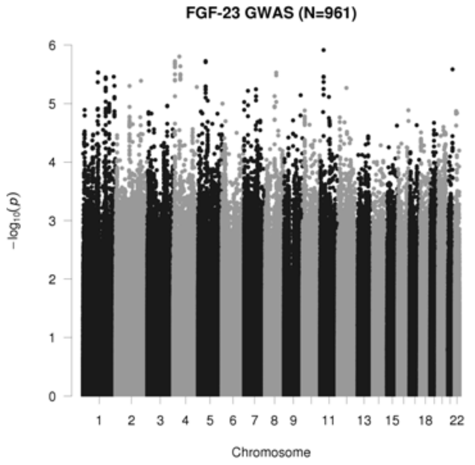
FGF-23 GWAS (N=961)
$-\log_{10}(p)$
Chromosome

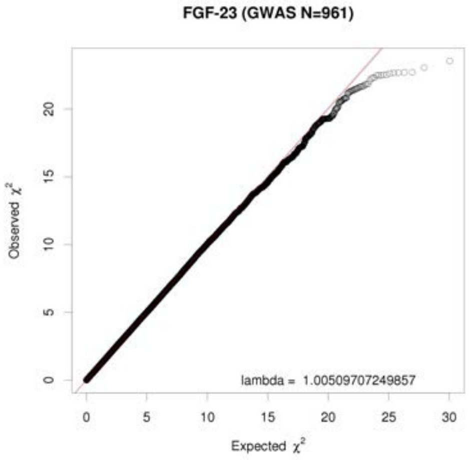
FGF-23 (GWAS N=961)
Observed χ^2
Expected χ^2
lambda = 1.00509707249857

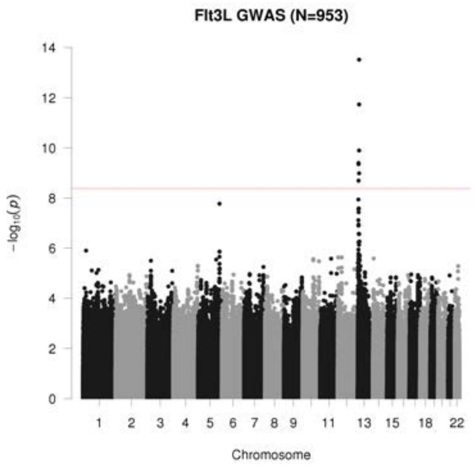
Flt3L GWAS (N=953)
$-\log_{10}(p)$
Chromosome

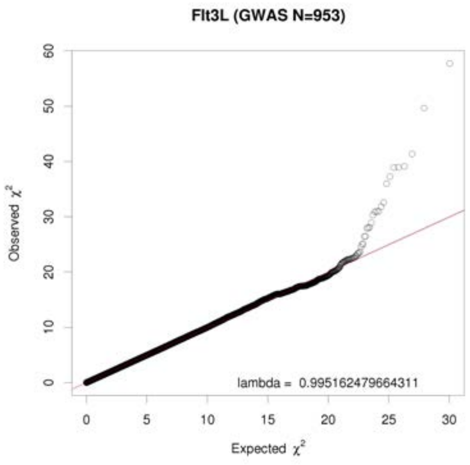
Flt3L (GWAS N=953)
Observed χ^2
Expected χ^2
lambda = 0.995162479664311

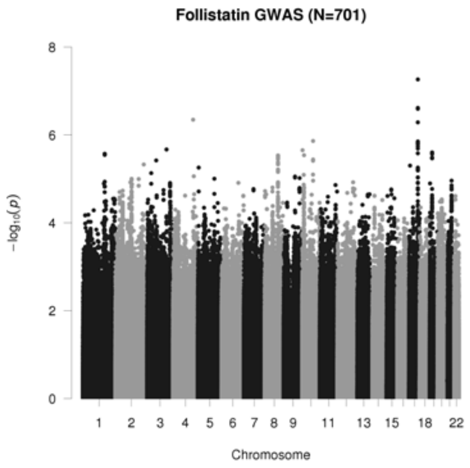
Follistatin GWAS (N=701)
$-\log_{10}(p)$
Chromosome

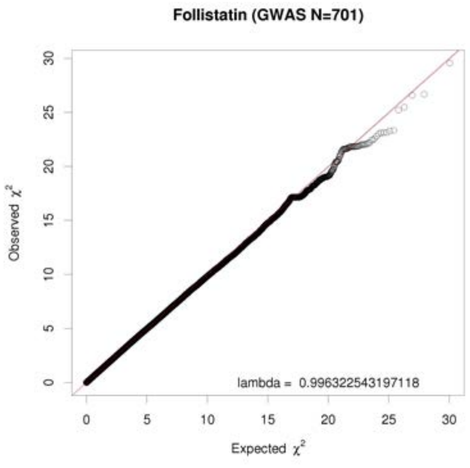
Follistatin (GWAS N=701)
Observed χ^2
Expected χ^2
lambda = 0.996322543197118

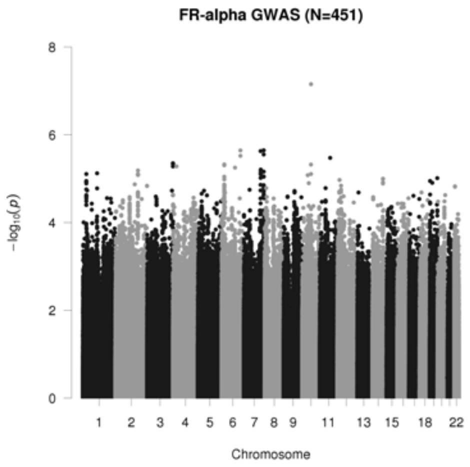
FR-alpha GWAS (N=451)
$-\log_{10}(p)$
Chromosome

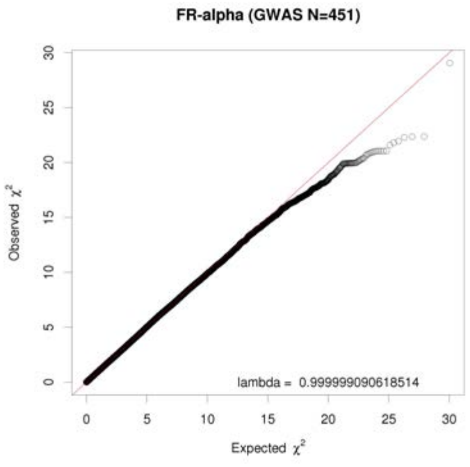
FR-alpha (GWAS N=451)
Observed χ^2
Expected χ^2
lambda = 0.999999090618514

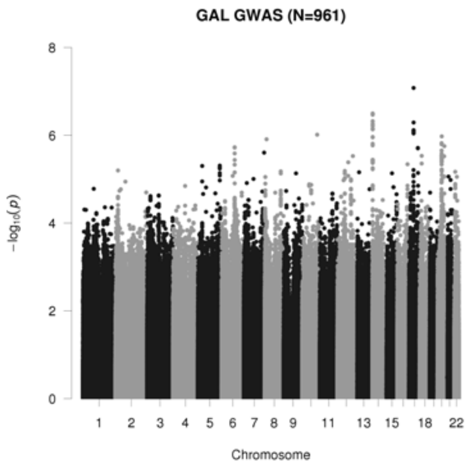
GAL GWAS (N=961)
$-\log_{10}(p)$
Chromosome

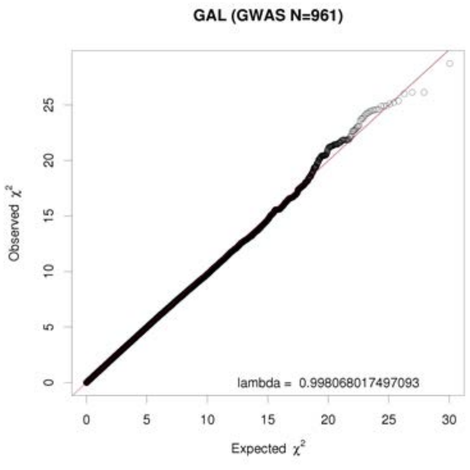
GAL (GWAS N=961)
Observed χ^2
Expected χ^2
lambda = 0.998068017497093

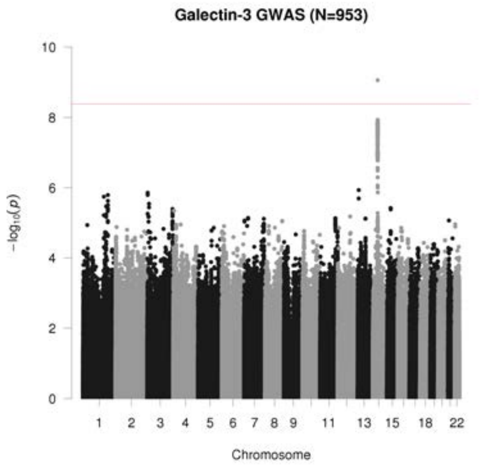
Galectin-3 GWAS (N=953)
$-\log_{10}(p)$
Chromosome

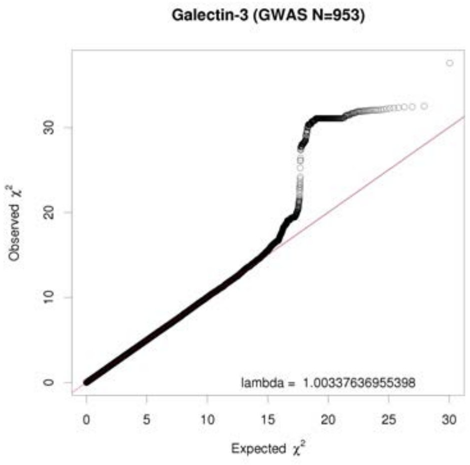
Galectin-3 (GWAS N=953)
Observed χ^2
Expected χ^2
lambda = 1.00337636955398

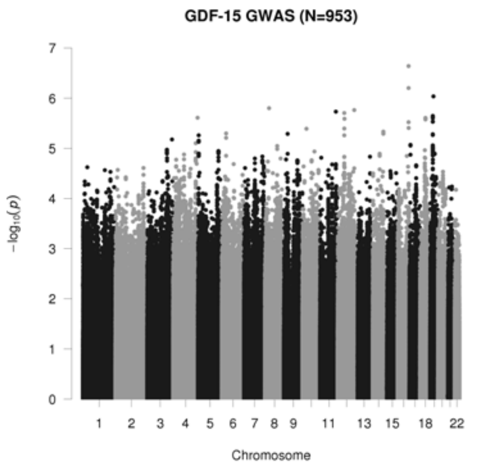
GDF-15 GWAS (N=953)
$-\log_{10}(p)$
Chromosome

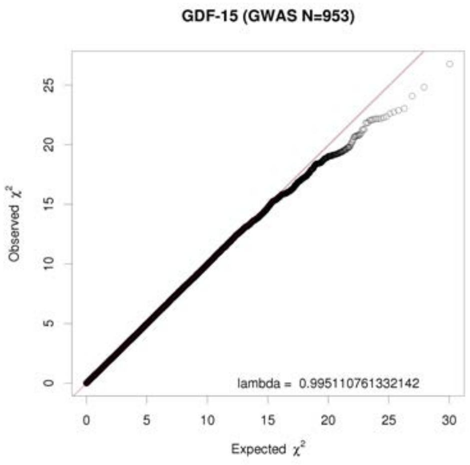
GDF-15 (GWAS N=953)
Observed χ^2
Expected χ^2
lambda = 0.995110761332142

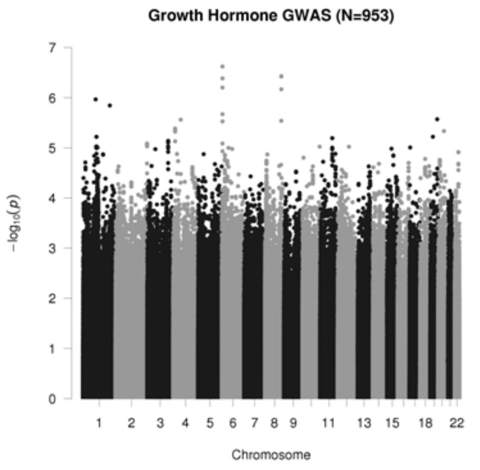
Growth Hormone GWAS (N=953)
$-\log_{10}(p)$
Chromosome

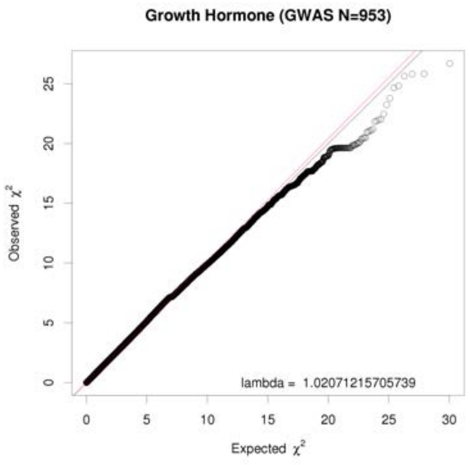
Growth Hormone (GWAS N=953)
Observed χ^2
Expected χ^2
lambda = 1.02071215705739

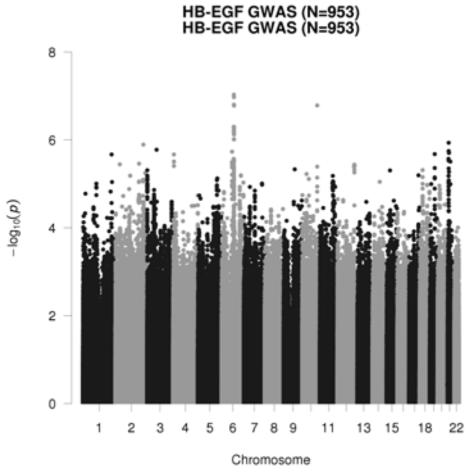
HB-EGF GWAS (N=953)
HB-EGF GWAS (N=953)
$-\log_{10}(p)$
Chromosome

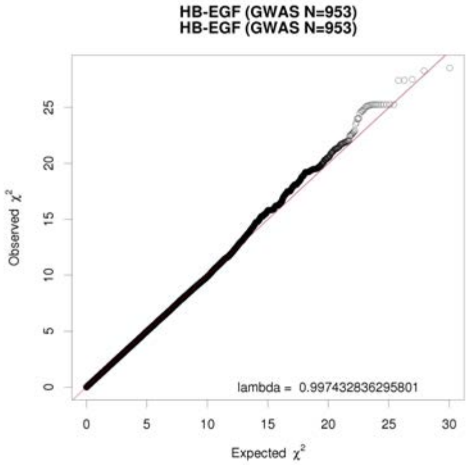
HB-EGF (GWAS N=953)
HB-EGF (GWAS N=953)
Observed χ^2
Expected χ^2
lambda = 0.997432836295801

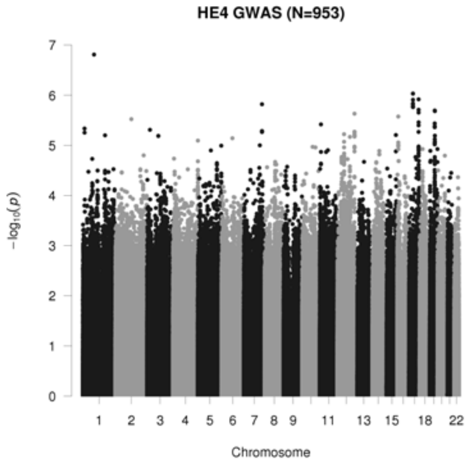
HE4 GWAS (N=953)
$-\log_{10}(p)$
Chromosome

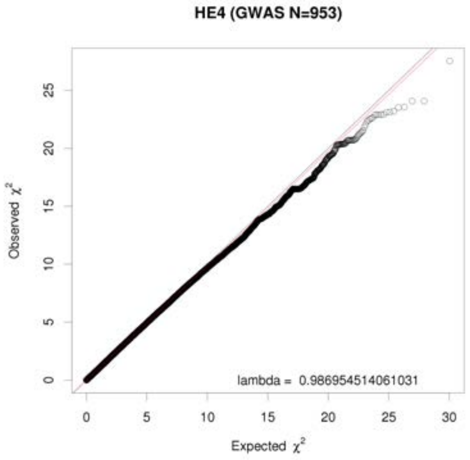
HE4 (GWAS N=953)
Observed χ^2
Expected χ^2
lambda = 0.986954514061031

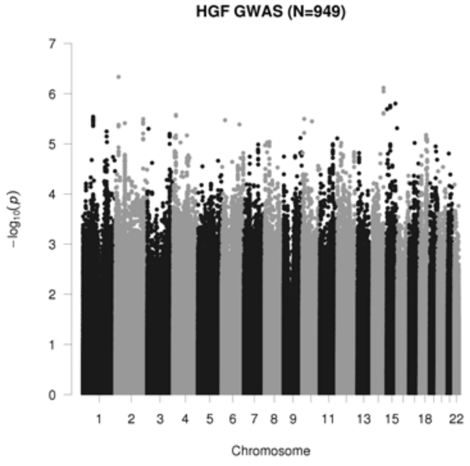
HGF GWAS (N=949)
$-\log_{10}(p)$
Chromosome

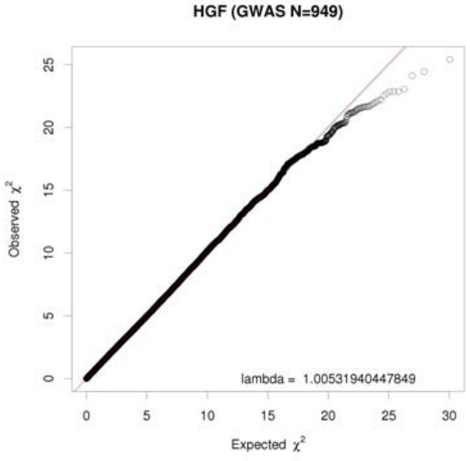
HGF (GWAS N=949)
Observed χ^2
Expected χ^2
lambda = 1.00531940447849

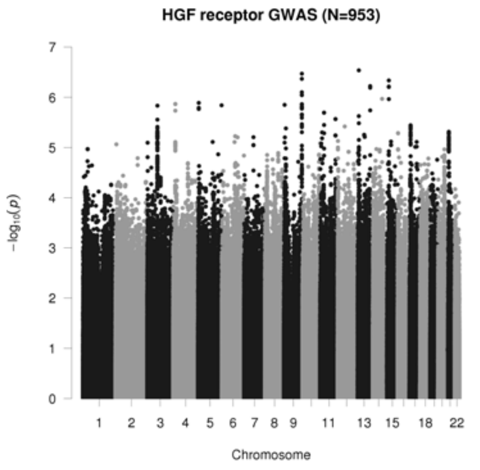
HGF receptor GWAS (N=953)
$-\log_{10}(p)$
Chromosome

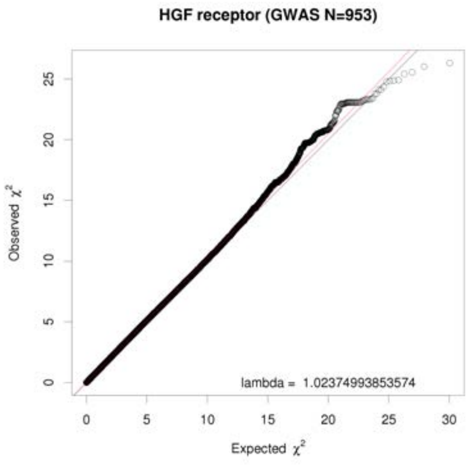
HGF receptor (GWAS N=953)
Observed χ^2
Expected χ^2
lambda = 1.02374993853574

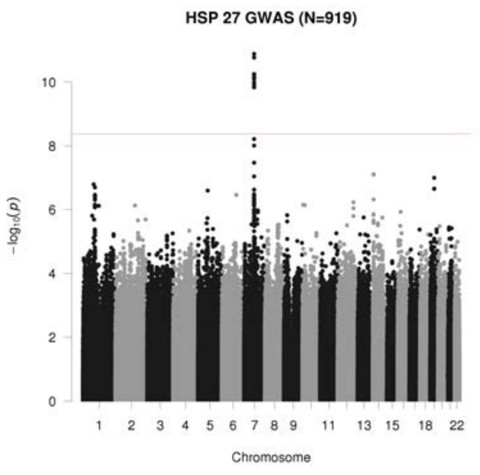
HSP 27 GWAS (N=919)
$-\log_{10}(p)$
Chromosome

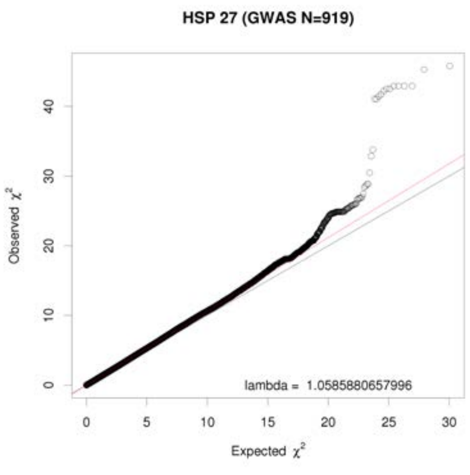
HSP 27 (GWAS N=919)
Observed χ^2
Expected χ^2
lambda = 1.0585880657996

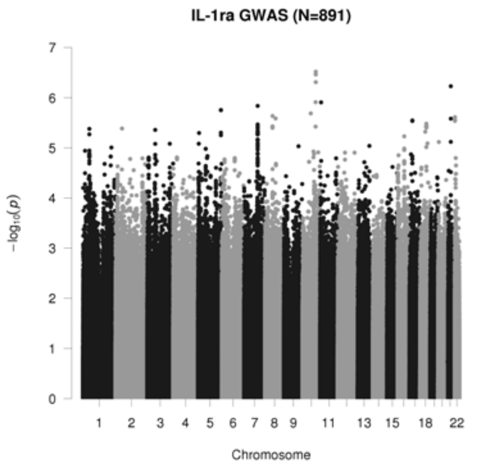
IL-1ra GWAS (N=891)
$-\log_{10}(p)$
Chromosome

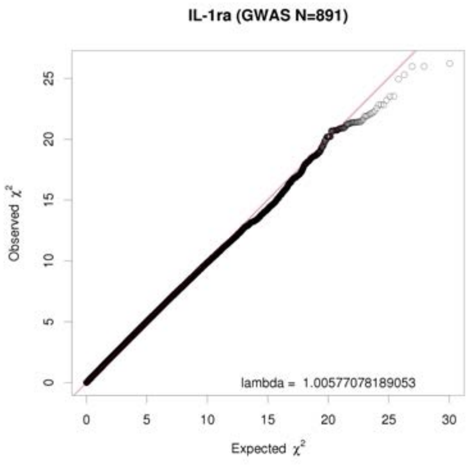
IL-1ra (GWAS N=891)
Observed χ^2
Expected χ^2
lambda = 1.00577078189053

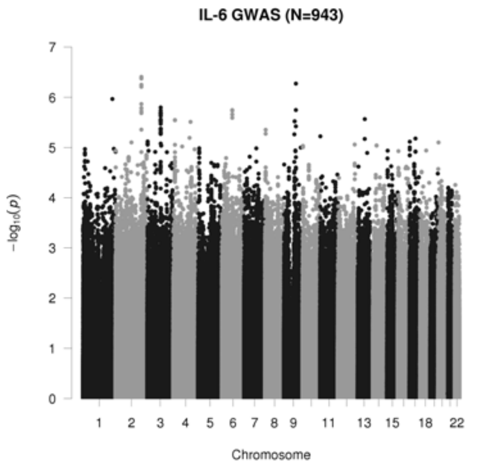
IL-6 GWAS (N=943)
$-\log_{10}(p)$
Chromosome

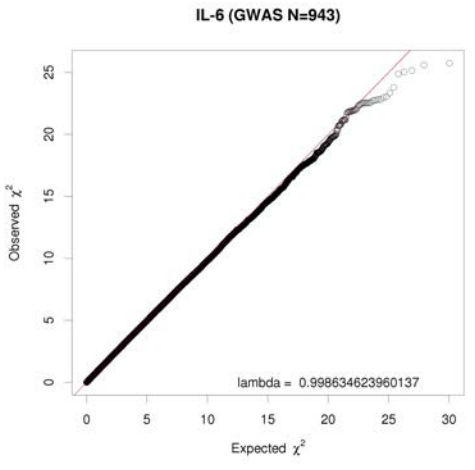
IL-6 (GWAS N=943)
Observed χ^2
Expected χ^2
lambda = 0.998634623960137

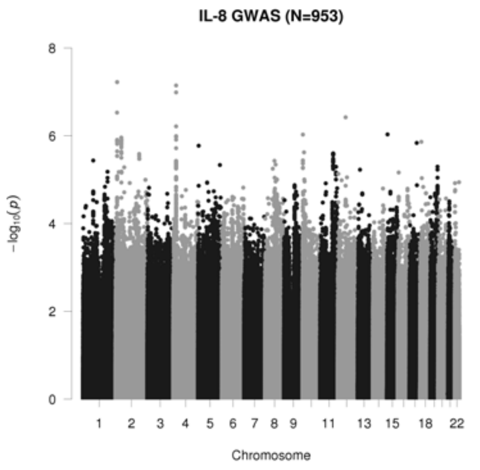
IL-8 GWAS (N=953)
$-\log_{10}(p)$
Chromosome

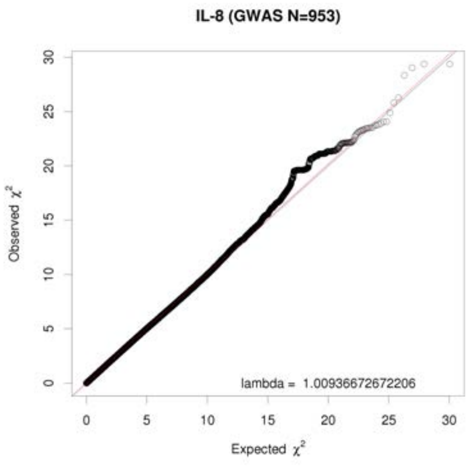
IL-8 (GWAS N=953)
Observed χ^2
Expected χ^2
lambda = 1.00936672672206

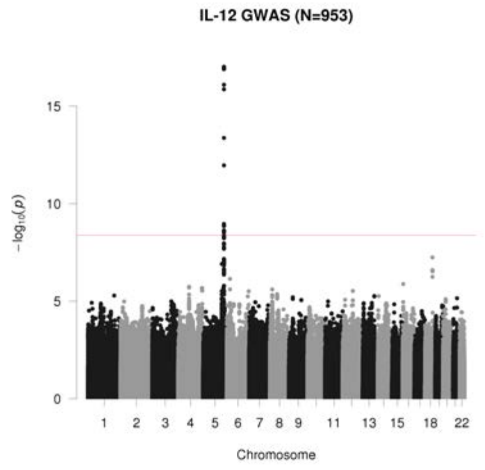
IL-12 GWAS (N=953)
$-\log_{10}(p)$
Chromosome

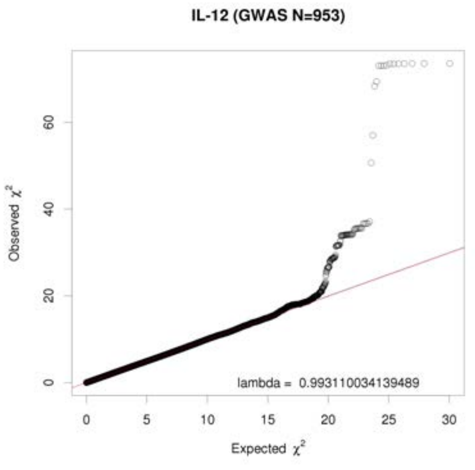
IL-12 (GWAS N=953)
Observed χ^2
Expected χ^2
lambda = 0.993110034139489

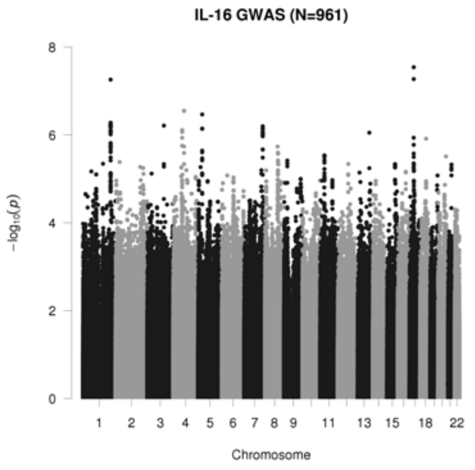
IL-16 GWAS (N=961)
$-\log_{10}(p)$
Chromosome

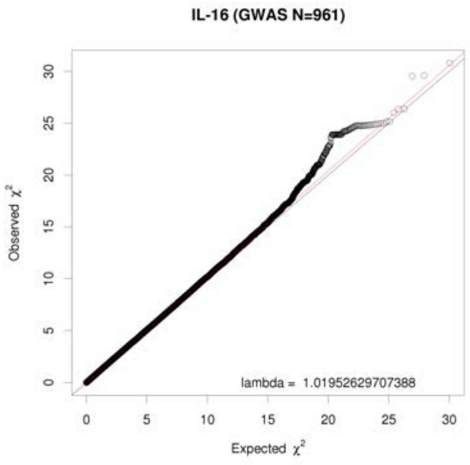
IL-16 (GWAS N=961)
Observed χ^2
Expected χ^2
lambda = 1.01952629707388

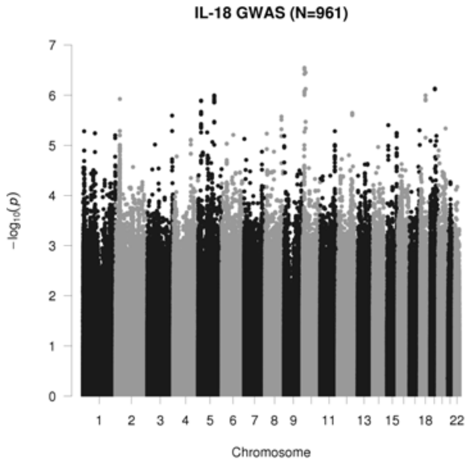
IL-18 GWAS (N=961)
$-\log_{10}(p)$
Chromosome

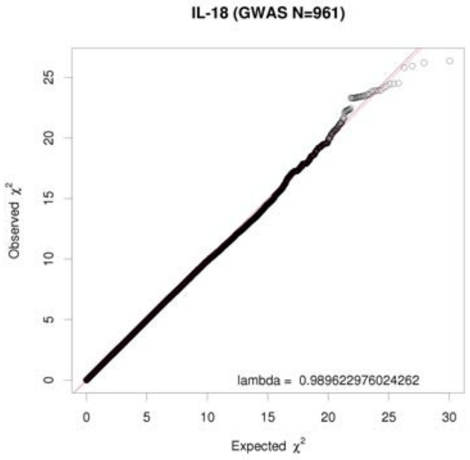
IL-18 (GWAS N=961)
Observed χ^2
Expected χ^2
lambda = 0.989622976024262

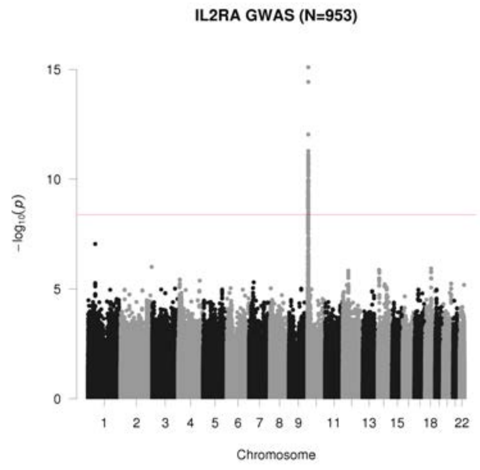
IL2RA GWAS (N=953)
$-\log_{10}(p)$
Chromosome

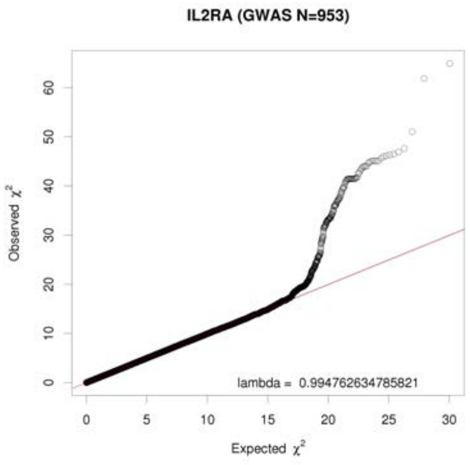
IL2RA (GWAS N=953)
Observed χ^2
Expected χ^2
lambda = 0.994762634785821

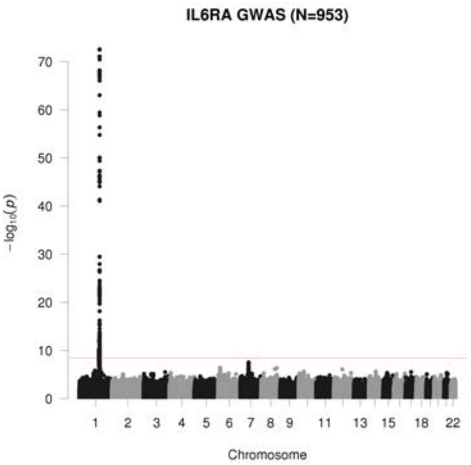
IL6RA GWAS (N=953)
$-\log_{10}(p)$
Chromosome

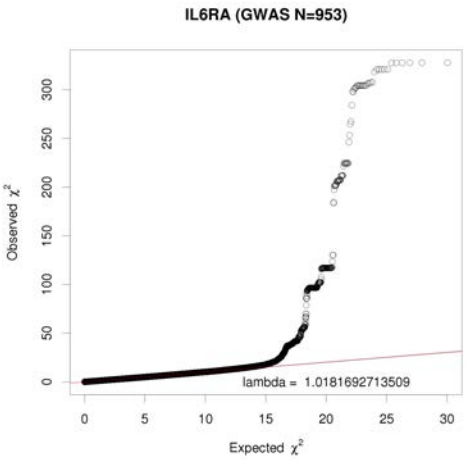
IL6RA (GWAS N=953)
Observed χ^2
Expected χ^2
lambda = 1.0181692713509

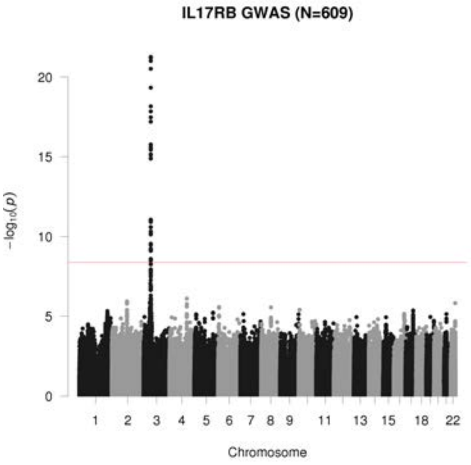
IL17RB GWAS (N=609)
$-\log_{10}(p)$
Chromosome

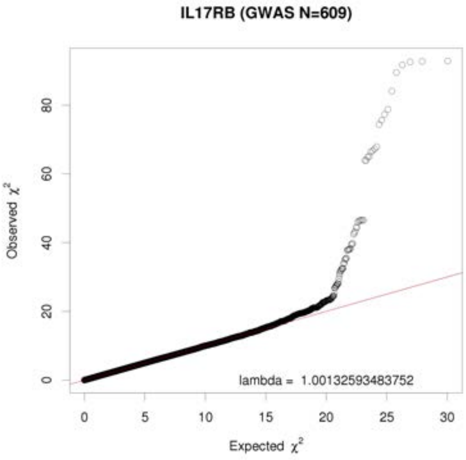
IL17RB (GWAS N=609)
Observed χ^2
Expected χ^2
lambda = 1.00132593483752

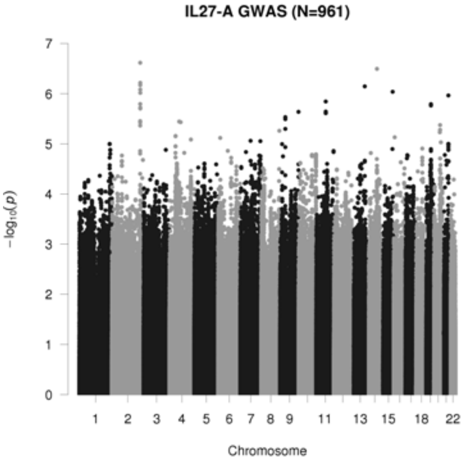
IL27-A GWAS (N=961)
$-\log_{10}(p)$
Chromosome

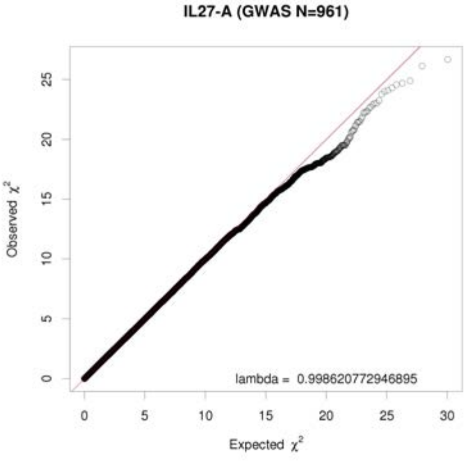
IL27-A (GWAS N=961)
Observed χ^2
Expected χ^2
lambda = 0.998620772946895

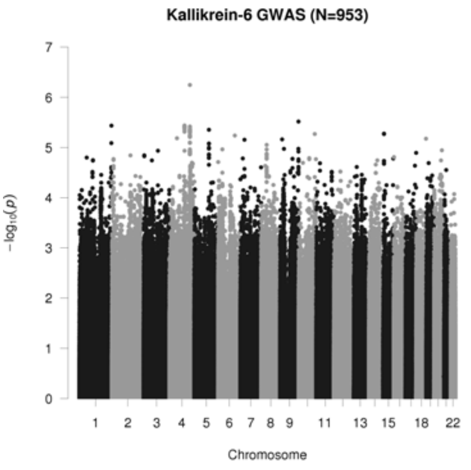
Kallikrein-6 GWAS (N=953)
$-\log_{10}(p)$
Chromosome
1 2 3 4 5 6 7 8 9 11 13 15 18 22

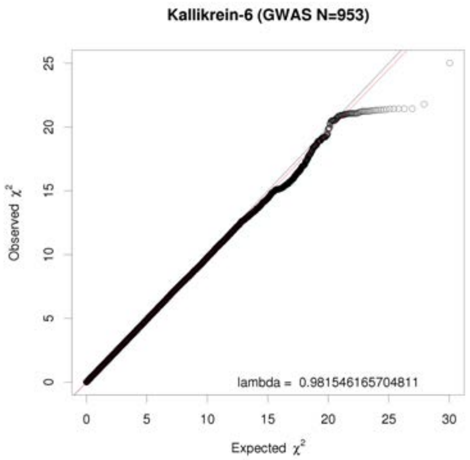
Kallikrein-6 (GWAS N=953)
Observed χ^2
Expected χ^2
lambda = 0.981546165704811

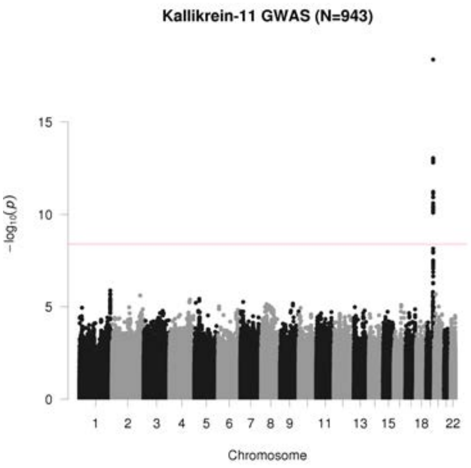
Kallikrein-11 GWAS (N=943)
$-\log_{10}(p)$
Chromosome
1 2 3 4 5 6 7 8 9 11 13 15 18 22

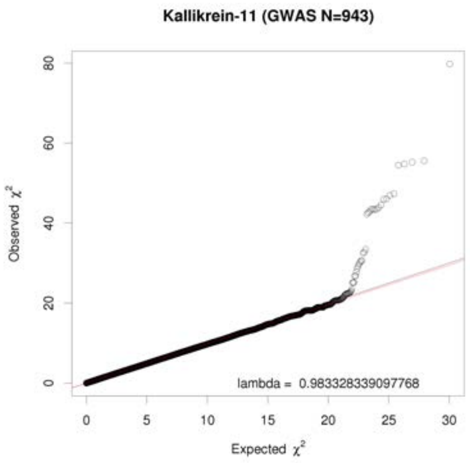
Kallikrein-11 (GWAS N=943)
Observed χ^2
Expected χ^2
lambda = 0.983328339097768

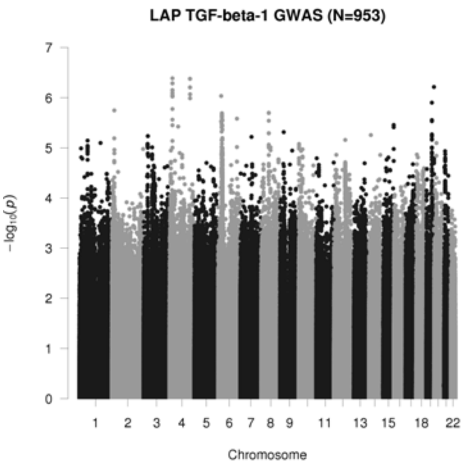
LAP TGF-beta-1 GWAS (N=953)
$-\log_{10}(p)$
Chromosome
1 2 3 4 5 6 7 8 9 11 13 15 18 22

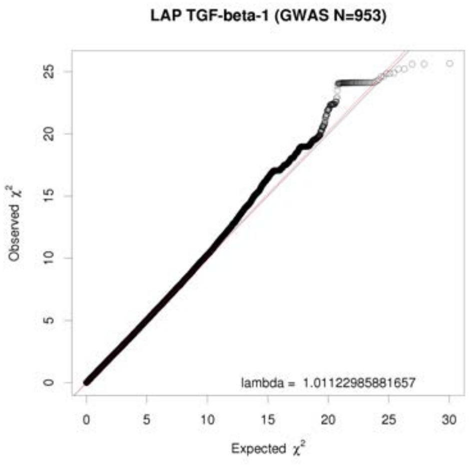
LAP TGF-beta-1 (GWAS N=953)
Observed χ^2
Expected χ^2
lambda = 1.01122985881657

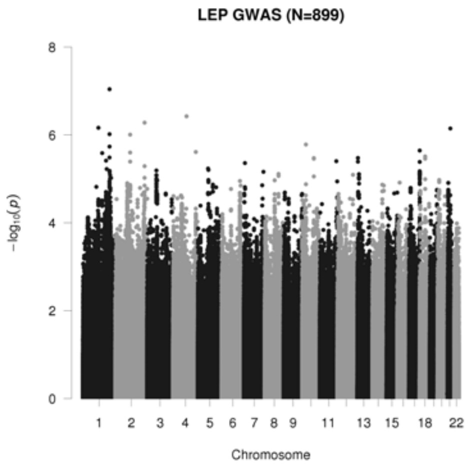
LEP GWAS (N=899)
−log₁₀(p)
Chromosome
1 2 3 4 5 6 7 8 9 11 13 15 18 22

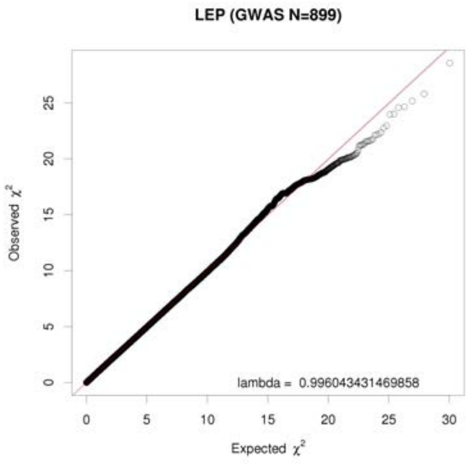
LEP (GWAS N=899)
Observed χ²
Expected χ²
lambda = 0.996043431469858

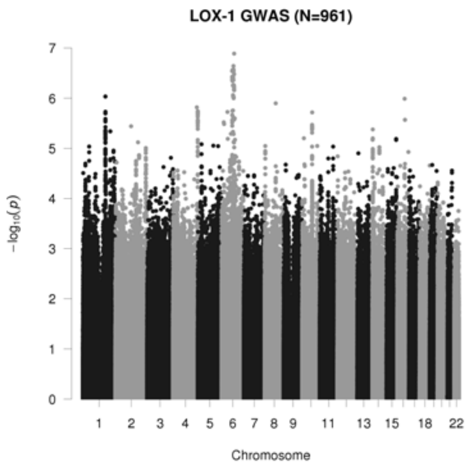
LOX-1 GWAS (N=961)
−log₁₀(p)
Chromosome
1 2 3 4 5 6 7 8 9 11 13 15 18 22

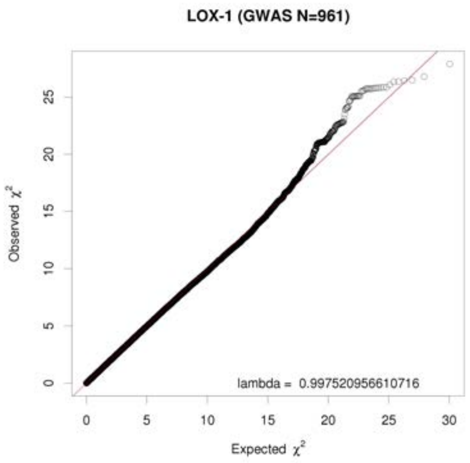
LOX-1 (GWAS N=961)
Observed χ²
Expected χ²
lambda = 0.997520956610716

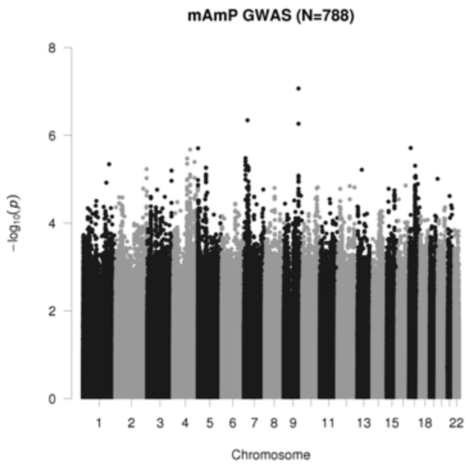
mAmP GWAS (N=788)
−log₁₀(p)
Chromosome
1 2 3 4 5 6 7 8 9 11 13 15 18 22

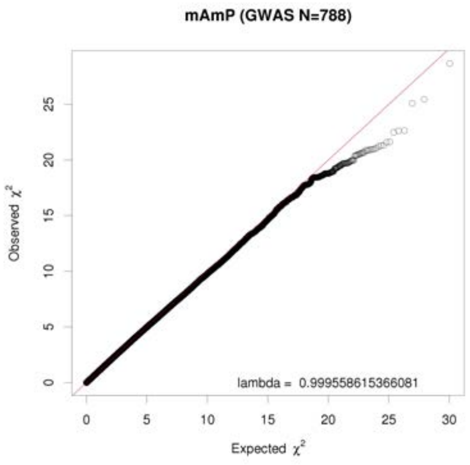
mAmP (GWAS N=788)
Observed χ²
Expected χ²
lambda = 0.999558615366081

MB GWAS (N=959)

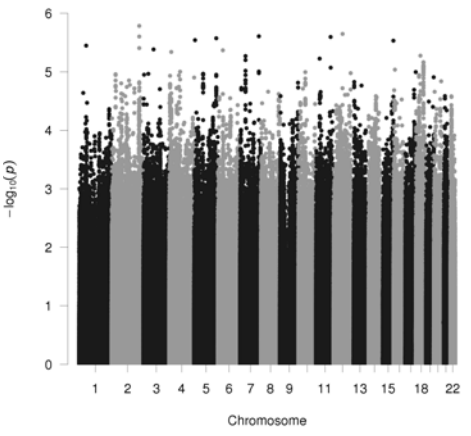

MB (GWAS N=959)

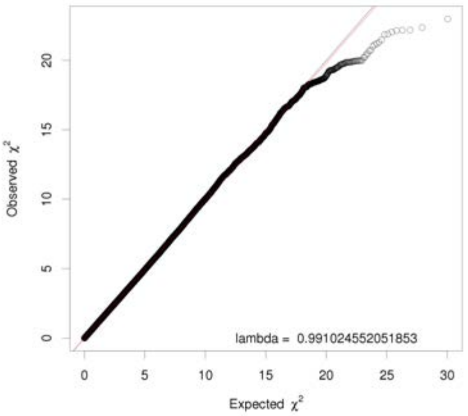

MCP-1 GWAS (N=953)

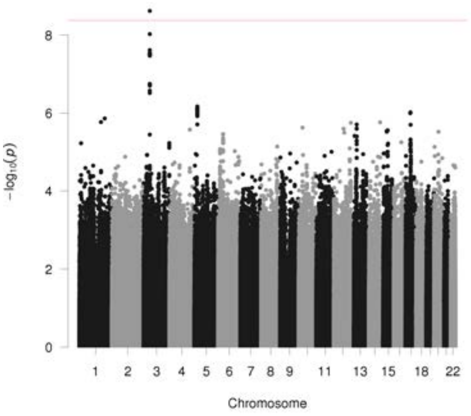

MCP-1 (GWAS N=953)

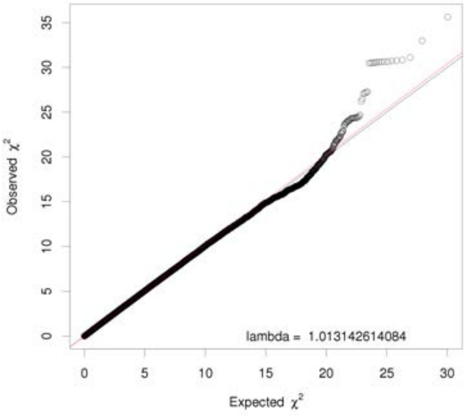

MIA GWAS (N=949)

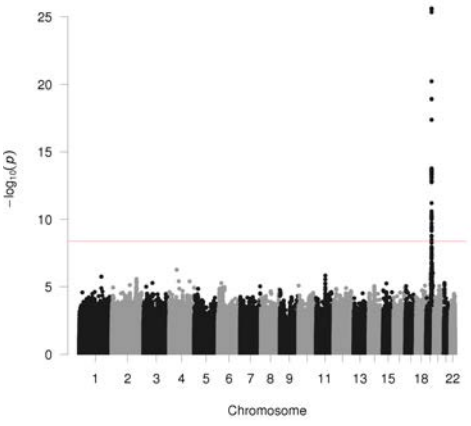

MIA (GWAS N=949)

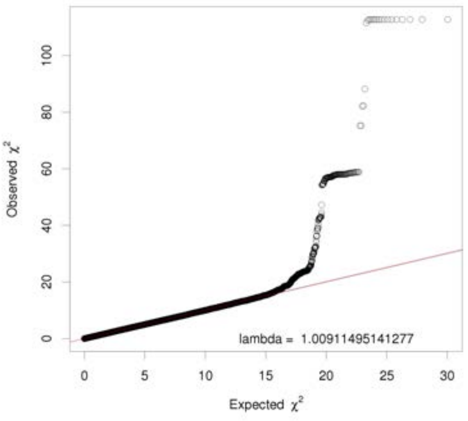

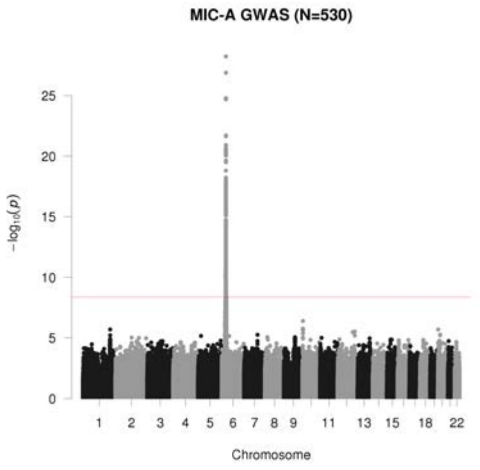
MIC-A GWAS (N=530)
$-\log_{10}(p)$
Chromosome

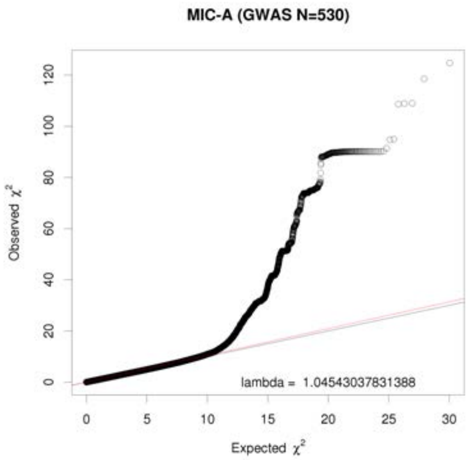
MIC-A (GWAS N=530)
Observed χ^2
Expected χ^2
lambda = 1.04543037831388

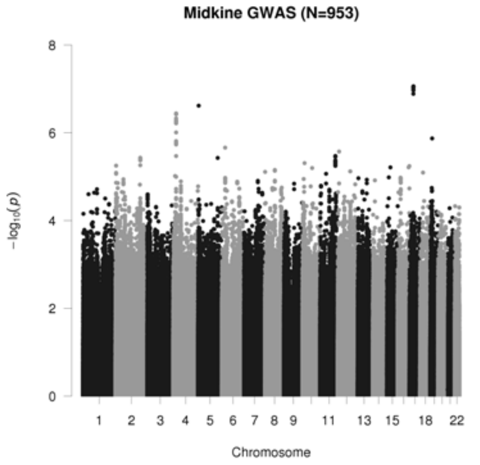
Midkine GWAS (N=953)
$-\log_{10}(p)$
Chromosome

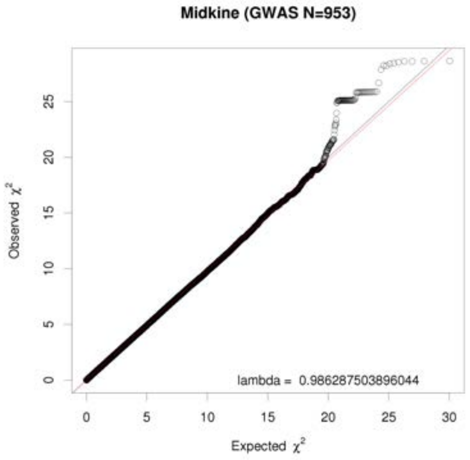
Midkine (GWAS N=953)
Observed χ^2
Expected χ^2
lambda = 0.986287503896044

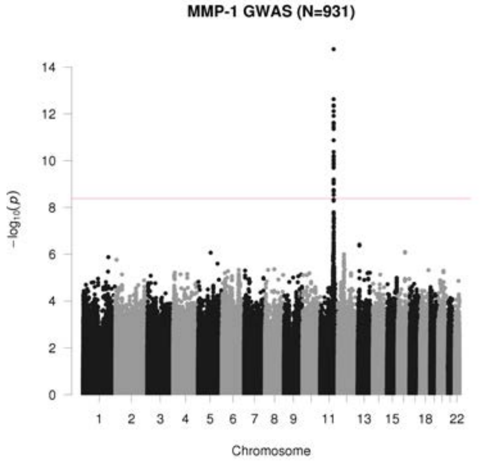
MMP-1 GWAS (N=931)
$-\log_{10}(p)$
Chromosome

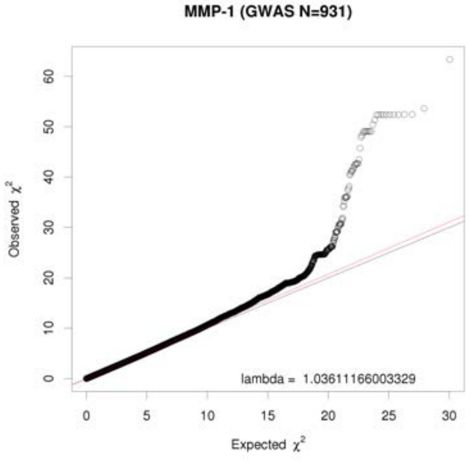
MMP-1 (GWAS N=931)
Observed χ^2
Expected χ^2
lambda = 1.03611166003329

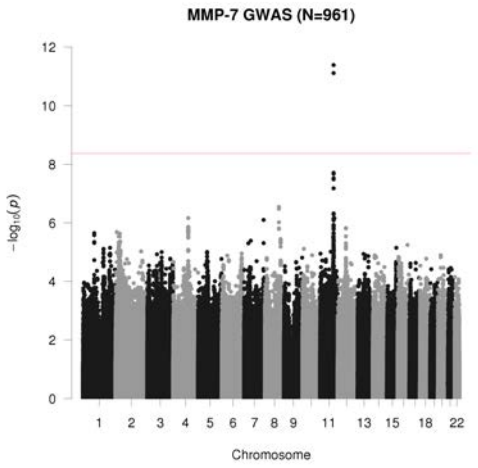
MMP-7 GWAS (N=961)
$-\log_{10}(p)$
Chromosome

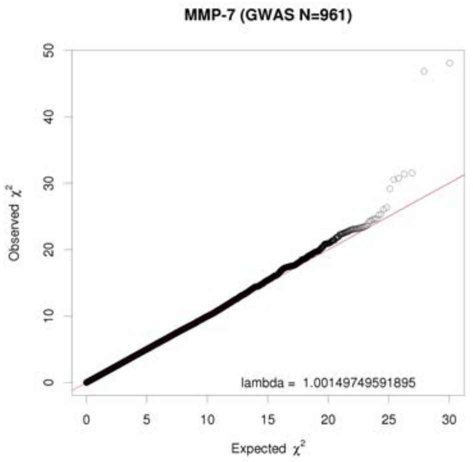
MMP-7 (GWAS N=961)
Observed χ^2
Expected χ^2
lambda = 1.00149749591895

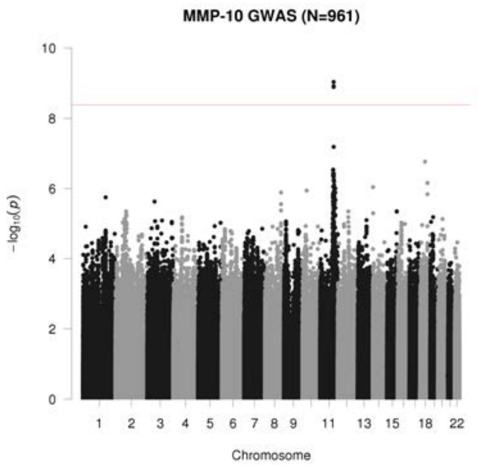
MMP-10 GWAS (N=961)
$-\log_{10}(p)$
Chromosome

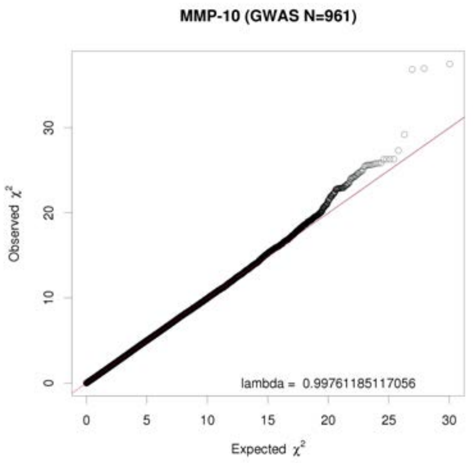
MMP-10 (GWAS N=961)
Observed χ^2
Expected χ^2
lambda = 0.99761185117056

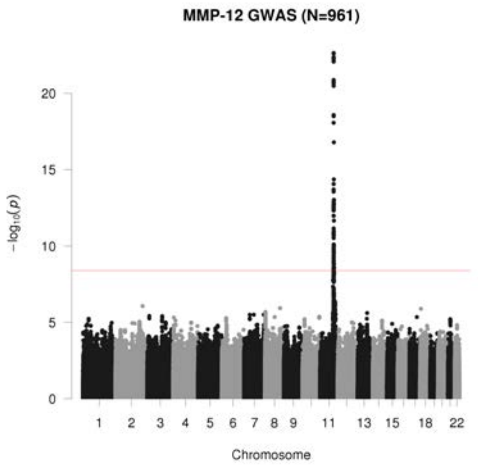
MMP-12 GWAS (N=961)
$-\log_{10}(p)$
Chromosome

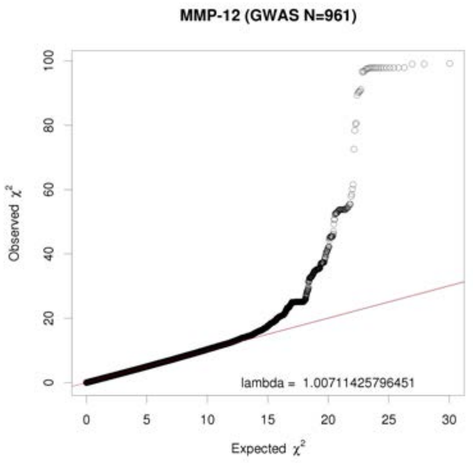
MMP-12 (GWAS N=961)
Observed χ^2
Expected χ^2
lambda = 1.00711425796451

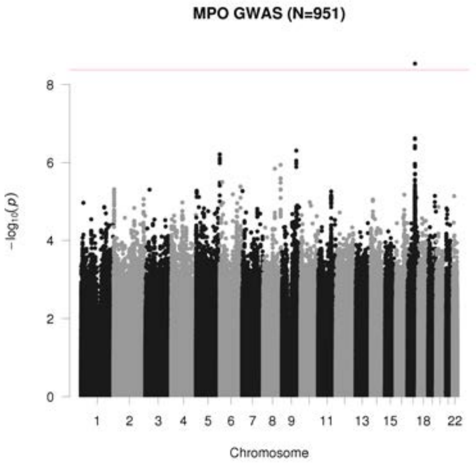
MPO GWAS (N=951)
$-\log_{10}(p)$
Chromosome

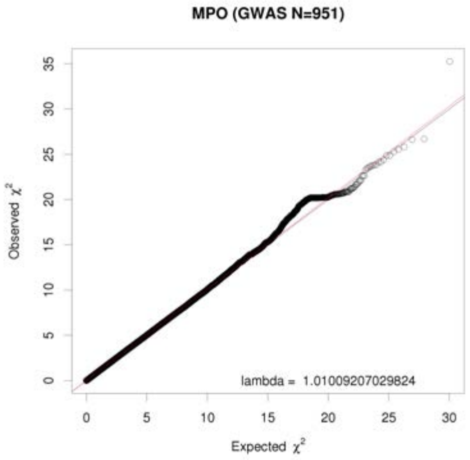
MPO (GWAS N=951)
Observed χ^2
Expected χ^2
lambda = 1.01009207029824

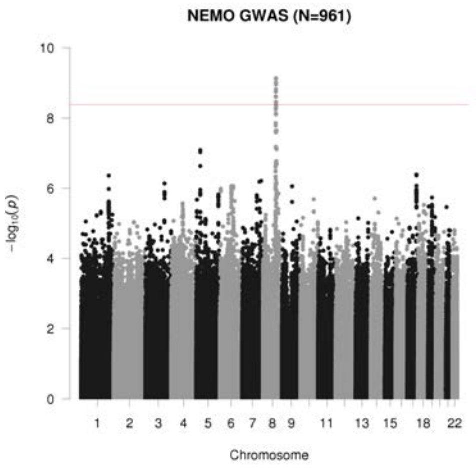
NEMO GWAS (N=961)
$-\log_{10}(p)$
Chromosome

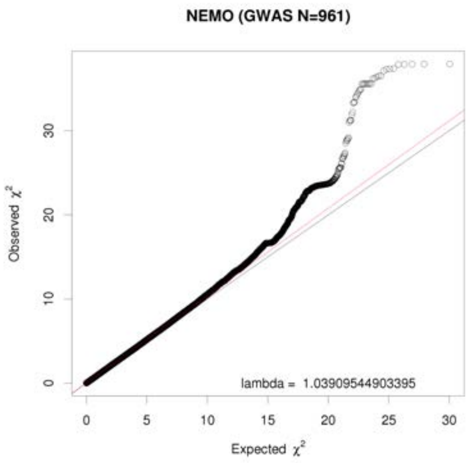
NEMO (GWAS N=961)
Observed χ^2
Expected χ^2
lambda = 1.03909544903395

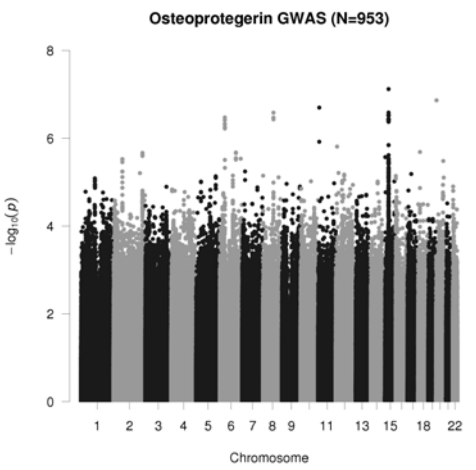
Osteoprotegerin GWAS (N=953)
$-\log_{10}(p)$
Chromosome

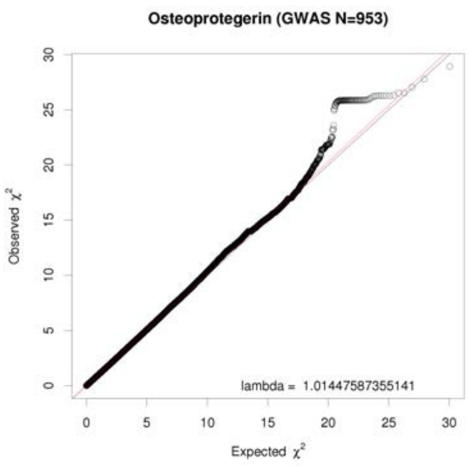
Osteoprotegerin (GWAS N=953)
Observed χ^2
Expected χ^2
lambda = 1.01447587355141

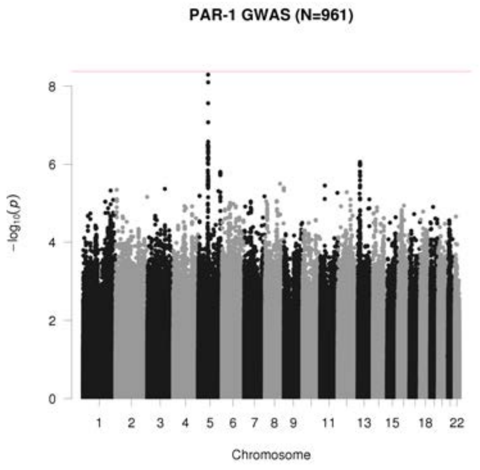
PAR-1 GWAS (N=961)
−log10(p)
Chromosome

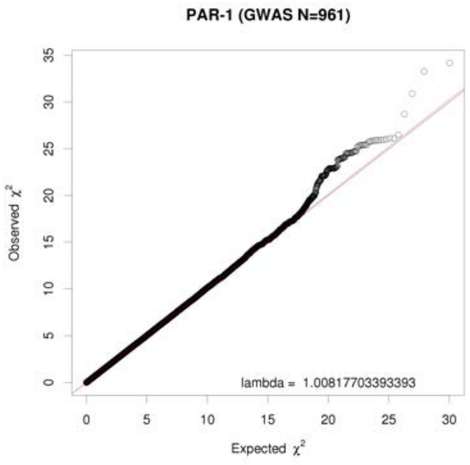
PAR-1 (GWAS N=961)
Observed χ2
Expected χ2
lambda = 1.00817703393393

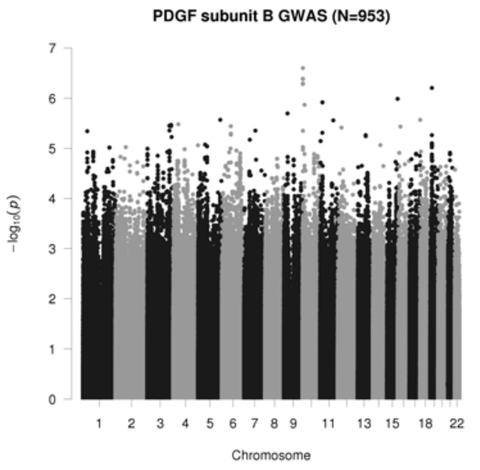
PDGF subunit B GWAS (N=953)
−log10(p)
Chromosome

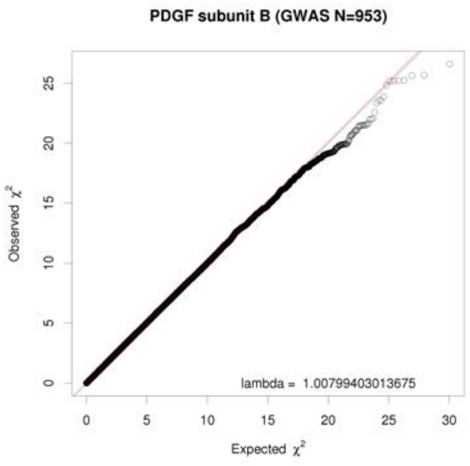
PDGF subunit B (GWAS N=953)
Observed χ2
Expected χ2
lambda = 1.00799403013675

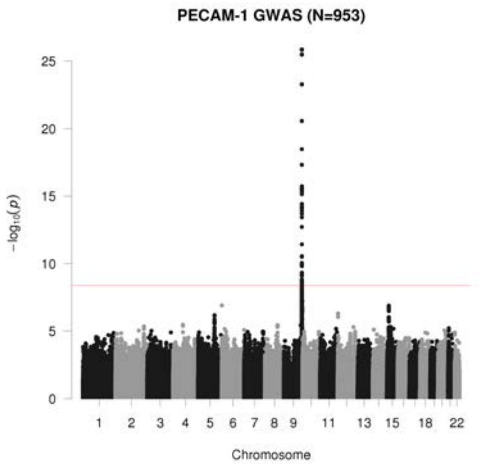
PECAM-1 GWAS (N=953)
−log10(p)
Chromosome

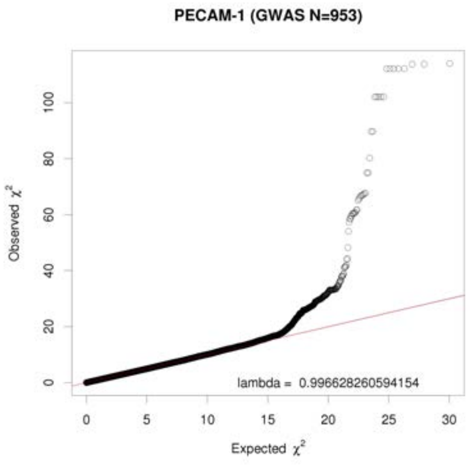
PECAM-1 (GWAS N=953)
Observed χ2
Expected χ2
lambda = 0.996628260594154

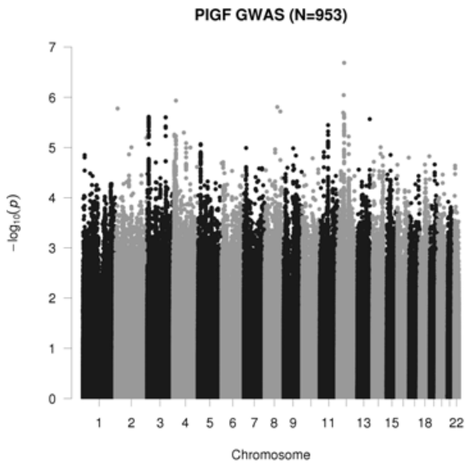
PlGF GWAS (N=953)
$-\log_{10}(p)$
Chromosome

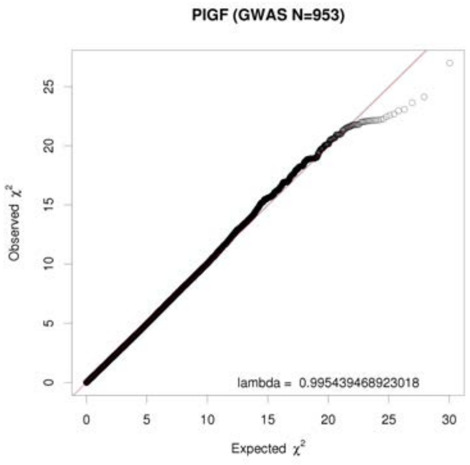
PlGF (GWAS N=953)
Observed χ^2
Expected χ^2
lambda = 0.995439468923018

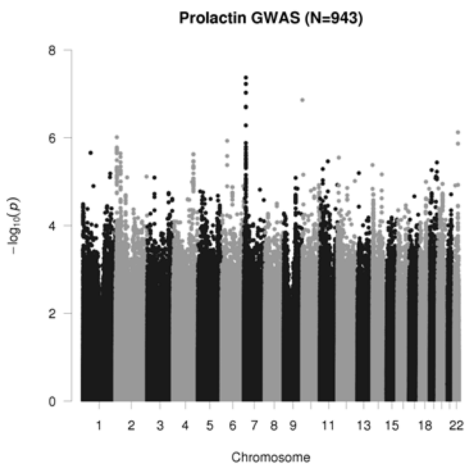
Prolactin GWAS (N=943)
$-\log_{10}(p)$
Chromosome

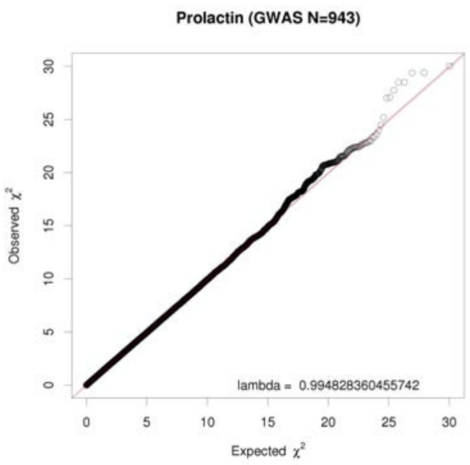
Prolactin (GWAS N=943)
Observed χ^2
Expected χ^2
lambda = 0.994828360455742

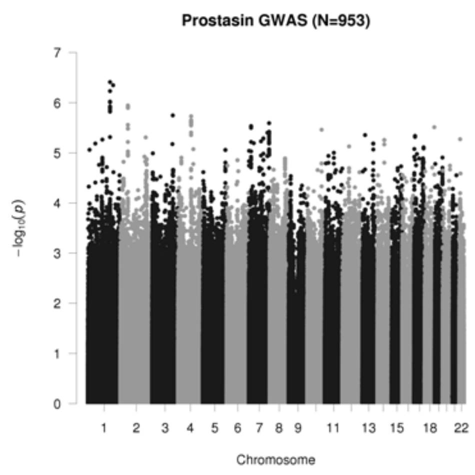
Prostasin GWAS (N=953)
$-\log_{10}(p)$
Chromosome

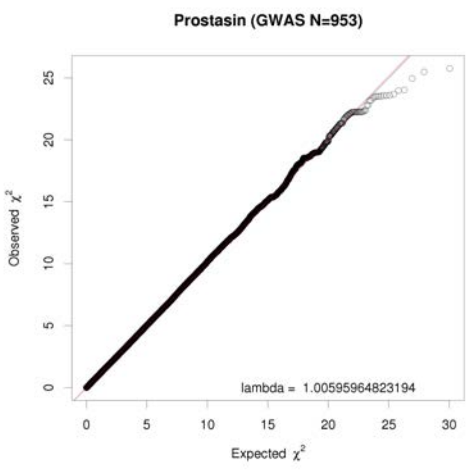
Prostasin (GWAS N=953)
Observed χ^2
Expected χ^2
lambda = 1.00595964823194

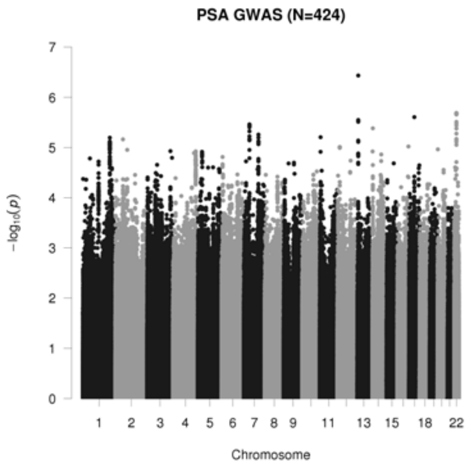
PSA GWAS (N=424)
$-\log_{10}(p)$
Chromosome

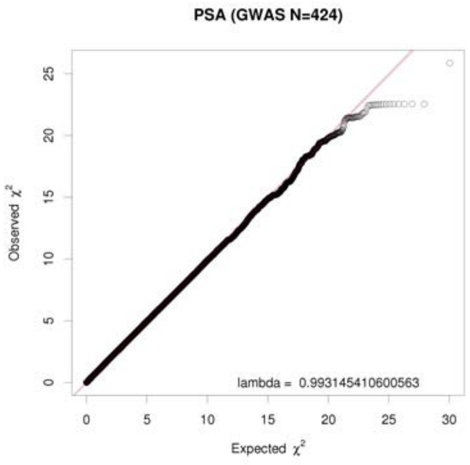
PSA (GWAS N=424)
Observed χ^2
Expected χ^2
lambda = 0.993145410600563

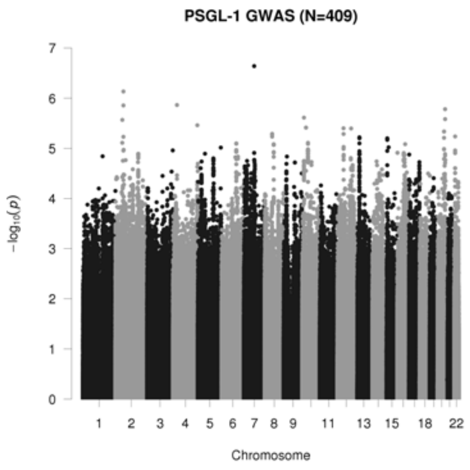
PSGL-1 GWAS (N=409)
$-\log_{10}(p)$
Chromosome

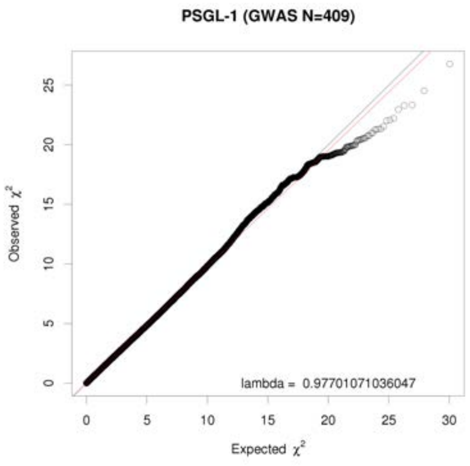
PSGL-1 (GWAS N=409)
Observed χ^2
Expected χ^2
lambda = 0.97701071036047

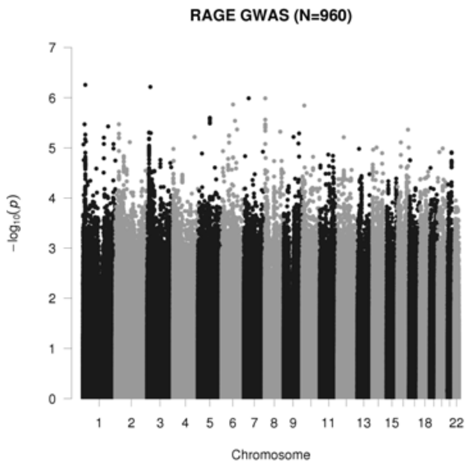
RAGE GWAS (N=960)
$-\log_{10}(p)$
Chromosome

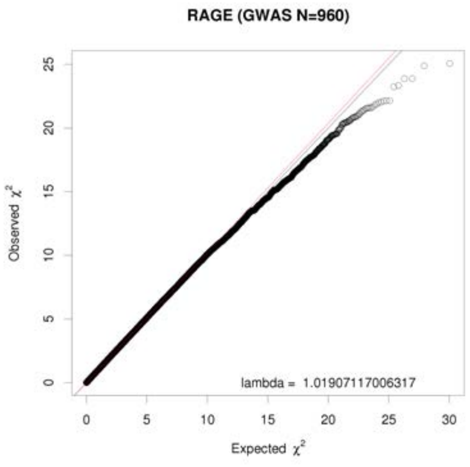
RAGE (GWAS N=960)
Observed χ^2
Expected χ^2
lambda = 1.01907117006317

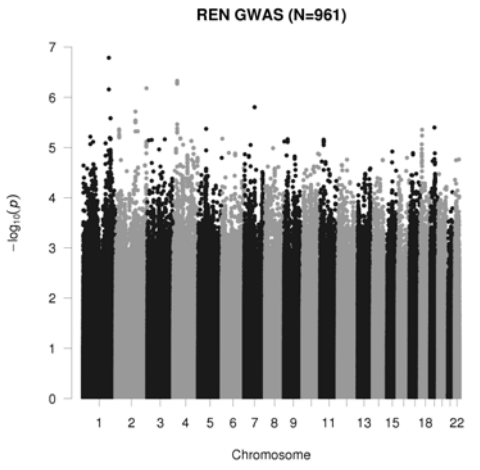
REN GWAS (N=961)
$-\log_{10}(p)$
Chromosome

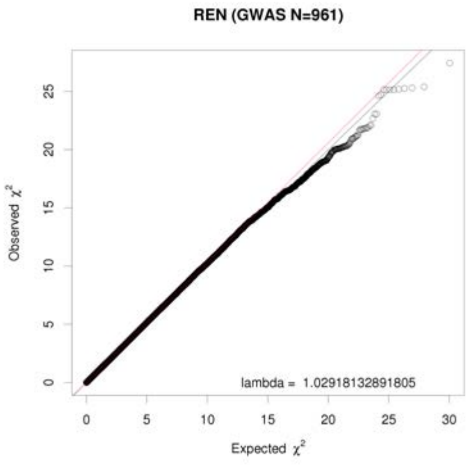
REN (GWAS N=961)
Observed χ^2
Expected χ^2
lambda = 1.02918132891805

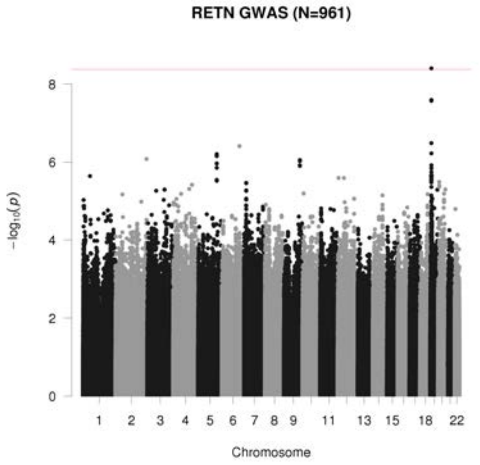
RETN GWAS (N=961)
$-\log_{10}(p)$
Chromosome

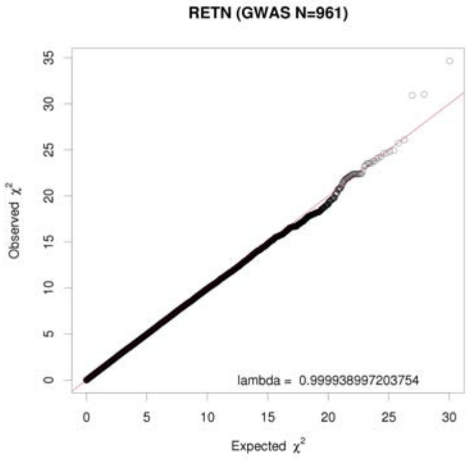
RETN (GWAS N=961)
Observed χ^2
Expected χ^2
lambda = 0.999938997203754

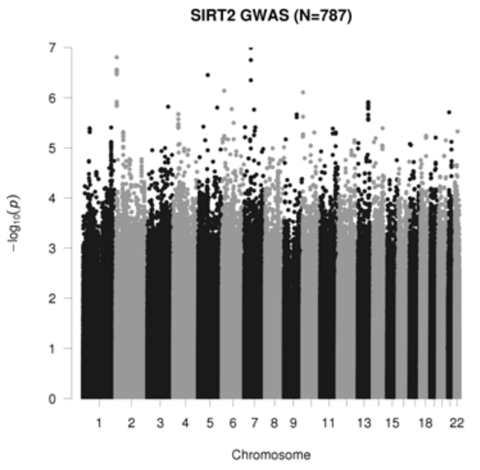
SIRT2 GWAS (N=787)
$-\log_{10}(p)$
Chromosome

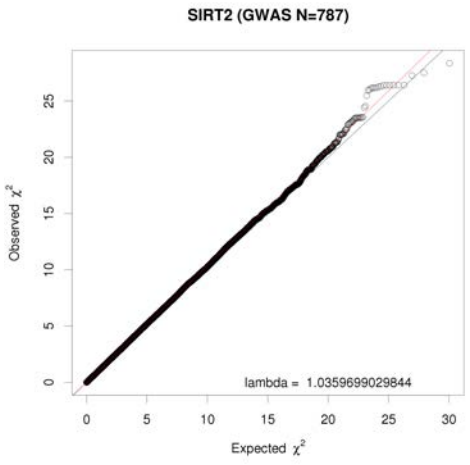
SIRT2 (GWAS N=787)
Observed χ^2
Expected χ^2
lambda = 1.0359699029844

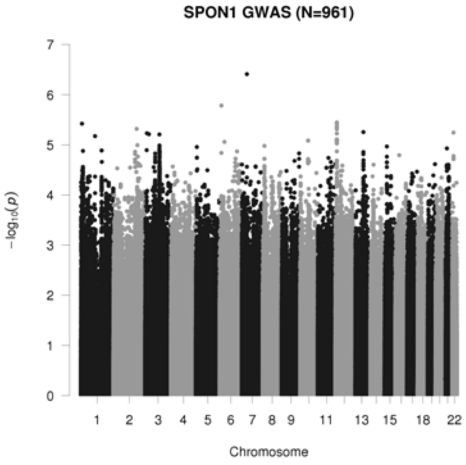
SPON1 GWAS (N=961)
−log10(p)
Chromosome

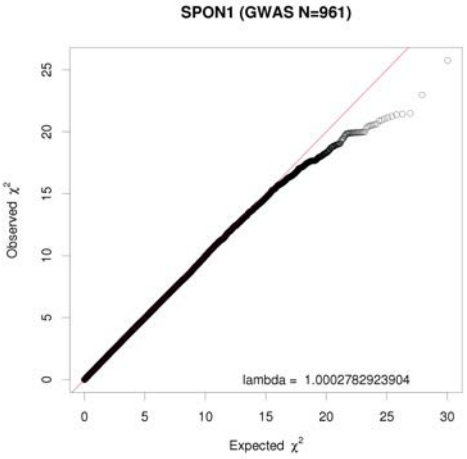
SPON1 (GWAS N=961)
Observed χ²
Expected χ²
lambda = 1.0002782923904

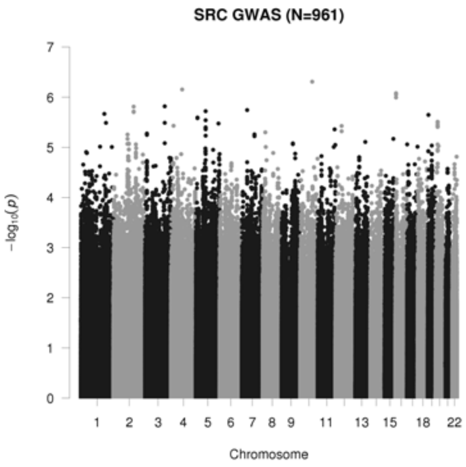
SRC GWAS (N=961)
−log10(p)
Chromosome

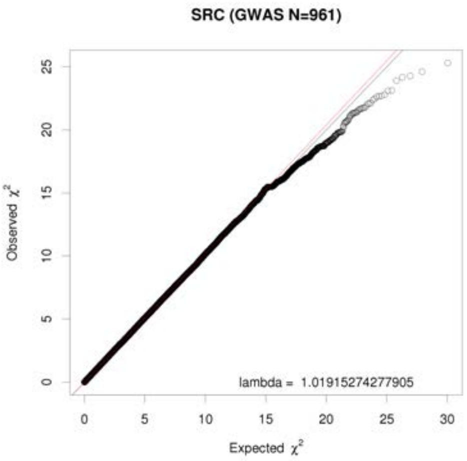
SRC (GWAS N=961)
Observed χ²
Expected χ²
lambda = 1.01915274277905

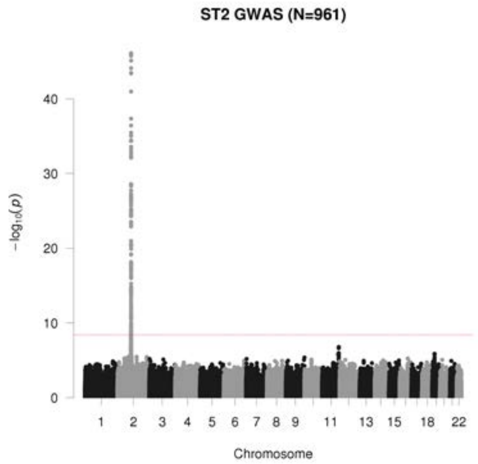
ST2 GWAS (N=961)
−log10(p)
Chromosome

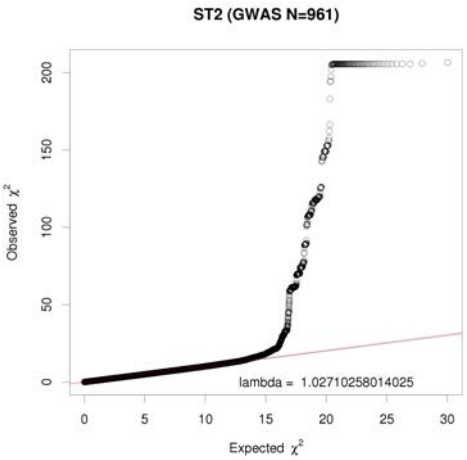
ST2 (GWAS N=961)
Observed χ²
Expected χ²
lambda = 1.02710258014025

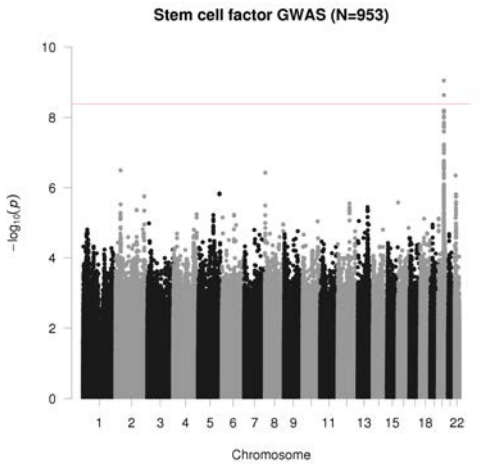
Stem cell factor GWAS (N=953)
$-\log_{10}(p)$
Chromosome

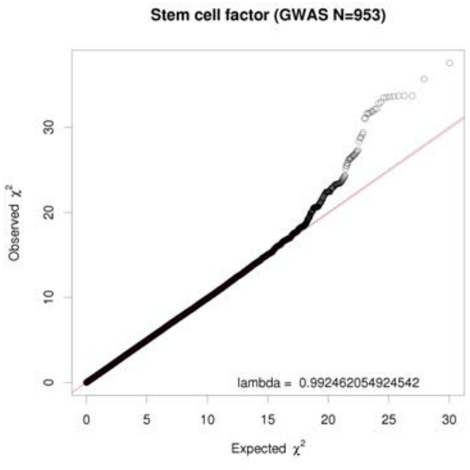
Stem cell factor (GWAS N=953)
Observed χ^2
Expected χ^2
lambda = 0.992462054924542

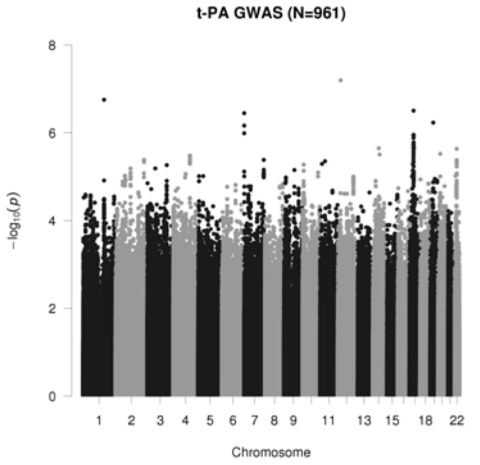
t-PA GWAS (N=961)
$-\log_{10}(p)$
Chromosome

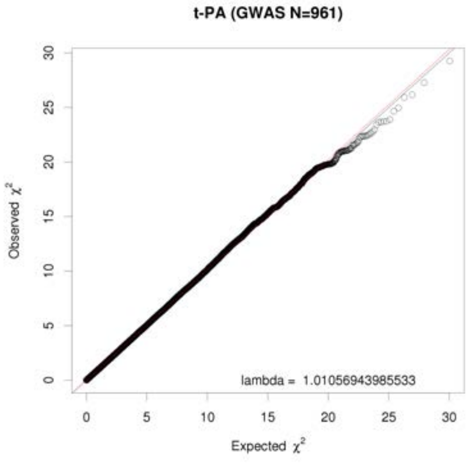
t-PA (GWAS N=961)
Observed χ^2
Expected χ^2
lambda = 1.01056943985533

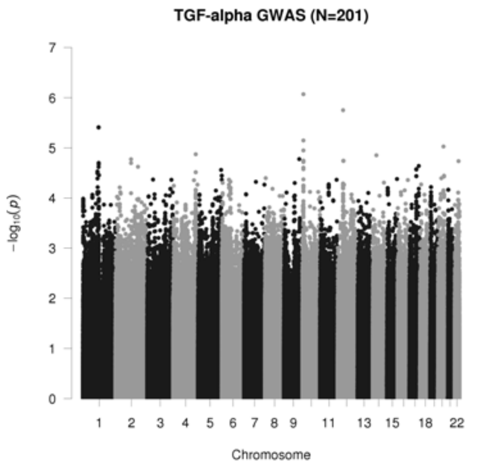
TGF-alpha GWAS (N=201)
$-\log_{10}(p)$
Chromosome

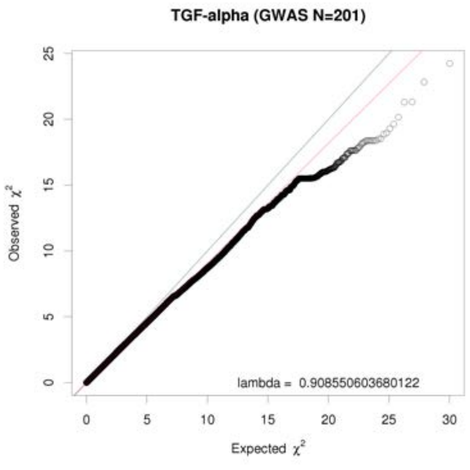
TGF-alpha (GWAS N=201)
Observed χ^2
Expected χ^2
lambda = 0.908550603680122

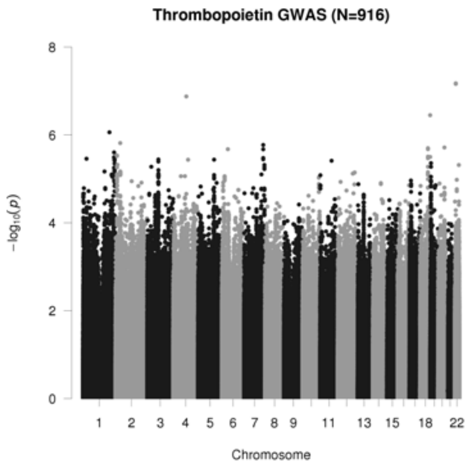
Thrombopoietin GWAS (N=916)
−log10(p)
Chromosome

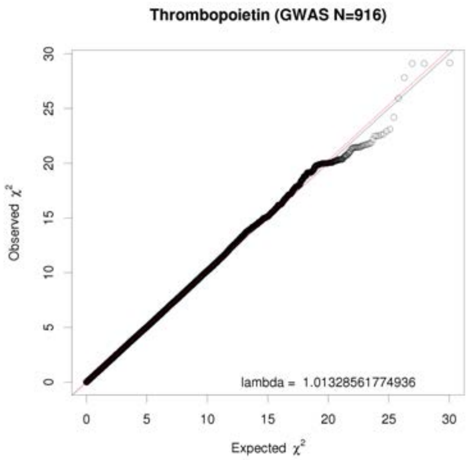
Thrombopoietin (GWAS N=916)
Observed χ2
Expected χ2
lambda = 1.01328561774936

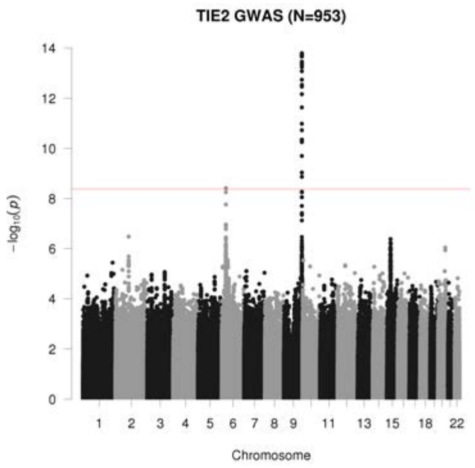
TIE2 GWAS (N=953)
−log10(p)
Chromosome

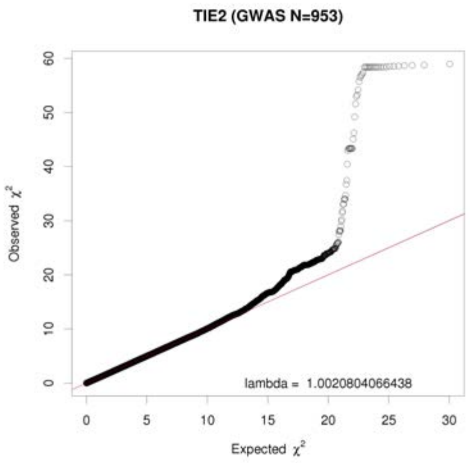
TIE2 (GWAS N=953)
Observed χ2
Expected χ2
lambda = 1.0020804066438

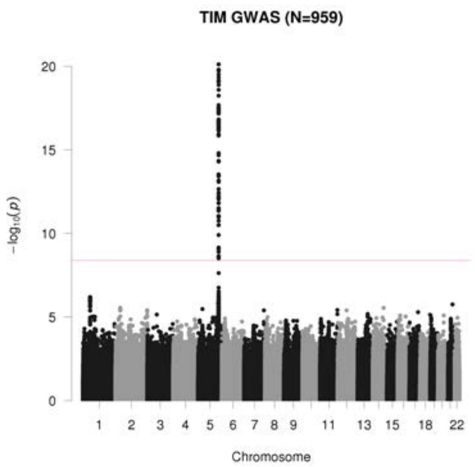
TIM GWAS (N=959)
−log10(p)
Chromosome

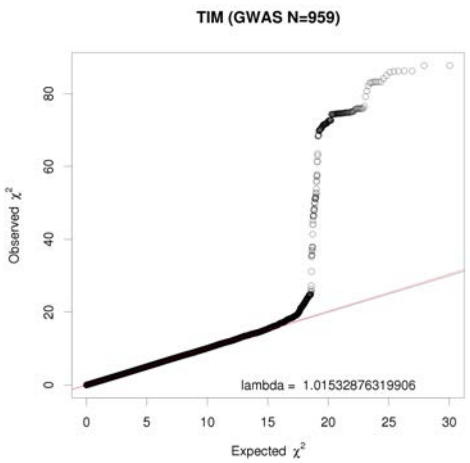
TIM (GWAS N=959)
Observed χ2
Expected χ2
lambda = 1.01532876319906

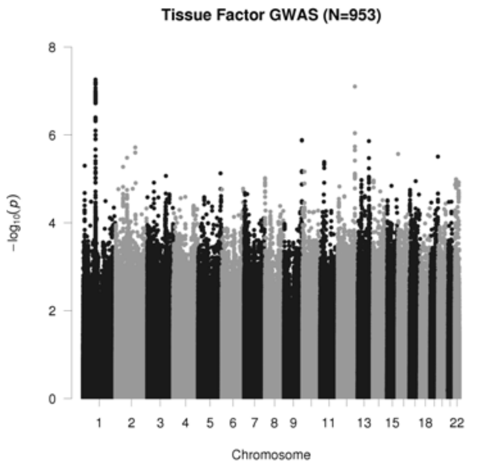
Tissue Factor GWAS (N=953)
$-\log_{10}(p)$
Chromosome

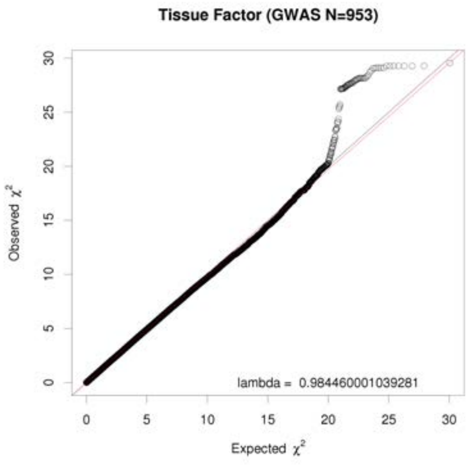
Tissue Factor (GWAS N=953)
Observed χ^2
Expected χ^2
lambda = 0.984460001039281

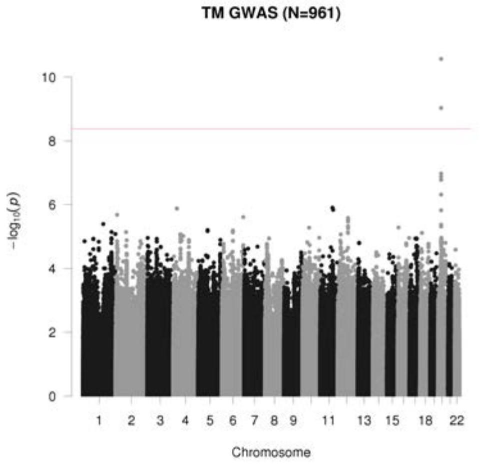
TM GWAS (N=961)
$-\log_{10}(p)$
Chromosome

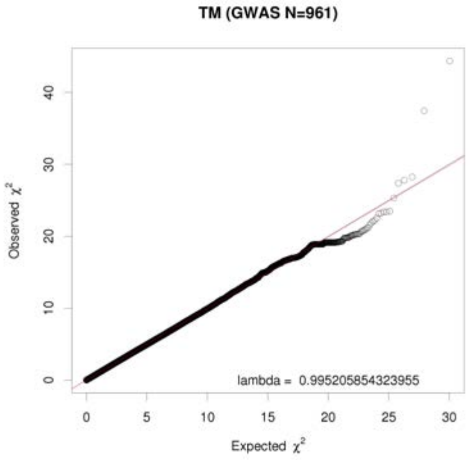
TM (GWAS N=961)
Observed χ^2
Expected χ^2
lambda = 0.995205854323955

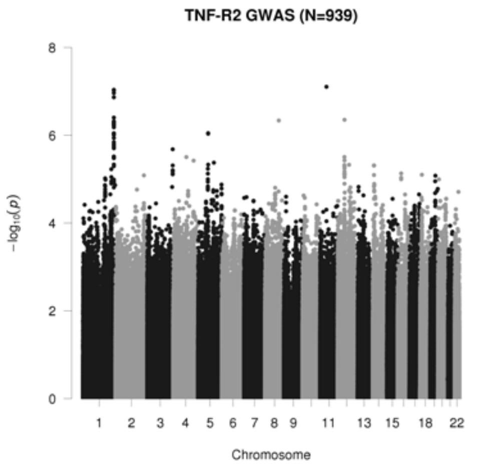
TNF-R2 GWAS (N=939)
$-\log_{10}(p)$
Chromosome

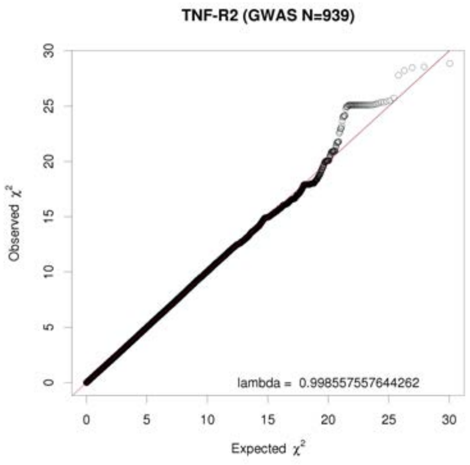
TNF-R2 (GWAS N=939)
Observed χ^2
Expected χ^2
lambda = 0.998557557644262

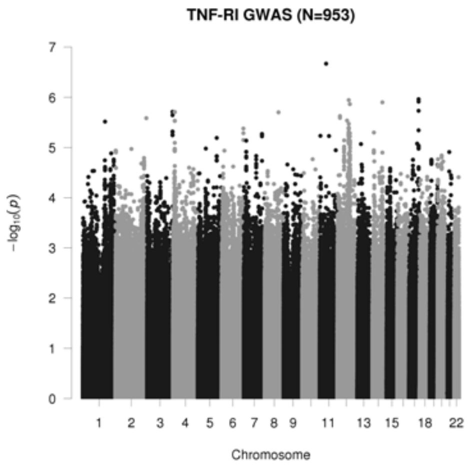
TNF-RI GWAS (N=953)
$-\log_{10}(p)$
Chromosome

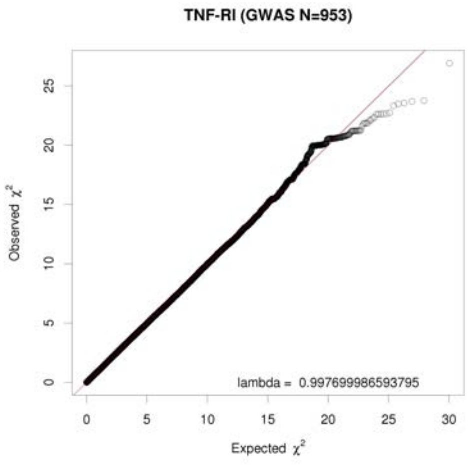
TNF-RI (GWAS N=953)
Observed χ^2
Expected χ^2
lambda = 0.997699986593795

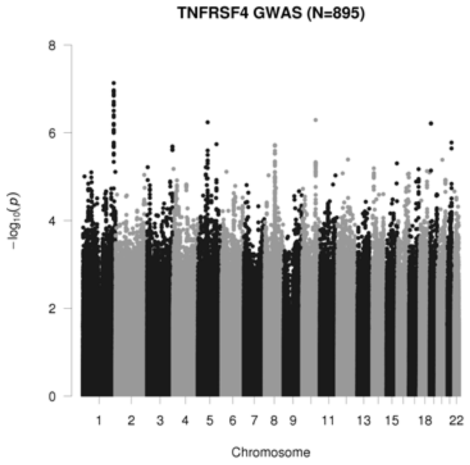
TNFRSF4 GWAS (N=895)
$-\log_{10}(p)$
Chromosome

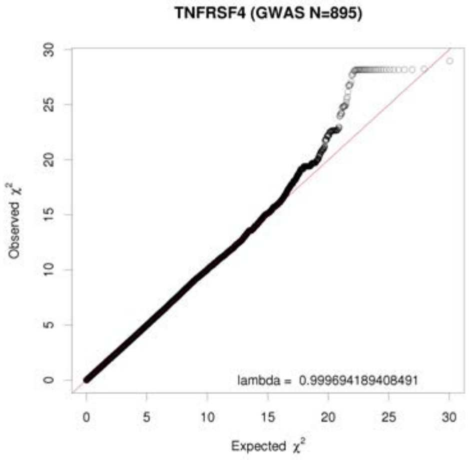
TNFRSF4 (GWAS N=895)
Observed χ^2
Expected χ^2
lambda = 0.999694189408491

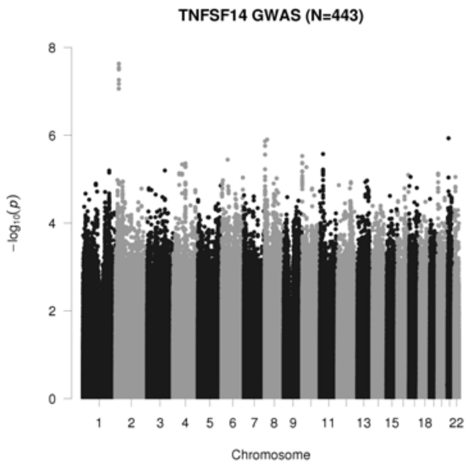
TNFSF14 GWAS (N=443)
$-\log_{10}(p)$
Chromosome

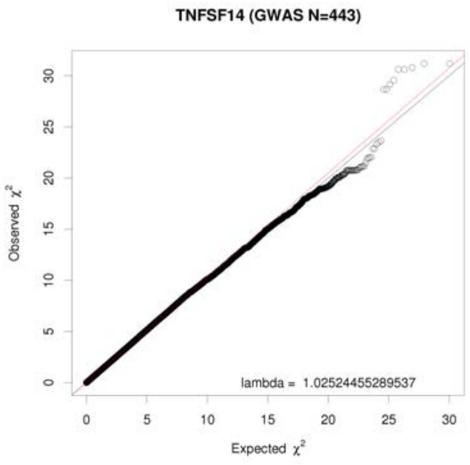
TNFSF14 (GWAS N=443)
Observed χ^2
Expected χ^2
lambda = 1.02524455289537

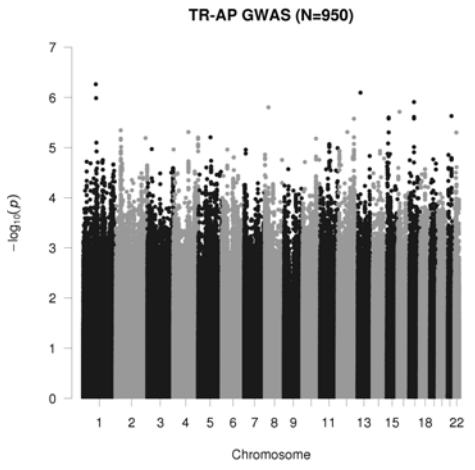
TR-AP GWAS (N=950)
$-\log_{10}(p)$
Chromosome

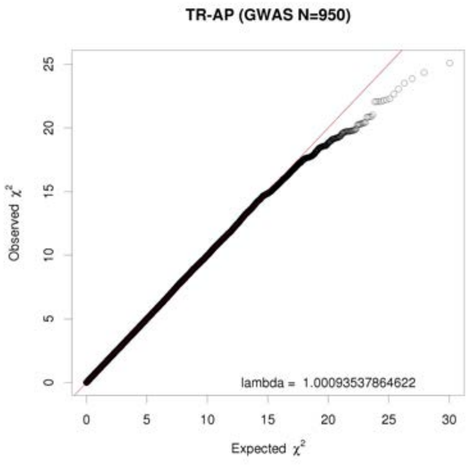
TR-AP (GWAS N=950)
Observed χ^2
Expected χ^2
lambda = 1.00093537864622

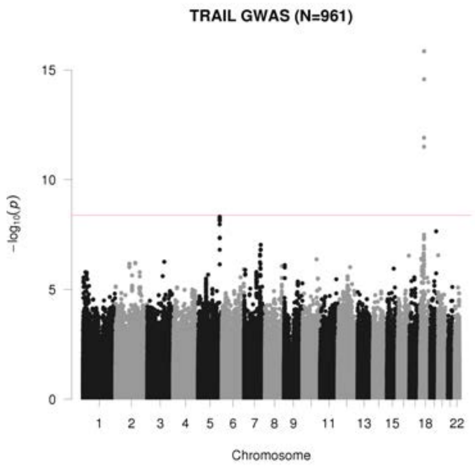
TRAIL GWAS (N=961)
$-\log_{10}(p)$
Chromosome

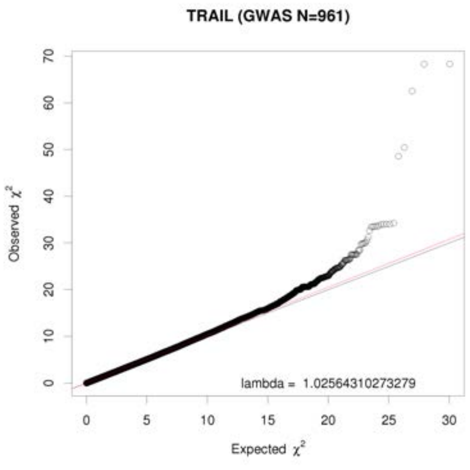
TRAIL (GWAS N=961)
Observed χ^2
Expected χ^2
lambda = 1.02564310273279

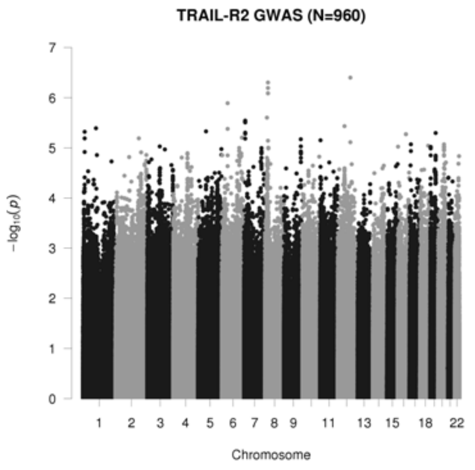
TRAIL-R2 GWAS (N=960)
$-\log_{10}(p)$
Chromosome

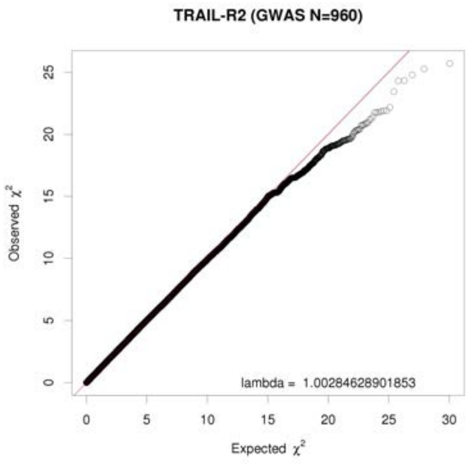
TRAIL-R2 (GWAS N=960)
Observed χ^2
Expected χ^2
lambda = 1.00284628901853

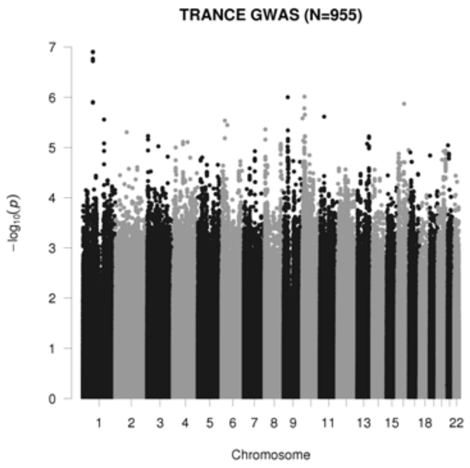
TRANCE GWAS (N=955)
$-\log_{10}(p)$
Chromosome

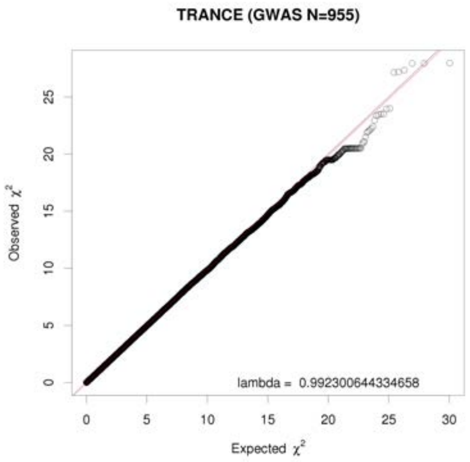
TRANCE (GWAS N=955)
Observed χ^2
Expected χ^2
lambda = 0.992300644334658

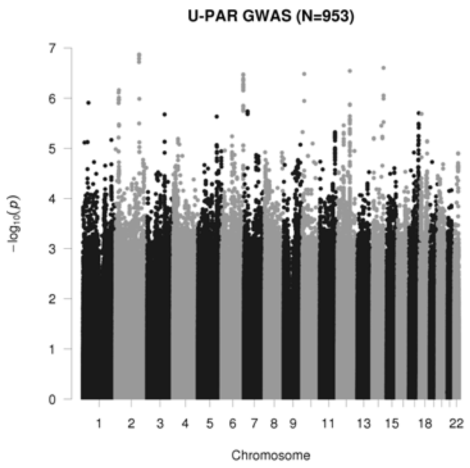
U-PAR GWAS (N=953)
$-\log_{10}(p)$
Chromosome

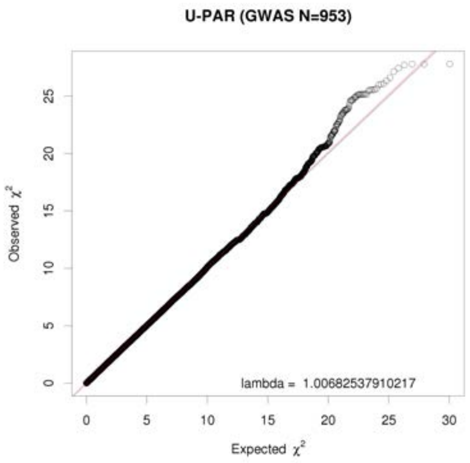
U-PAR (GWAS N=953)
Observed χ^2
Expected χ^2
lambda = 1.00682537910217

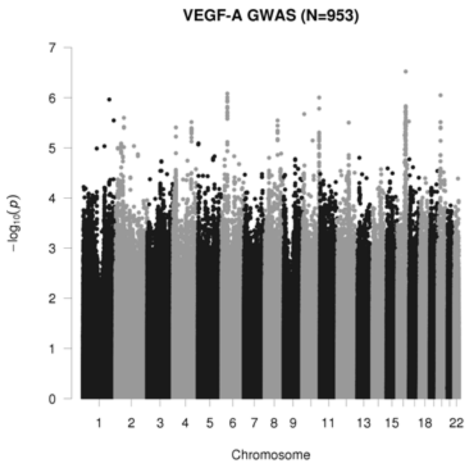
VEGF-A GWAS (N=953)
$-\log_{10}(p)$
Chromosome

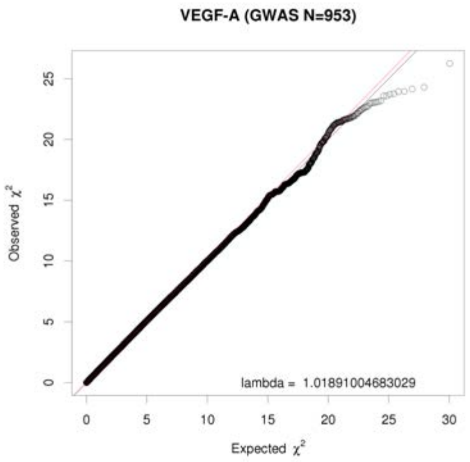
VEGF-A (GWAS N=953)
Observed χ^2
Expected χ^2
lambda = 1.01891004683029

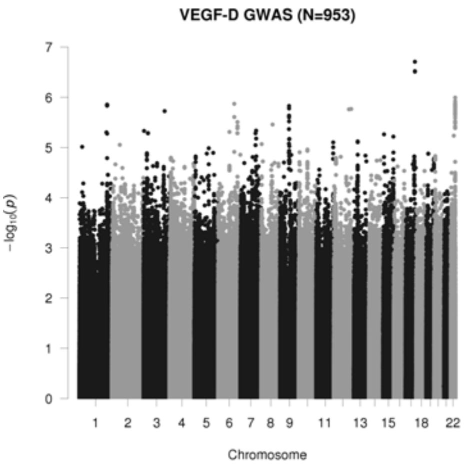
VEGF-D GWAS (N=953)
$-\log_{10}(p)$
Chromosome
0
1
2
3
4
5
6
7
1 2 3 4 5 6 7 8 9 11 13 15 18 22

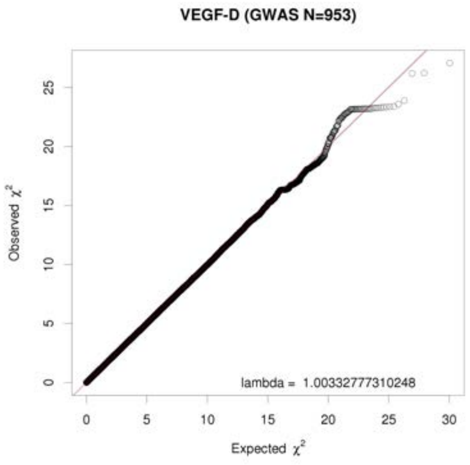
VEGF-D (GWAS N=953)
Observed χ^2
Expected χ^2
lambda = 1.00332777310248
0 5 10 15 20 25
0 5 10 15 20 25 30

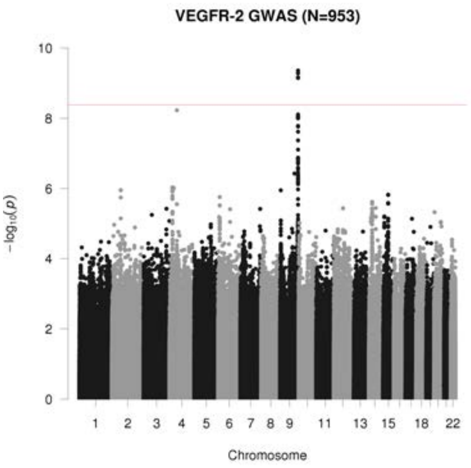
VEGFR-2 GWAS (N=953)
$-\log_{10}(p)$
Chromosome
0
2
4
6
8
10
1 2 3 4 5 6 7 8 9 11 13 15 18 22

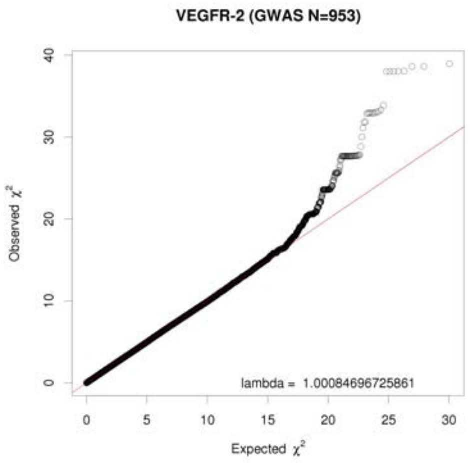
VEGFR-2 (GWAS N=953)
Observed χ^2
Expected χ^2
lambda = 1.00084696725861
0 10 20 30 40
0 5 10 15 20 25 30
