# Supplementary material for: The relative contribution of DNA methylation and genetic variants on protein biomarkers for human diseases
Source: PLoS Genet. 2017 Sep 15;13(9):e1007005. doi: 10.1371/journal.pgen.1007005 (PMC5617224; doi:10.1371/journal.pgen.1007005)
Supplement: S3 Fig — (PDF) [file pgen.1007005.s003.pdf]

***Supplemental Fig S3**. Manhattan plots and QQ plots for EWAS results for all analyzed biomarkers.*

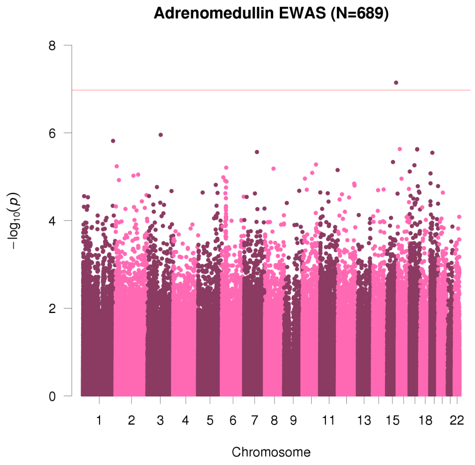

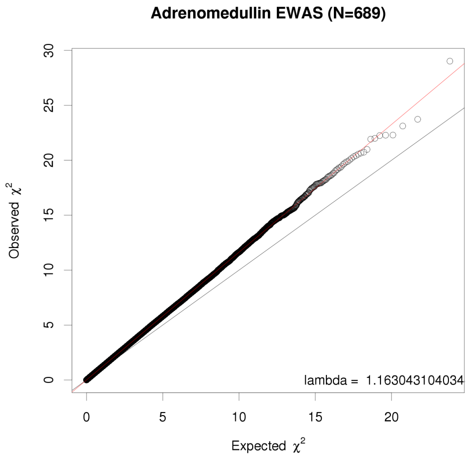

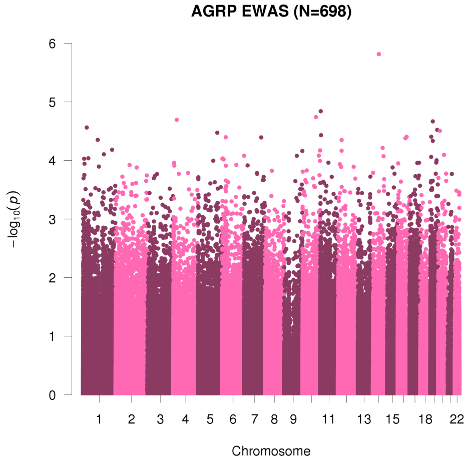

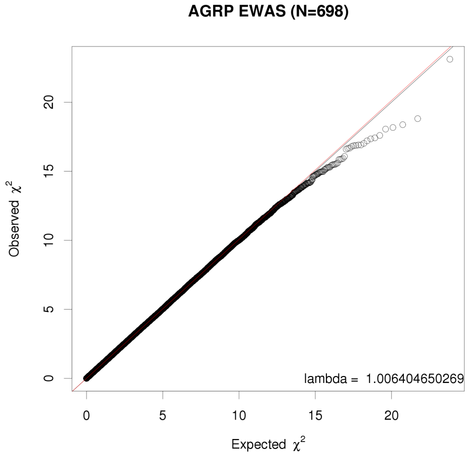

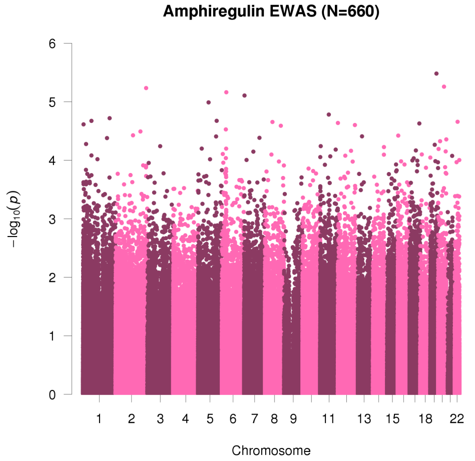

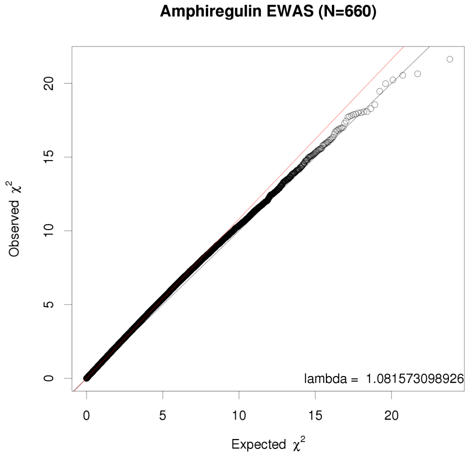

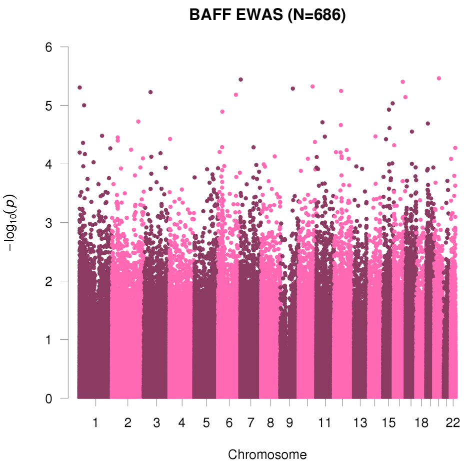
BAFF EWAS (N=686)
$-\log_{10}(p)$
0 1 2 3 4 5 6
1 2 3 4 5 6 7 8 9 11 13 15 18 22
Chromosome

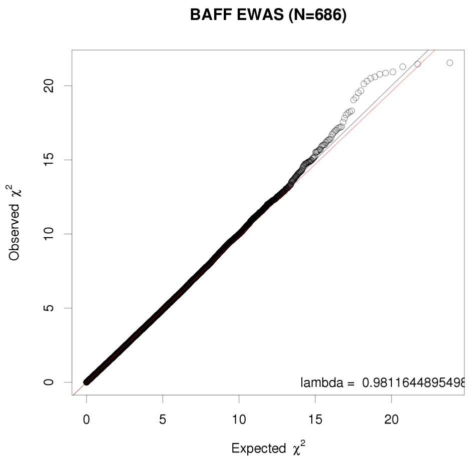
BAFF EWAS (N=686)
Observed χ^2
0 5 10 15 20
lambda = 0.9811644895498
0 5 10 15 20
Expected χ^2

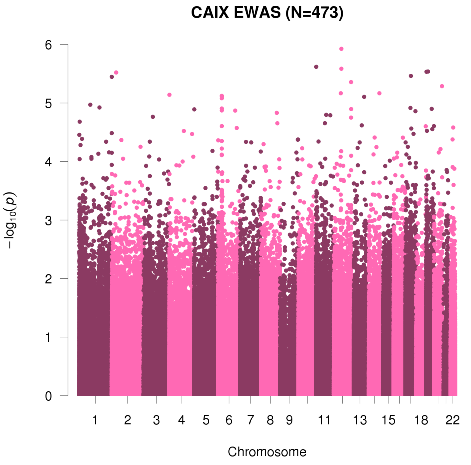
CAIX EWAS (N=473)
$-\log_{10}(p)$
0 1 2 3 4 5 6
1 2 3 4 5 6 7 8 9 11 13 15 18 22
Chromosome

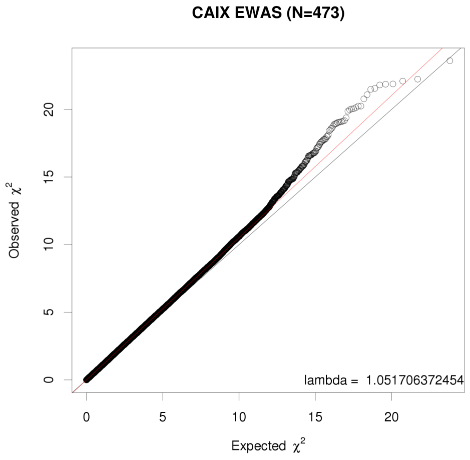
CAIX EWAS (N=473)
Observed χ^2
0 5 10 15 20
lambda = 1.051706372454
0 5 10 15 20
Expected χ^2

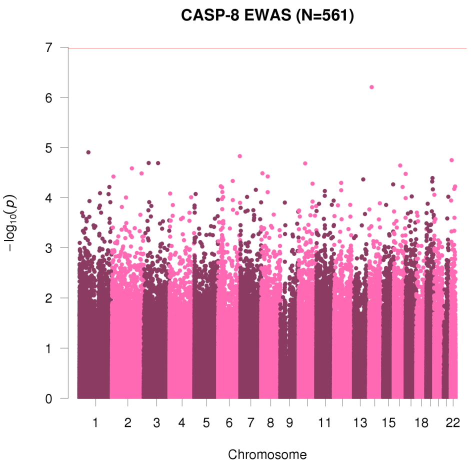
CASP-8 EWAS (N=561)
$-\log_{10}(p)$
0 1 2 3 4 5 6 7
1 2 3 4 5 6 7 8 9 11 13 15 18 22
Chromosome

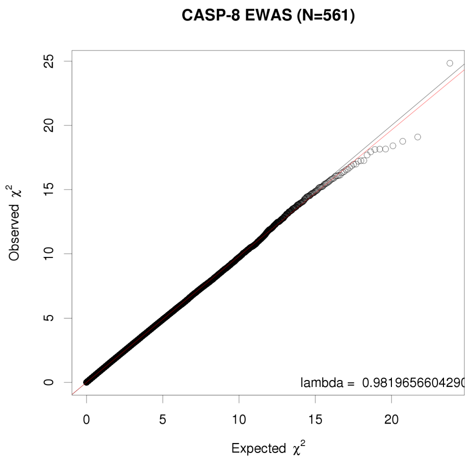
CASP-8 EWAS (N=561)
Observed χ^2
0 5 10 15 20 25
lambda = 0.9819656604290
0 5 10 15 20
Expected χ^2

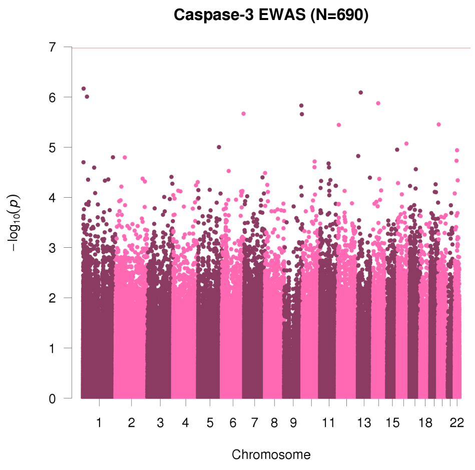
Caspase-3 EWAS (N=690)
$-\log_{10}(p)$
Chromosome

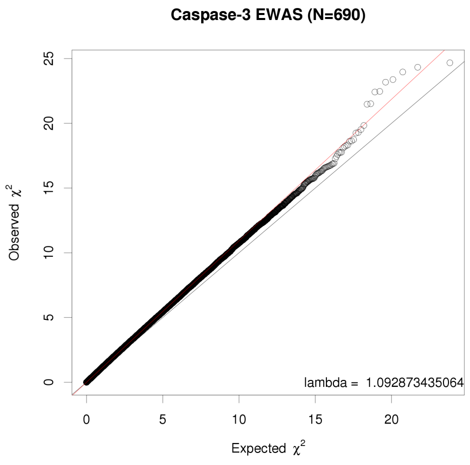
Caspase-3 EWAS (N=690)
Observed χ^2
Expected χ^2
lambda = 1.092873435064

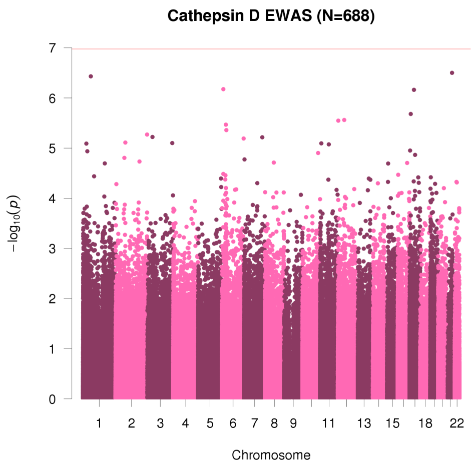
Cathepsin D EWAS (N=688)
$-\log_{10}(p)$
Chromosome

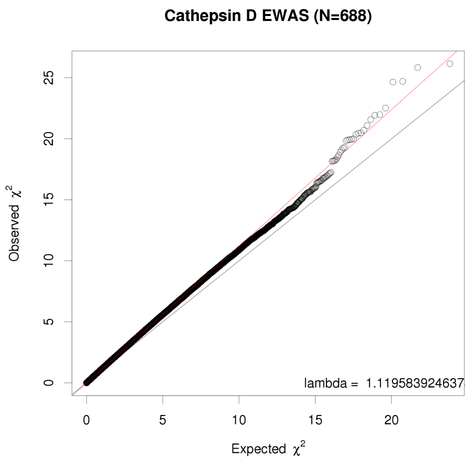
Cathepsin D EWAS (N=688)
Observed χ^2
Expected χ^2
lambda = 1.119583924637

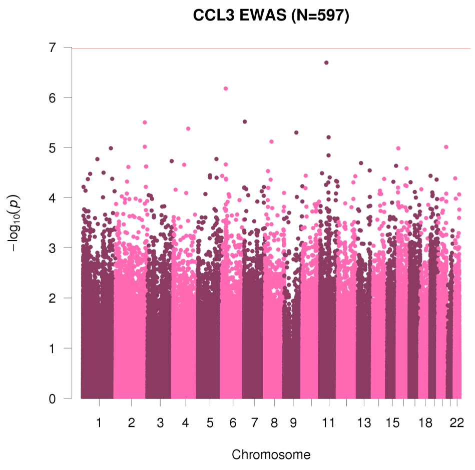
CCL3 EWAS (N=597)
$-\log_{10}(p)$
Chromosome

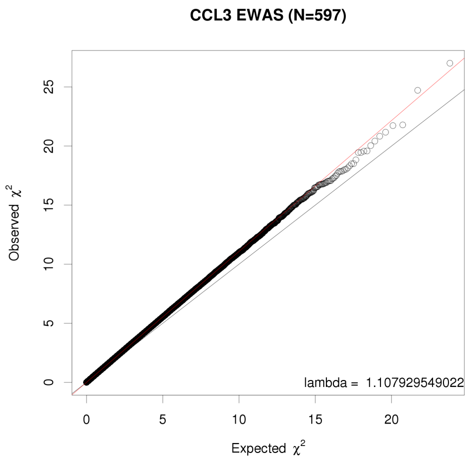
CCL3 EWAS (N=597)
Observed χ^2
Expected χ^2
lambda = 1.107929549022

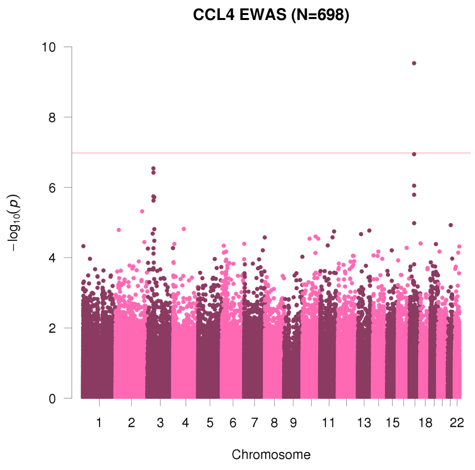
CCL4 EWAS (N=698)
10
8
6
4
2
0
$-\log_{10}(p)$
1 2 3 4 5 6 7 8 9 11 13 15 18 22
Chromosome

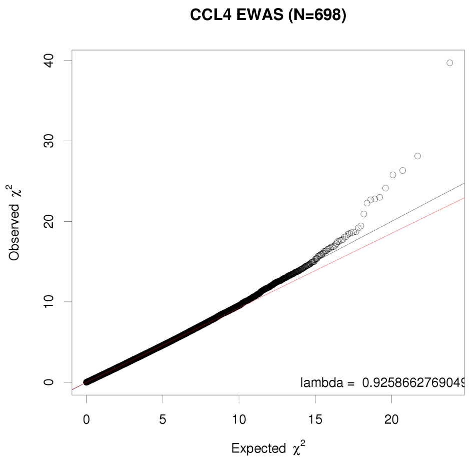
CCL4 EWAS (N=698)
40
30
20
10
0
Observed χ^2
lambda = 0.925866276904
0 5 10 15 20
Expected χ^2

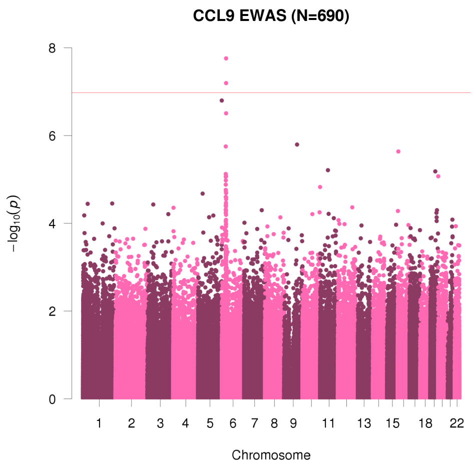
CCL9 EWAS (N=690)
8
6
4
2
0
$-\log_{10}(p)$
1 2 3 4 5 6 7 8 9 11 13 15 18 22
Chromosome

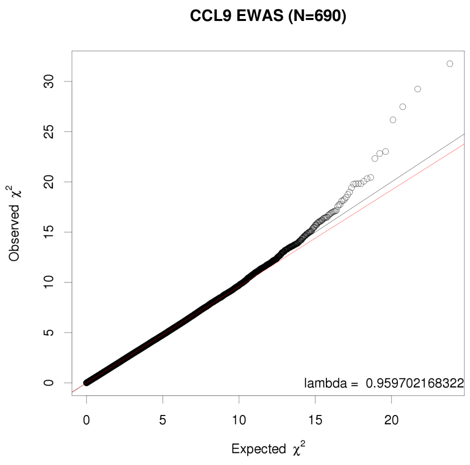
CCL9 EWAS (N=690)
30
25
20
15
10
5
0
Observed χ^2
lambda = 0.959702168322
0 5 10 15 20
Expected χ^2

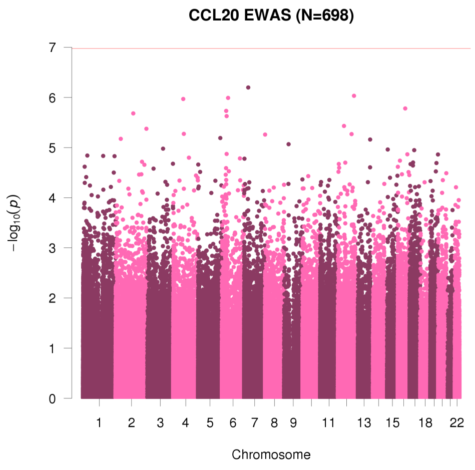
CCL20 EWAS (N=698)
7
6
5
4
3
2
1
0
$-\log_{10}(p)$
1 2 3 4 5 6 7 8 9 11 13 15 18 22
Chromosome

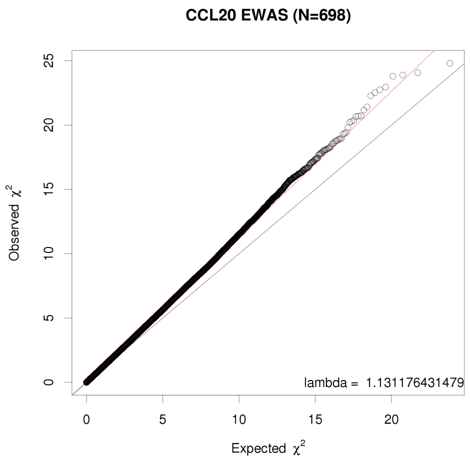
CCL20 EWAS (N=698)
25
20
15
10
5
0
Observed χ^2
lambda = 1.131176431479
0 5 10 15 20
Expected χ^2

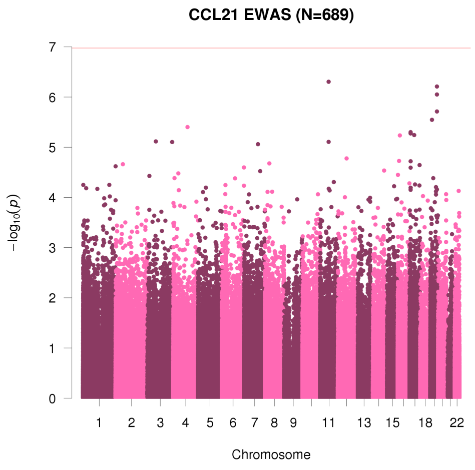
CCL21 EWAS (N=689)
−log10(p)
Chromosome
1 2 3 4 5 6 7 8 9 11 13 15 18 22

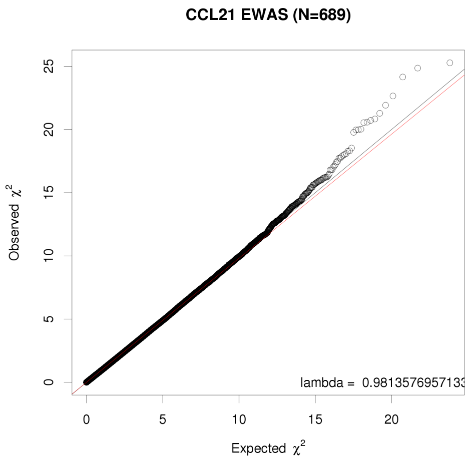
CCL21 EWAS (N=689)
Observed χ²
Expected χ²
lambda = 0.981357695713

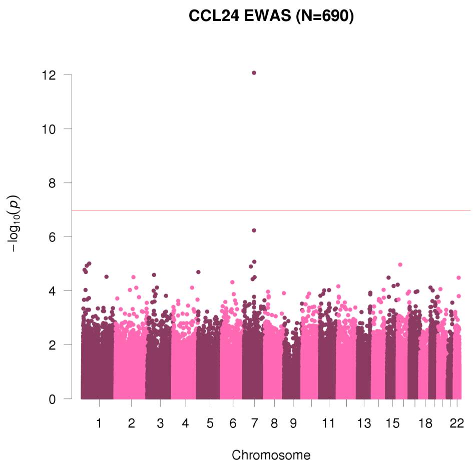
CCL24 EWAS (N=690)
−log10(p)
Chromosome
1 2 3 4 5 6 7 8 9 11 13 15 18 22

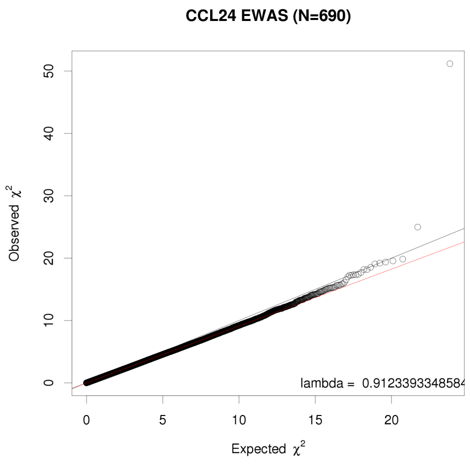
CCL24 EWAS (N=690)
Observed χ²
Expected χ²
lambda = 0.912339334858

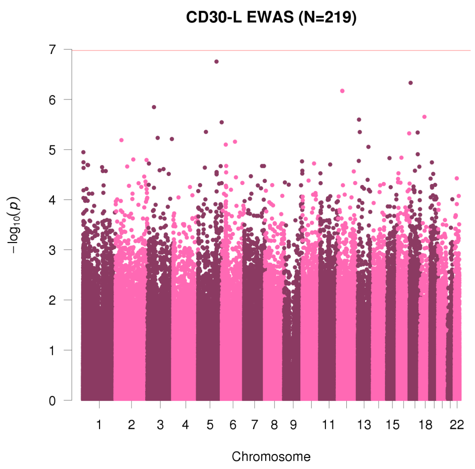
CD30-L EWAS (N=219)
−log10(p)
Chromosome
1 2 3 4 5 6 7 8 9 11 13 15 18 22

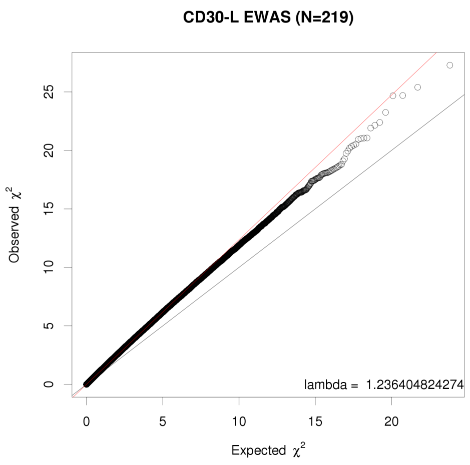
CD30-L EWAS (N=219)
Observed χ²
Expected χ²
lambda = 1.236404824274

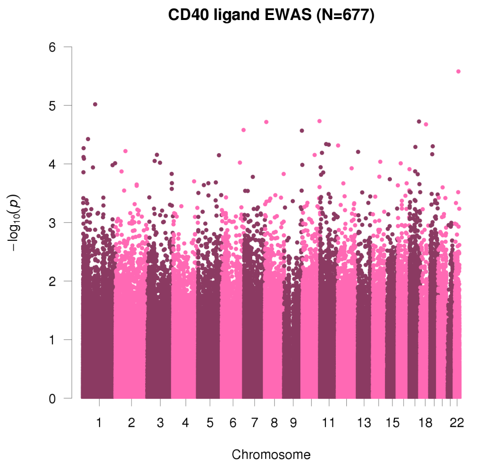
CD40 ligand EWAS (N=677)
−log10(p)
Chromosome

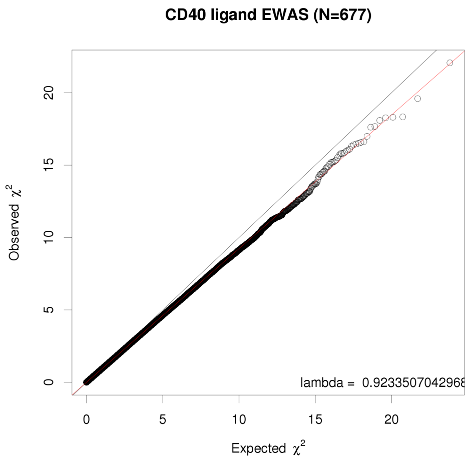
CD40 ligand EWAS (N=677)
Observed χ2
Expected χ2
lambda = 0.9233507042968

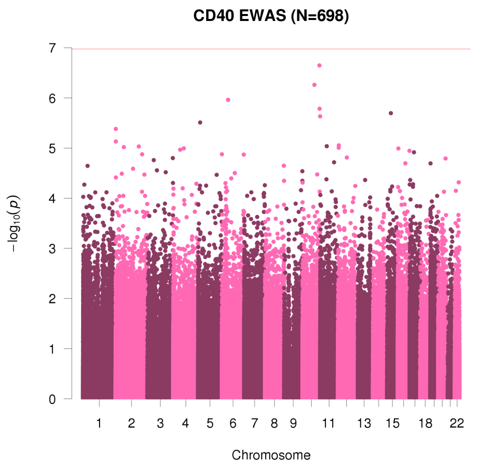
CD40 EWAS (N=698)
−log10(p)
Chromosome

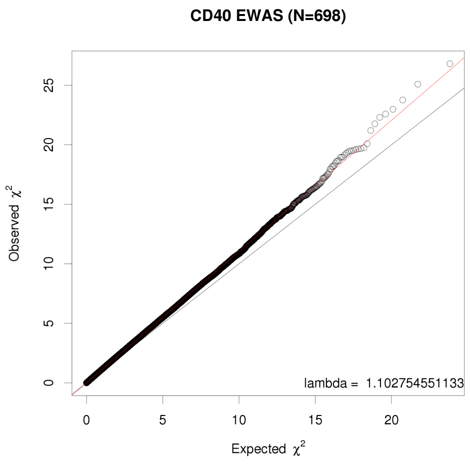
CD40 EWAS (N=698)
Observed χ2
Expected χ2
lambda = 1.102754551133

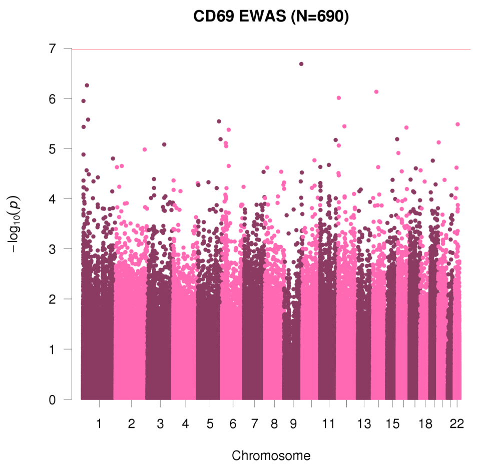
CD69 EWAS (N=690)
−log10(p)
Chromosome

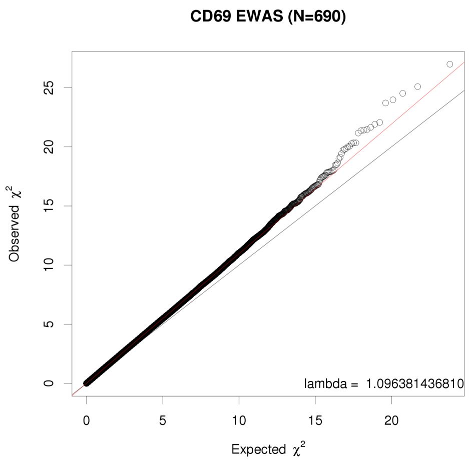
CD69 EWAS (N=690)
Observed χ2
Expected χ2
lambda = 1.096381436810

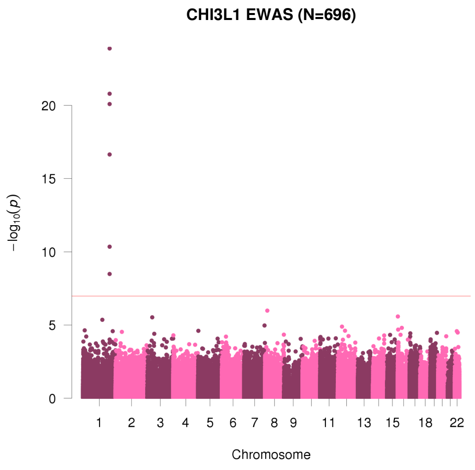
CHI3L1 EWAS (N=696)
$-\log_{10}(p)$
Chromosome

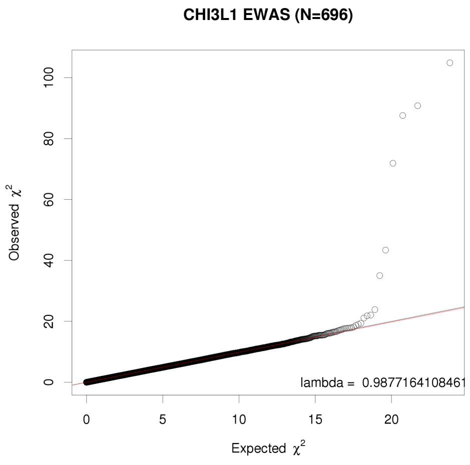
CHI3L1 EWAS (N=696)
Observed χ^2
Expected χ^2
lambda = 0.9877164108461

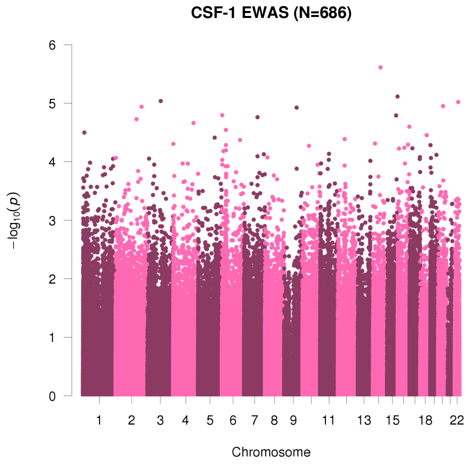
CSF-1 EWAS (N=686)
$-\log_{10}(p)$
Chromosome

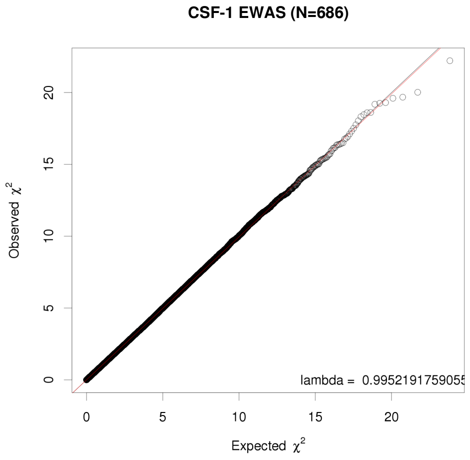
CSF-1 EWAS (N=686)
Observed χ^2
Expected χ^2
lambda = 0.9952191759055

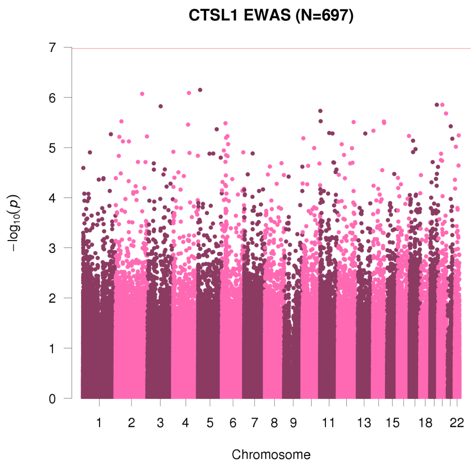
CTSL1 EWAS (N=697)
$-\log_{10}(p)$
Chromosome

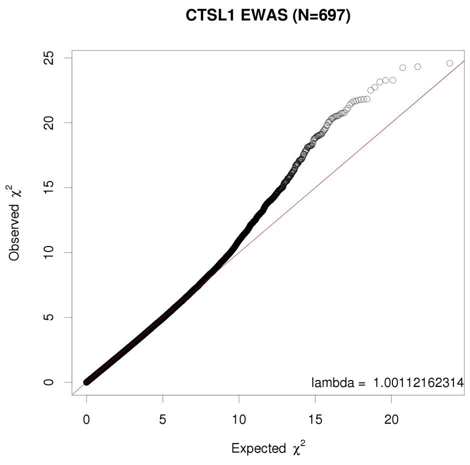
CTSL1 EWAS (N=697)
Observed χ^2
Expected χ^2
lambda = 1.00112162314

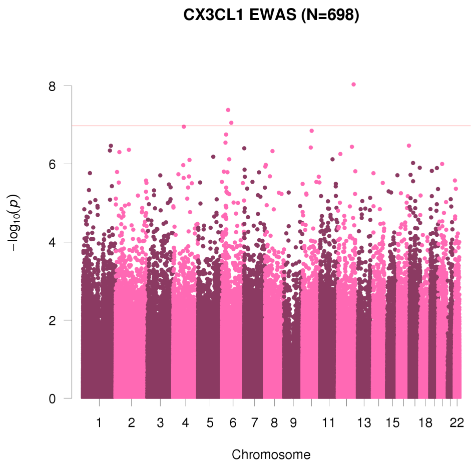
CX3CL1 EWAS (N=698)
$-\log_{10}(p)$
Chromosome
1 2 3 4 5 6 7 8 9 11 13 15 18 22

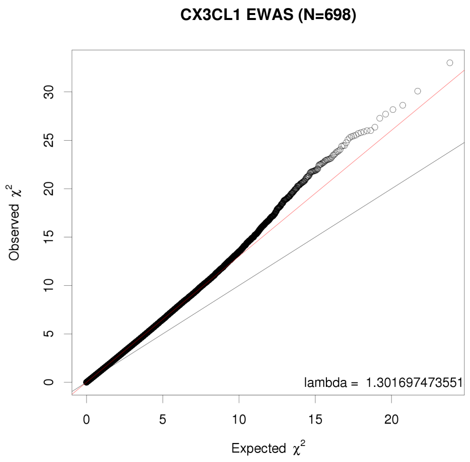
CX3CL1 EWAS (N=698)
Observed χ^2
Expected χ^2
lambda = 1.301697473551

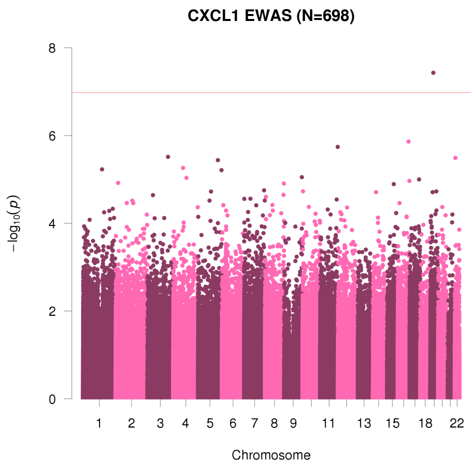
CXCL1 EWAS (N=698)
$-\log_{10}(p)$
Chromosome
1 2 3 4 5 6 7 8 9 11 13 15 18 22

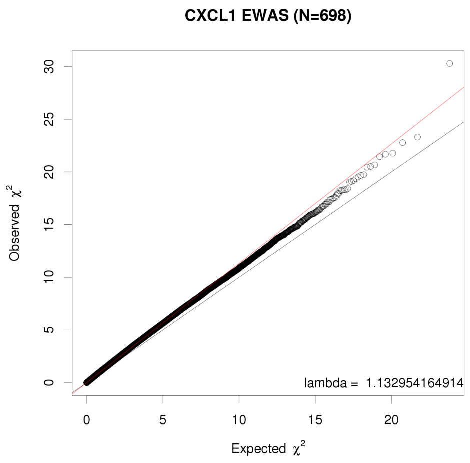
CXCL1 EWAS (N=698)
Observed χ^2
Expected χ^2
lambda = 1.132954164914

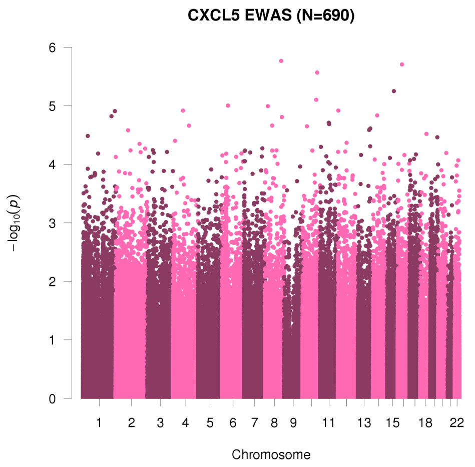
CXCL5 EWAS (N=690)
$-\log_{10}(p)$
Chromosome
1 2 3 4 5 6 7 8 9 11 13 15 18 22

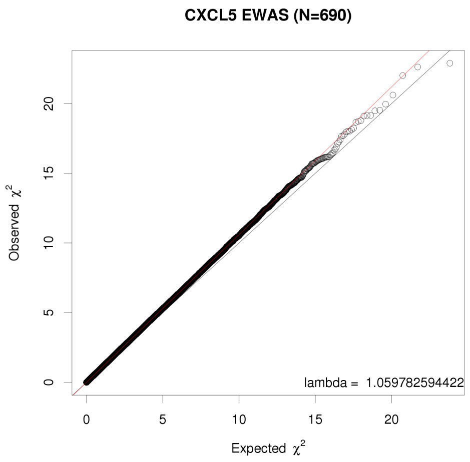
CXCL5 EWAS (N=690)
Observed χ^2
Expected χ^2
lambda = 1.059782594422

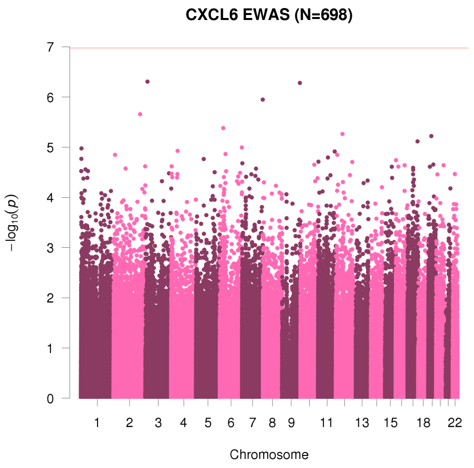
CXCL6 EWAS (N=698)
−log10(p)
Chromosome

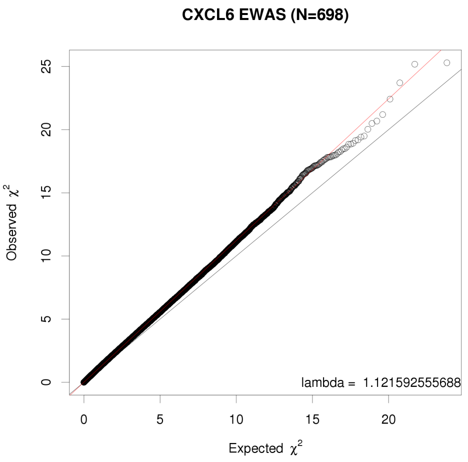
CXCL6 EWAS (N=698)
Observed χ2
Expected χ2
lambda = 1.121592555688

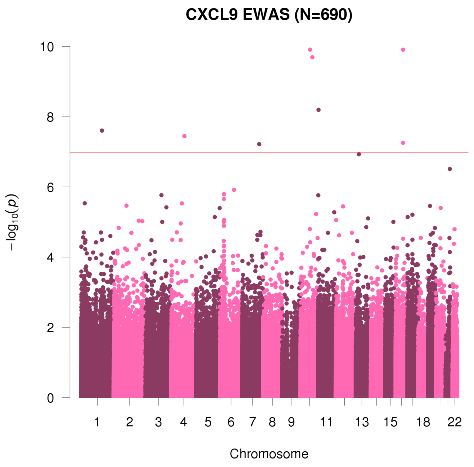
CXCL9 EWAS (N=690)
−log10(p)
Chromosome

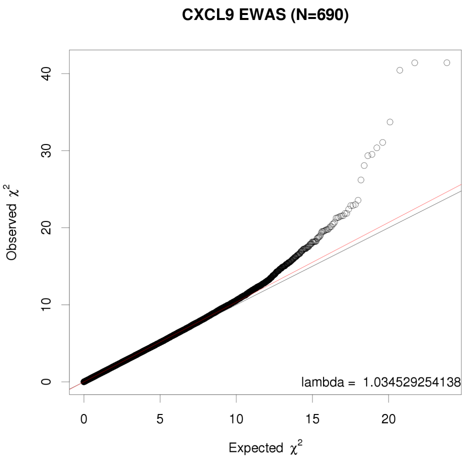
CXCL9 EWAS (N=690)
Observed χ2
Expected χ2
lambda = 1.034529254138

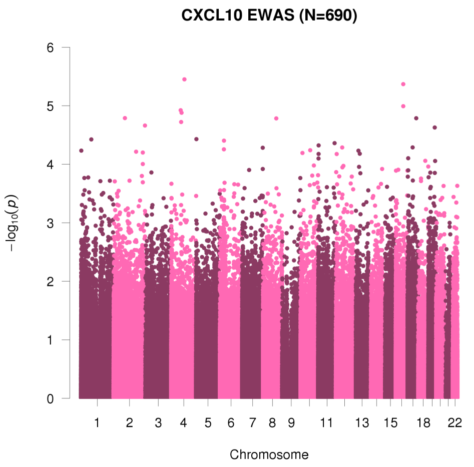
CXCL10 EWAS (N=690)
−log10(p)
Chromosome

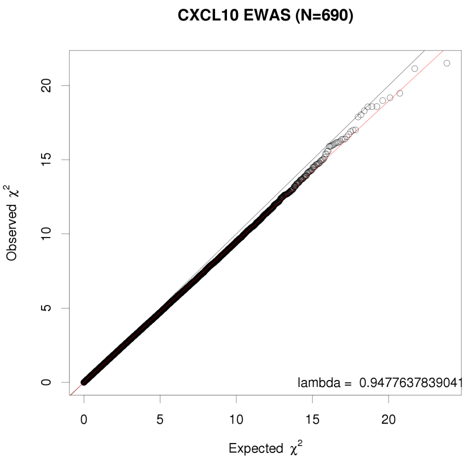
CXCL10 EWAS (N=690)
Observed χ2
Expected χ2
lambda = 0.9477637839041

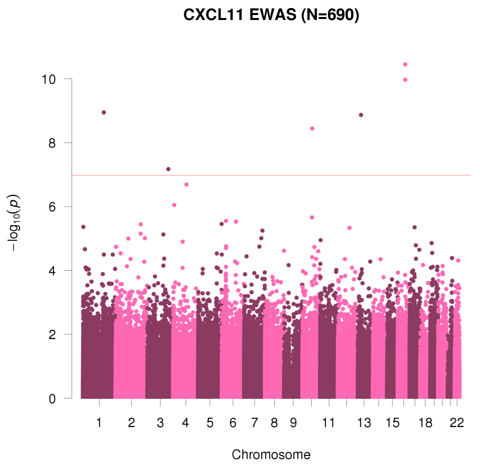
CXCL11 EWAS (N=690)
$-\log_{10}(p)$
Chromosome

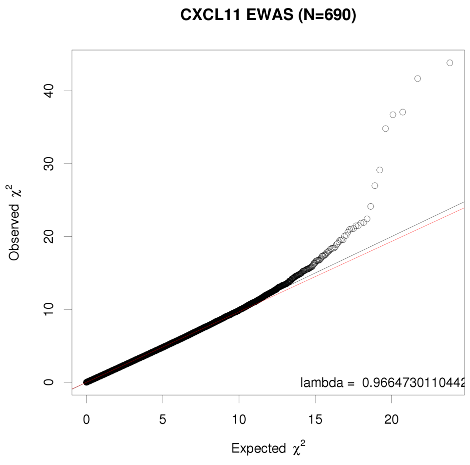
CXCL11 EWAS (N=690)
Observed χ^2
Expected χ^2
lambda = 0.9664730110442

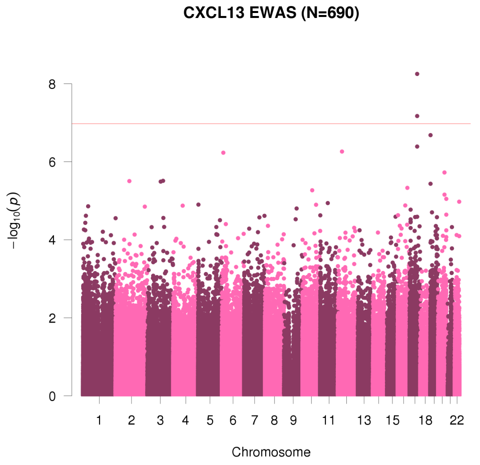
CXCL13 EWAS (N=690)
$-\log_{10}(p)$
Chromosome

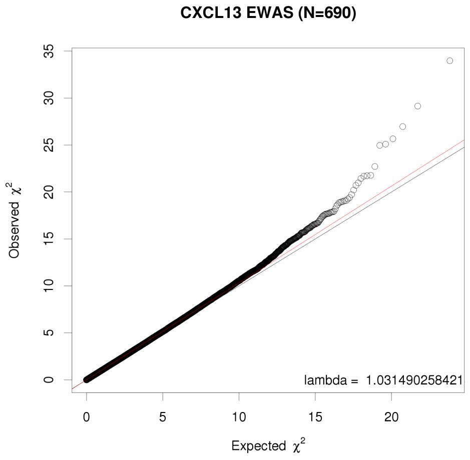
CXCL13 EWAS (N=690)
Observed χ^2
Expected χ^2
lambda = 1.031490258421

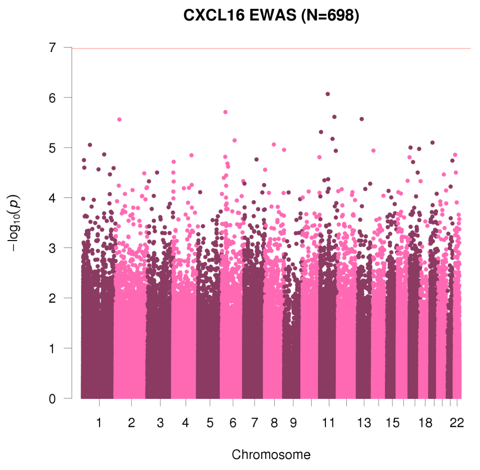
CXCL16 EWAS (N=698)
$-\log_{10}(p)$
Chromosome

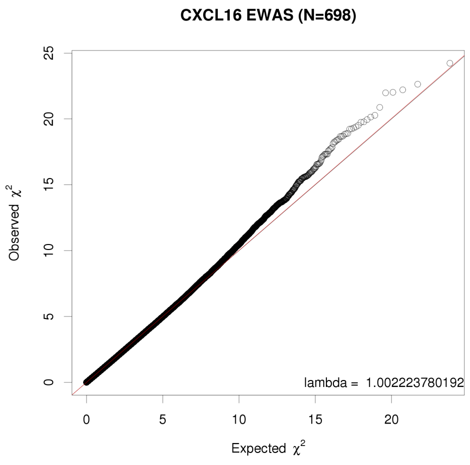
CXCL16 EWAS (N=698)
Observed χ^2
Expected χ^2
lambda = 1.002223780192

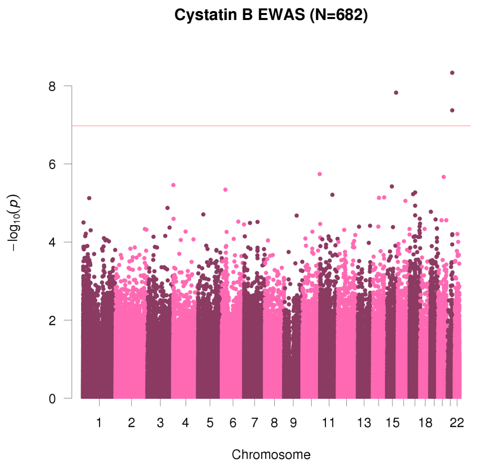
Cystatin B EWAS (N=682)
$-\log_{10}(p)$
Chromosome
1 2 3 4 5 6 7 8 9 11 13 15 18 22

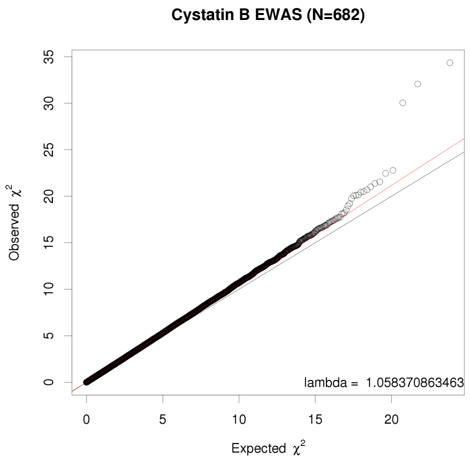
Cystatin B EWAS (N=682)
Observed χ^2
Expected χ^2
lambda = 1.058370863463

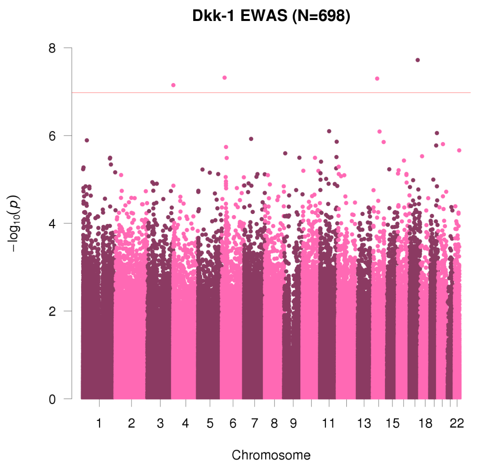
Dkk-1 EWAS (N=698)
$-\log_{10}(p)$
Chromosome
1 2 3 4 5 6 7 8 9 11 13 15 18 22

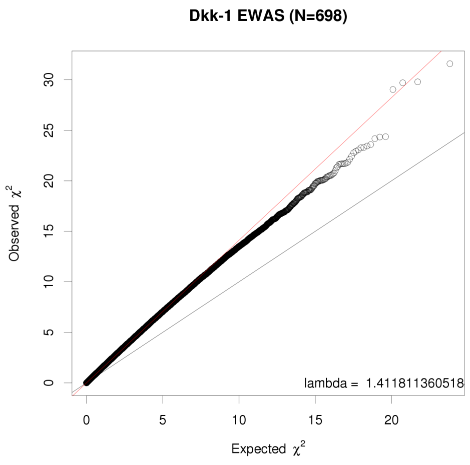
Dkk-1 EWAS (N=698)
Observed χ^2
Expected χ^2
lambda = 1.411811360518

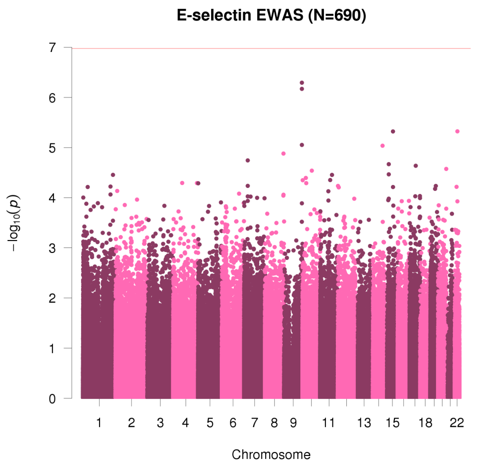
E-selectin EWAS (N=690)
$-\log_{10}(p)$
Chromosome
1 2 3 4 5 6 7 8 9 11 13 15 18 22

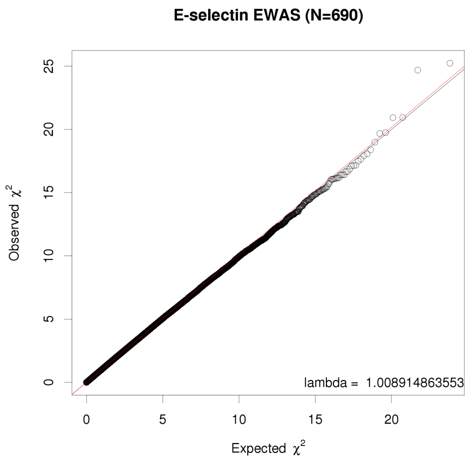
E-selectin EWAS (N=690)
Observed χ^2
Expected χ^2
lambda = 1.008914863553

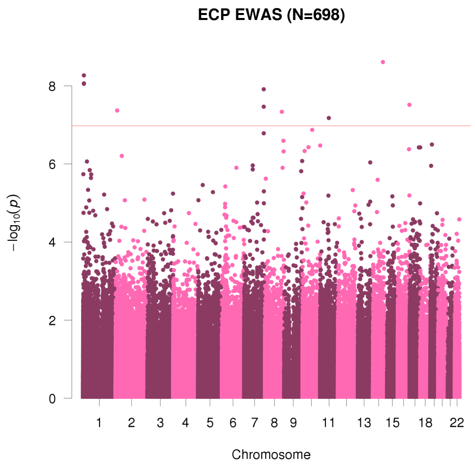
ECP EWAS (N=698)
$-\log_{10}(p)$
Chromosome

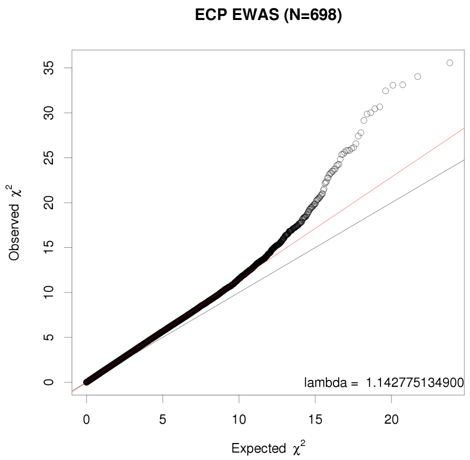
ECP EWAS (N=698)
Observed χ^2
Expected χ^2
lambda = 1.142775134900

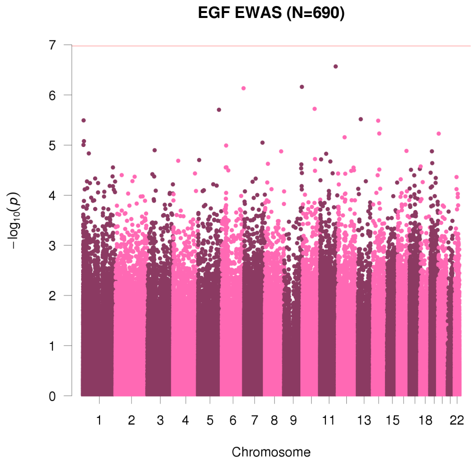
EGF EWAS (N=690)
$-\log_{10}(p)$
Chromosome

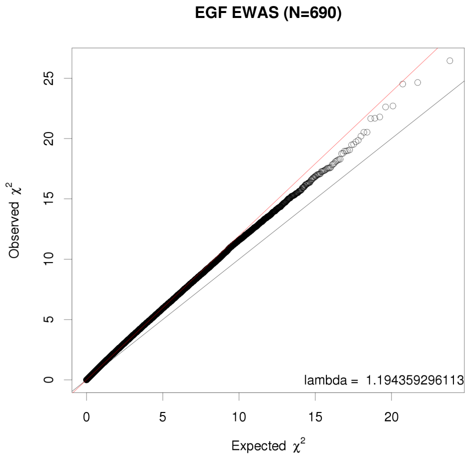
EGF EWAS (N=690)
Observed χ^2
Expected χ^2
lambda = 1.194359296113

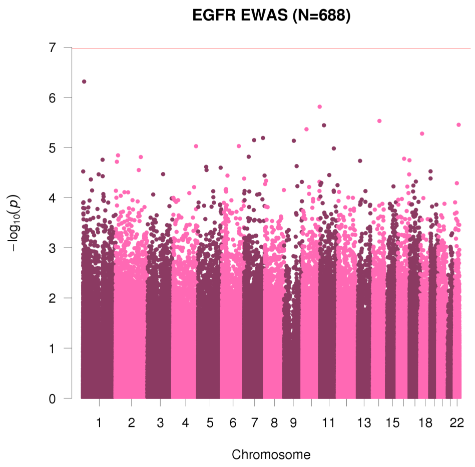
EGFR EWAS (N=688)
$-\log_{10}(p)$
Chromosome

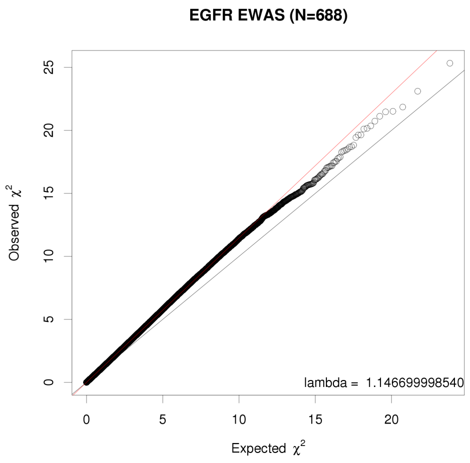
EGFR EWAS (N=688)
Observed χ^2
Expected χ^2
lambda = 1.146699998540

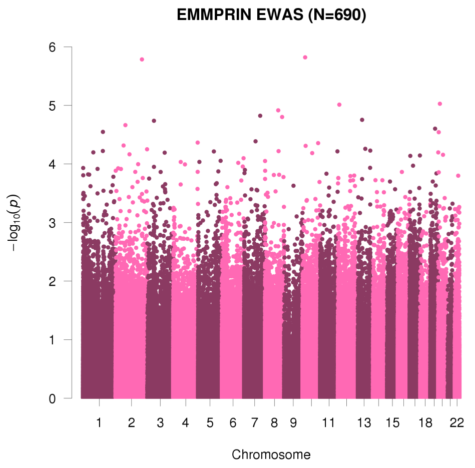
EMMPRIN EWAS (N=690)
$-\log_{10}(p)$
1 2 3 4 5 6 7 8 9 11 13 15 18 22
Chromosome

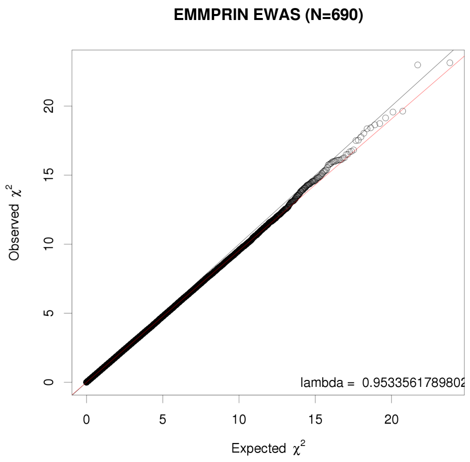
EMMPRIN EWAS (N=690)
Observed χ^2
Expected χ^2
lambda = 0.9533561789802

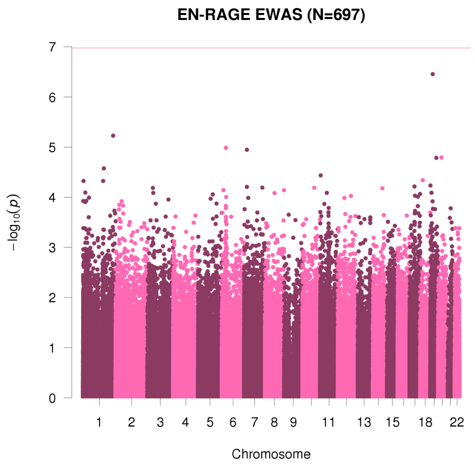
EN-RAGE EWAS (N=697)
$-\log_{10}(p)$
1 2 3 4 5 6 7 8 9 11 13 15 18 22
Chromosome

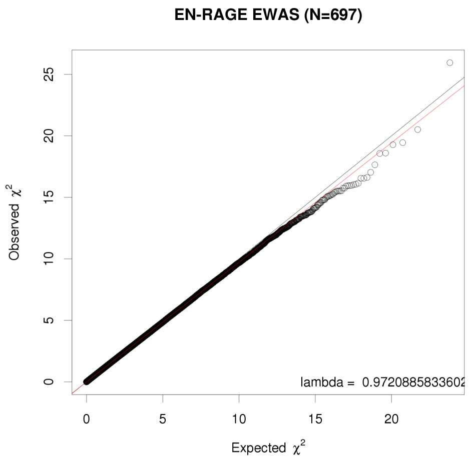
EN-RAGE EWAS (N=697)
Observed χ^2
Expected χ^2
lambda = 0.9720885833602

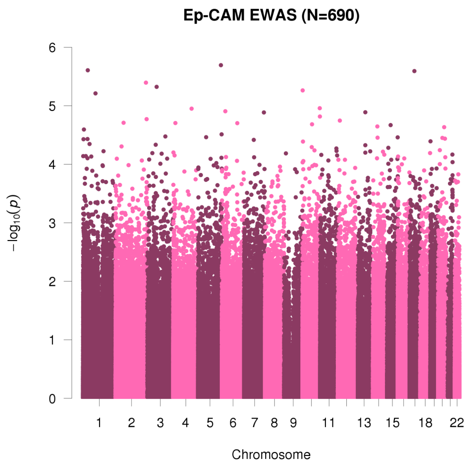
Ep-CAM EWAS (N=690)
$-\log_{10}(p)$
1 2 3 4 5 6 7 8 9 11 13 15 18 22
Chromosome

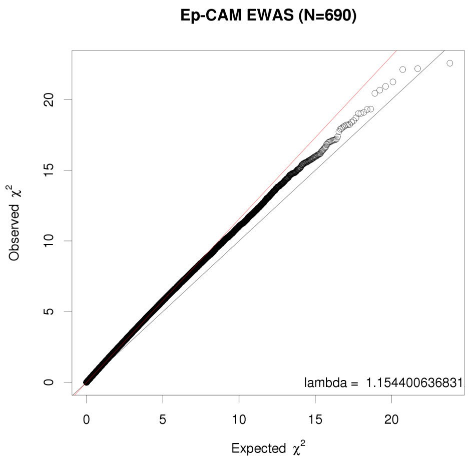
Ep-CAM EWAS (N=690)
Observed χ^2
Expected χ^2
lambda = 1.154400636831

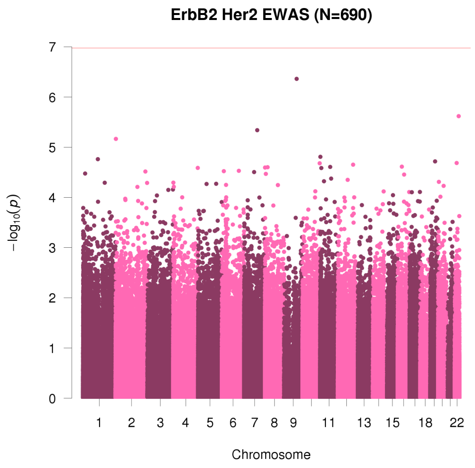
ErbB2 Her2 EWAS (N=690)
$-\log_{10}(p)$
Chromosome
1 2 3 4 5 6 7 8 9 11 13 15 18 22

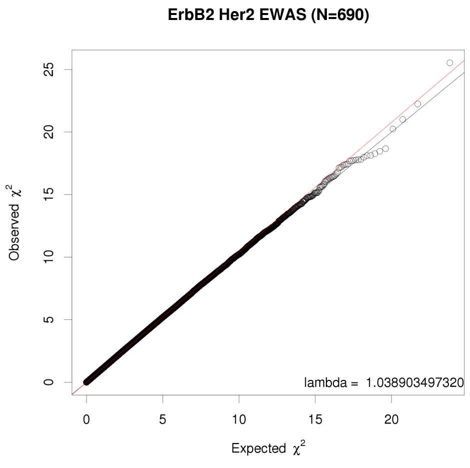
ErbB2 Her2 EWAS (N=690)
Observed χ^2
Expected χ^2
lambda = 1.038903497320

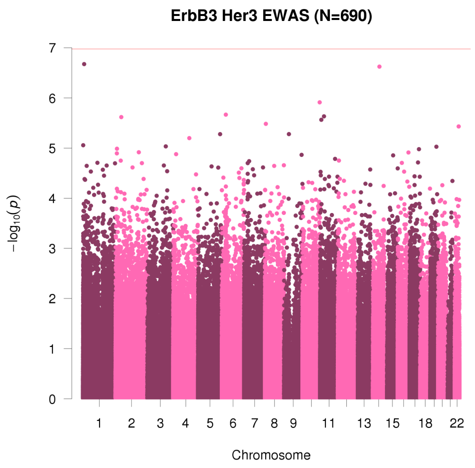
ErbB3 Her3 EWAS (N=690)
$-\log_{10}(p)$
Chromosome
1 2 3 4 5 6 7 8 9 11 13 15 18 22

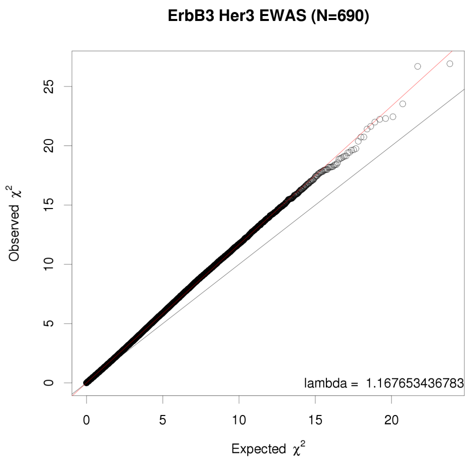
ErbB3 Her3 EWAS (N=690)
Observed χ^2
Expected χ^2
lambda = 1.167653436783

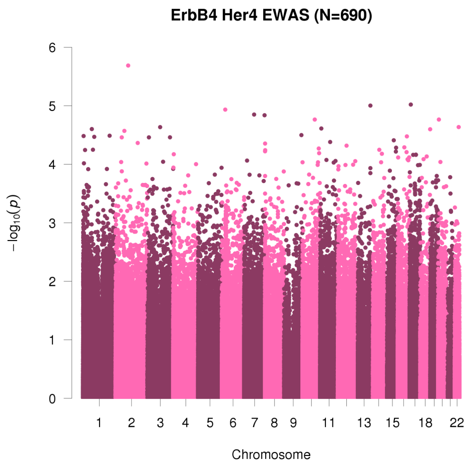
ErbB4 Her4 EWAS (N=690)
$-\log_{10}(p)$
Chromosome
1 2 3 4 5 6 7 8 9 11 13 15 18 22

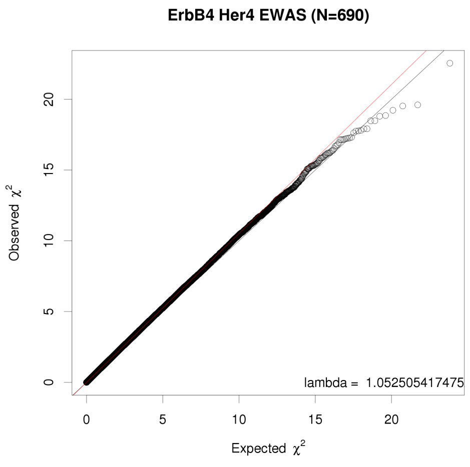
ErbB4 Her4 EWAS (N=690)
Observed χ^2
Expected χ^2
lambda = 1.052505417475

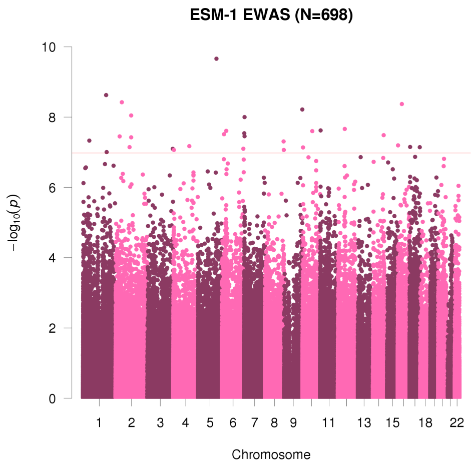
ESM-1 EWAS (N=698)
$-\log_{10}(p)$
0
2
4
6
8
10
1 2 3 4 5 6 7 8 9 11 13 15 18 22
Chromosome

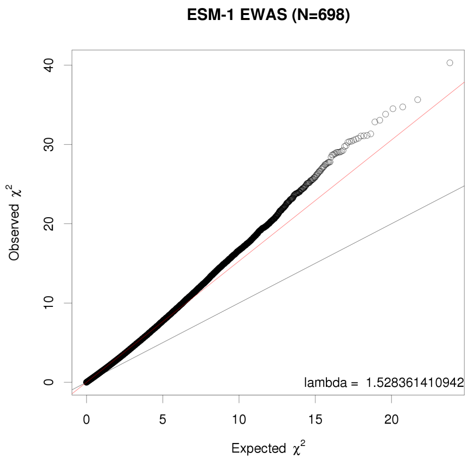
ESM-1 EWAS (N=698)
Observed χ^2
0
10
20
30
40
lambda = 1.528361410942
0 5 10 15 20
Expected χ^2

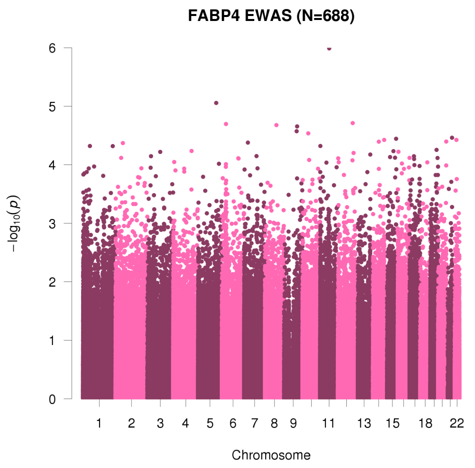
FABP4 EWAS (N=688)
$-\log_{10}(p)$
0
1
2
3
4
5
6
1 2 3 4 5 6 7 8 9 11 13 15 18 22
Chromosome

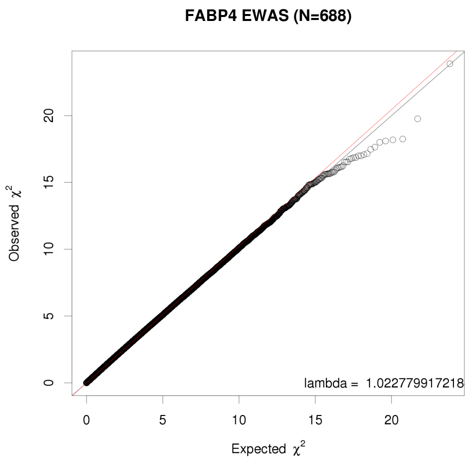
FABP4 EWAS (N=688)
Observed χ^2
0
5
10
15
20
lambda = 1.022779917218
0 5 10 15 20
Expected χ^2

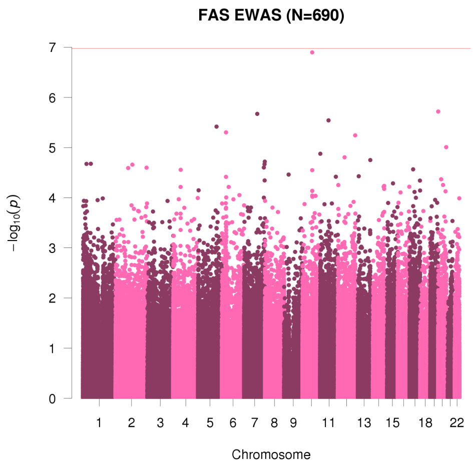
FAS EWAS (N=690)
$-\log_{10}(p)$
0
1
2
3
4
5
6
7
1 2 3 4 5 6 7 8 9 11 13 15 18 22
Chromosome

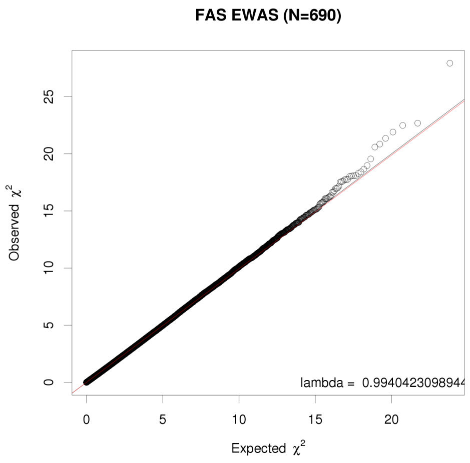
FAS EWAS (N=690)
Observed χ^2
0
5
10
15
20
25
lambda = 0.9940423098944
0 5 10 15 20
Expected χ^2

FGF-23 EWAS (N=698)

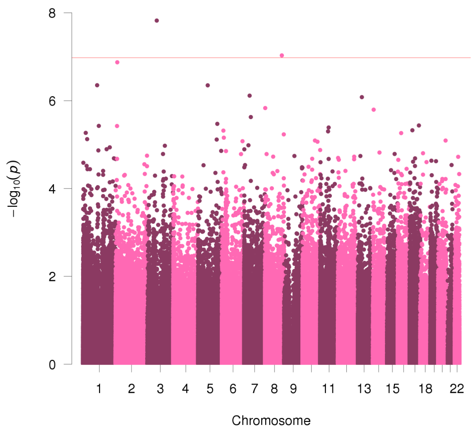

FGF-23 EWAS (N=698)

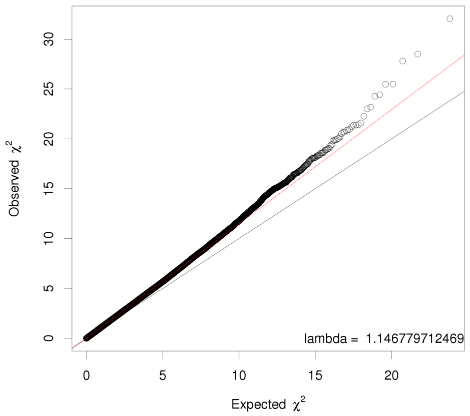

Flt3L EWAS (N=690)

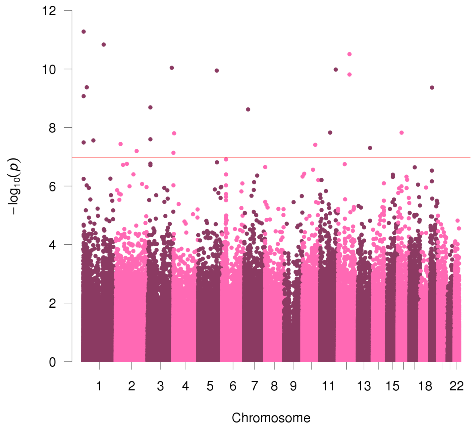

Flt3L EWAS (N=690)

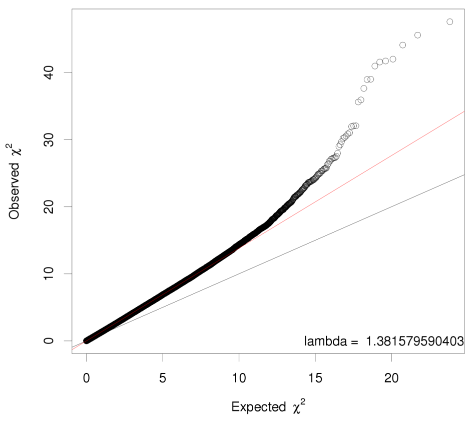

Follistatin EWAS (N=545)

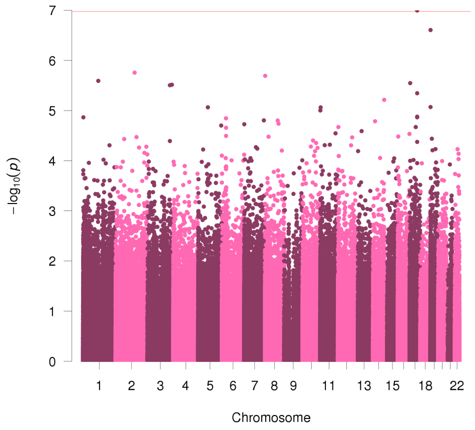

Follistatin EWAS (N=545)

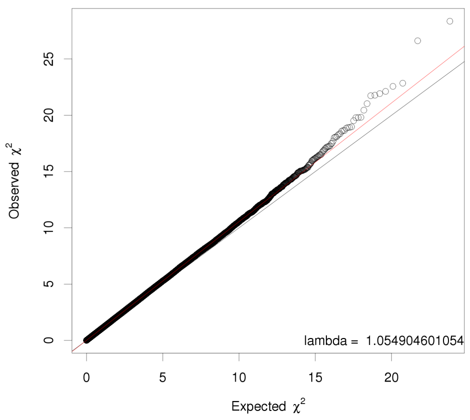

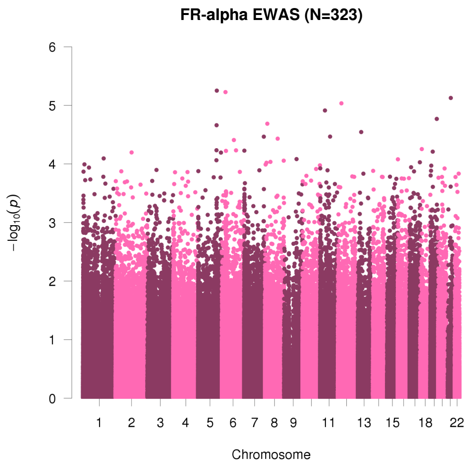
FR-alpha EWAS (N=323)
$-\log_{10}(p)$
Chromosome

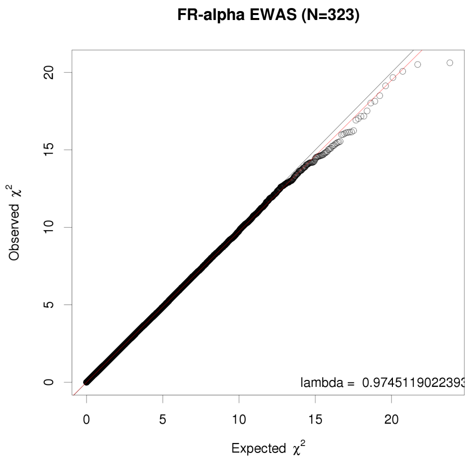
FR-alpha EWAS (N=323)
Observed χ^2
Expected χ^2
lambda = 0.9745119022393

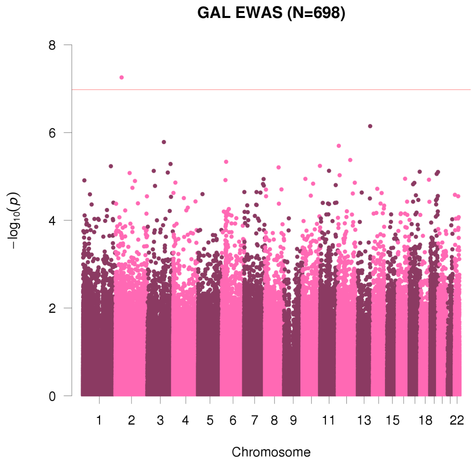
GAL EWAS (N=698)
$-\log_{10}(p)$
Chromosome

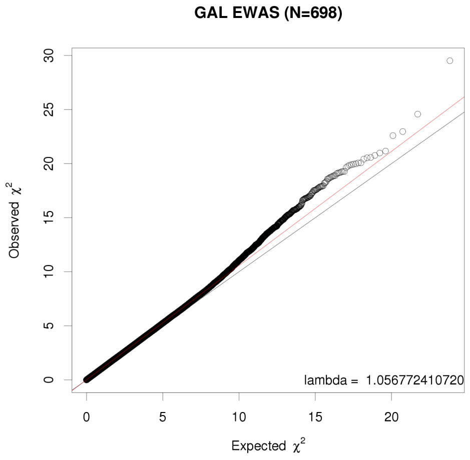
GAL EWAS (N=698)
Observed χ^2
Expected χ^2
lambda = 1.056772410720

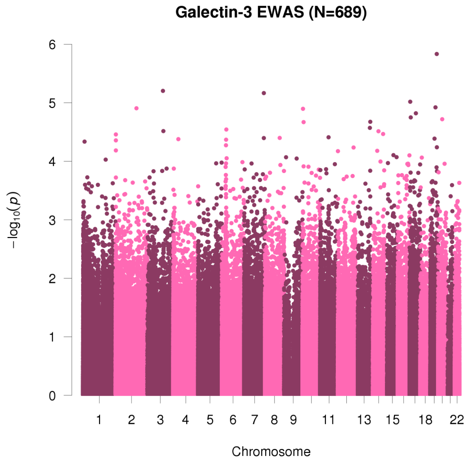
Galectin-3 EWAS (N=689)
$-\log_{10}(p)$
Chromosome

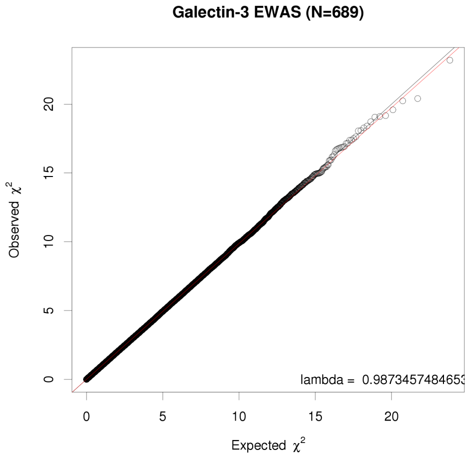
Galectin-3 EWAS (N=689)
Observed χ^2
Expected χ^2
lambda = 0.9873457484653

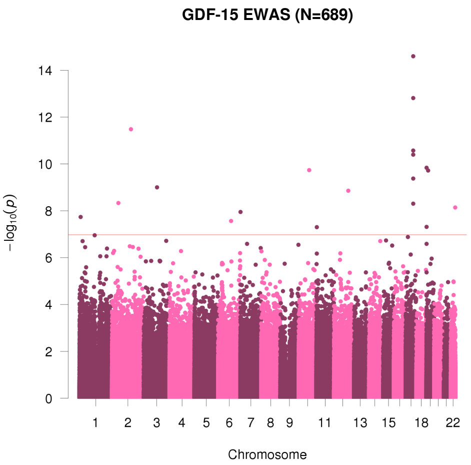
GDF-15 EWAS (N=689)
$-\log_{10}(p)$
Chromosome

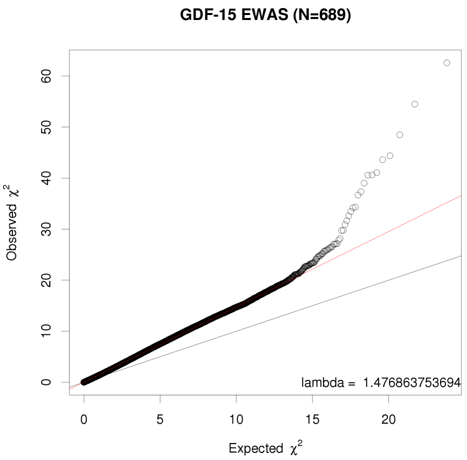
GDF-15 EWAS (N=689)
Observed χ^2
Expected χ^2
lambda = 1.476863753694

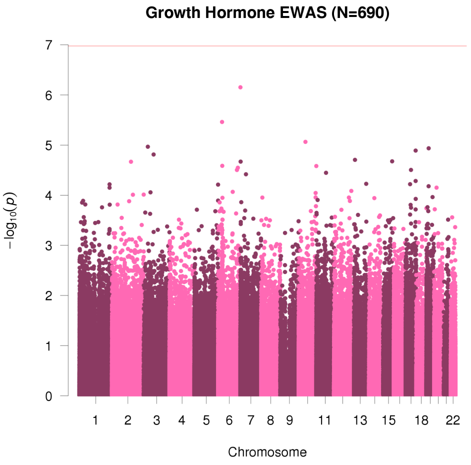
Growth Hormone EWAS (N=690)
$-\log_{10}(p)$
Chromosome

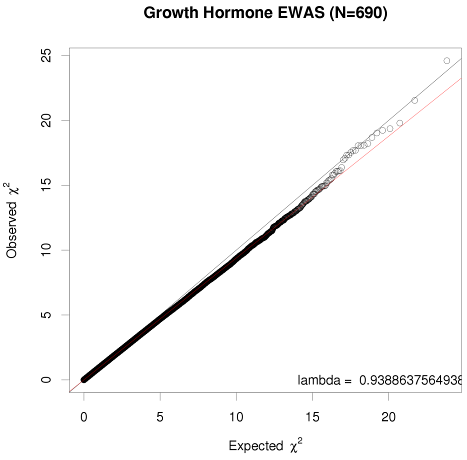
Growth Hormone EWAS (N=690)
Observed χ^2
Expected χ^2
lambda = 0.9388637564938

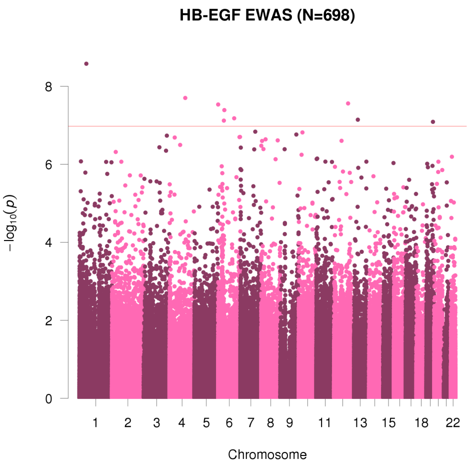
HB-EGF EWAS (N=698)
$-\log_{10}(p)$
Chromosome

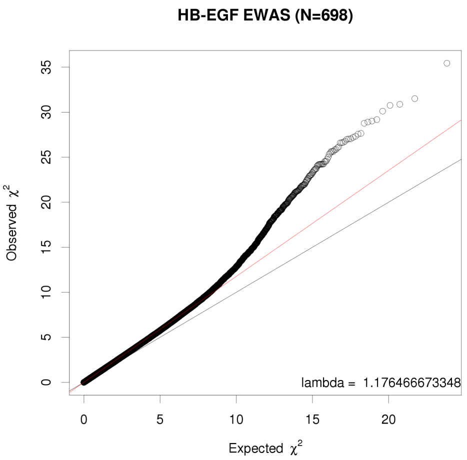
HB-EGF EWAS (N=698)
Observed χ^2
Expected χ^2
lambda = 1.176466673348

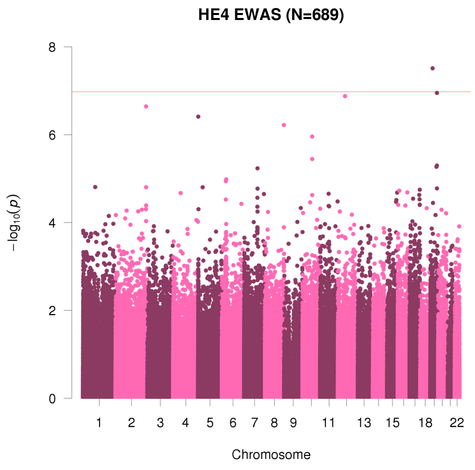
HE4 EWAS (N=689)
$-\log_{10}(p)$
0 2 4 6 8
1 2 3 4 5 6 7 8 9 11 13 15 18 22
Chromosome

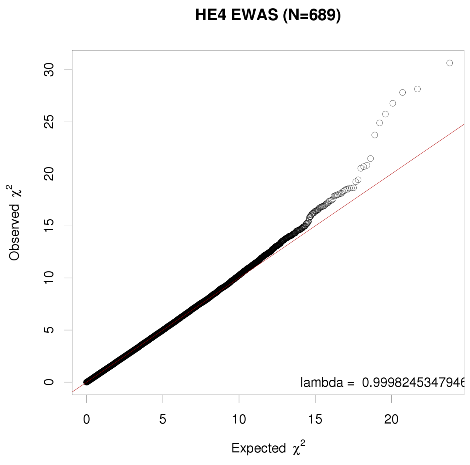
HE4 EWAS (N=689)
Observed χ^2
0 5 10 15 20 25 30
0 5 10 15 20
Expected χ^2
lambda = 0.9998245347946

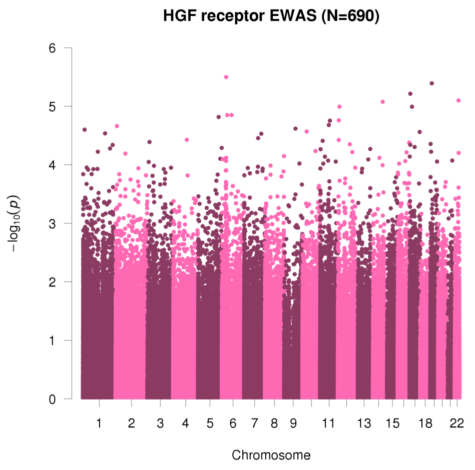
HGF receptor EWAS (N=690)
$-\log_{10}(p)$
0 1 2 3 4 5 6
1 2 3 4 5 6 7 8 9 11 13 15 18 22
Chromosome

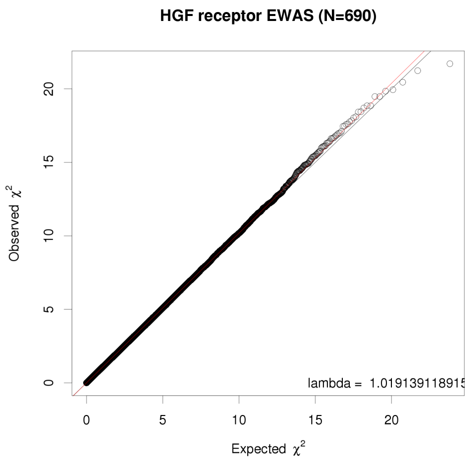
HGF receptor EWAS (N=690)
Observed χ^2
0 5 10 15 20
0 5 10 15 20
Expected χ^2
lambda = 1.019139118915

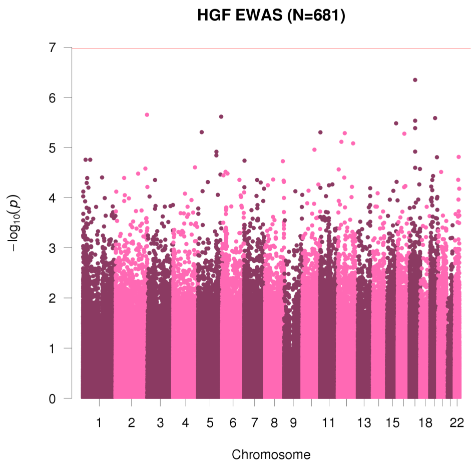
HGF EWAS (N=681)
$-\log_{10}(p)$
0 1 2 3 4 5 6 7
1 2 3 4 5 6 7 8 9 11 13 15 18 22
Chromosome

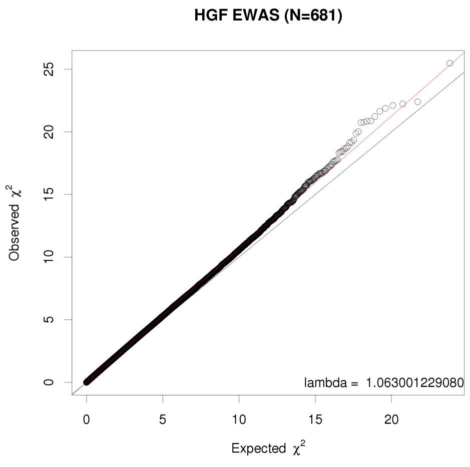
HGF EWAS (N=681)
Observed χ^2
0 5 10 15 20 25
0 5 10 15 20
Expected χ^2
lambda = 1.063001229080

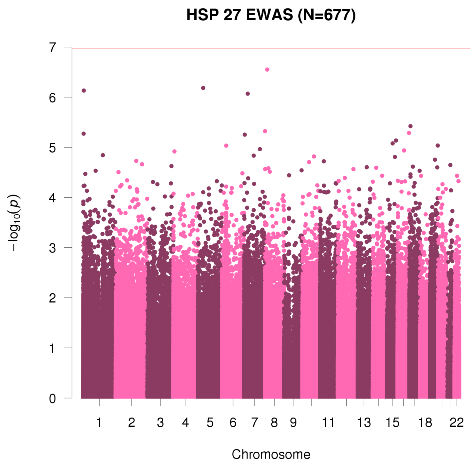
HSP 27 EWAS (N=677)
$-\log_{10}(p)$
Chromosome

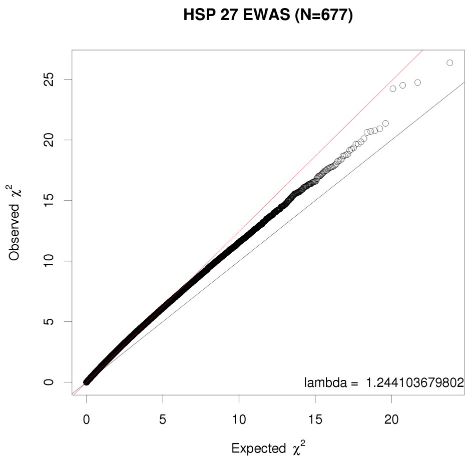
HSP 27 EWAS (N=677)
Observed χ^2
Expected χ^2
lambda = 1.244103679802

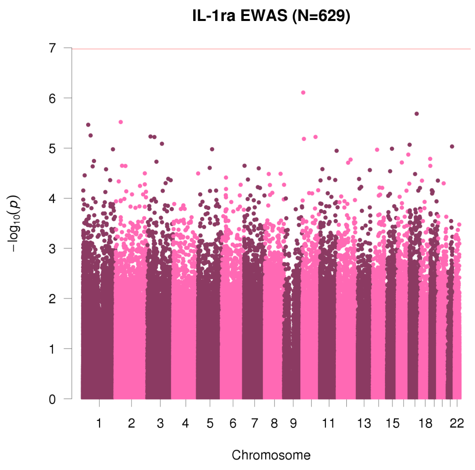
IL-1ra EWAS (N=629)
$-\log_{10}(p)$
Chromosome

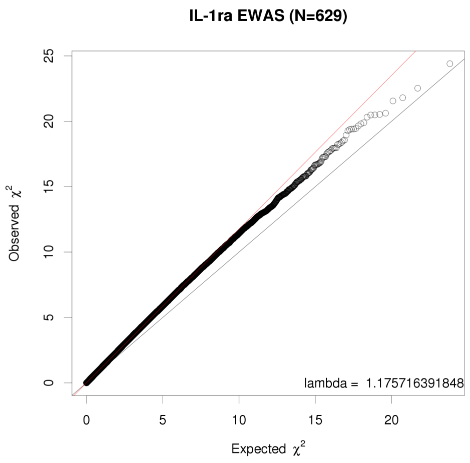
IL-1ra EWAS (N=629)
Observed χ^2
Expected χ^2
lambda = 1.175716391848

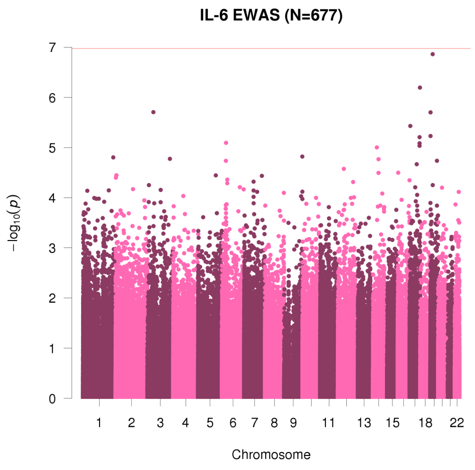
IL-6 EWAS (N=677)
$-\log_{10}(p)$
Chromosome

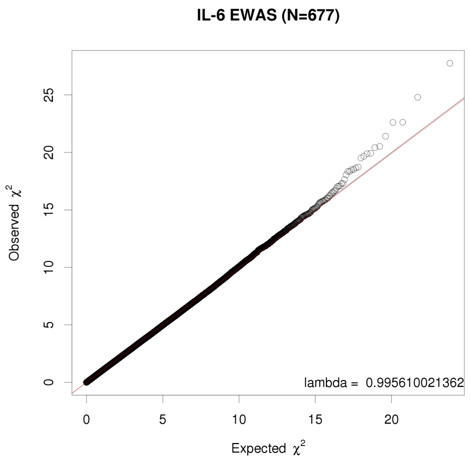
IL-6 EWAS (N=677)
Observed χ^2
Expected χ^2
lambda = 0.995610021362

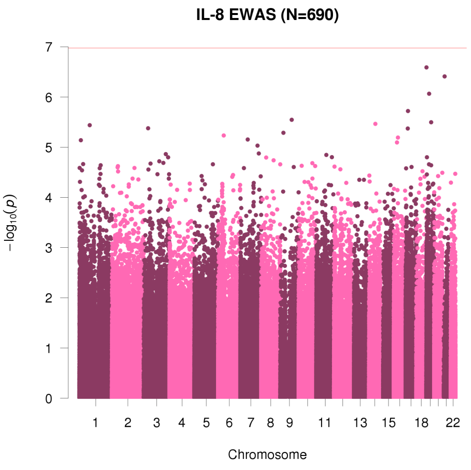
IL-8 EWAS (N=690)
−log10(p)
Chromosome

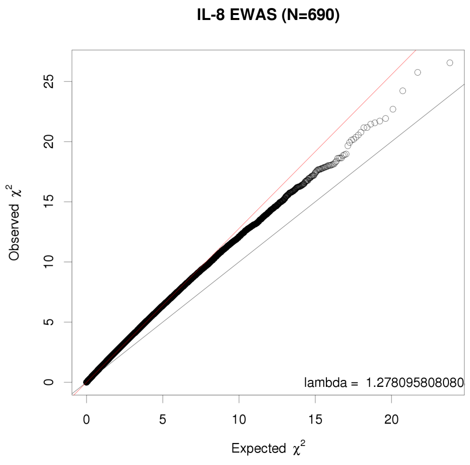
IL-8 EWAS (N=690)
Observed χ2
Expected χ2
lambda = 1.278095808080

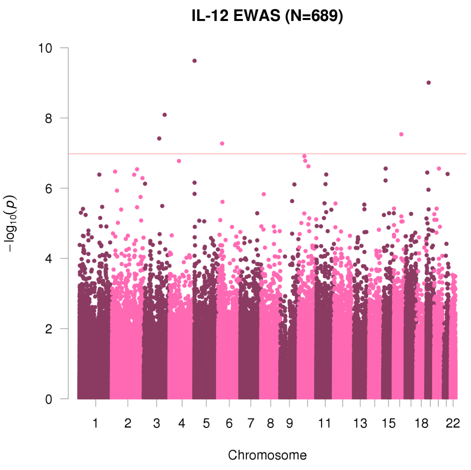
IL-12 EWAS (N=689)
−log10(p)
Chromosome

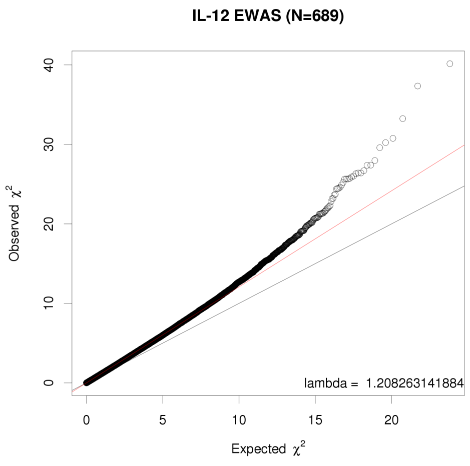
IL-12 EWAS (N=689)
Observed χ2
Expected χ2
lambda = 1.208263141884

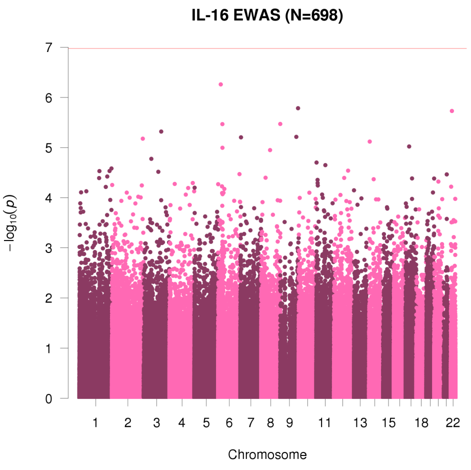
IL-16 EWAS (N=698)
−log10(p)
Chromosome

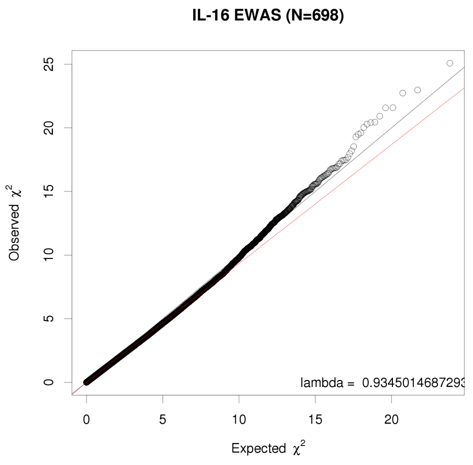
IL-16 EWAS (N=698)
Observed χ2
Expected χ2
lambda = 0.934501468729

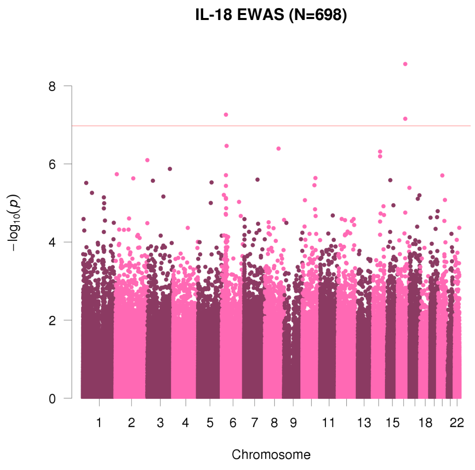
IL-18 EWAS (N=698)
−log10(p)
Chromosome

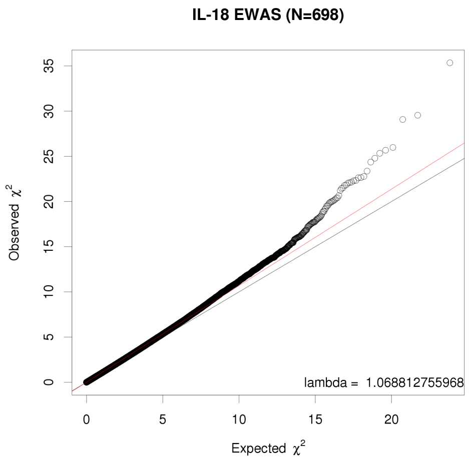
IL-18 EWAS (N=698)
Observed χ2
Expected χ2
lambda = 1.068812755968

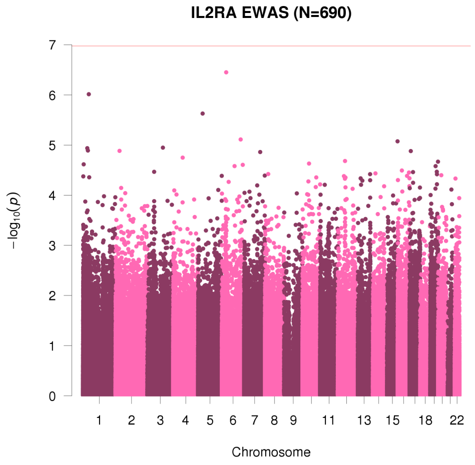
IL2RA EWAS (N=690)
−log10(p)
Chromosome

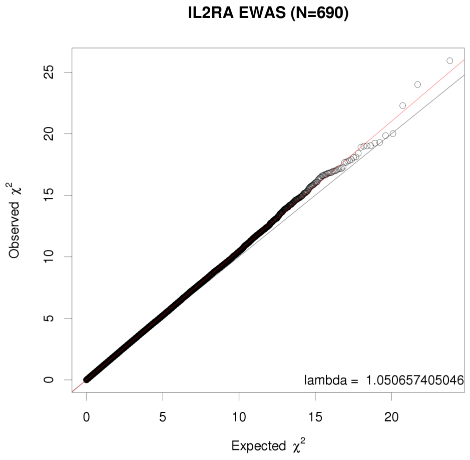
IL2RA EWAS (N=690)
Observed χ2
Expected χ2
lambda = 1.050657405046

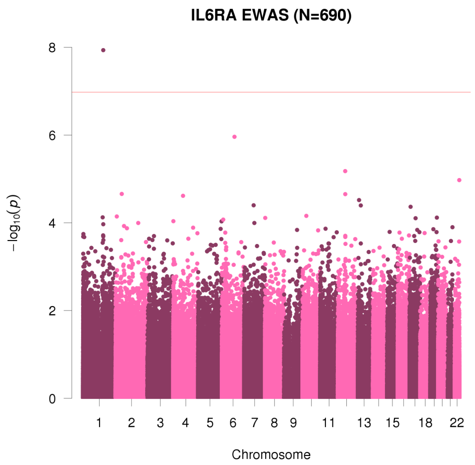
IL6RA EWAS (N=690)
−log10(p)
Chromosome

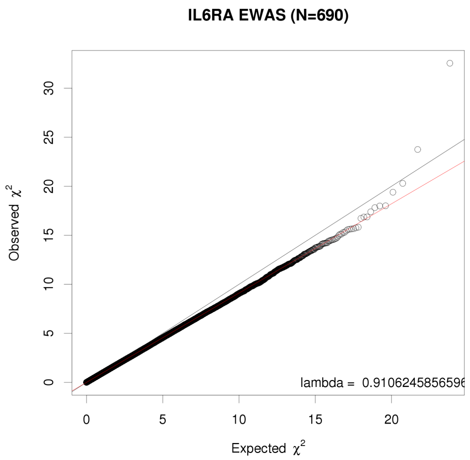
IL6RA EWAS (N=690)
Observed χ2
Expected χ2
lambda = 0.910624585659

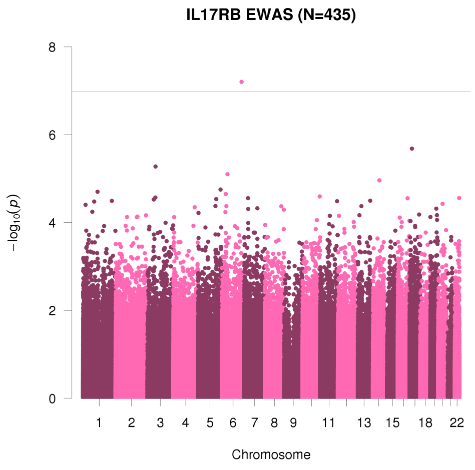
IL17RB EWAS (N=435)
$-\log_{10}(p)$
Chromosome

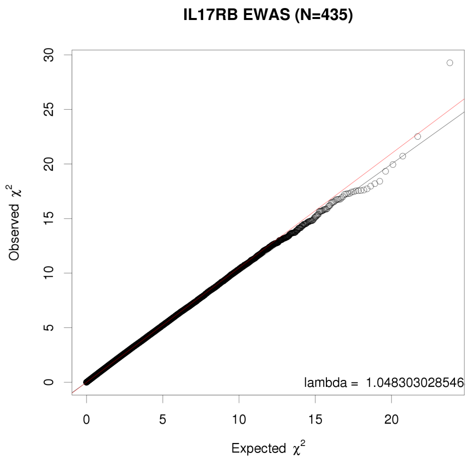
IL17RB EWAS (N=435)
Observed χ^2
Expected χ^2
lambda = 1.048303028546

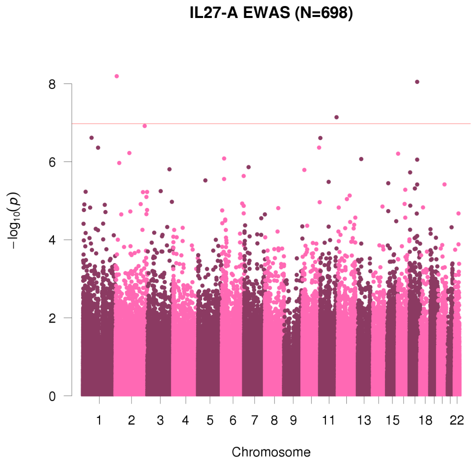
IL27-A EWAS (N=698)
$-\log_{10}(p)$
Chromosome

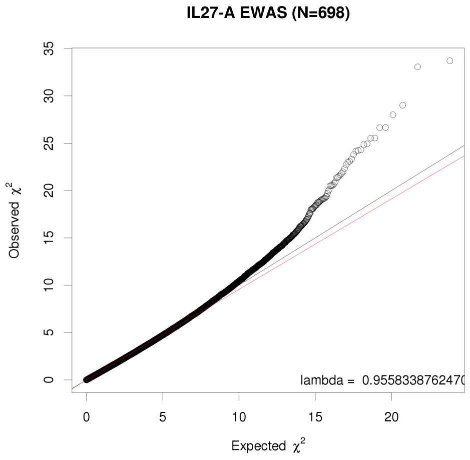
IL27-A EWAS (N=698)
Observed χ^2
Expected χ^2
lambda = 0.955833876247

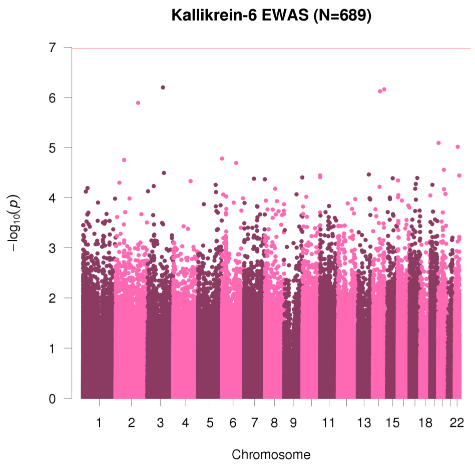
Kallikrein-6 EWAS (N=689)
$-\log_{10}(p)$
Chromosome

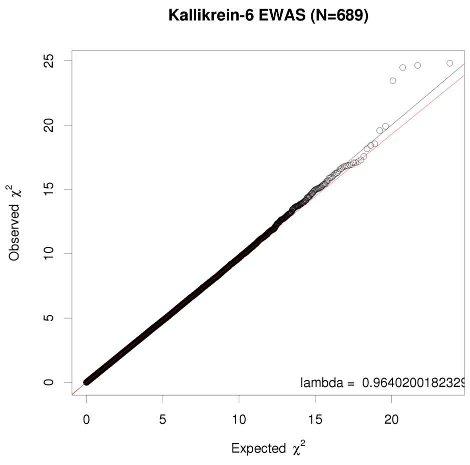
Kallikrein-6 EWAS (N=689)
Observed χ^2
Expected χ^2
lambda = 0.964020018232

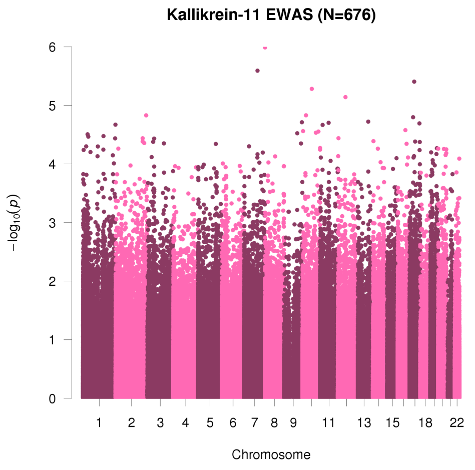
Kallikrein-11 EWAS (N=676)
$-\log_{10}(p)$
Chromosome

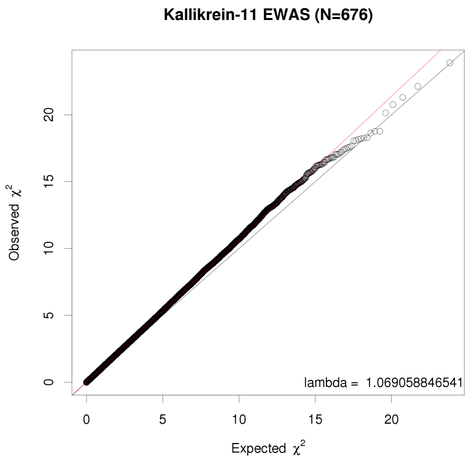
Kallikrein-11 EWAS (N=676)
Observed χ^2
Expected χ^2
lambda = 1.069058846541

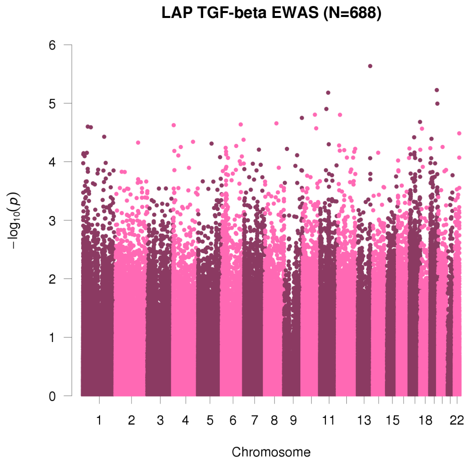
LAP TGF-beta EWAS (N=688)
$-\log_{10}(p)$
Chromosome

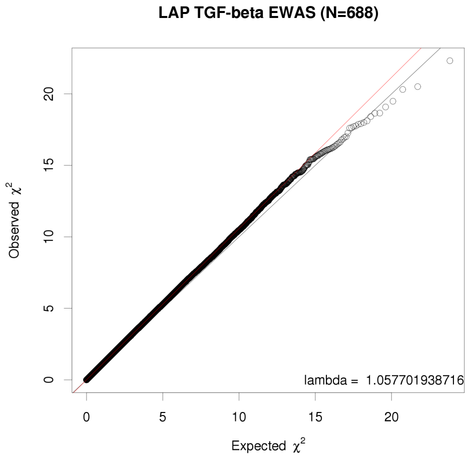
LAP TGF-beta EWAS (N=688)
Observed χ^2
Expected χ^2
lambda = 1.057701938716

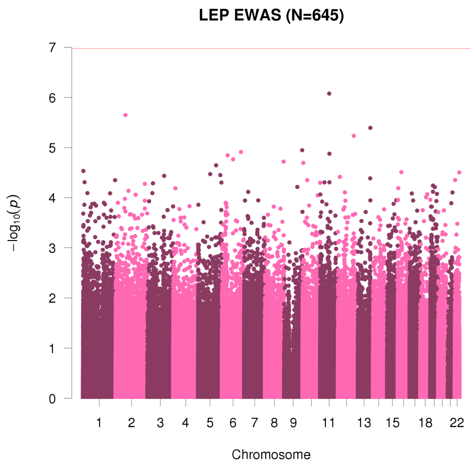
LEP EWAS (N=645)
$-\log_{10}(p)$
Chromosome

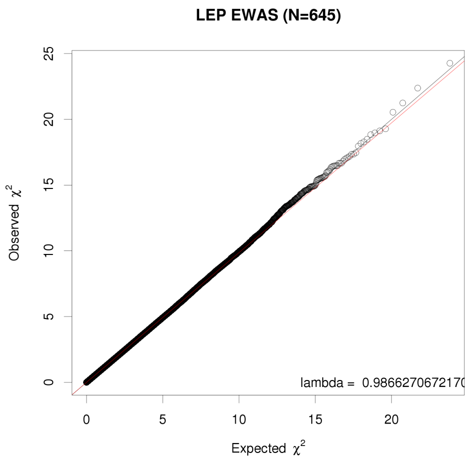
LEP EWAS (N=645)
Observed χ^2
Expected χ^2
lambda = 0.986627067217

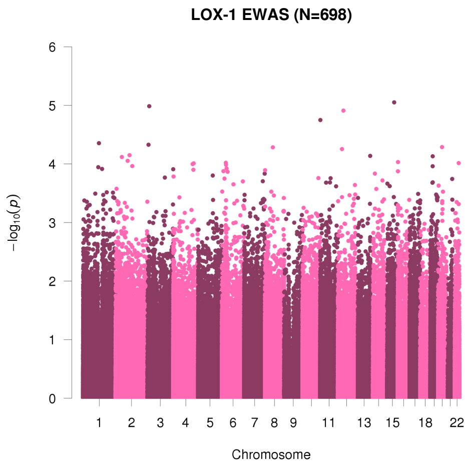
LOX-1 EWAS (N=698)
$-\log_{10}(p)$
Chromosome
1 2 3 4 5 6 7 8 9 11 13 15 18 22

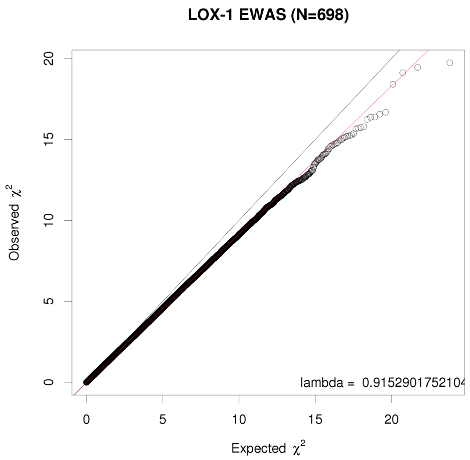
LOX-1 EWAS (N=698)
Observed χ^2
Expected χ^2
lambda = 0.9152901752104

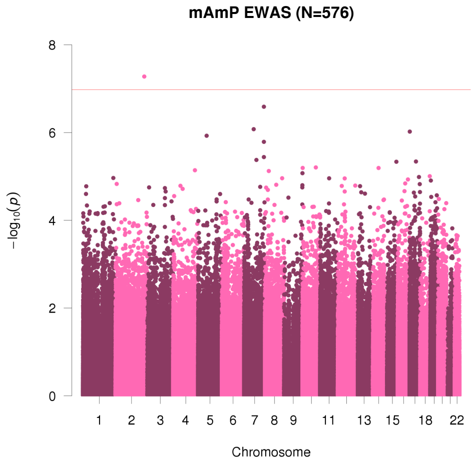
mAmP EWAS (N=576)
$-\log_{10}(p)$
Chromosome
1 2 3 4 5 6 7 8 9 11 13 15 18 22

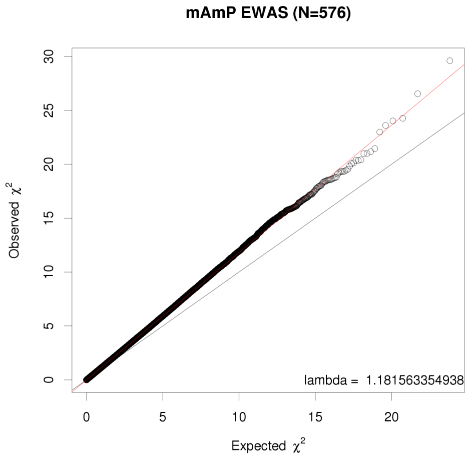
mAmP EWAS (N=576)
Observed χ^2
Expected χ^2
lambda = 1.181563354938

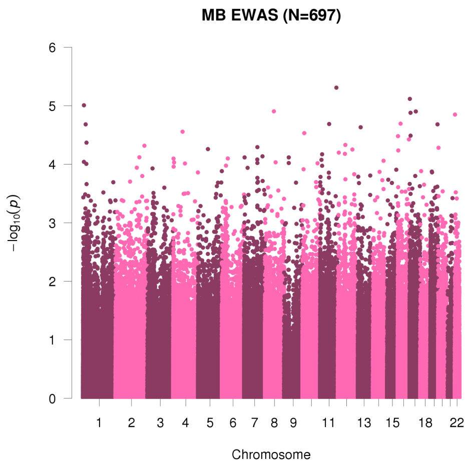
MB EWAS (N=697)
$-\log_{10}(p)$
Chromosome
1 2 3 4 5 6 7 8 9 11 13 15 18 22

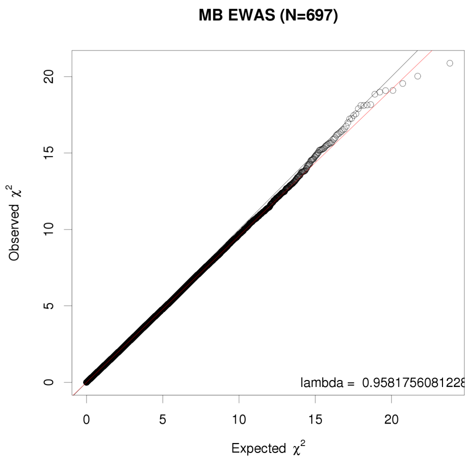
MB EWAS (N=697)
Observed χ^2
Expected χ^2
lambda = 0.958175608122

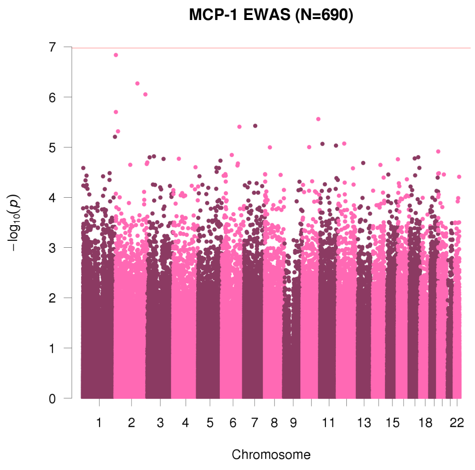
MCP-1 EWAS (N=690)
$-\log_{10}(p)$
Chromosome

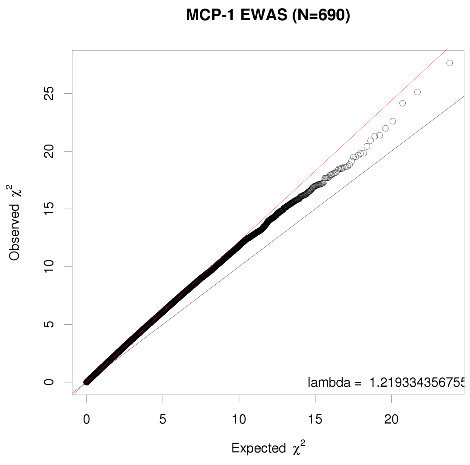
MCP-1 EWAS (N=690)
Observed χ^2
Expected χ^2
lambda = 1.21933435675

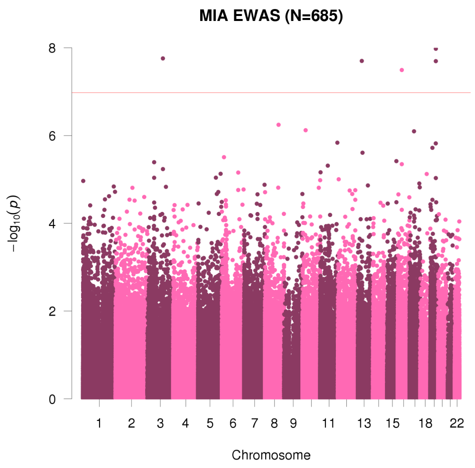
MIA EWAS (N=685)
$-\log_{10}(p)$
Chromosome

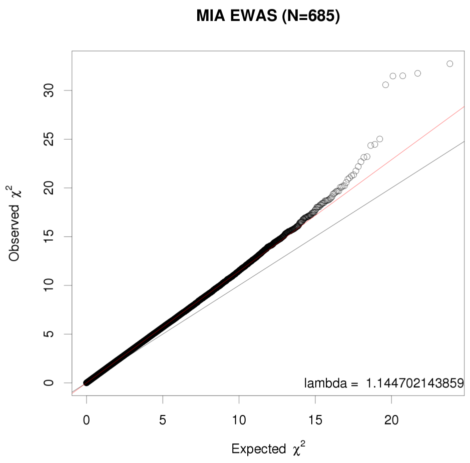
MIA EWAS (N=685)
Observed χ^2
Expected χ^2
lambda = 1.144702143859

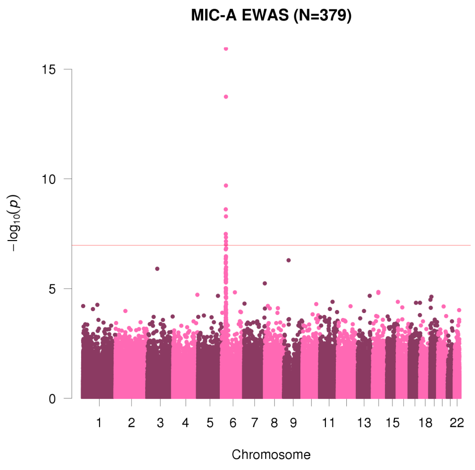
MIC-A EWAS (N=379)
$-\log_{10}(p)$
Chromosome

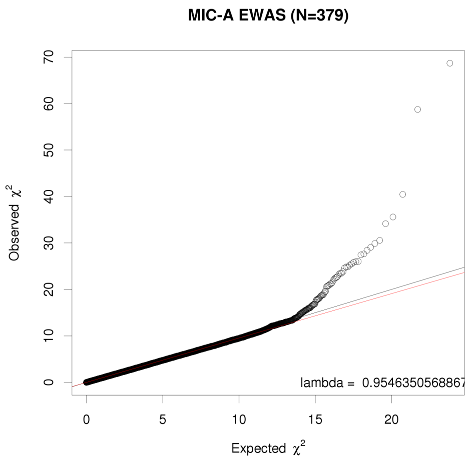
MIC-A EWAS (N=379)
Observed χ^2
Expected χ^2
lambda = 0.954635056886

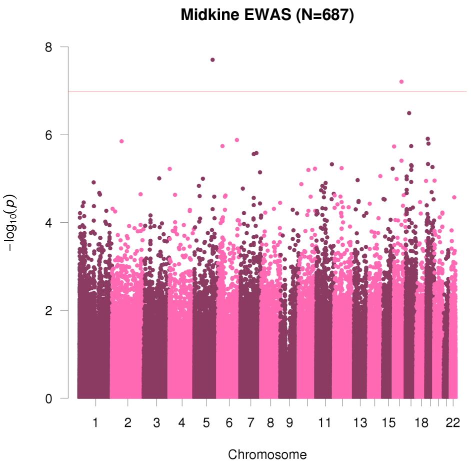
Midkine EWAS (N=687)
$-\log_{10}(p)$
Chromosome

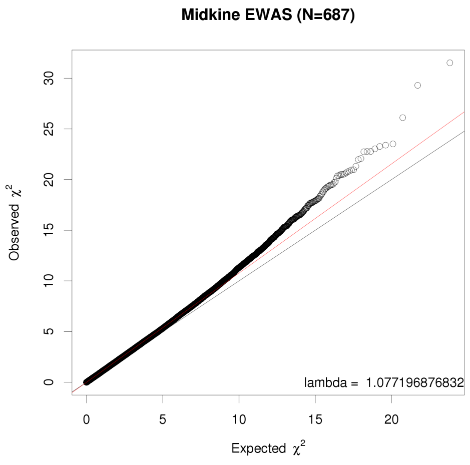
Midkine EWAS (N=687)
Observed χ^2
Expected χ^2
lambda = 1.077196876832

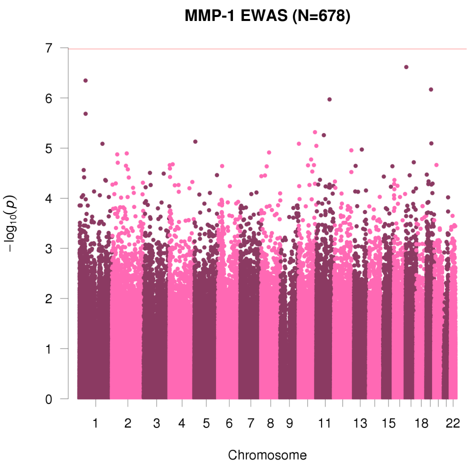
MMP-1 EWAS (N=678)
$-\log_{10}(p)$
Chromosome

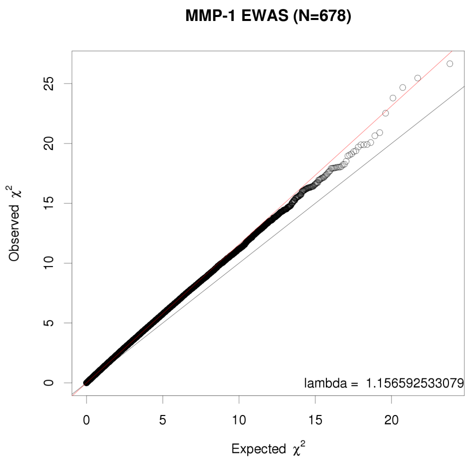
MMP-1 EWAS (N=678)
Observed χ^2
Expected χ^2
lambda = 1.156592533079

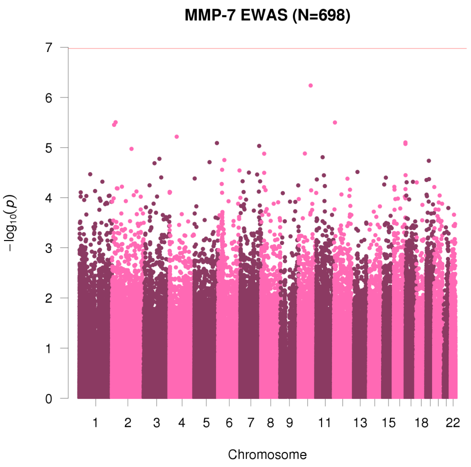
MMP-7 EWAS (N=698)
$-\log_{10}(p)$
Chromosome

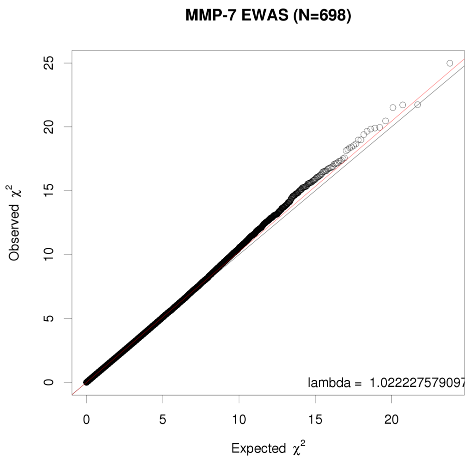
MMP-7 EWAS (N=698)
Observed χ^2
Expected χ^2
lambda = 1.022227579097

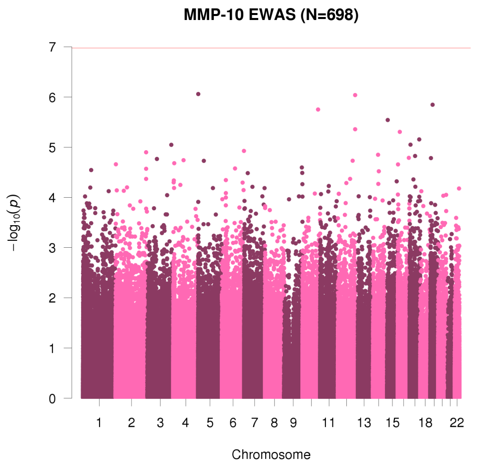
MMP-10 EWAS (N=698)
−log10(p)
0
1
2
3
4
5
6
7
1 2 3 4 5 6 7 8 9 11 13 15 18 22
Chromosome

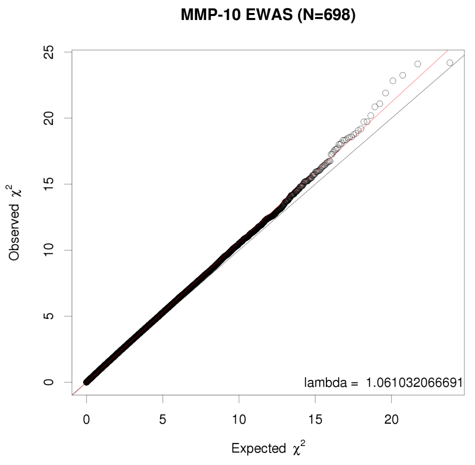
MMP-10 EWAS (N=698)
Observed χ2
0
5
10
15
20
25
lambda = 1.061032066691
0 5 10 15 20
Expected χ2

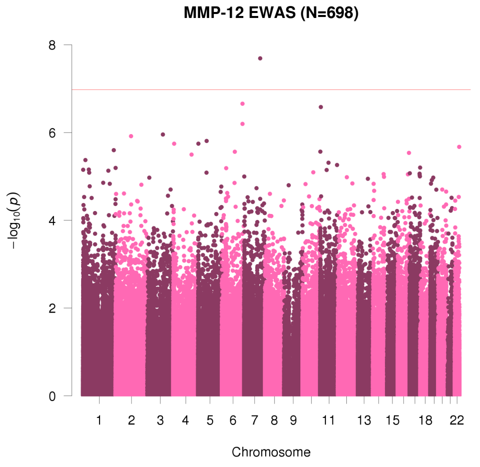
MMP-12 EWAS (N=698)
−log10(p)
0
2
4
6
8
1 2 3 4 5 6 7 8 9 11 13 15 18 22
Chromosome

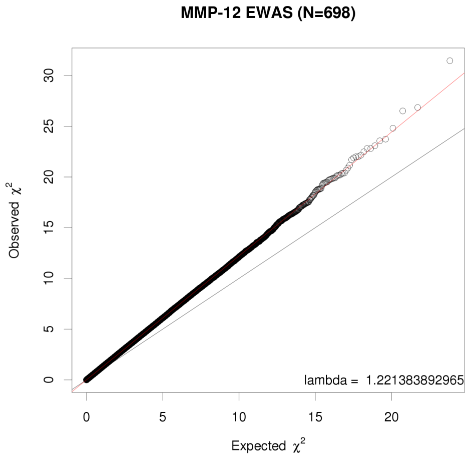
MMP-12 EWAS (N=698)
Observed χ2
0
5
10
15
20
25
30
lambda = 1.221383892965
0 5 10 15 20
Expected χ2

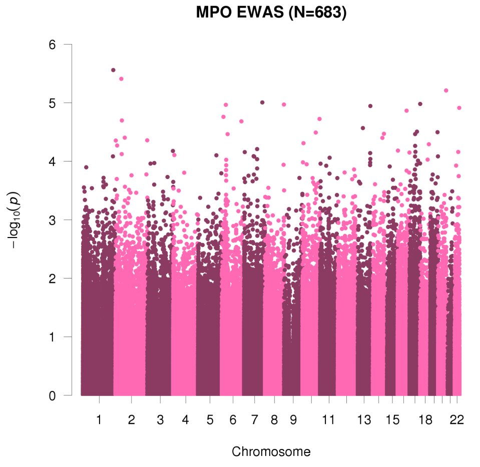
MPO EWAS (N=683)
−log10(p)
0
1
2
3
4
5
6
1 2 3 4 5 6 7 8 9 11 13 15 18 22
Chromosome

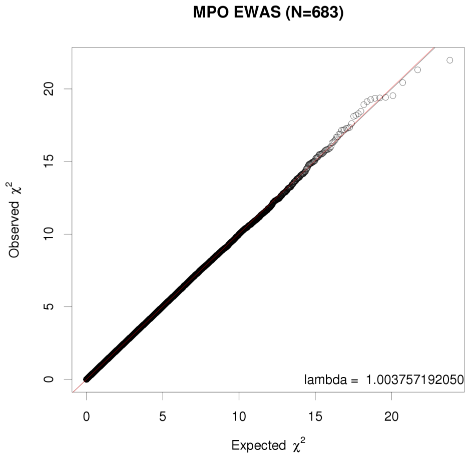
MPO EWAS (N=683)
Observed χ2
0
5
10
15
20
lambda = 1.003757192050
0 5 10 15 20
Expected χ2

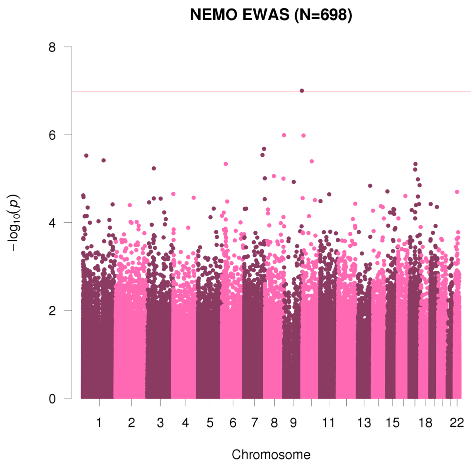
NEMO EWAS (N=698)
$-\log_{10}(p)$
Chromosome
1 2 3 4 5 6 7 8 9 11 13 15 18 22

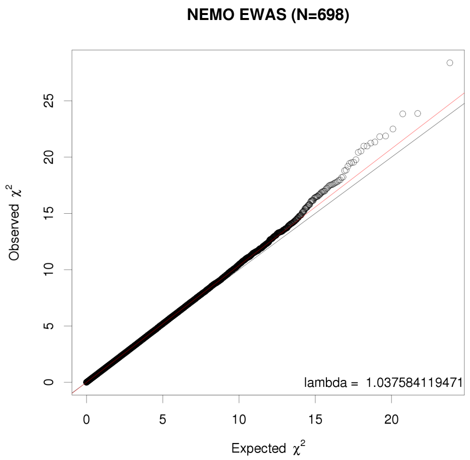
NEMO EWAS (N=698)
Observed χ^2
Expected χ^2
lambda = 1.037584119471

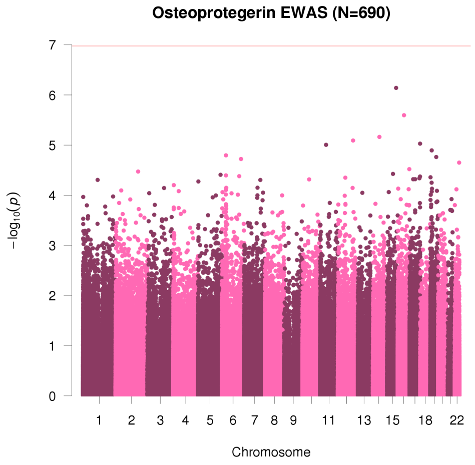
Osteoprotegerin EWAS (N=690)
$-\log_{10}(p)$
Chromosome
1 2 3 4 5 6 7 8 9 11 13 15 18 22

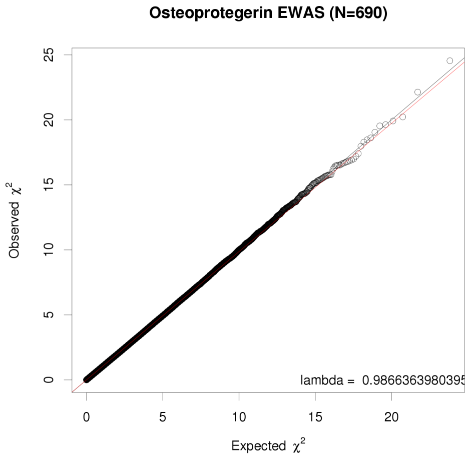
Osteoprotegerin EWAS (N=690)
Observed χ^2
Expected χ^2
lambda = 0.986636398039

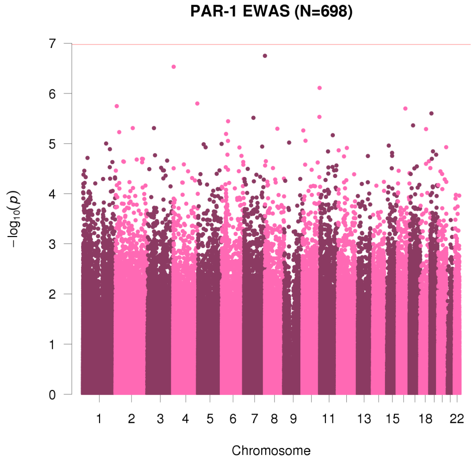
PAR-1 EWAS (N=698)
$-\log_{10}(p)$
Chromosome
1 2 3 4 5 6 7 8 9 11 13 15 18 22

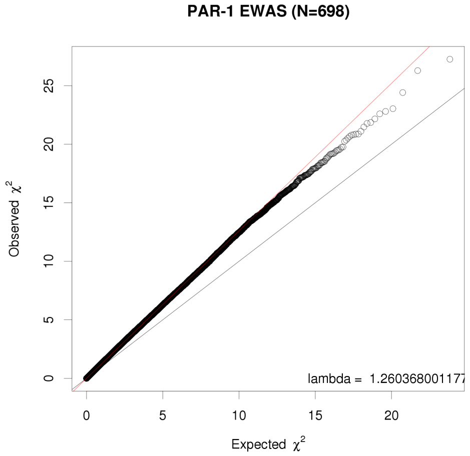
PAR-1 EWAS (N=698)
Observed χ^2
Expected χ^2
lambda = 1.26036800117

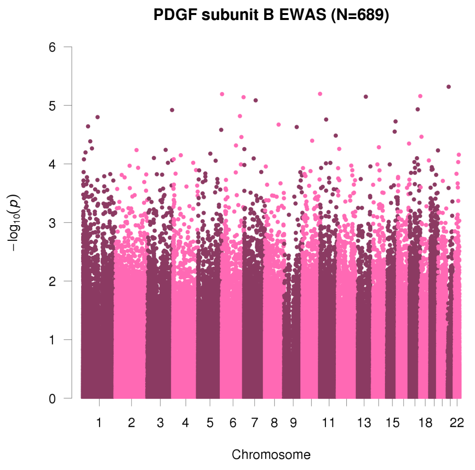
PDGF subunit B EWAS (N=689)
$-\log_{10}(p)$
Chromosome

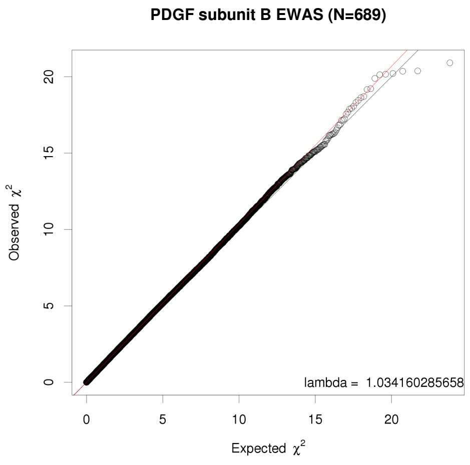
PDGF subunit B EWAS (N=689)
Observed χ^2
Expected χ^2
lambda = 1.034160285658

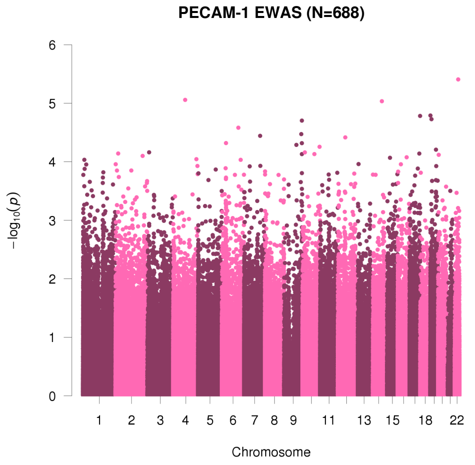
PECAM-1 EWAS (N=688)
$-\log_{10}(p)$
Chromosome

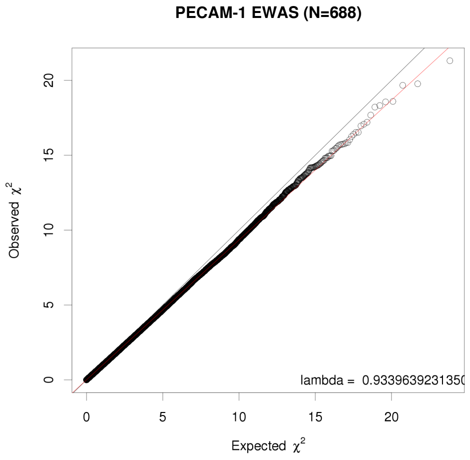
PECAM-1 EWAS (N=688)
Observed χ^2
Expected χ^2
lambda = 0.9339639231350

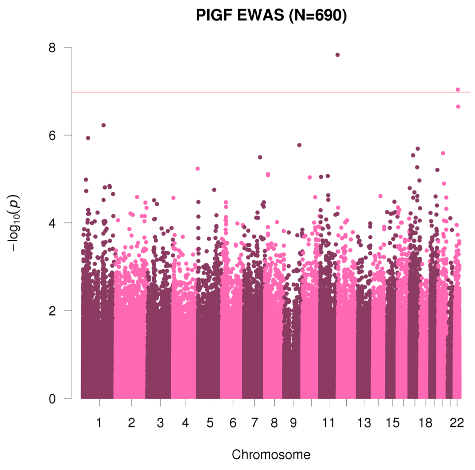
PIGF EWAS (N=690)
$-\log_{10}(p)$
Chromosome

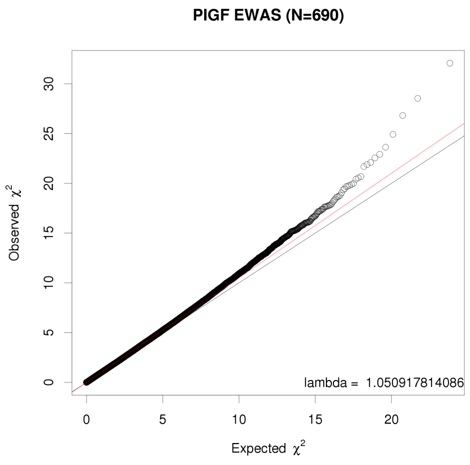
PIGF EWAS (N=690)
Observed χ^2
Expected χ^2
lambda = 1.050917814086

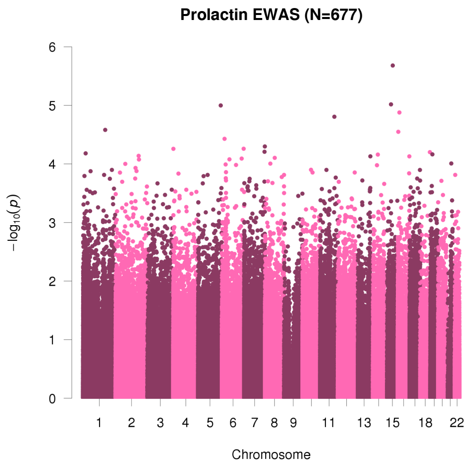
Prolactin EWAS (N=677)
−log10(p)
Chromosome

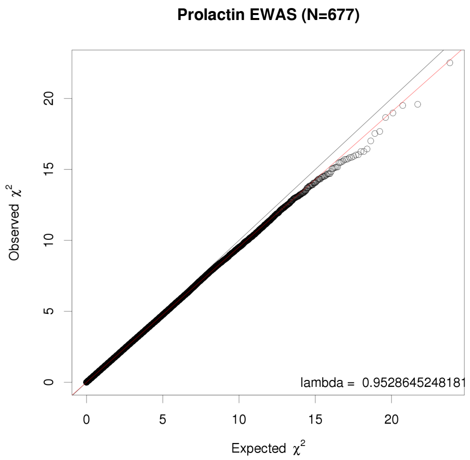
Prolactin EWAS (N=677)
Observed χ2
Expected χ2
lambda = 0.9528645248181

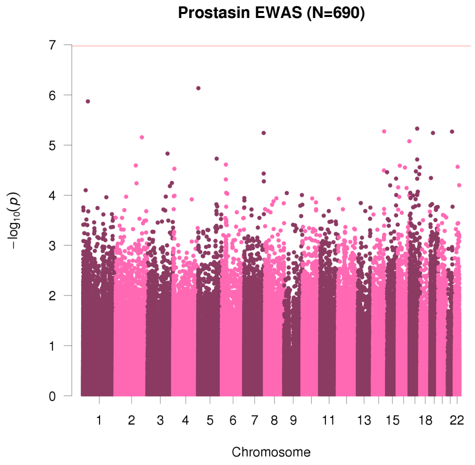
Prostasin EWAS (N=690)
−log10(p)
Chromosome

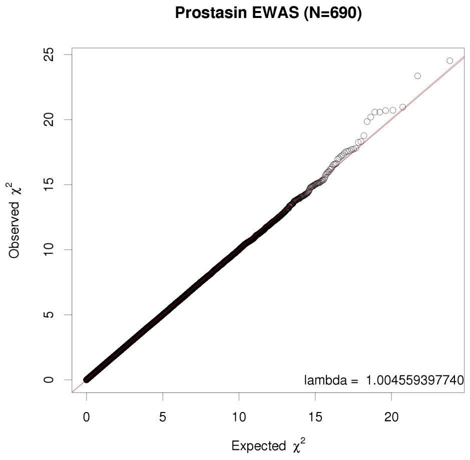
Prostasin EWAS (N=690)
Observed χ2
Expected χ2
lambda = 1.004559397740

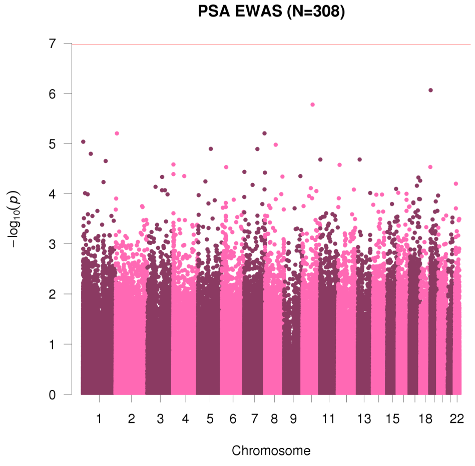
PSA EWAS (N=308)
−log10(p)
Chromosome

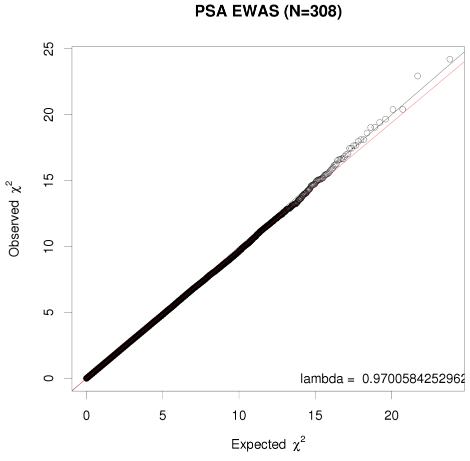
PSA EWAS (N=308)
Observed χ2
Expected χ2
lambda = 0.9700584252962

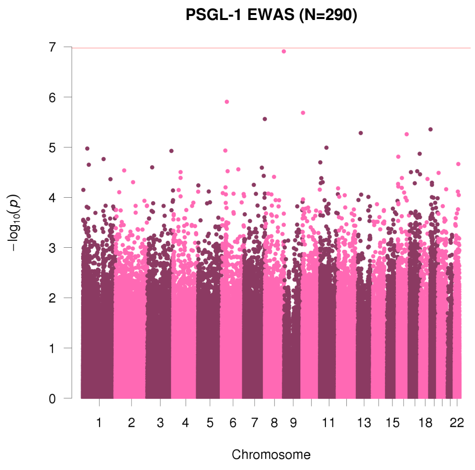
PSGL-1 EWAS (N=290)
−log10(p)
Chromosome

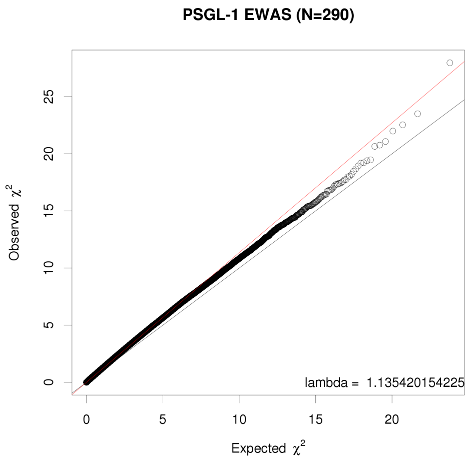
PSGL-1 EWAS (N=290)
Observed χ2
Expected χ2
lambda = 1.135420154225

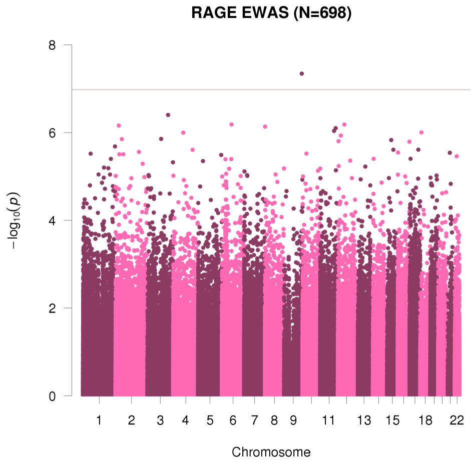
RAGE EWAS (N=698)
−log10(p)
Chromosome

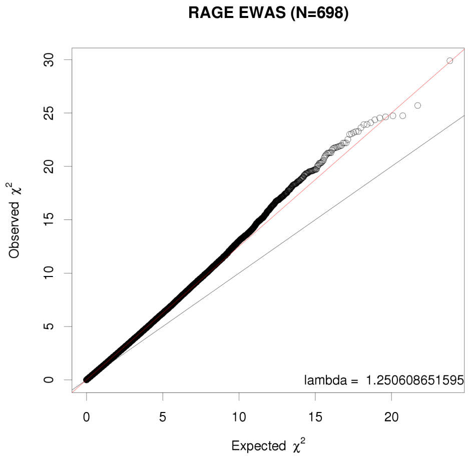
RAGE EWAS (N=698)
Observed χ2
Expected χ2
lambda = 1.250608651595

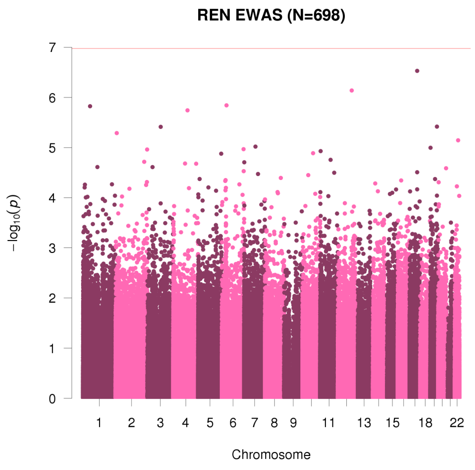
REN EWAS (N=698)
−log10(p)
Chromosome

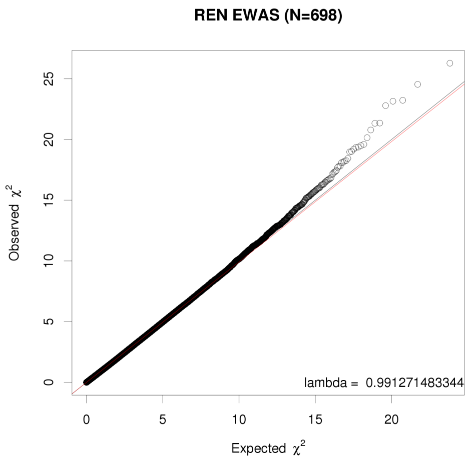
REN EWAS (N=698)
Observed χ2
Expected χ2
lambda = 0.991271483344

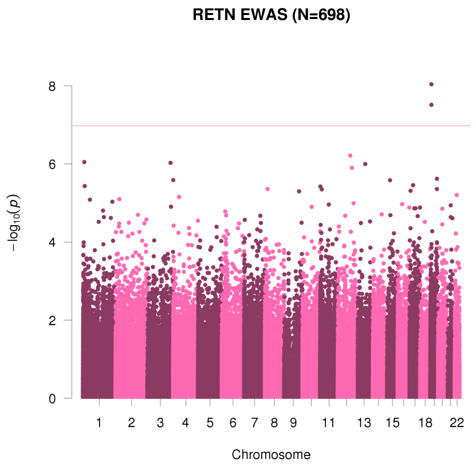
RETN EWAS (N=698)
−log10(p)
0
2
4
6
8
1 2 3 4 5 6 7 8 9 11 13 15 18 22
Chromosome

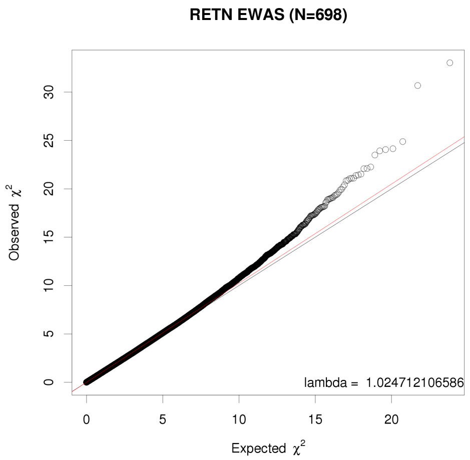
RETN EWAS (N=698)
Observed χ2
Expected χ2
0 5 10 15 20 25 30
0 5 10 15 20
lambda = 1.024712106586

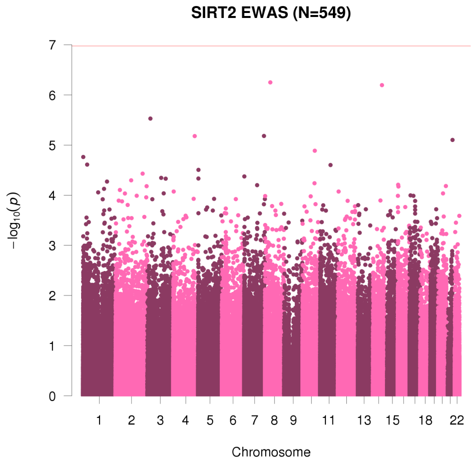
SIRT2 EWAS (N=549)
−log10(p)
0
1
2
3
4
5
6
7
1 2 3 4 5 6 7 8 9 11 13 15 18 22
Chromosome

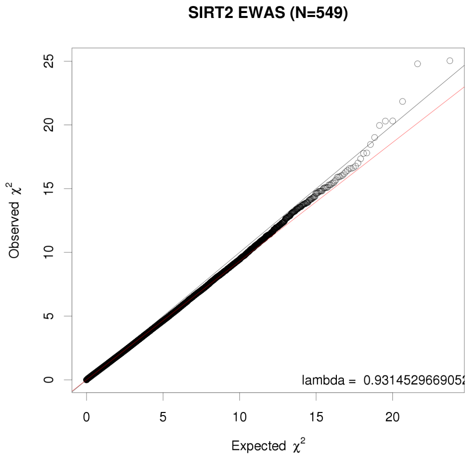
SIRT2 EWAS (N=549)
Observed χ2
Expected χ2
0 5 10 15 20 25
0 5 10 15 20
lambda = 0.931452966905

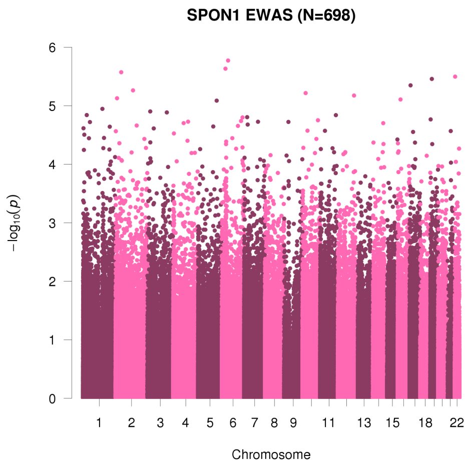
SPON1 EWAS (N=698)
−log10(p)
0
1
2
3
4
5
6
1 2 3 4 5 6 7 8 9 11 13 15 18 22
Chromosome

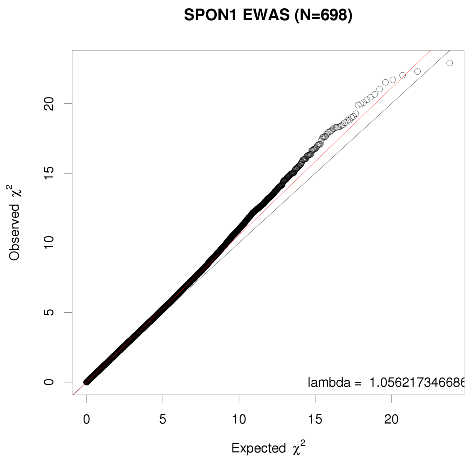
SPON1 EWAS (N=698)
Observed χ2
Expected χ2
0 5 10 15 20
0 5 10 15 20
lambda = 1.05621734668

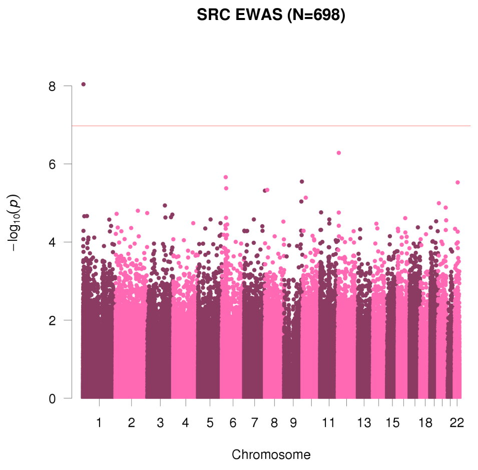
SRC EWAS (N=698)
−log10(p)
0
2
4
6
8
1 2 3 4 5 6 7 8 9 11 13 15 18 22
Chromosome

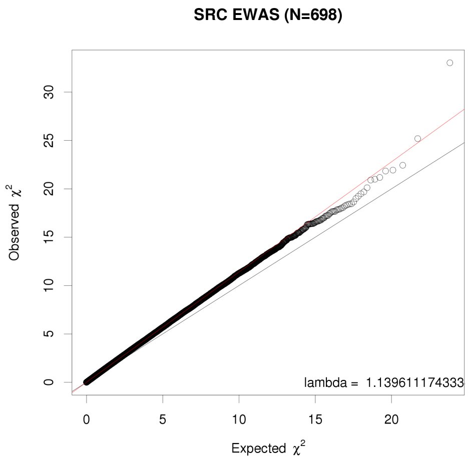
SRC EWAS (N=698)
Observed χ2
0 5 10 15 20 25 30
Expected χ2
0 5 10 15 20
lambda = 1.139611174333

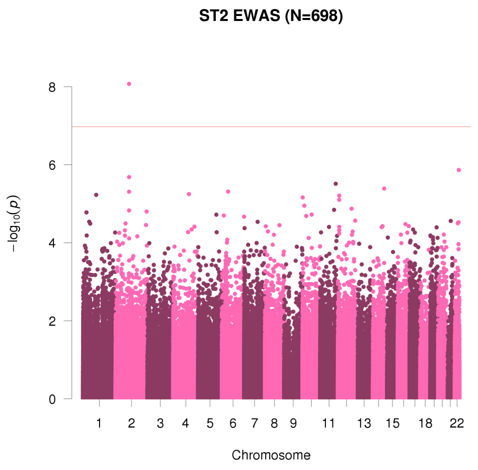
ST2 EWAS (N=698)
−log10(p)
0
2
4
6
8
1 2 3 4 5 6 7 8 9 11 13 15 18 22
Chromosome

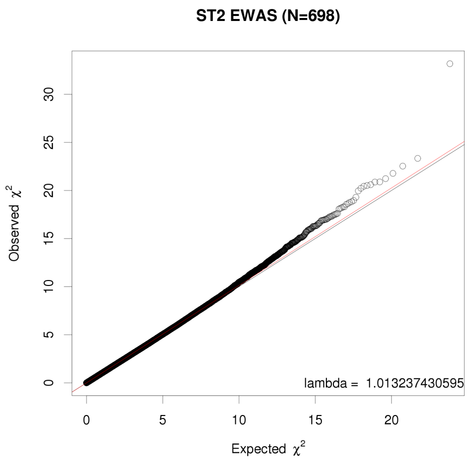
ST2 EWAS (N=698)
Observed χ2
0 5 10 15 20 25 30
Expected χ2
0 5 10 15 20
lambda = 1.013237430595

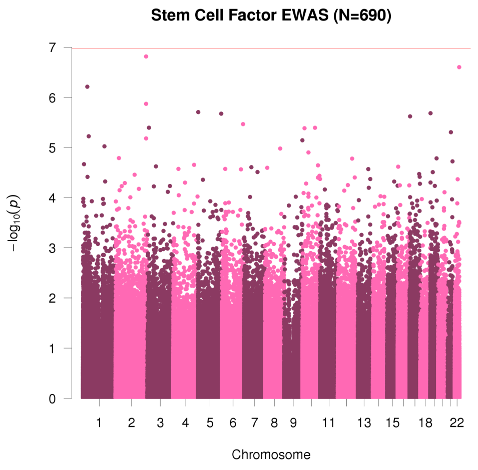
Stem Cell Factor EWAS (N=690)
−log10(p)
0
1
2
3
4
5
6
7
1 2 3 4 5 6 7 8 9 11 13 15 18 22
Chromosome

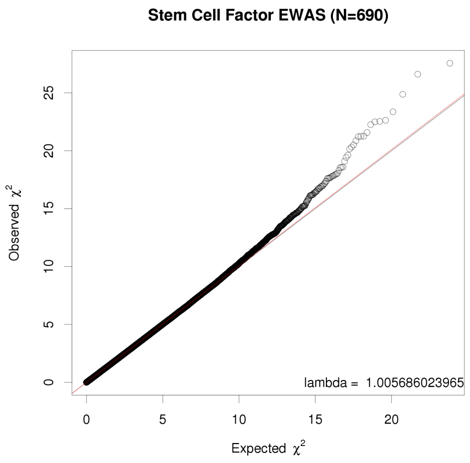
Stem Cell Factor EWAS (N=690)
Observed χ2
0 5 10 15 20 25
Expected χ2
0 5 10 15 20
lambda = 1.005686023965

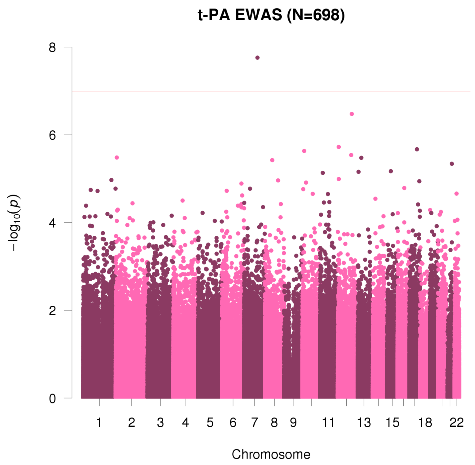
t-PA EWAS (N=698)
−log10(p)
Chromosome

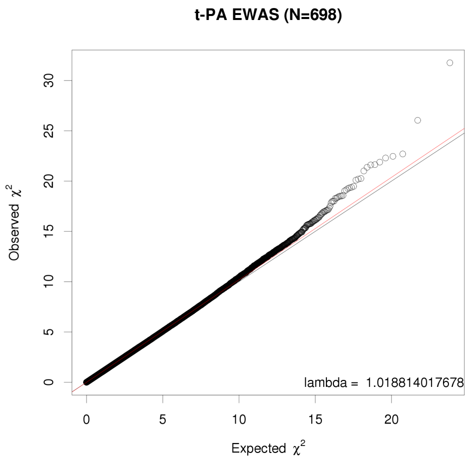
t-PA EWAS (N=698)
Observed χ2
Expected χ2
lambda = 1.018814017678

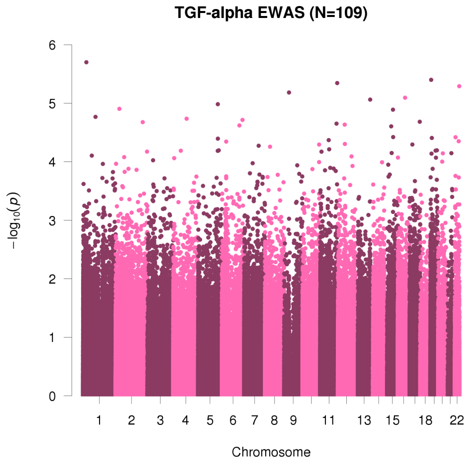
TGF-alpha EWAS (N=109)
−log10(p)
Chromosome

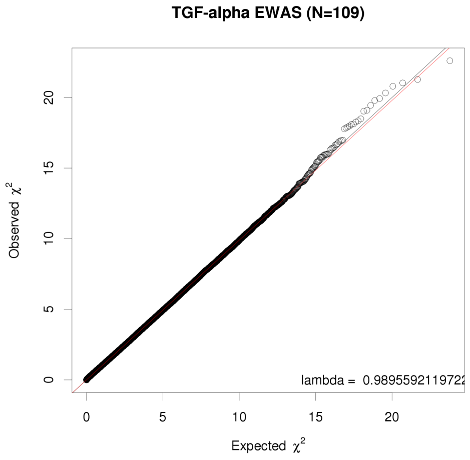
TGF-alpha EWAS (N=109)
Observed χ2
Expected χ2

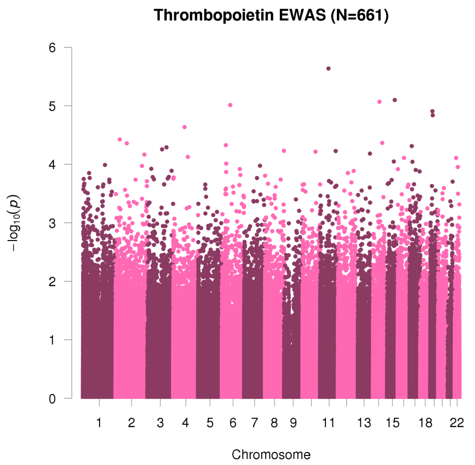
Thrombopoietin EWAS (N=661)
−log10(p)
Chromosome

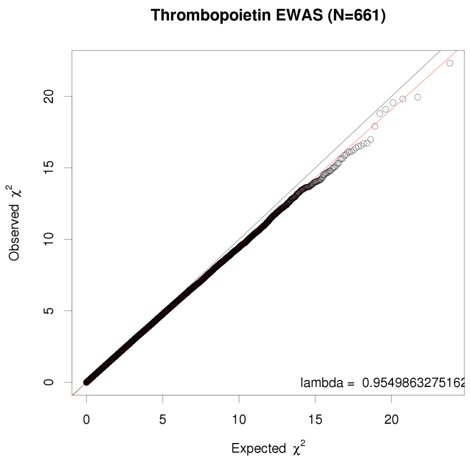
Thrombopoietin EWAS (N=661)
Observed χ2
Expected χ2

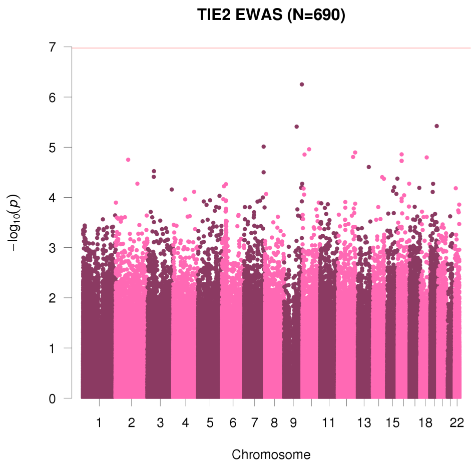
TIE2 EWAS (N=690)
$-\log_{10}(p)$
Chromosome
1 2 3 4 5 6 7 8 9 11 13 15 18 22

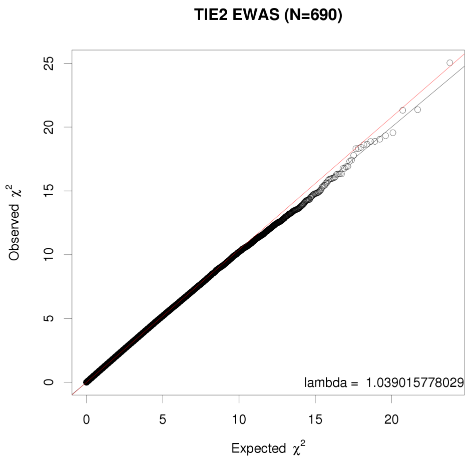
TIE2 EWAS (N=690)
Observed χ^2
Expected χ^2
lambda = 1.039015778029

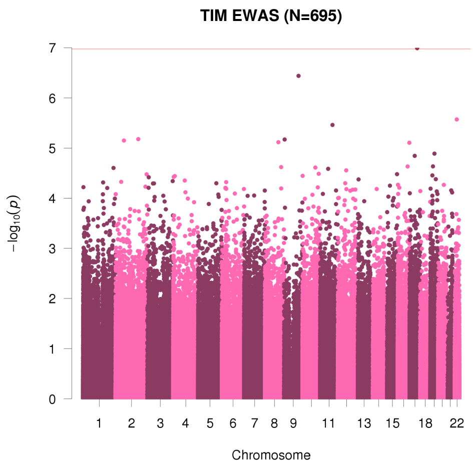
TIM EWAS (N=695)
$-\log_{10}(p)$
Chromosome
1 2 3 4 5 6 7 8 9 11 13 15 18 22

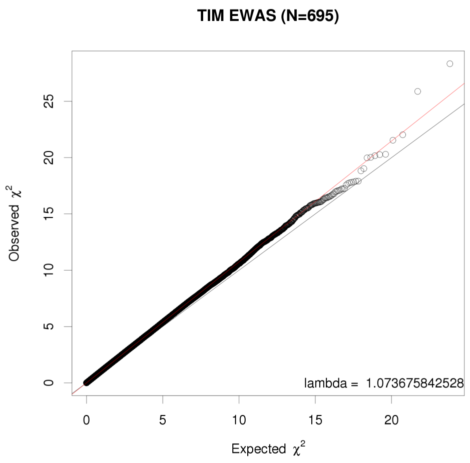
TIM EWAS (N=695)
Observed χ^2
Expected χ^2
lambda = 1.073675842528

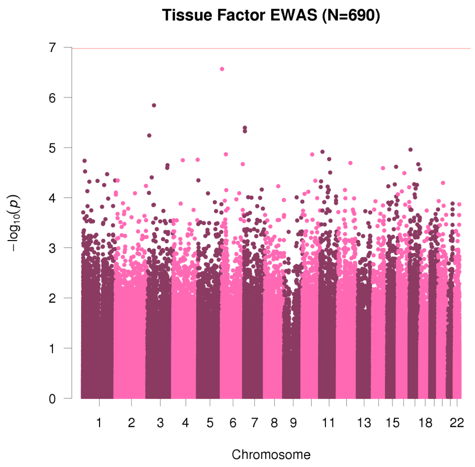
Tissue Factor EWAS (N=690)
$-\log_{10}(p)$
Chromosome
1 2 3 4 5 6 7 8 9 11 13 15 18 22

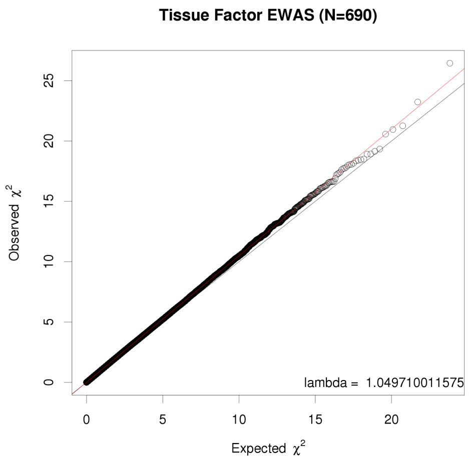
Tissue Factor EWAS (N=690)
Observed χ^2
Expected χ^2
lambda = 1.049710011575

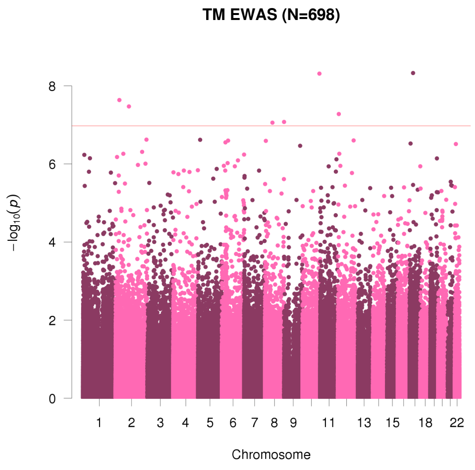
TM EWAS (N=698)
−log10(p)
Chromosome

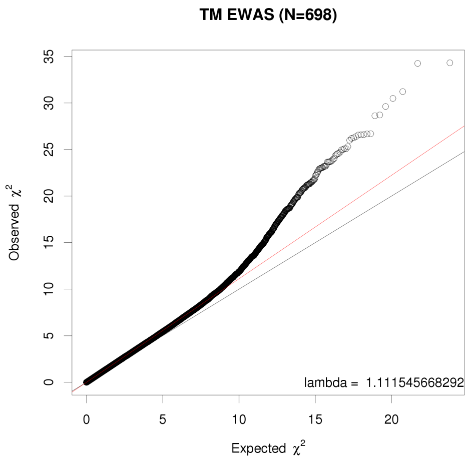
TM EWAS (N=698)
Observed χ2
Expected χ2
lambda = 1.111545668292

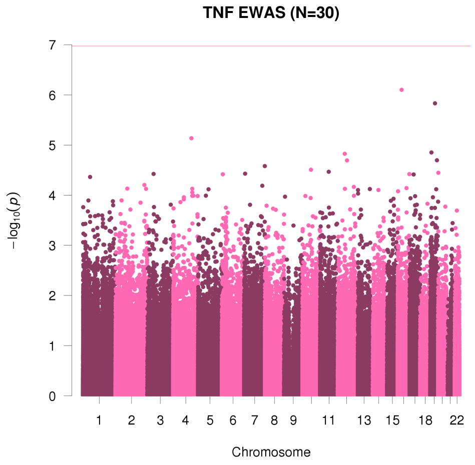
TNF EWAS (N=30)
−log10(p)
Chromosome

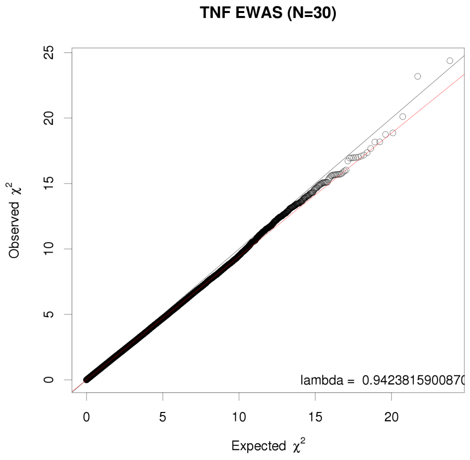
TNF EWAS (N=30)
Observed χ2
Expected χ2
lambda = 0.942381590087

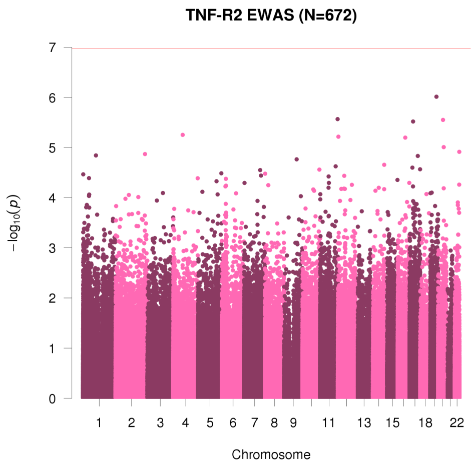
TNF-R2 EWAS (N=672)
−log10(p)
Chromosome

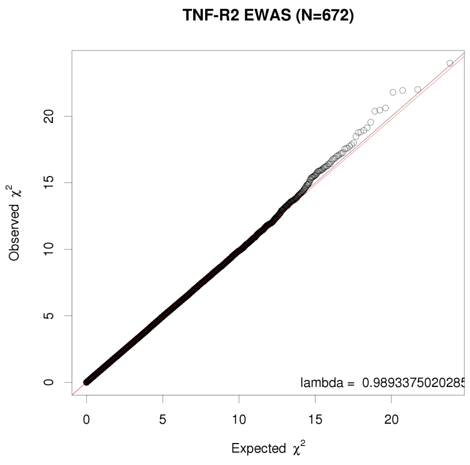
TNF-R2 EWAS (N=672)
Observed χ2
Expected χ2
lambda = 0.989337502028

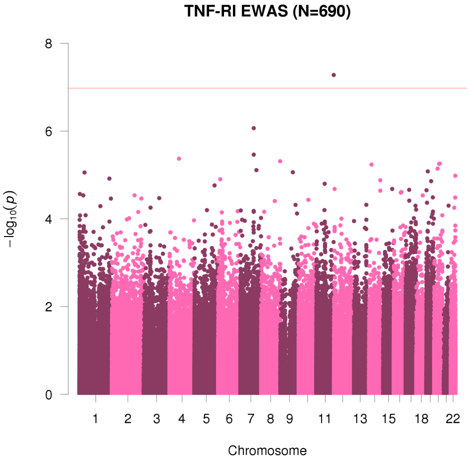
TNF-RI EWAS (N=690)
$-\log_{10}(p)$
Chromosome
1 2 3 4 5 6 7 8 9 11 13 15 18 22

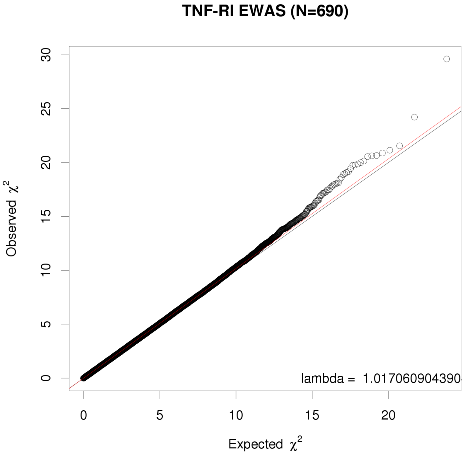
TNF-RI EWAS (N=690)
Observed χ^2
Expected χ^2
lambda = 1.017060904390

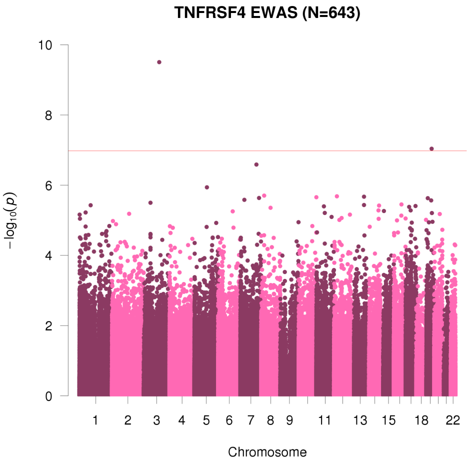
TNFRSF4 EWAS (N=643)
$-\log_{10}(p)$
Chromosome
1 2 3 4 5 6 7 8 9 11 13 15 18 22

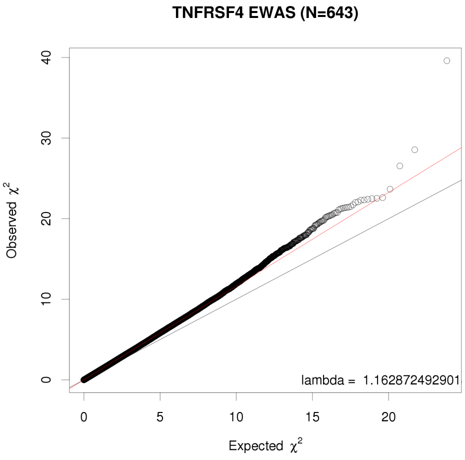
TNFRSF4 EWAS (N=643)
Observed χ^2
Expected χ^2
lambda = 1.162872492901

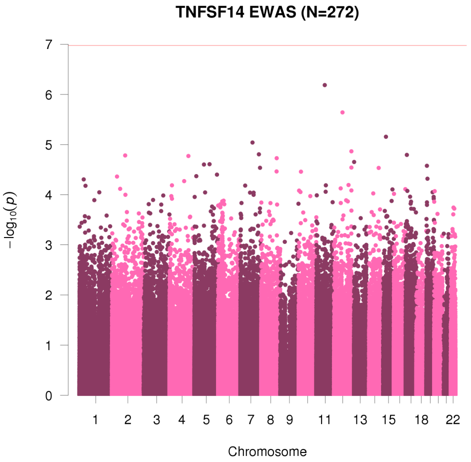
TNFSF14 EWAS (N=272)
$-\log_{10}(p)$
Chromosome
1 2 3 4 5 6 7 8 9 11 13 15 18 22

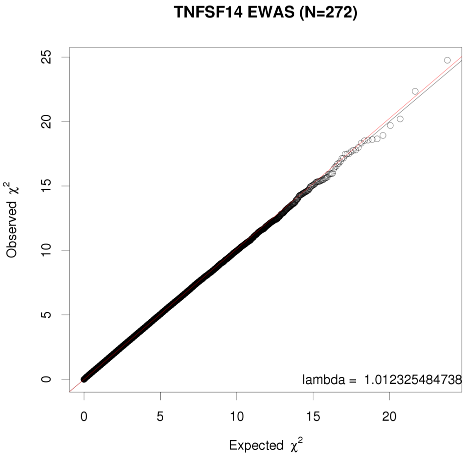
TNFSF14 EWAS (N=272)
Observed χ^2
Expected χ^2
lambda = 1.012325484738

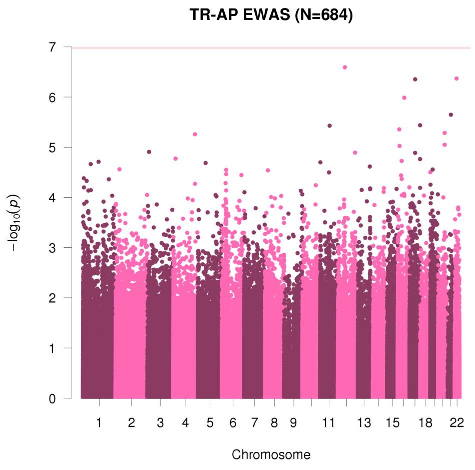
TR-AP EWAS (N=684)
$-\log_{10}(p)$
Chromosome

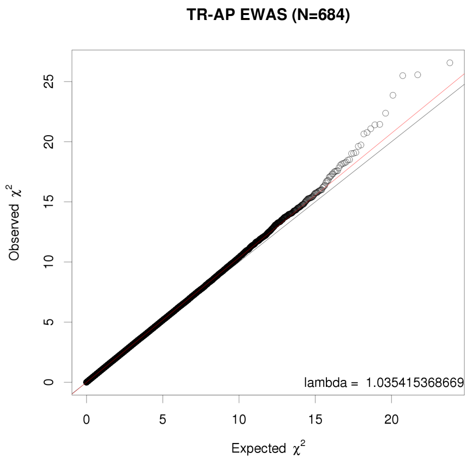
TR-AP EWAS (N=684)
Observed χ^2
Expected χ^2
lambda = 1.035415368669

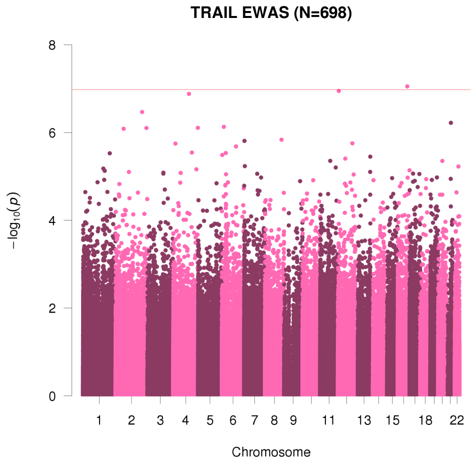
TRAIL EWAS (N=698)
$-\log_{10}(p)$
Chromosome

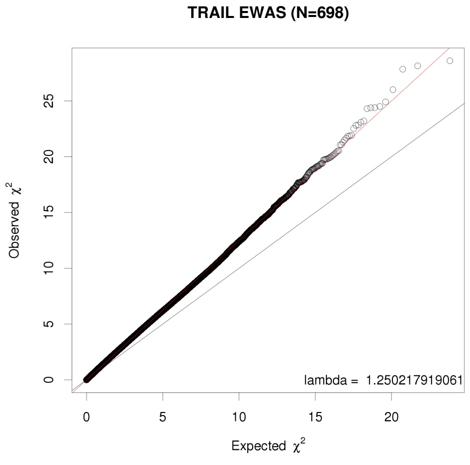
TRAIL EWAS (N=698)
Observed χ^2
Expected χ^2
lambda = 1.250217919061

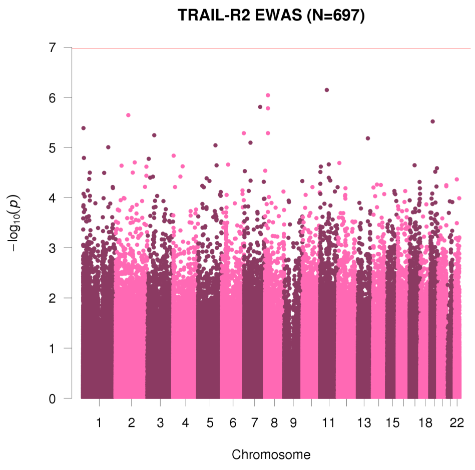
TRAIL-R2 EWAS (N=697)
$-\log_{10}(p)$
Chromosome

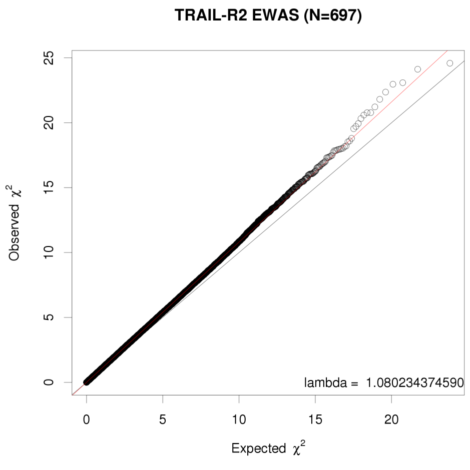
TRAIL-R2 EWAS (N=697)
Observed χ^2
Expected χ^2
lambda = 1.080234374590

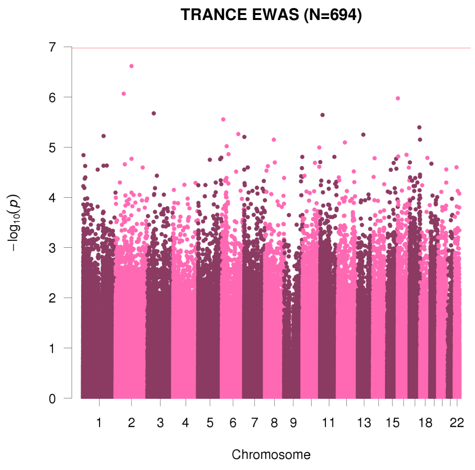
TRANCE EWAS (N=694)
$-\log_{10}(p)$
Chromosome
1 2 3 4 5 6 7 8 9 11 13 15 18 22

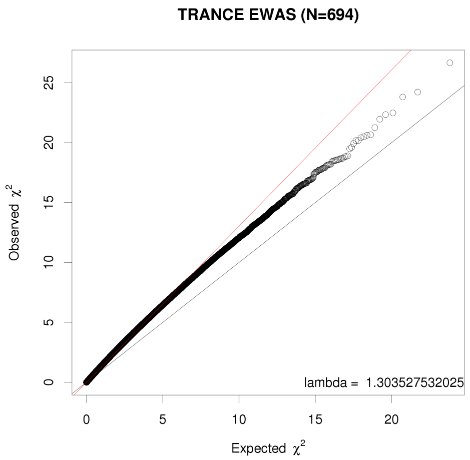
TRANCE EWAS (N=694)
Observed χ^2
Expected χ^2
lambda = 1.303527532025

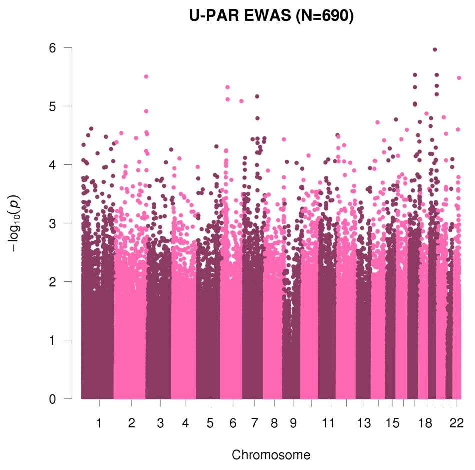
U-PAR EWAS (N=690)
$-\log_{10}(p)$
Chromosome
1 2 3 4 5 6 7 8 9 11 13 15 18 22

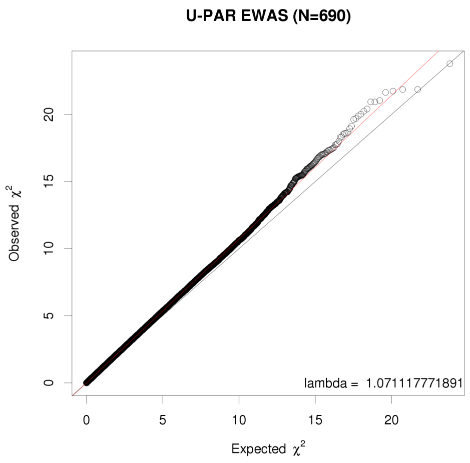
U-PAR EWAS (N=690)
Observed χ^2
Expected χ^2
lambda = 1.071117771891

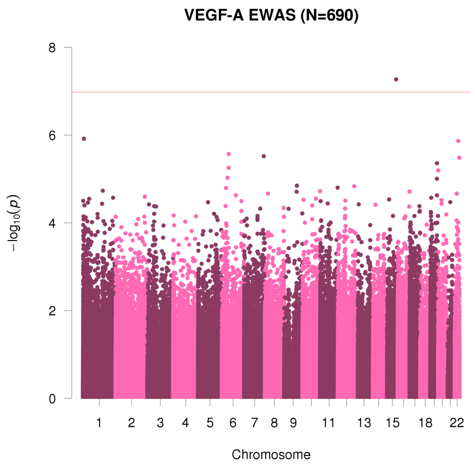
VEGF-A EWAS (N=690)
$-\log_{10}(p)$
Chromosome
1 2 3 4 5 6 7 8 9 11 13 15 18 22

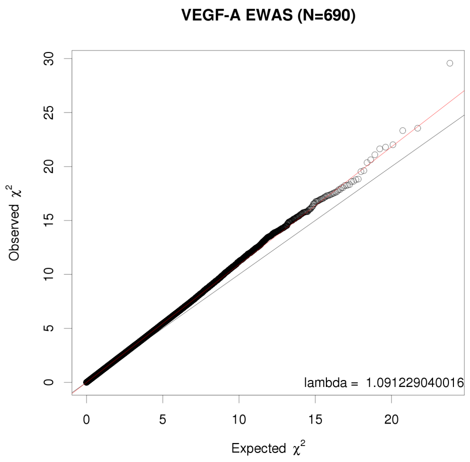
VEGF-A EWAS (N=690)
Observed χ^2
Expected χ^2
lambda = 1.091229040016

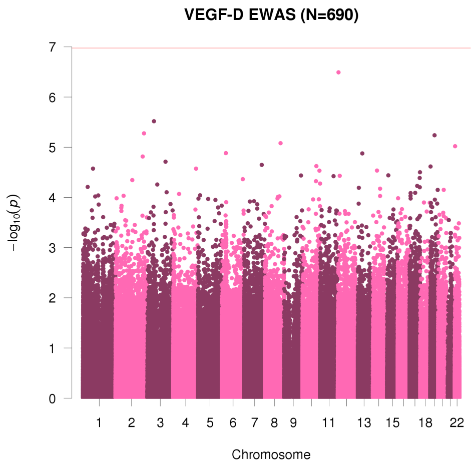
VEGF-D EWAS (N=690)
$-\log_{10}(p)$
Chromosome
1 2 3 4 5 6 7 8 9 11 13 15 18 22

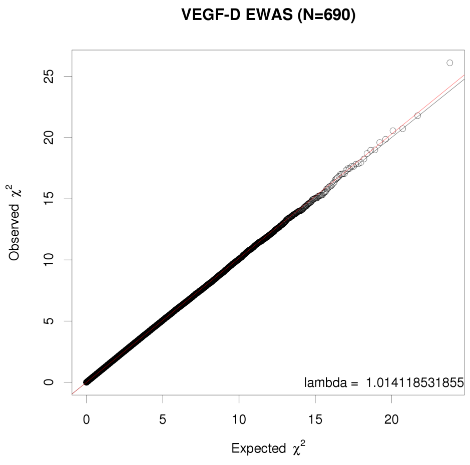
VEGF-D EWAS (N=690)
Observed χ^2
Expected χ^2
lambda = 1.014118531855

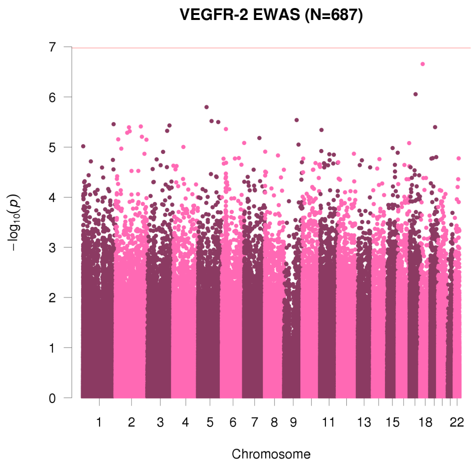
VEGFR-2 EWAS (N=687)
$-\log_{10}(p)$
Chromosome
1 2 3 4 5 6 7 8 9 11 13 15 18 22

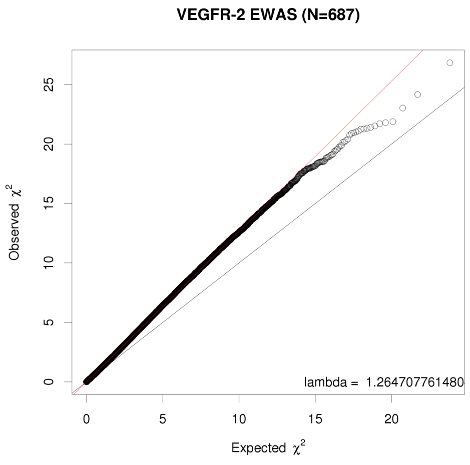
VEGFR-2 EWAS (N=687)
Observed χ^2
Expected χ^2
lambda = 1.264707761480
