# Supplementary material for: The relative contribution of DNA methylation and genetic variants on protein biomarkers for human diseases
Source: PLoS Genet. 2017 Sep 15;13(9):e1007005. doi: 10.1371/journal.pgen.1007005 (PMC5617224; doi:10.1371/journal.pgen.1007005)
Supplement: S4 Fig — A total of 18 biomarkers with both EWAS and GWAS hits are included. (PDF) [file pgen.1007005.s004.pdf]

***Supplemental Fig S4.*** *Manhattan plots for EWAS results when adjusted for independent GWAS SNPs. A total of 18 biomarkers with both EWAS and GWAS hits are included.*

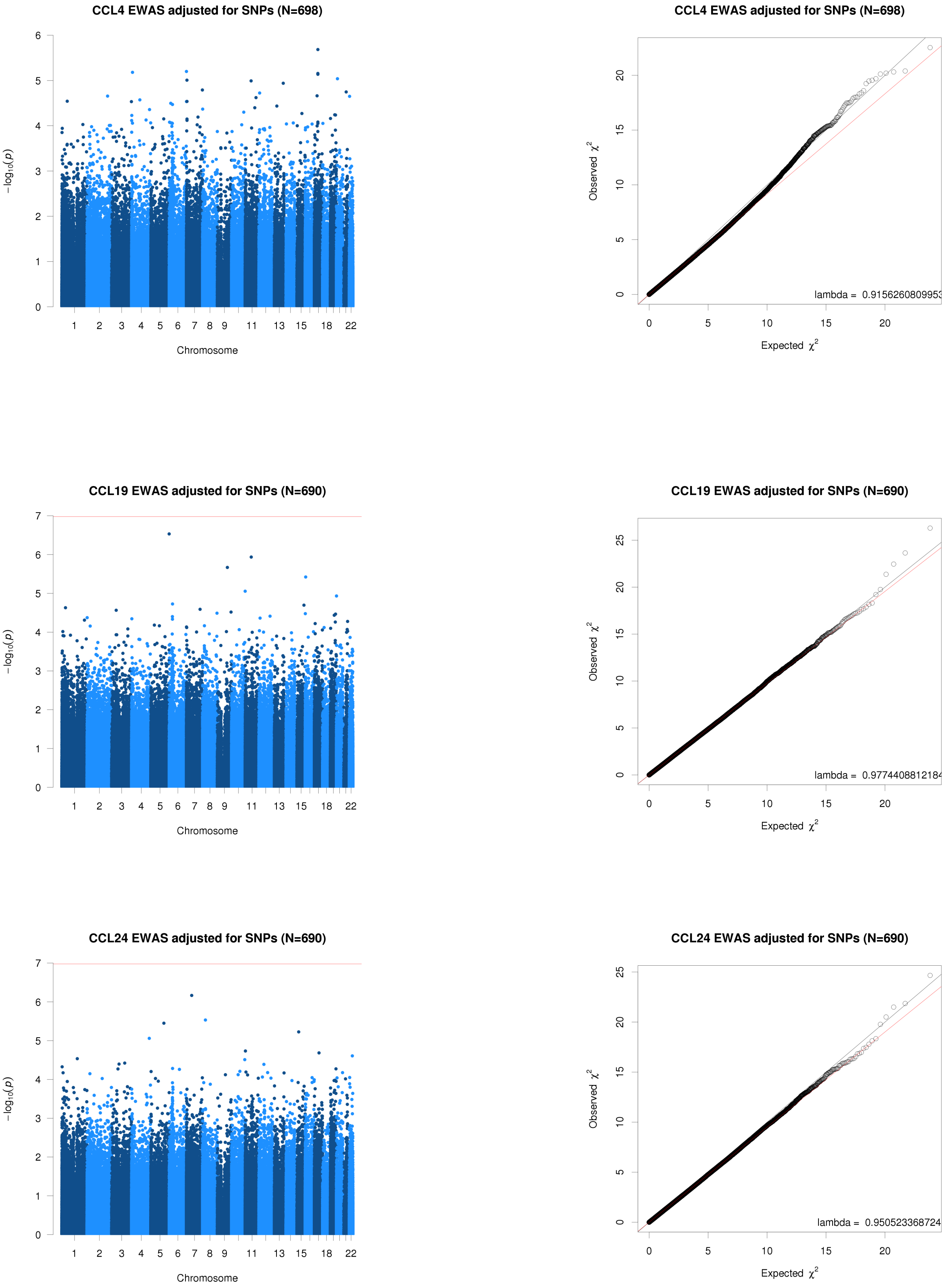

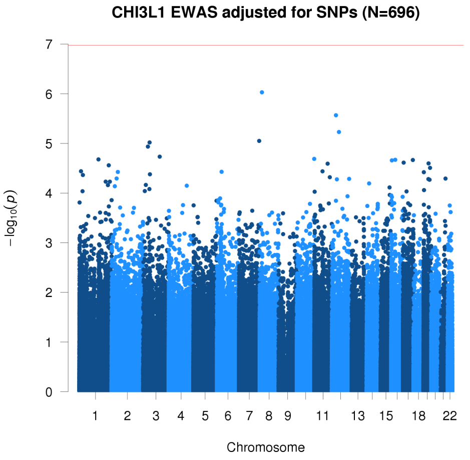
CHI3L1 EWAS adjusted for SNPs (N=696)
$-\log_{10}(p)$
Chromosome

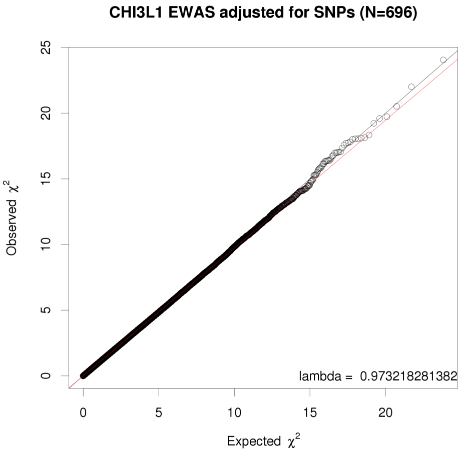
CHI3L1 EWAS adjusted for SNPs (N=696)
Observed $\chi^2$
Expected $\chi^2$
lambda = 0.973218281382

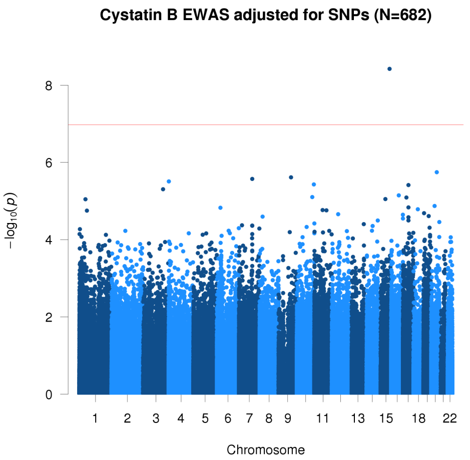
Cystatin B EWAS adjusted for SNPs (N=682)
$-\log_{10}(p)$
Chromosome

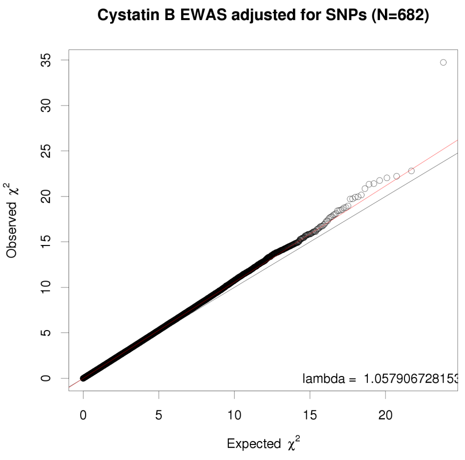
Cystatin B EWAS adjusted for SNPs (N=682)
Observed $\chi^2$
Expected $\chi^2$
lambda = 1.05790672815

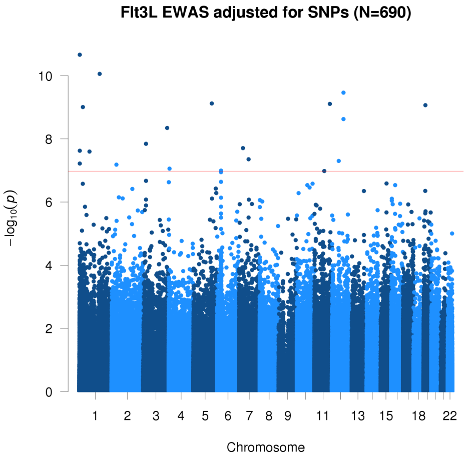
Flt3L EWAS adjusted for SNPs (N=690)
$-\log_{10}(p)$
Chromosome

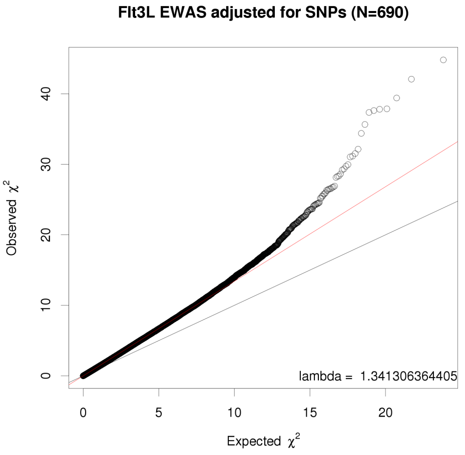
Flt3L EWAS adjusted for SNPs (N=690)
Observed $\chi^2$
Expected $\chi^2$
lambda = 1.341306364405

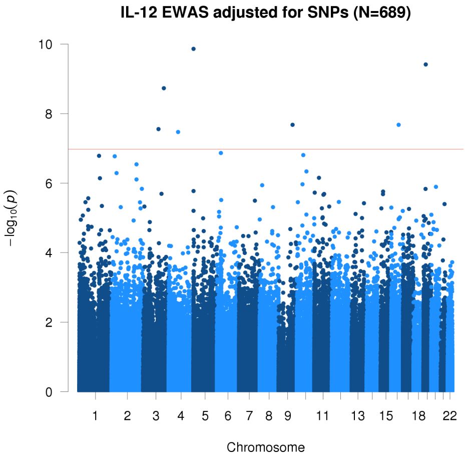
IL-12 EWAS adjusted for SNPs (N=689)
$-\log_{10}(p)$
Chromosome

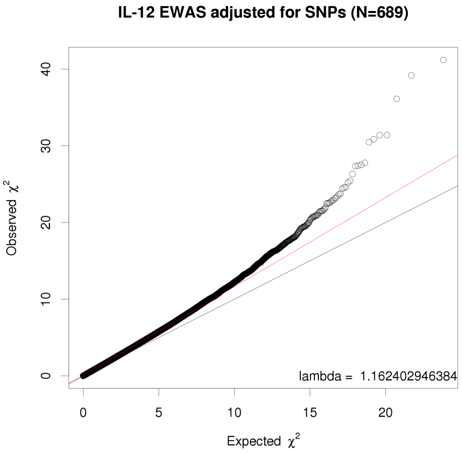
IL-12 EWAS adjusted for SNPs (N=689)
Observed $\chi^2$
Expected $\chi^2$
lambda = 1.162402946384

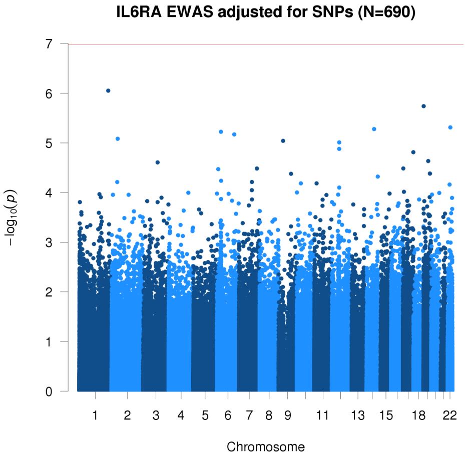
IL6RA EWAS adjusted for SNPs (N=690)
$-\log_{10}(p)$
Chromosome

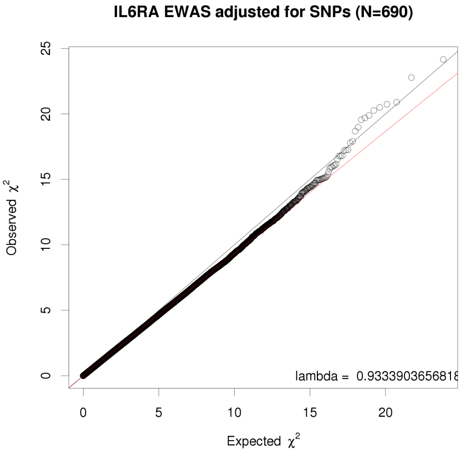
IL6RA EWAS adjusted for SNPs (N=690)
Observed $\chi^2$
Expected $\chi^2$
lambda = 0.933390365681

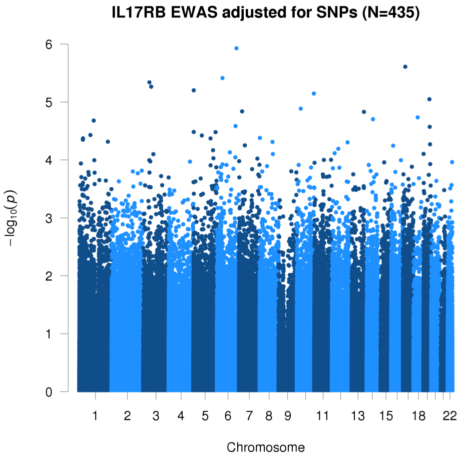
IL17RB EWAS adjusted for SNPs (N=435)
$-\log_{10}(p)$
Chromosome

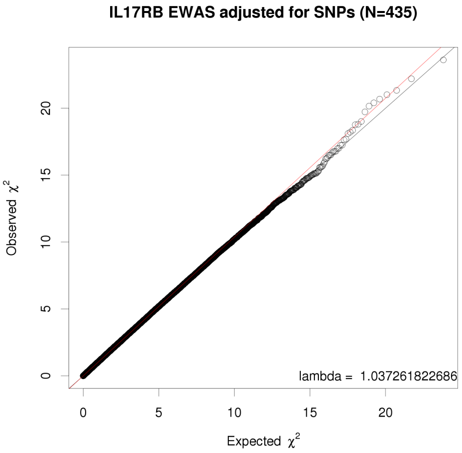
IL17RB EWAS adjusted for SNPs (N=435)
Observed $\chi^2$
Expected $\chi^2$
lambda = 1.037261822686

MIA EWAS adjusted for SNPs (N=685)

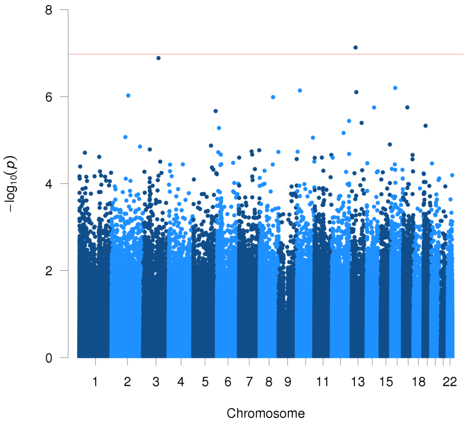


MIA EWAS adjusted for SNPs (N=685)

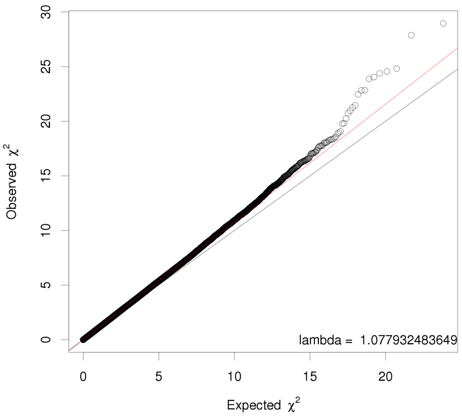


MIC-A EWAS adjusted for SNPs (N=379)

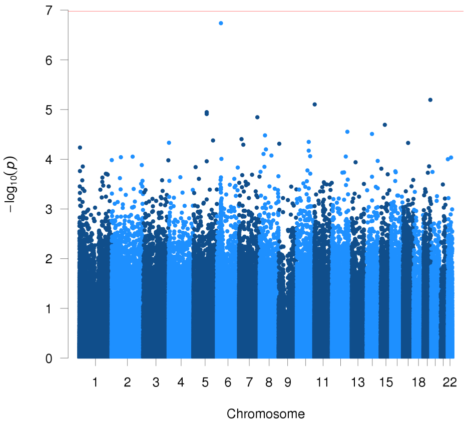


MIC-A EWAS adjusted for SNPs (N=379)

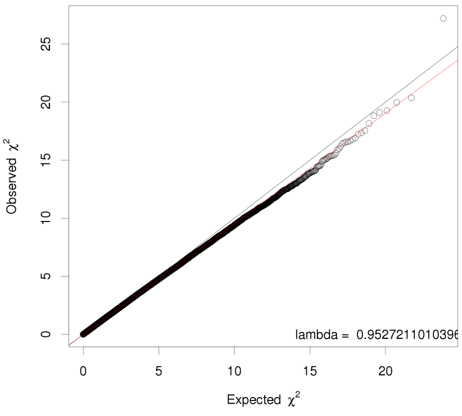


MMP-12 EWAS adjusted for SNPs (N=698)

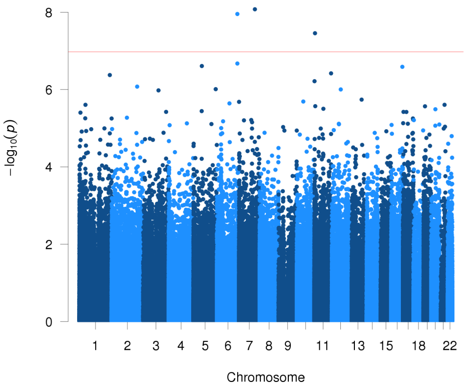


MMP-12 EWAS adjusted for SNPs (N=698)

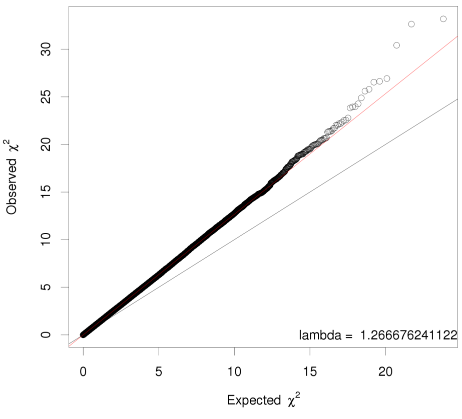

NEMO EWAS adjusted for SNPs (N=698)

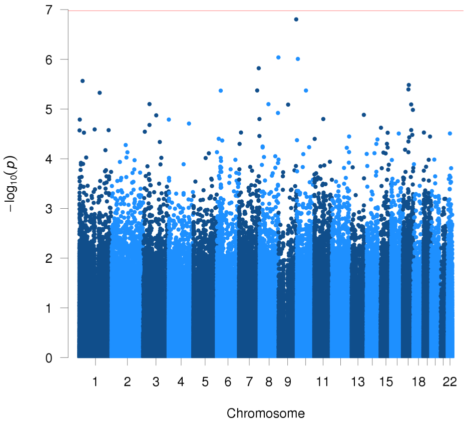


NEMO EWAS adjusted for SNPs (N=698)

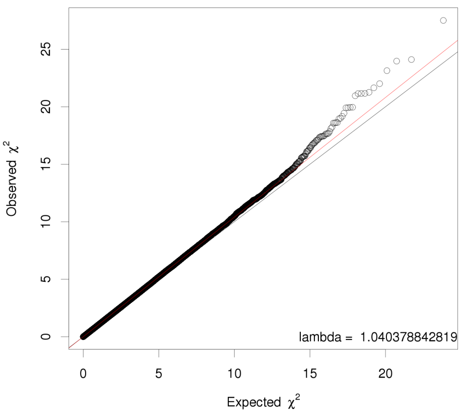


RETN EWAS adjusted for SNPs (N=698)

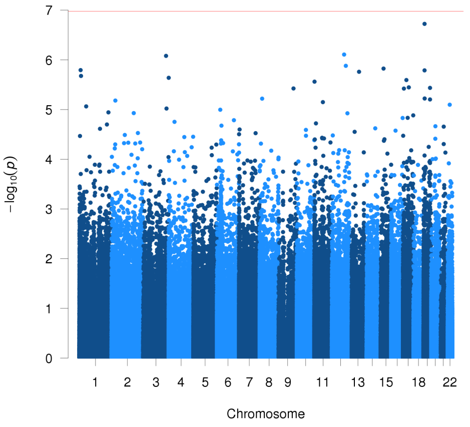


RETN EWAS adjusted for SNPs (N=698)

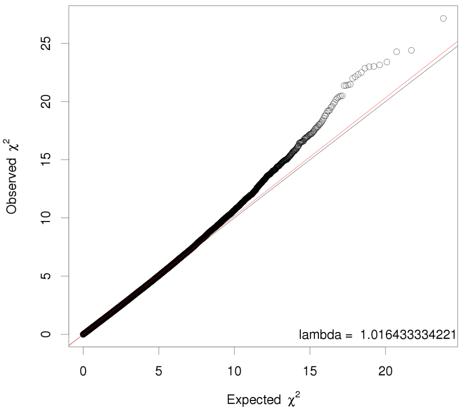


ST2 EWAS adjusted for SNPs (N=698)

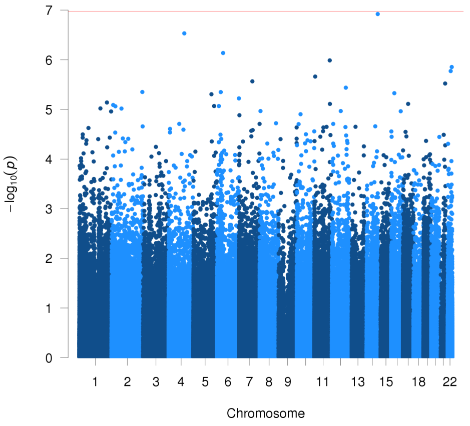


ST2 EWAS adjusted for SNPs (N=698)

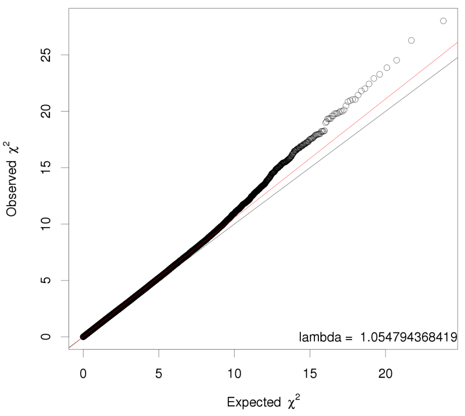

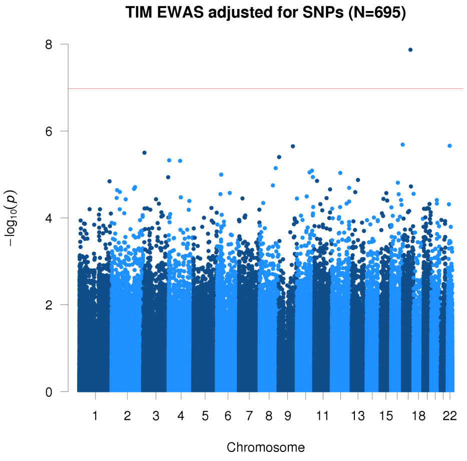
TIM EWAS adjusted for SNPs (N=695)
$-\log_{10}(p)$
Chromosome

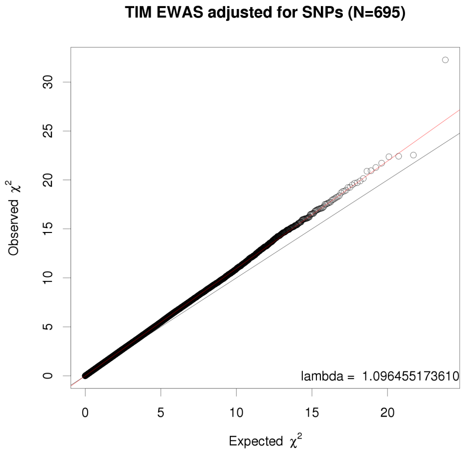
TIM EWAS adjusted for SNPs (N=695)
Observed $\chi^2$
Expected $\chi^2$
lambda = 1.096455173610

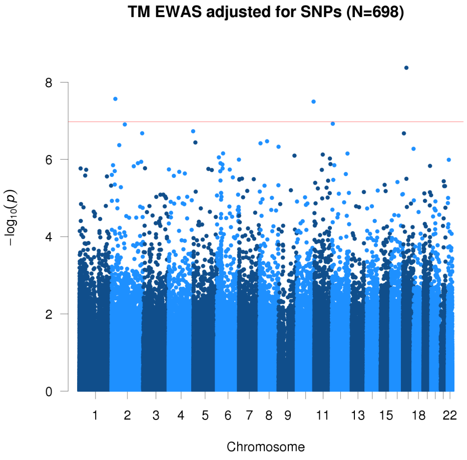
TM EWAS adjusted for SNPs (N=698)
$-\log_{10}(p)$
Chromosome

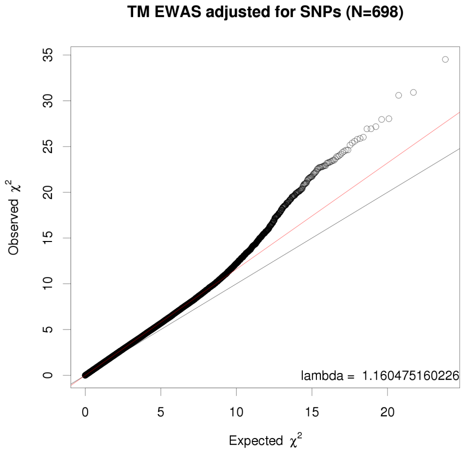
TM EWAS adjusted for SNPs (N=698)
Observed $\chi^2$
Expected $\chi^2$
lambda = 1.160475160226

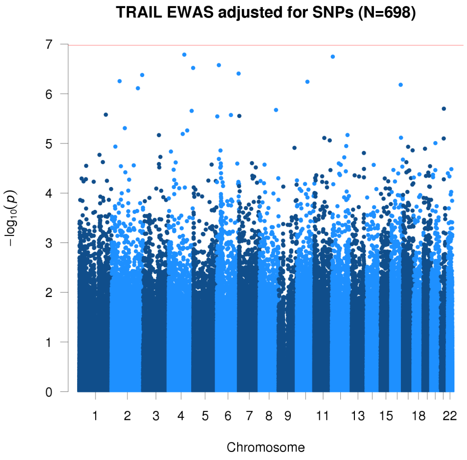
TRAIL EWAS adjusted for SNPs (N=698)
$-\log_{10}(p)$
Chromosome

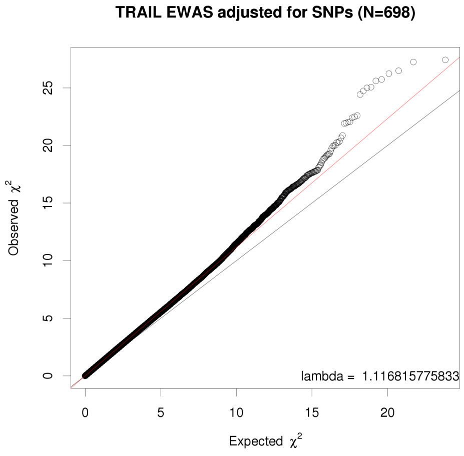
TRAIL EWAS adjusted for SNPs (N=698)
Observed $\chi^2$
Expected $\chi^2$
lambda = 1.116815775833
