# Supplementary material for: The relative contribution of DNA methylation and genetic variants on protein biomarkers for human diseases
Source: PLoS Genet. 2017 Sep 15;13(9):e1007005. doi: 10.1371/journal.pgen.1007005 (PMC5617224; doi:10.1371/journal.pgen.1007005)
Supplement: S5 Fig — Results from the different analyses are shown as: A) the primary EWAS, B) primary GWAS, C) EWAS adjusted for independent GWAS SNPs, and D) EWAS for GS, calculated from cis-regularity SNP, that influence biomarker levels. (PDF) [file pgen.1007005.s005.pdf]

Supplemental Fig S5. *Manhattan plots for comparison of the results. Results from the different analyses are shown as: A) the primary EWAS, B) primary GWAS, C) EWAS adjusted for independent GWAS SNPs, and D) EWAS for GS, calculated from cis-regularity SNP, that influence biomarker levels*.

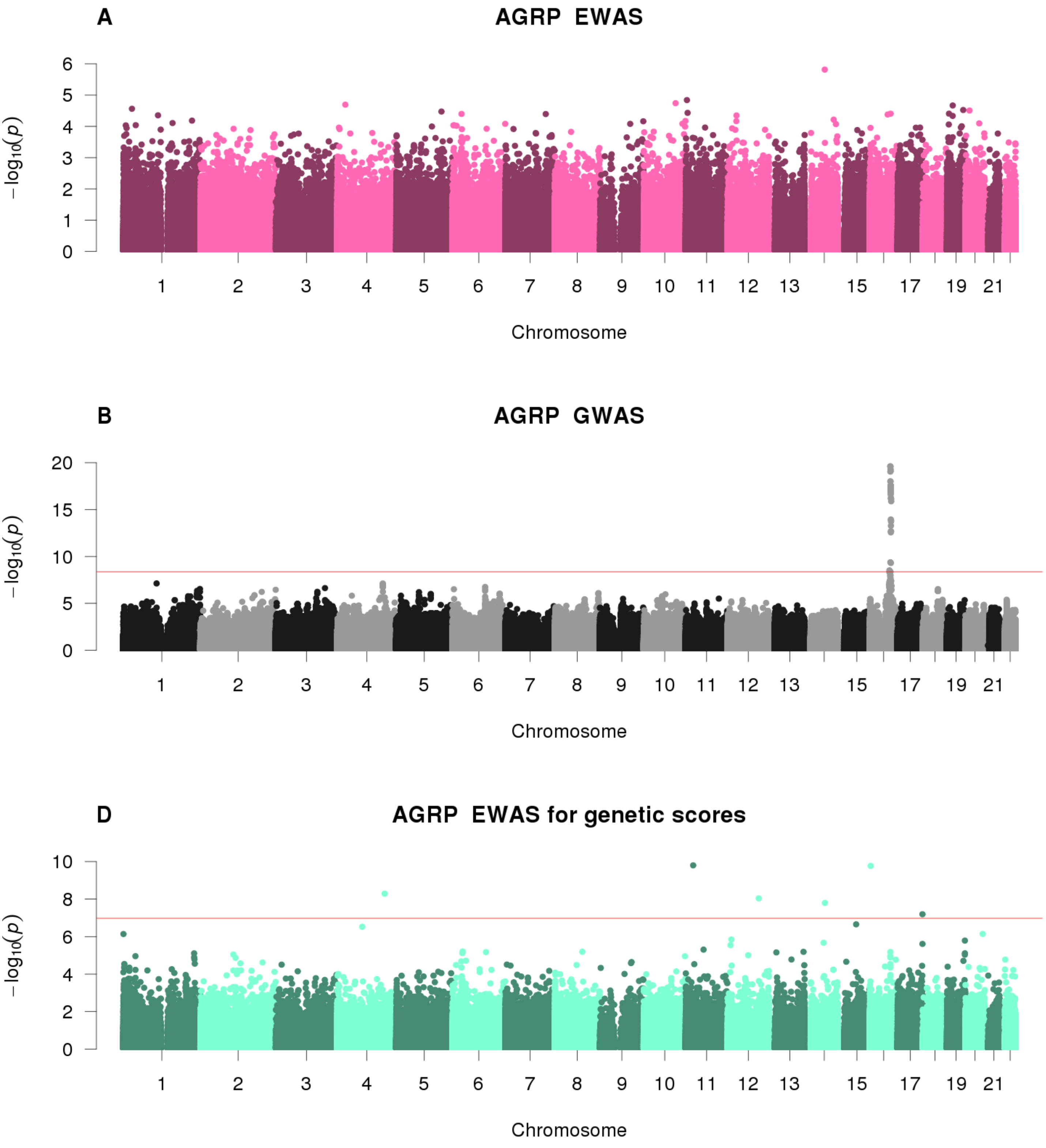

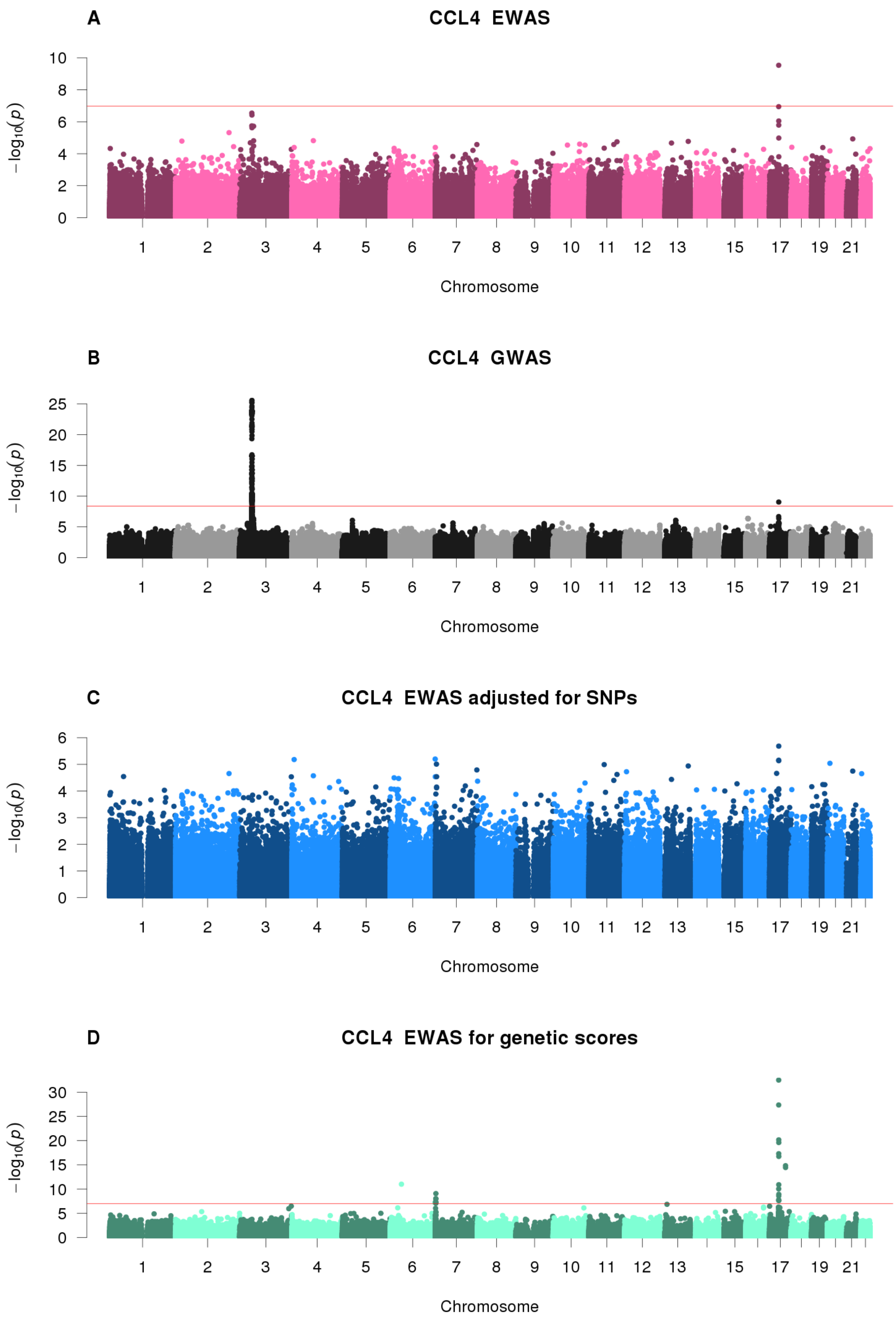
A
CCL4 EWAS
B
CCL4 GWAS
C
CCL4 EWAS adjusted for SNPs
D
CCL4 EWAS for genetic scores
$-\log_{10}(p)$
Chromosome
1 2 3 4 5 6 7 8 9 10 11 12 13 15 17 19 21

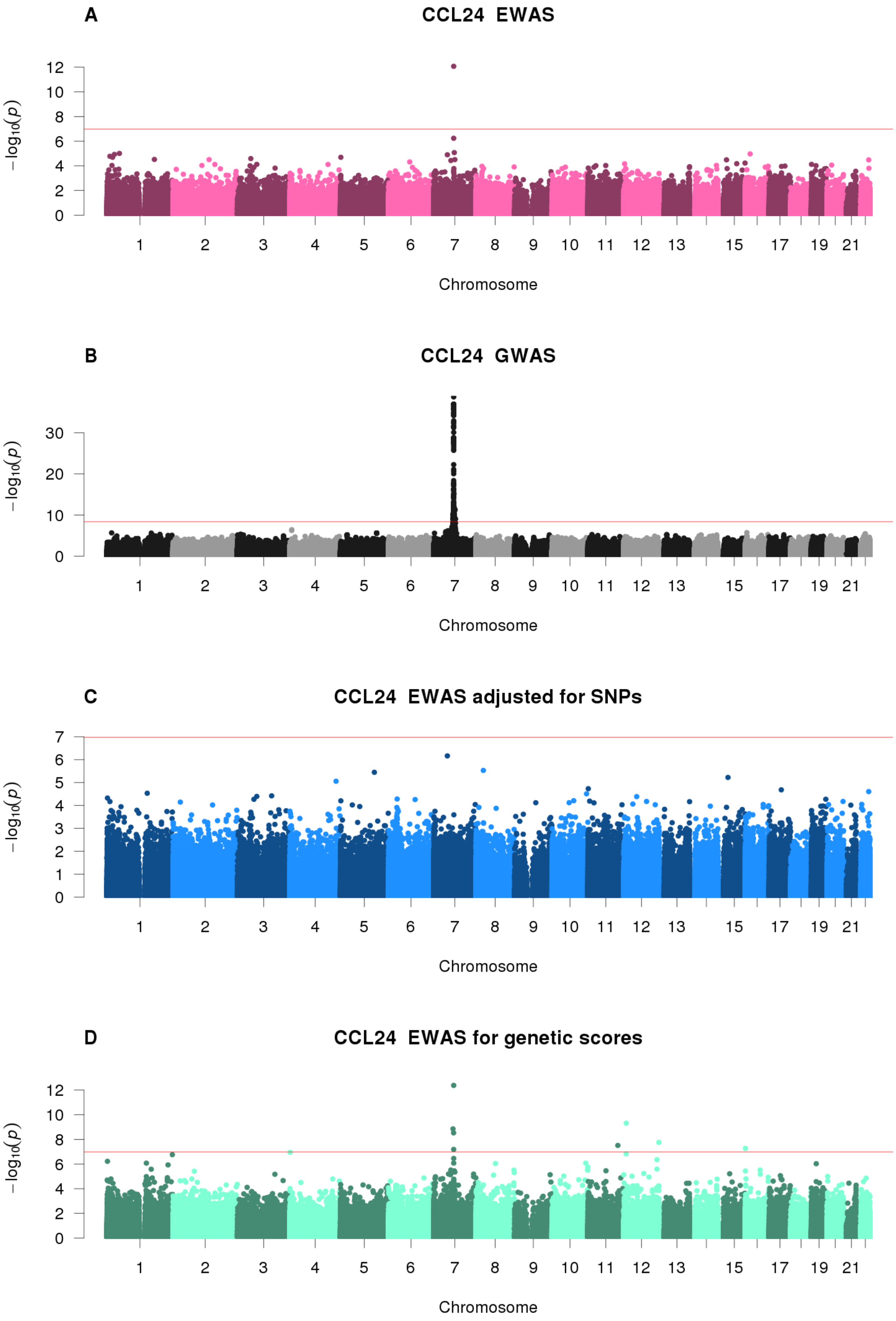

A
CCL24 EWAS
−log10(p)
Chromosome
B
CCL24 GWAS
−log10(p)
Chromosome
C
CCL24 EWAS adjusted for SNPs
−log10(p)
Chromosome
D
CCL24 EWAS for genetic scores
−log10(p)
Chromosome

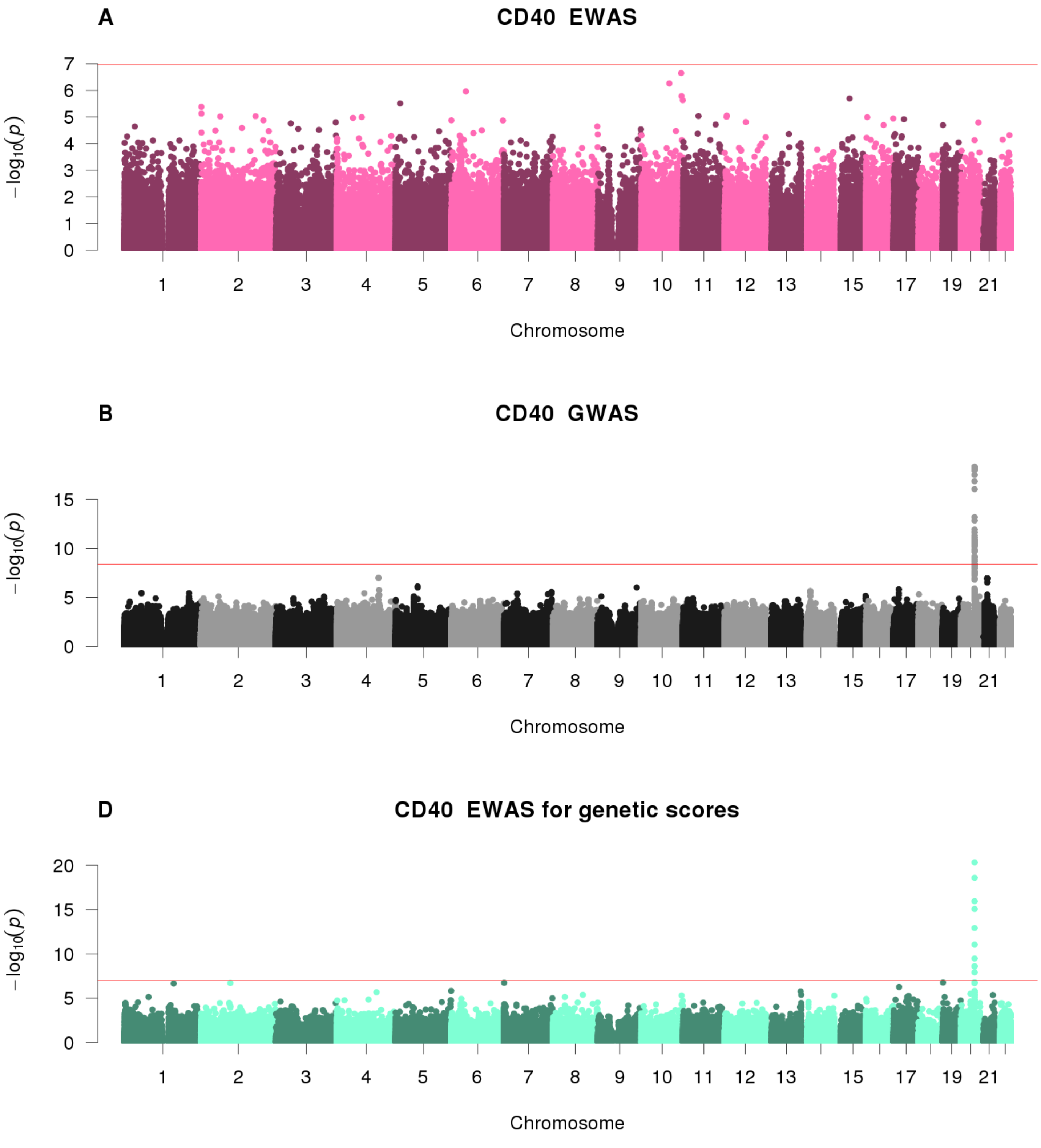
A
CD40 EWAS
−log10(p)
Chromosome
B
CD40 GWAS
−log10(p)
Chromosome
D
CD40 EWAS for genetic scores
−log10(p)
Chromosome

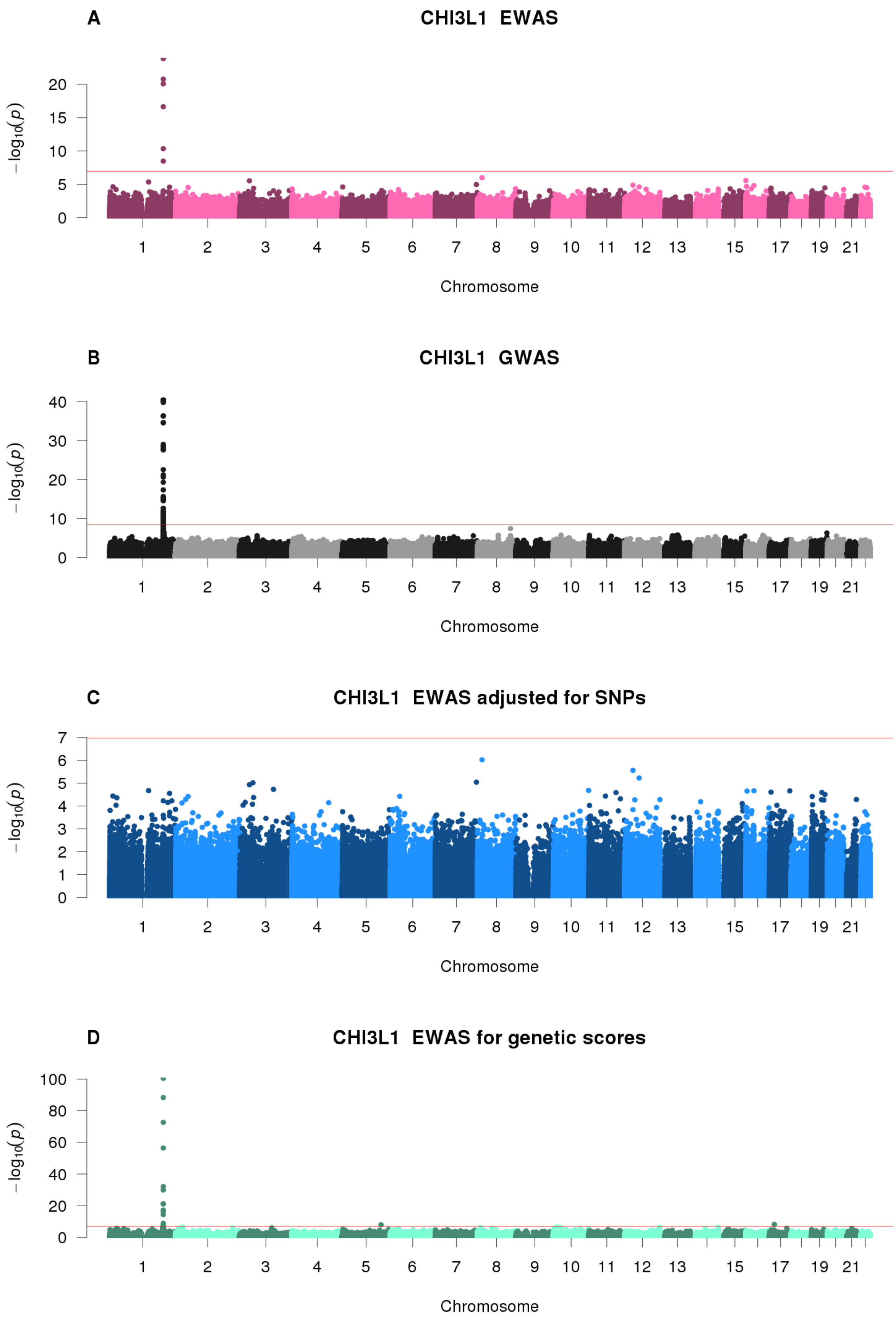
A
CHI3L1 EWAS
B
CHI3L1 GWAS
C
CHI3L1 EWAS adjusted for SNPs
D
CHI3L1 EWAS for genetic scores
−log10(p)
Chromosome
1 2 3 4 5 6 7 8 9 10 11 12 13 15 17 19 21

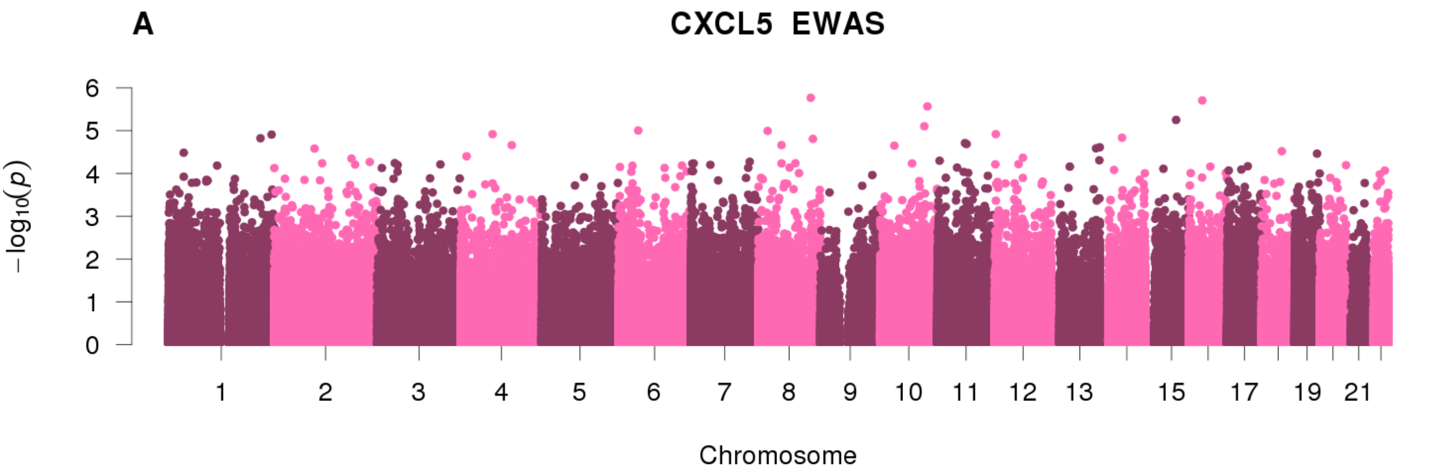
A
CXCL5 EWAS
−log10(p)
Chromosome

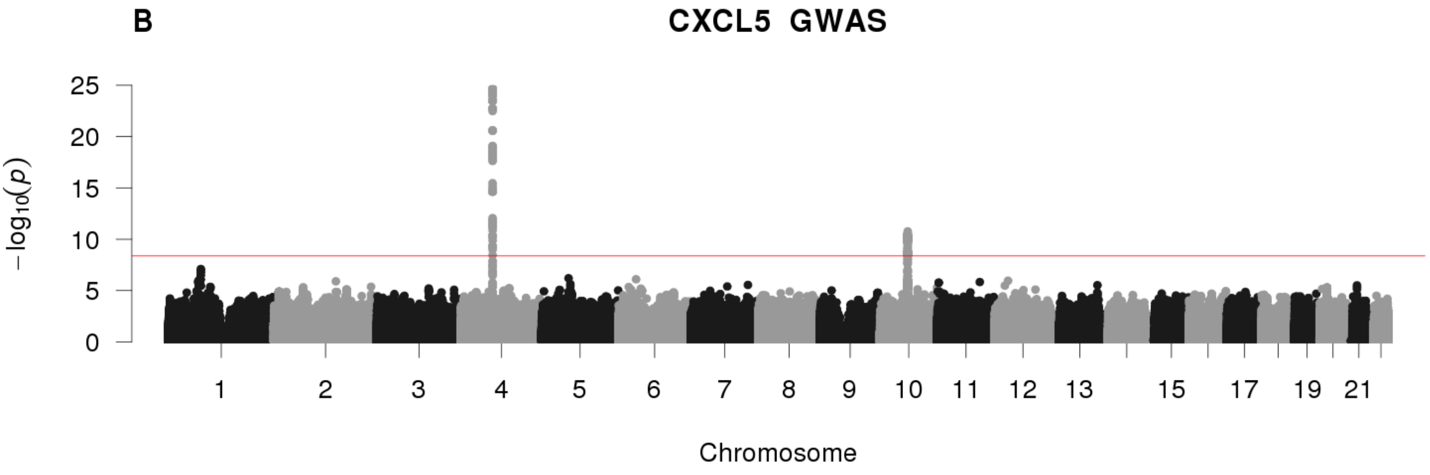
B
CXCL5 GWAS
−log10(p)
Chromosome

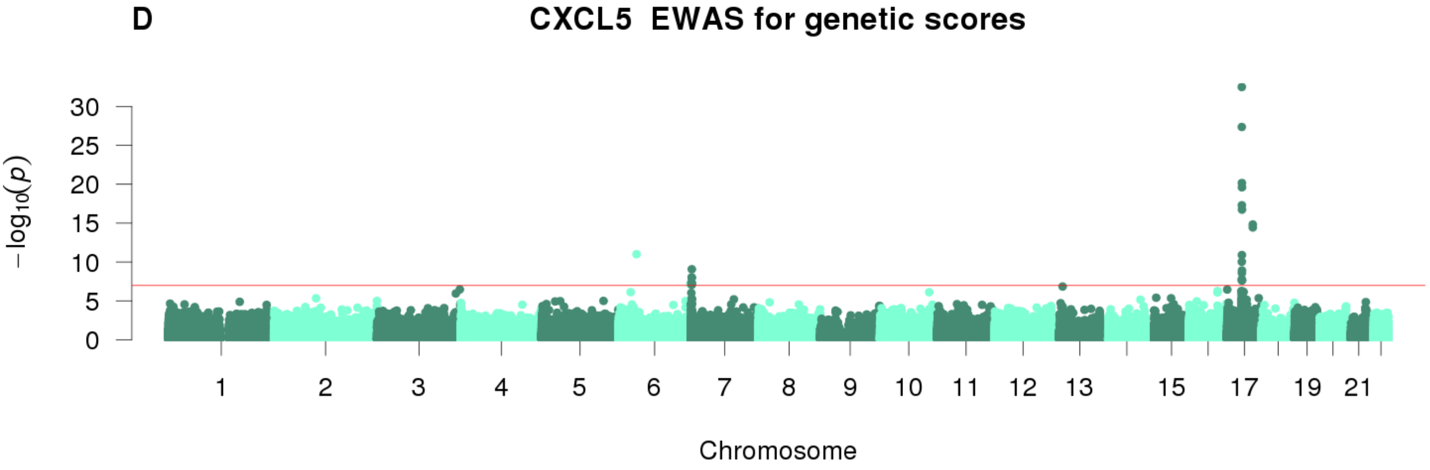
D
CXCL5 EWAS for genetic scores
−log10(p)
Chromosome

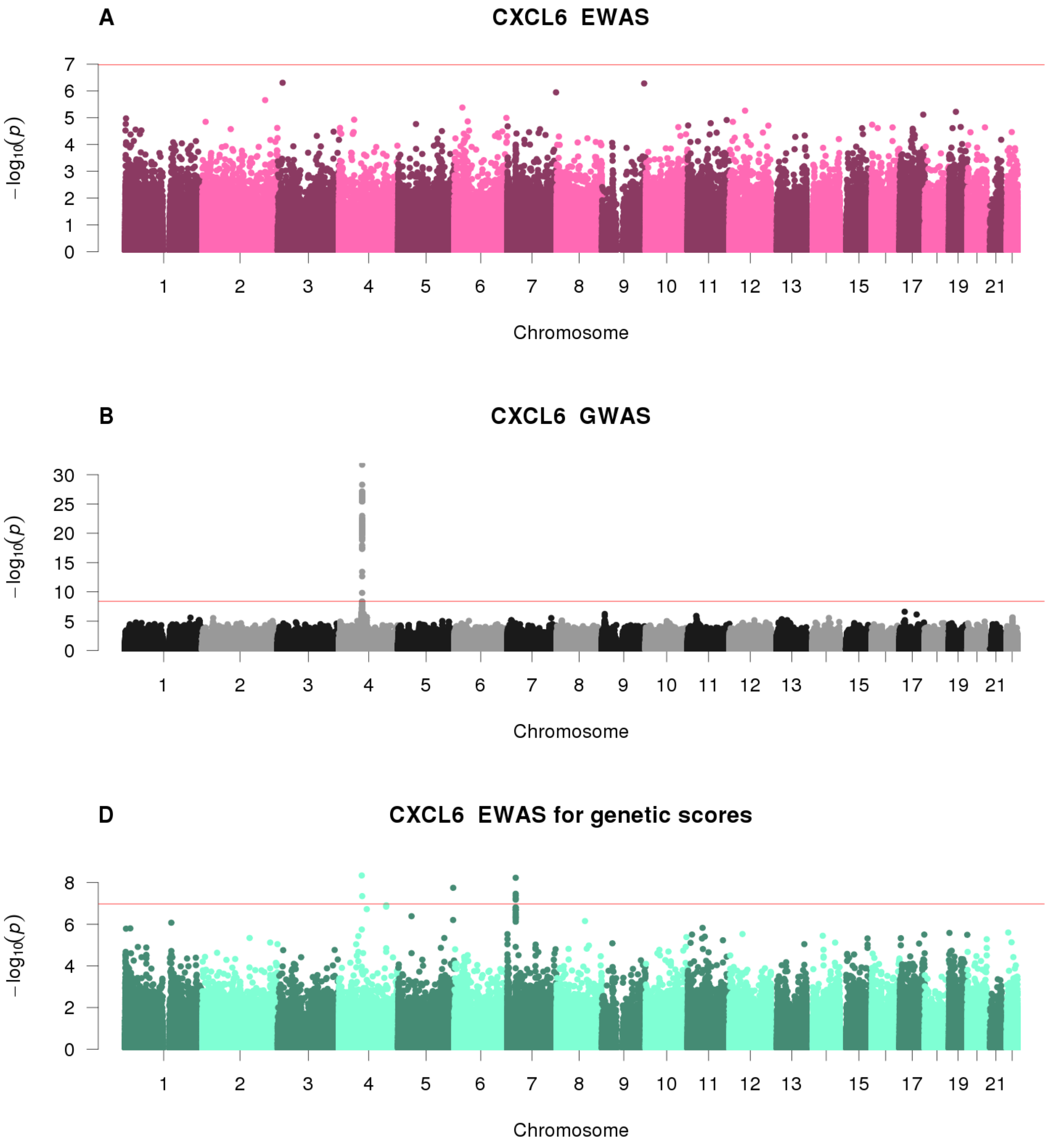

A
CXCL6 EWAS
−log10(p)
Chromosome
B
CXCL6 GWAS
−log10(p)
Chromosome
D
CXCL6 EWAS for genetic scores
−log10(p)
Chromosome

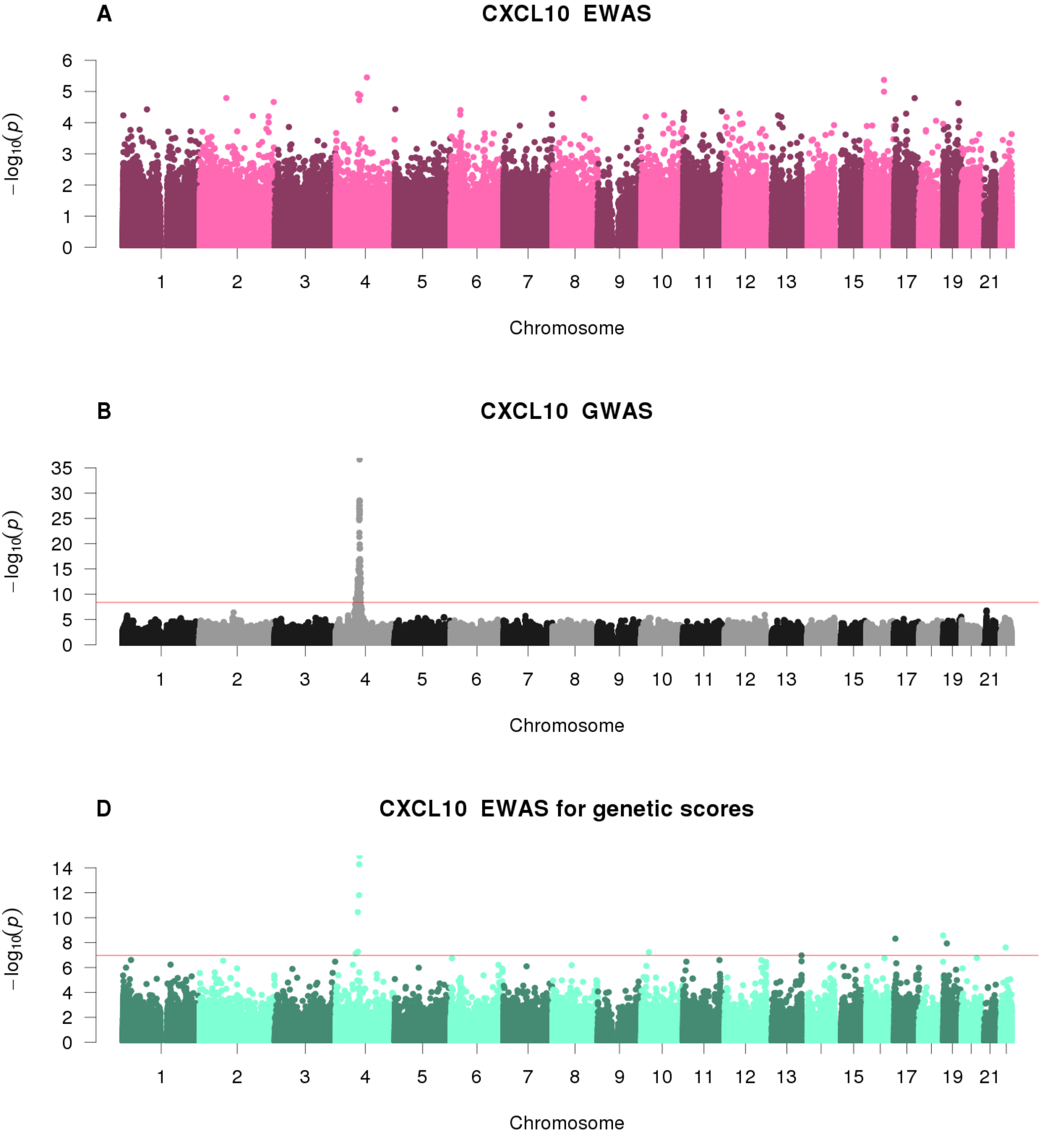
A
CXCL10 EWAS
−log10(p)
0
1
2
3
4
5
6
1 2 3 4 5 6 7 8 9 10 11 12 13 15 17 19 21
Chromosome
B
CXCL10 GWAS
−log10(p)
0
5
10
15
20
25
30
35
1 2 3 4 5 6 7 8 9 10 11 12 13 15 17 19 21
Chromosome
D
CXCL10 EWAS for genetic scores
−log10(p)
0
2
4
6
8
10
12
14
1 2 3 4 5 6 7 8 9 10 11 12 13 15 17 19 21
Chromosome

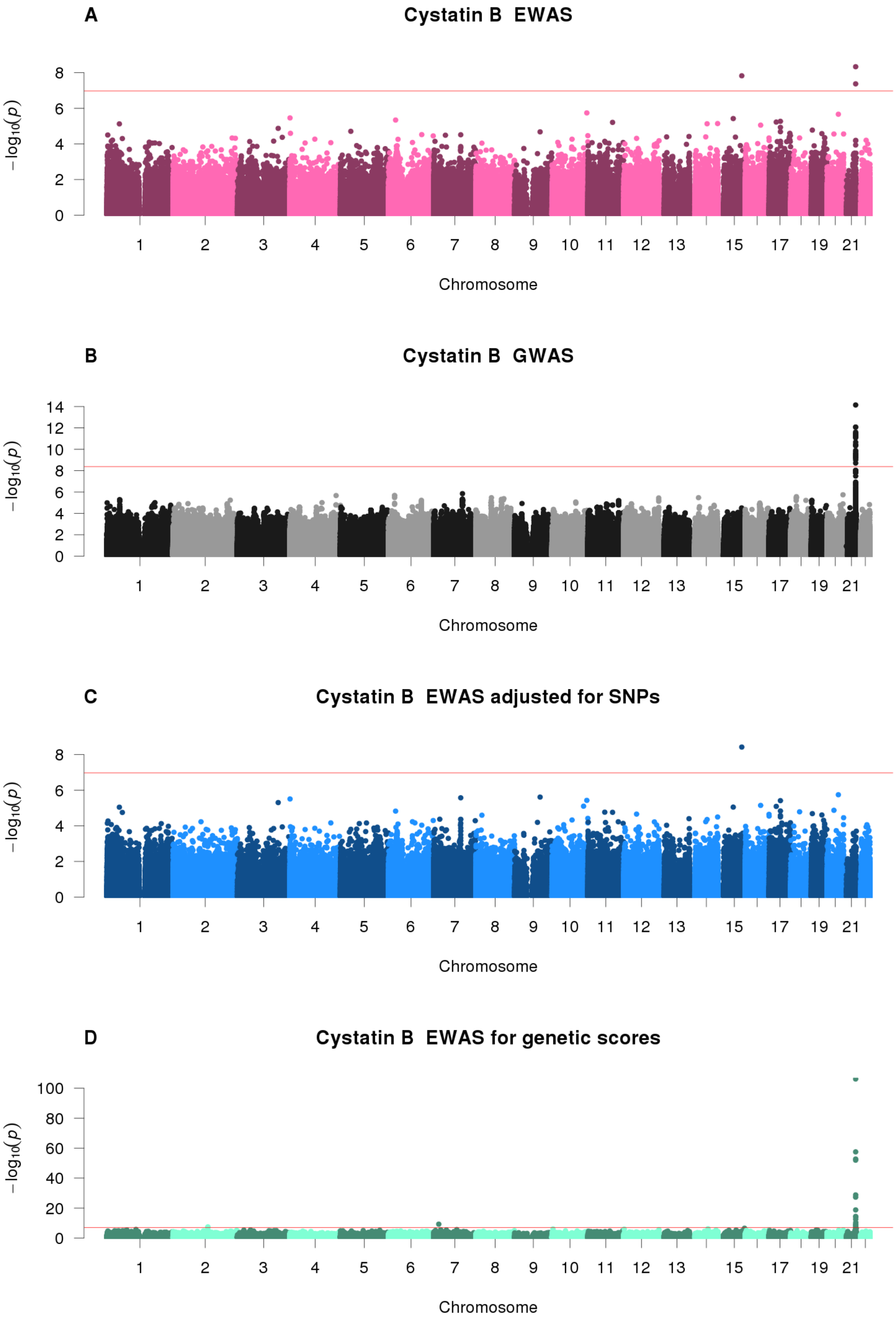

A
Cystatin B EWAS
B
Cystatin B GWAS
C
Cystatin B EWAS adjusted for SNPs
D
Cystatin B EWAS for genetic scores
−log10(p)
Chromosome
1
2
3
4
5
6
7
8
9
10
11
12
13
15
17
19
21

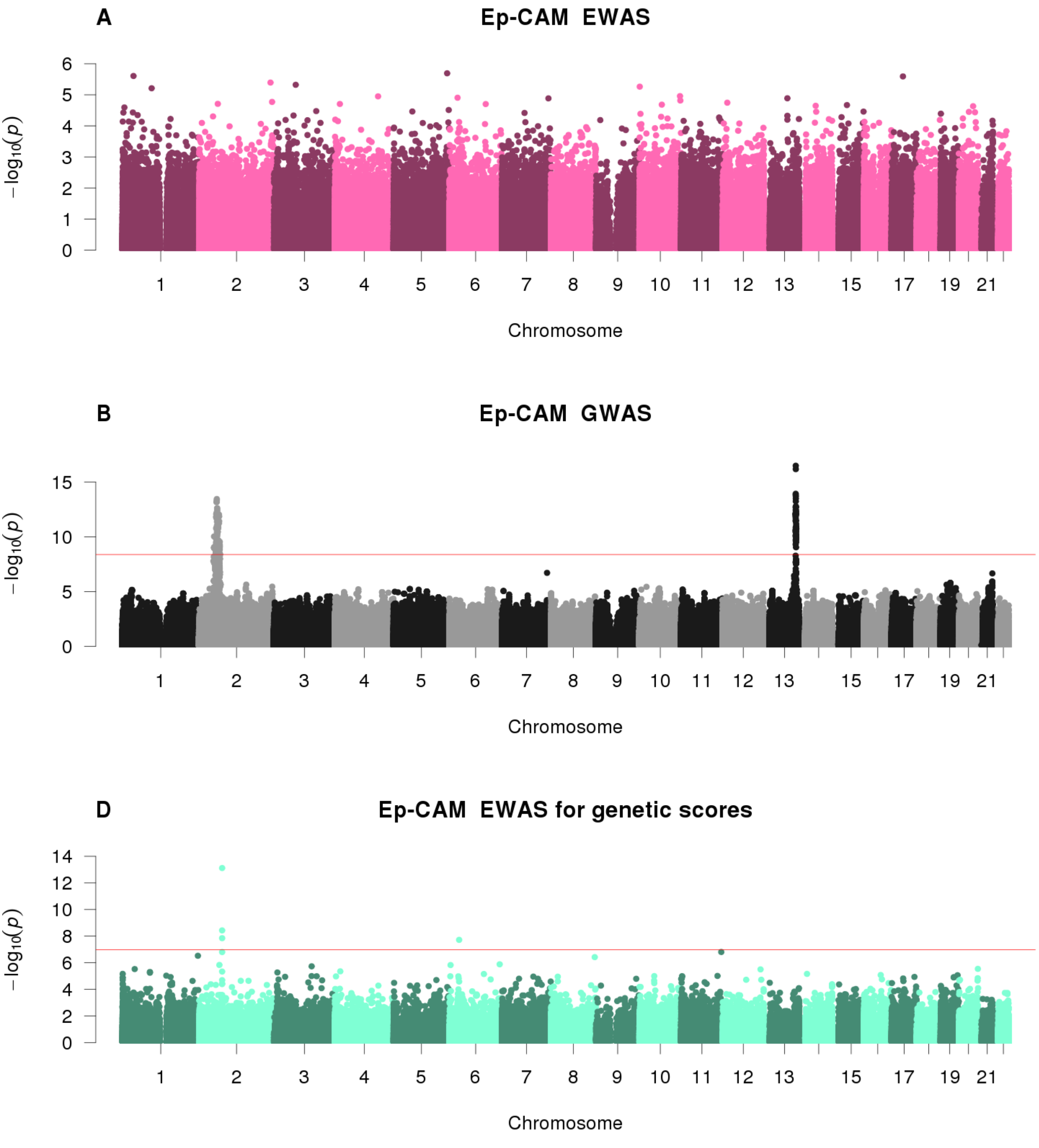

A
Ep-CAM EWAS
$-\log_{10}(p)$
Chromosome
B
Ep-CAM GWAS
$-\log_{10}(p)$
Chromosome
D
Ep-CAM EWAS for genetic scores
$-\log_{10}(p)$
Chromosome

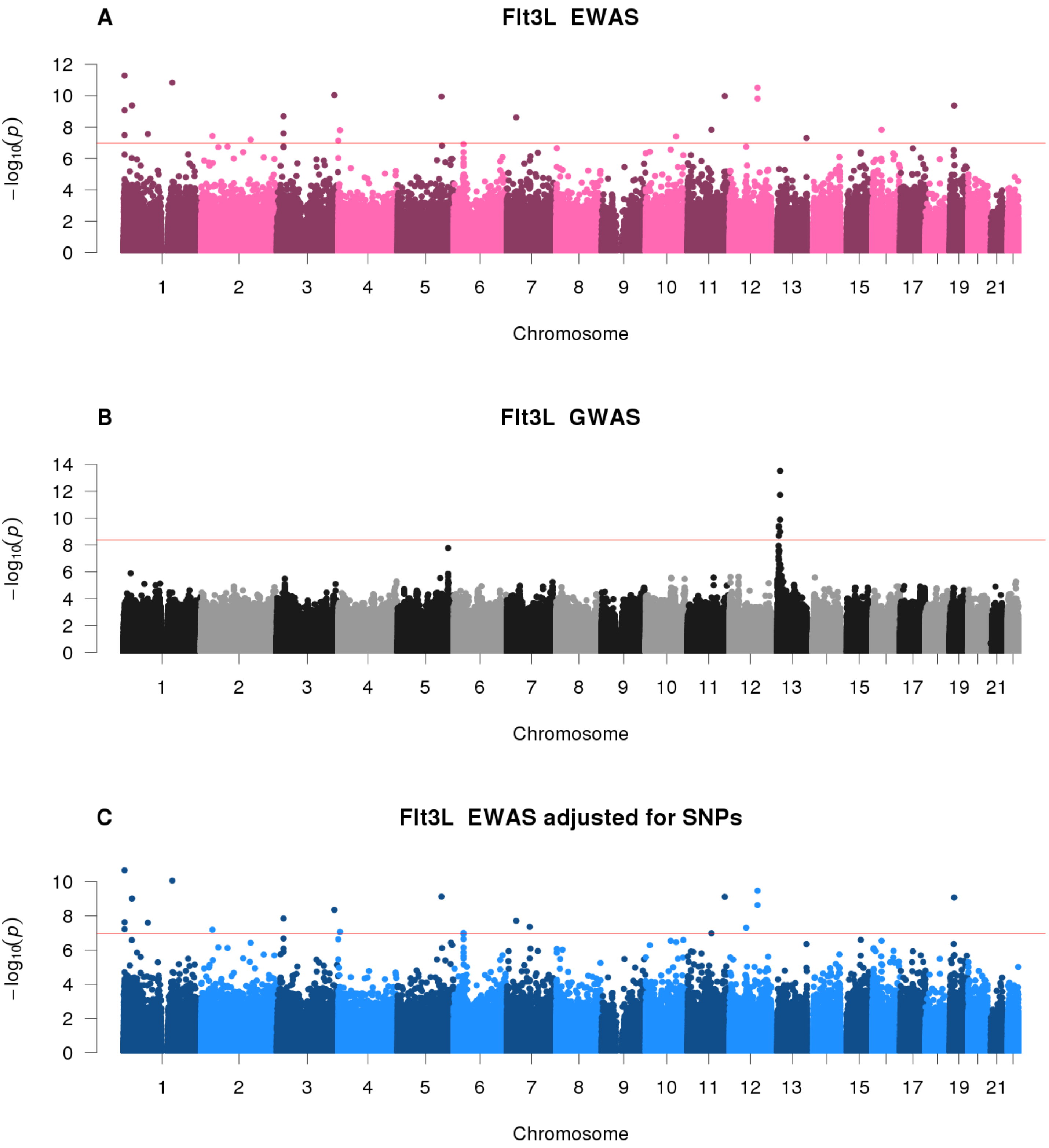
A
Flt3L EWAS
−log10(p)
0
2
4
6
8
10
12
Chromosome
1
2
3
4
5
6
7
8
9
10
11
12
13
15
17
19
21
B
Flt3L GWAS
−log10(p)
0
2
4
6
8
10
12
14
Chromosome
1
2
3
4
5
6
7
8
9
10
11
12
13
15
17
19
21
C
Flt3L EWAS adjusted for SNPs
−log10(p)
0
2
4
6
8
10
Chromosome
1
2
3
4
5
6
7
8
9
10
11
12
13
15
17
19
21

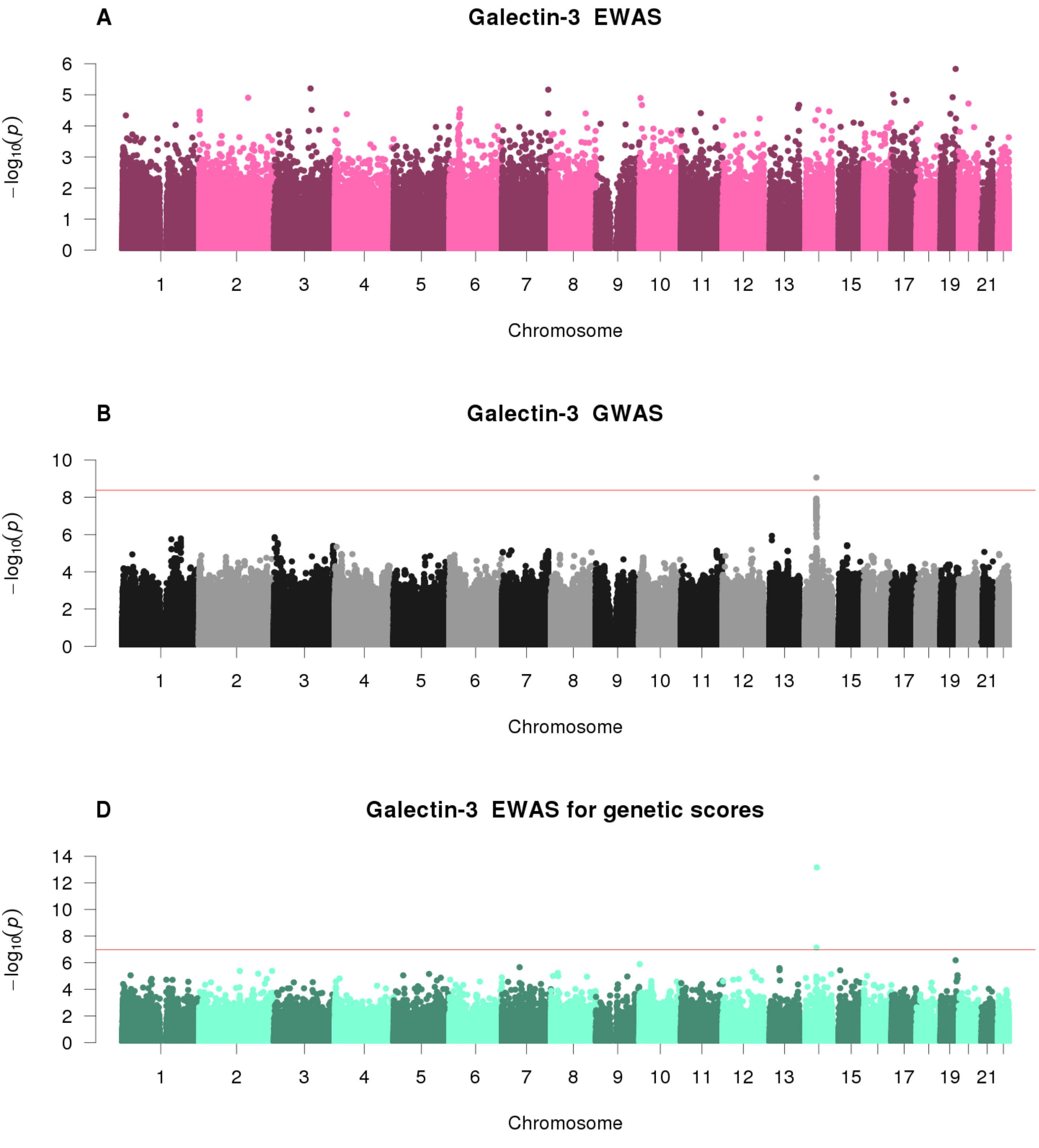
A
Galectin-3 EWAS
$-\log_{10}(p)$
Chromosome
B
Galectin-3 GWAS
$-\log_{10}(p)$
Chromosome
D
Galectin-3 EWAS for genetic scores
$-\log_{10}(p)$
Chromosome

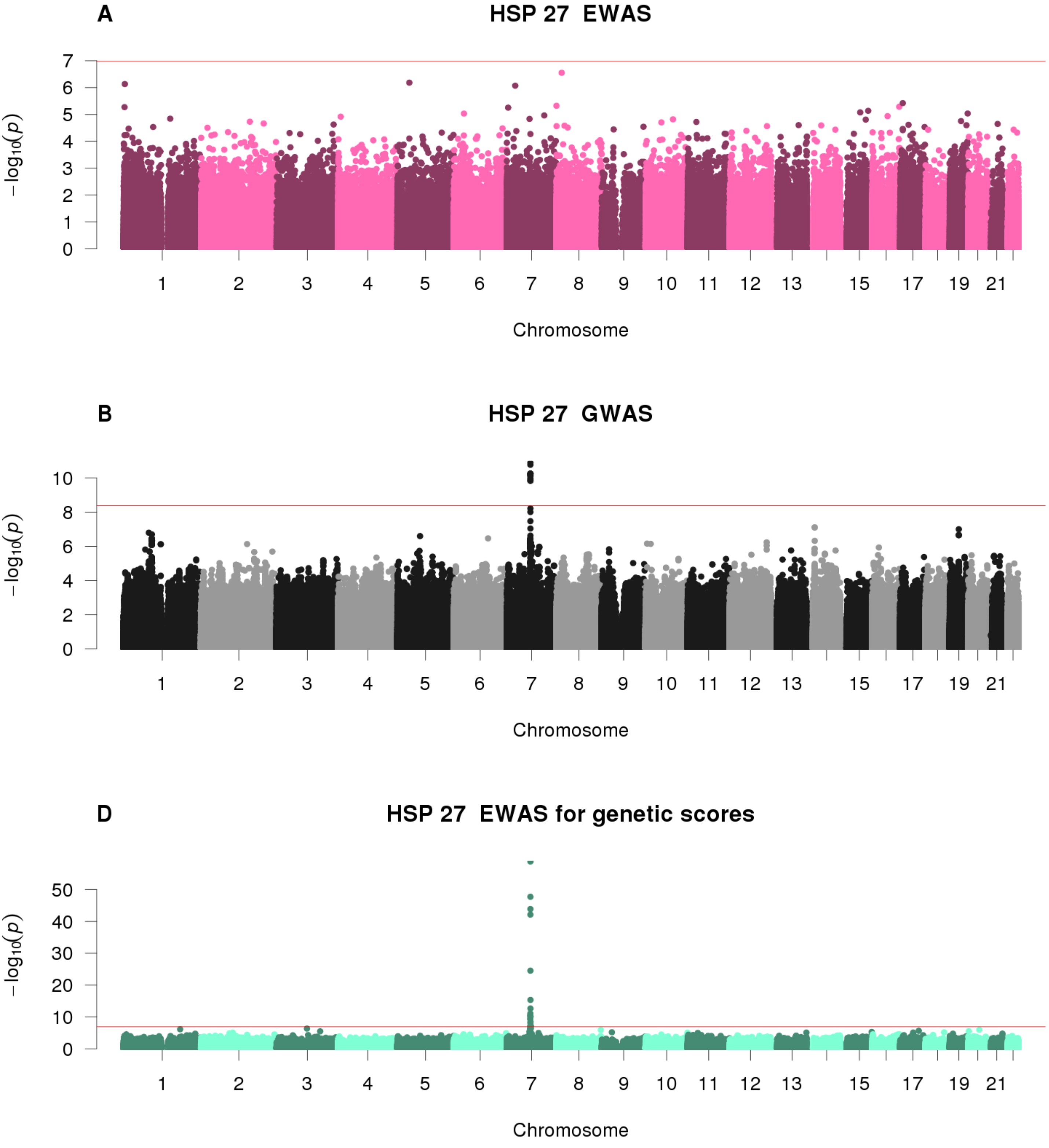
A
HSP 27 EWAS
−log10(p)
Chromosome
B
HSP 27 GWAS
−log10(p)
Chromosome
D
HSP 27 EWAS for genetic scores
−log10(p)
Chromosome

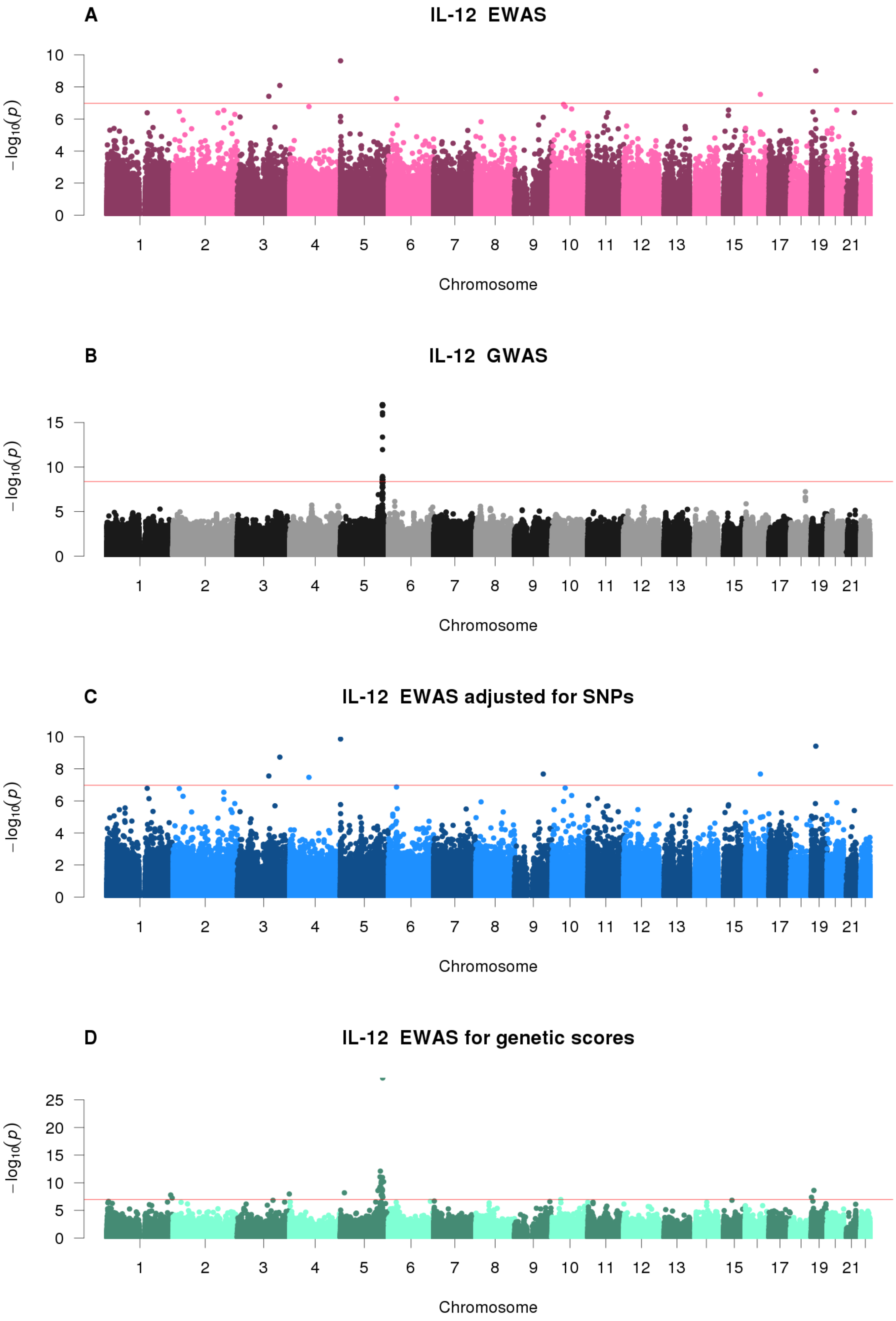
A
IL-12 EWAS
B
IL-12 GWAS
C
IL-12 EWAS adjusted for SNPs
D
IL-12 EWAS for genetic scores
−log10(p)
Chromosome
1
2
3
4
5
6
7
8
9
10
11
12
13
15
17
19
21

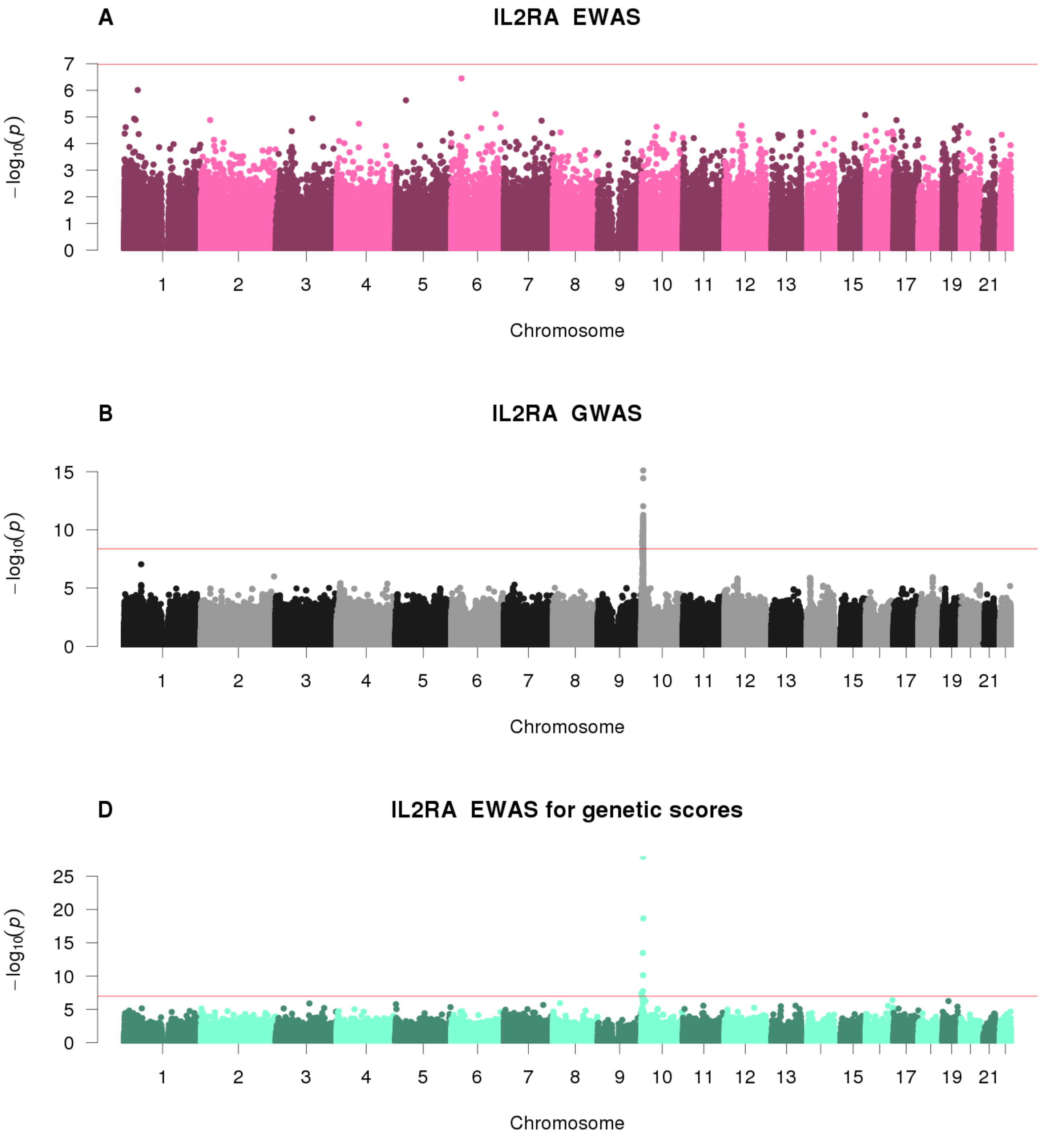
A
IL2RA EWAS
−log10(p)
Chromosome
B
IL2RA GWAS
−log10(p)
Chromosome
D
IL2RA EWAS for genetic scores
−log10(p)
Chromosome

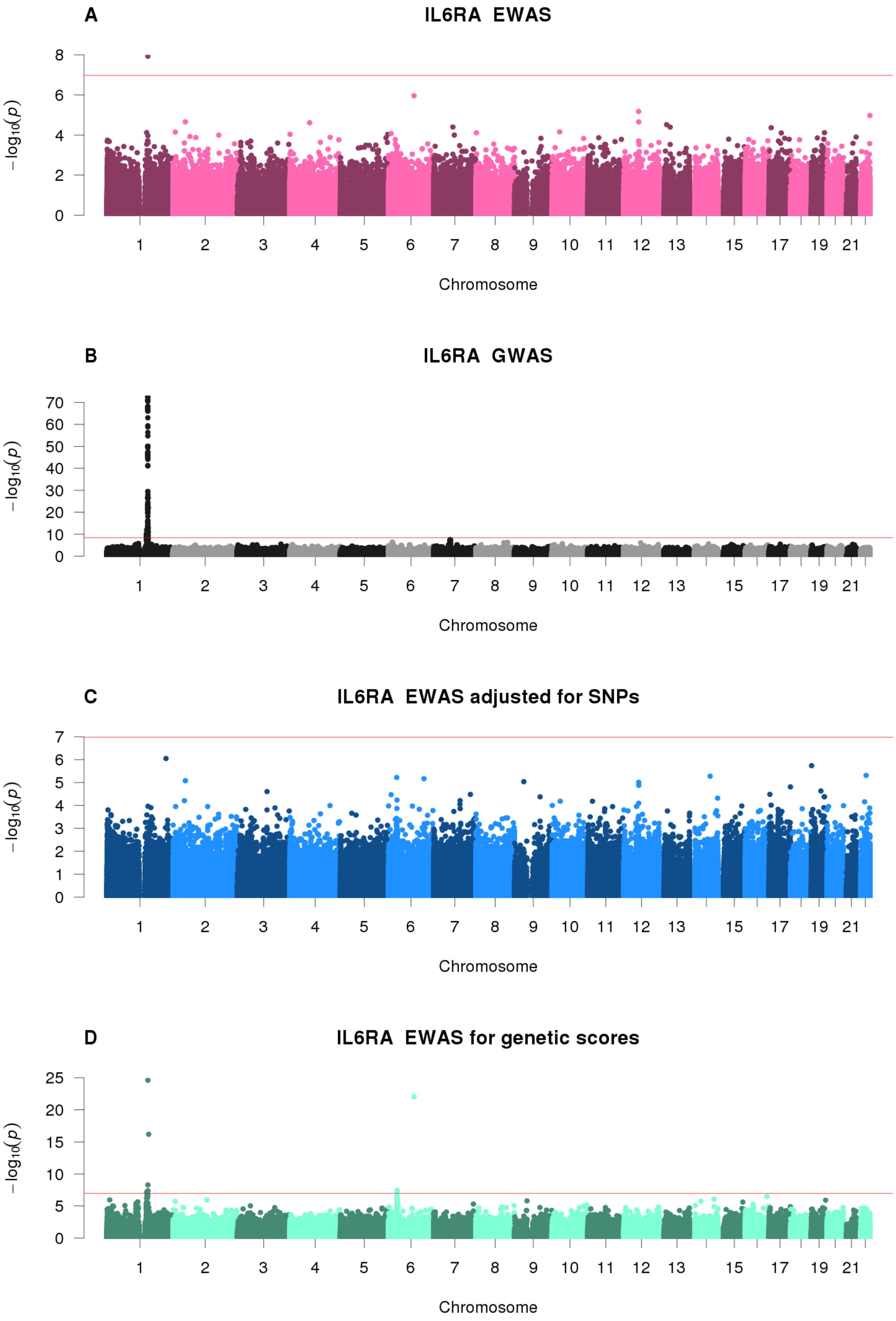

A
IL6RA EWAS
B
IL6RA GWAS
C
IL6RA EWAS adjusted for SNPs
D
IL6RA EWAS for genetic scores
−log10(p)
Chromosome
1
2
3
4
5
6
7
8
9
10
11
12
13
15
17
19
21

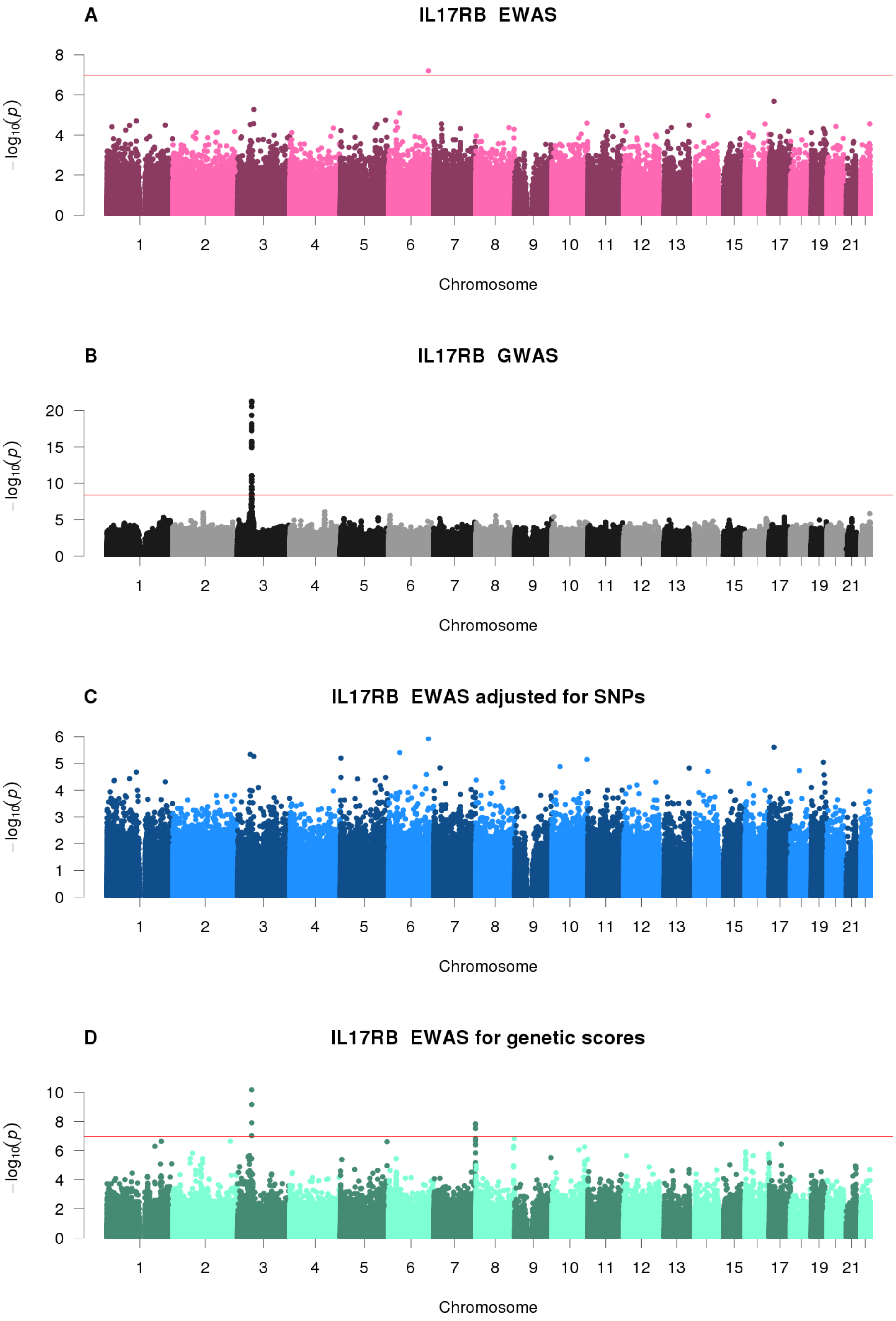

A
IL17RB EWAS
B
IL17RB GWAS
C
IL17RB EWAS adjusted for SNPs
D
IL17RB EWAS for genetic scores
$-\log_{10}(p)$
Chromosome
1 2 3 4 5 6 7 8 9 10 11 12 13 15 17 19 21

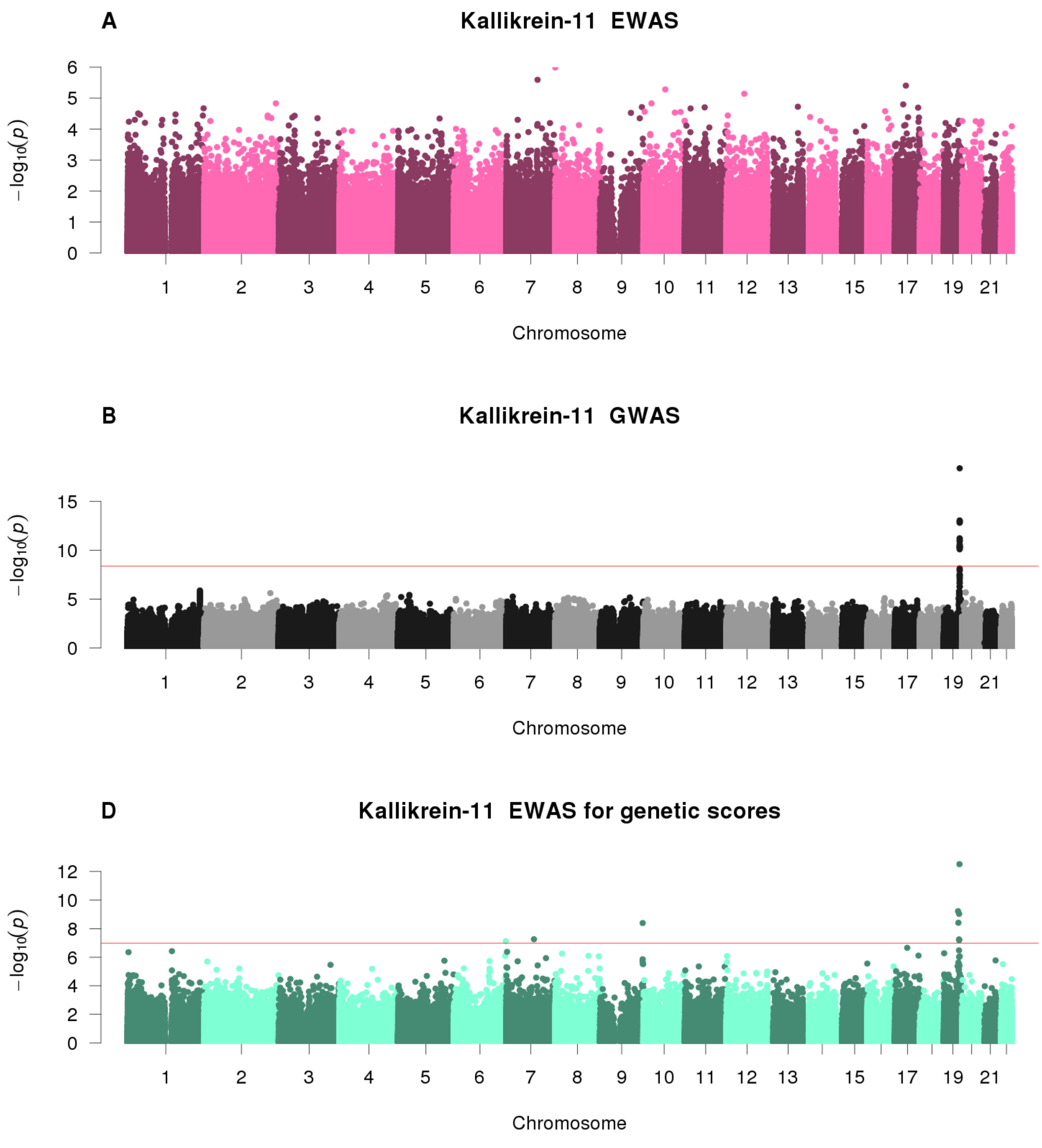
A
Kallikrein-11 EWAS
−log10(p)
Chromosome
B
Kallikrein-11 GWAS
−log10(p)
Chromosome
D
Kallikrein-11 EWAS for genetic scores
−log10(p)
Chromosome

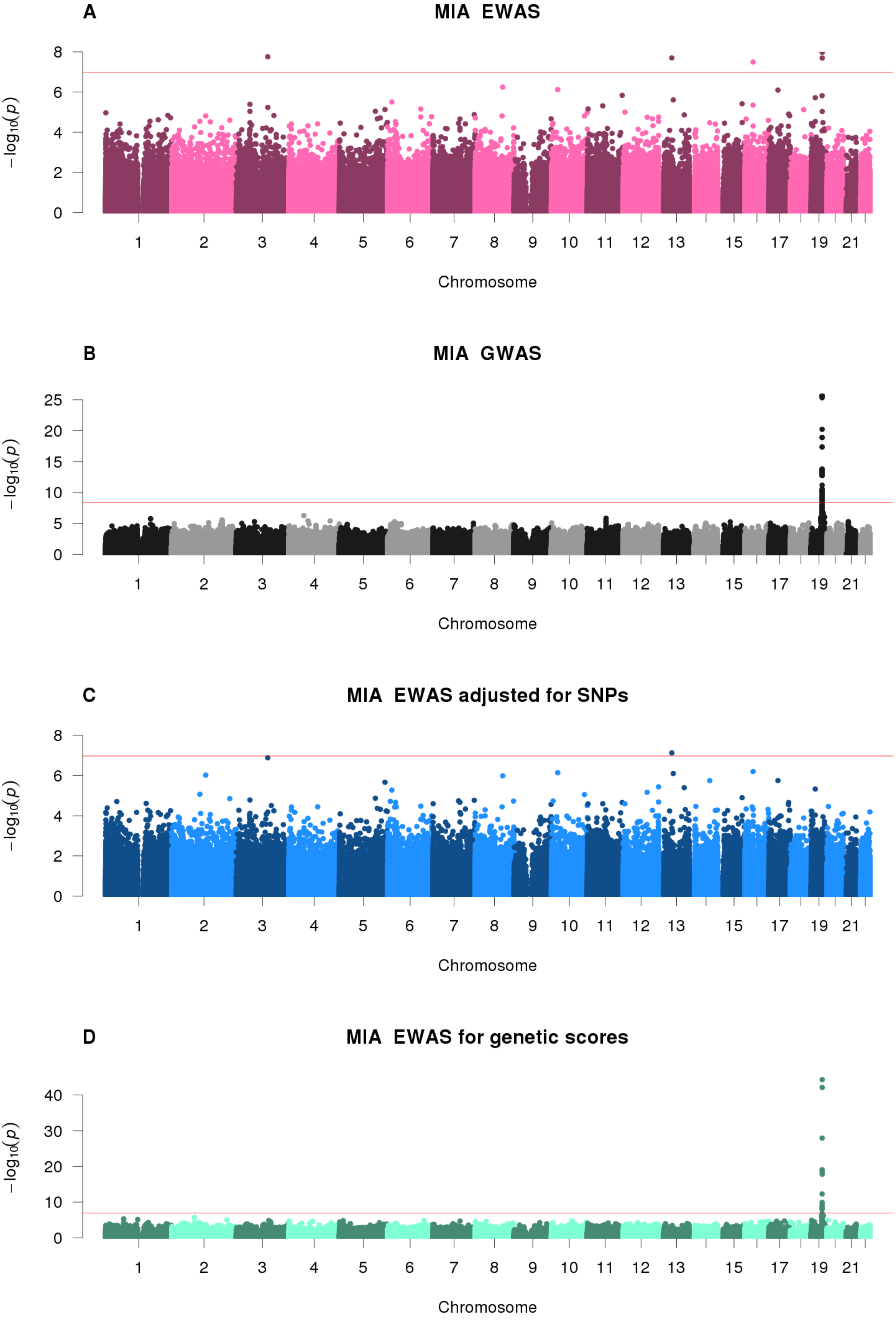
A
MIA EWAS
−log10(p)
0
2
4
6
8
Chromosome
1
2
3
4
5
6
7
8
9
10
11
12
13
15
17
19
21
B
MIA GWAS
−log10(p)
0
5
10
15
20
25
Chromosome
C
MIA EWAS adjusted for SNPs
−log10(p)
Chromosome
D
MIA EWAS for genetic scores
−log10(p)
0
10
20
30
40
Chromosome

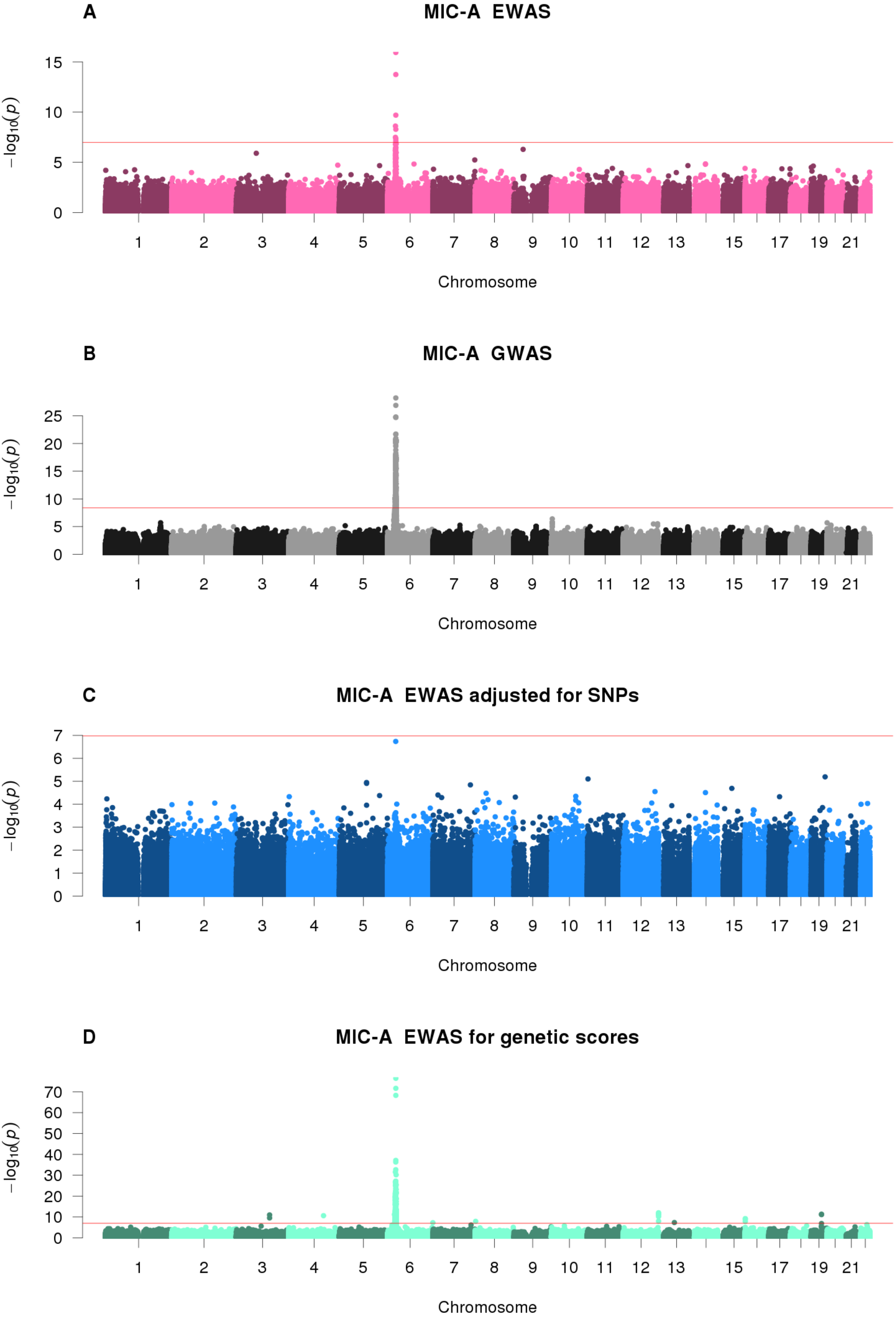
A
MIC-A EWAS
B
MIC-A GWAS
C
MIC-A EWAS adjusted for SNPs
D
MIC-A EWAS for genetic scores
$-\log_{10}(p)$
Chromosome
1
2
3
4
5
6
7
8
9
10
11
12
13
15
17
19
21

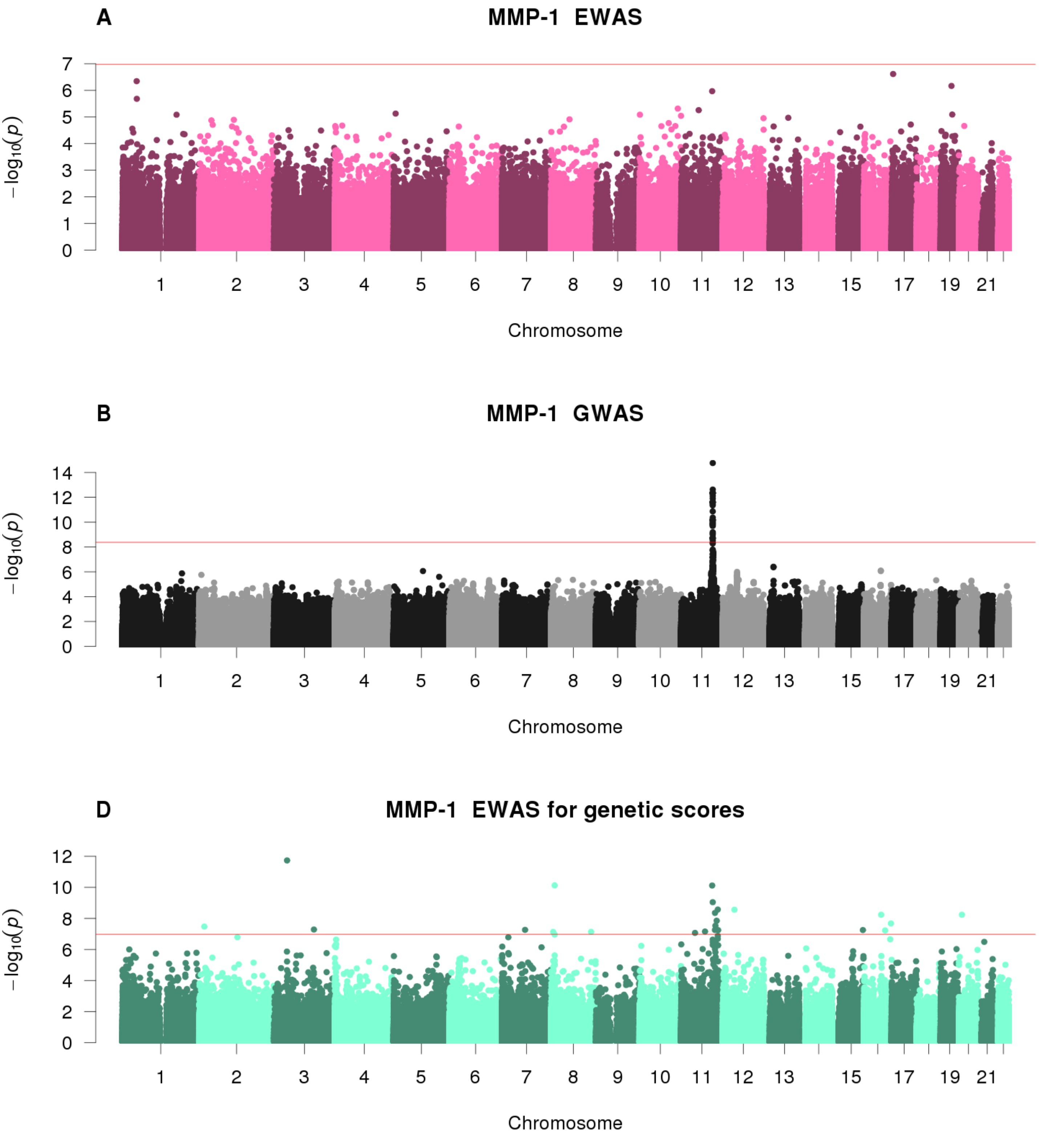

A
MMP-1 EWAS
$-\log_{10}(p)$
Chromosome
B
MMP-1 GWAS
$-\log_{10}(p)$
Chromosome
D
MMP-1 EWAS for genetic scores
$-\log_{10}(p)$
Chromosome

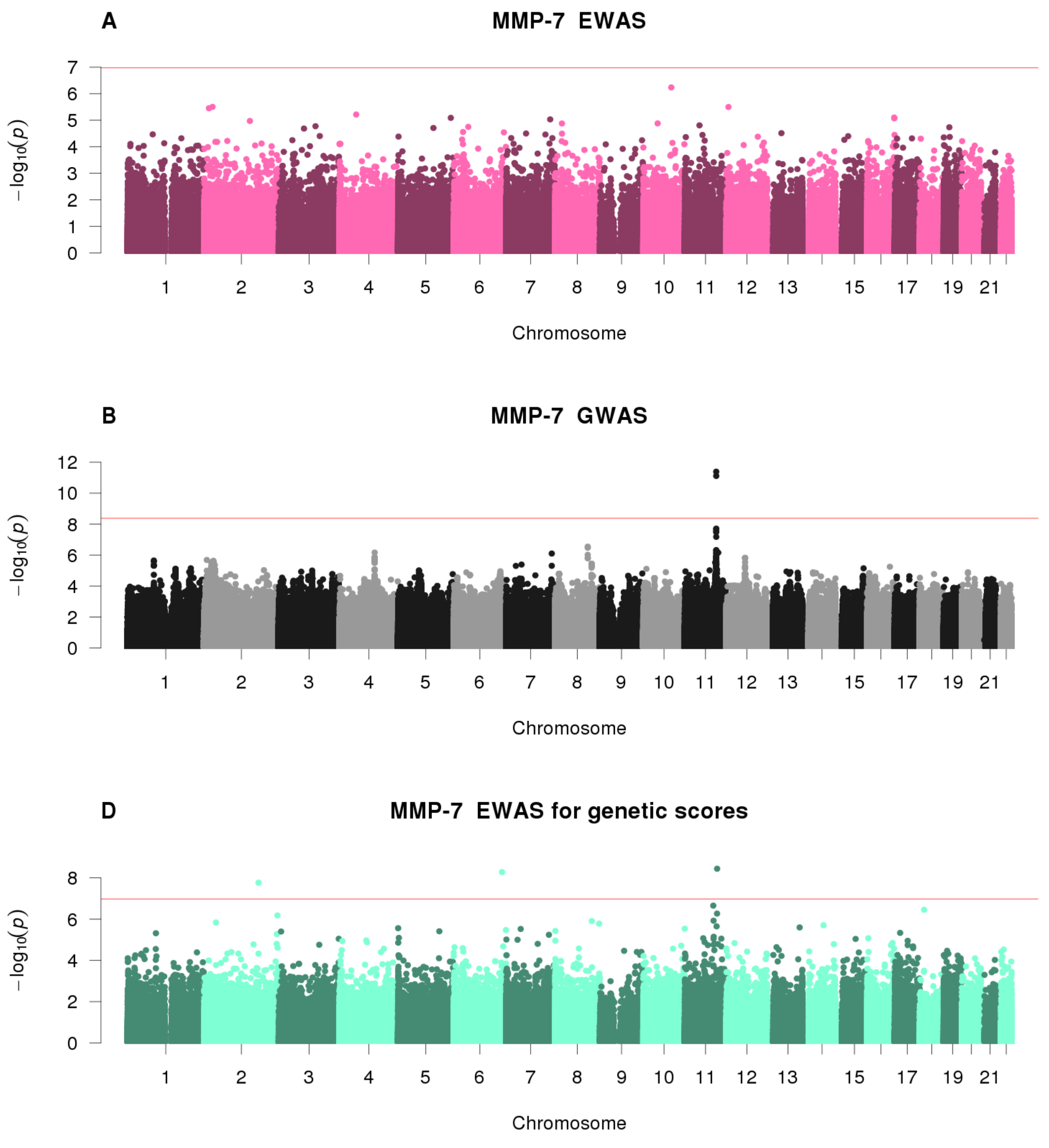
A
MMP-7 EWAS
$-\log_{10}(p)$
0 1 2 3 4 5 6 7
1 2 3 4 5 6 7 8 9 10 11 12 13 15 17 19 21
Chromosome
B
MMP-7 GWAS
$-\log_{10}(p)$
0 2 4 6 8 10 12
1 2 3 4 5 6 7 8 9 10 11 12 13 15 17 19 21
Chromosome
D
MMP-7 EWAS for genetic scores
$-\log_{10}(p)$
0 2 4 6 8
1 2 3 4 5 6 7 8 9 10 11 12 13 15 17 19 21
Chromosome

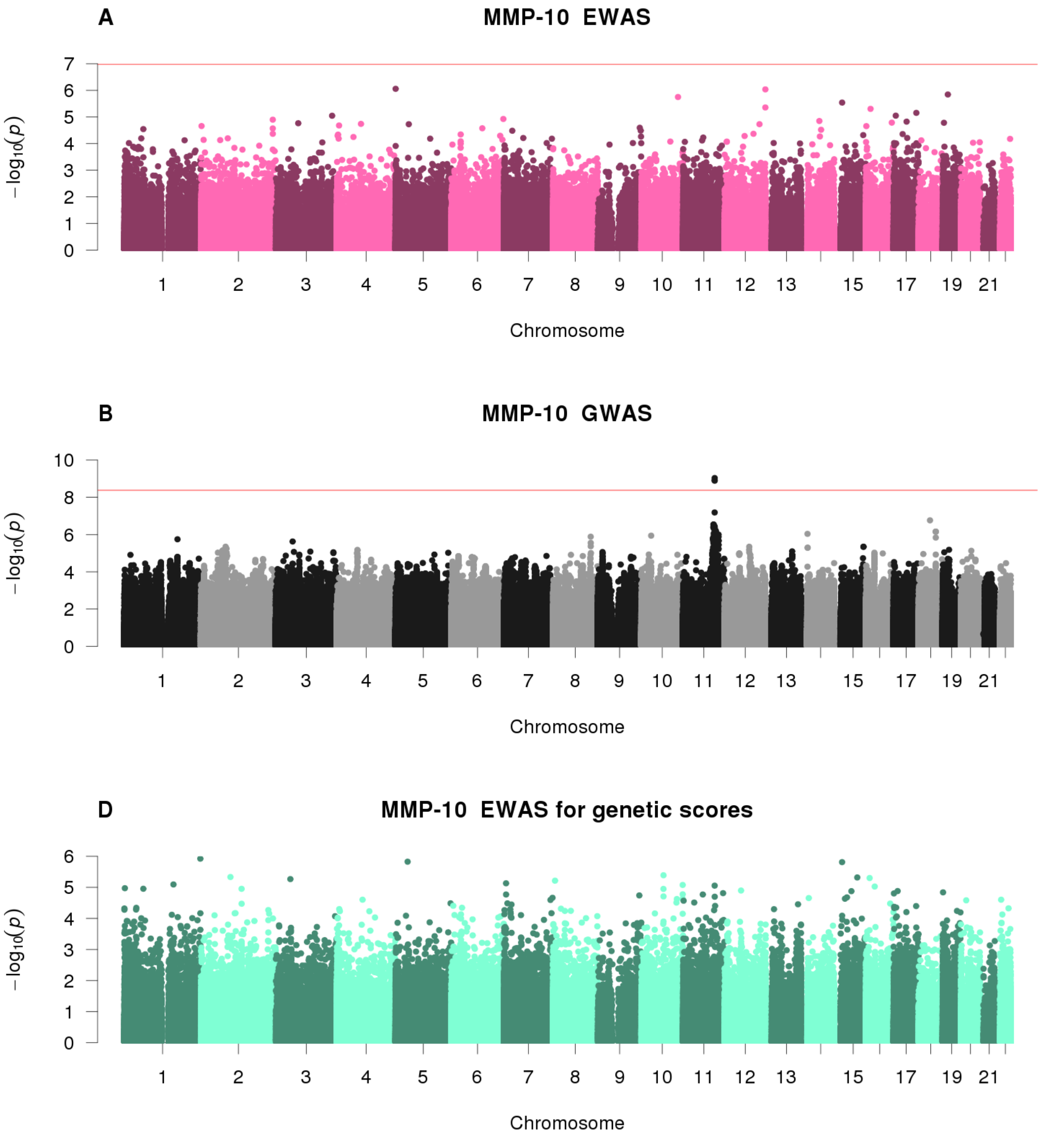
A
MMP-10 EWAS
B
MMP-10 GWAS
D
MMP-10 EWAS for genetic scores
−log10(p)
Chromosome
1
2
3
4
5
6
7
8
9
10
11
12
13
15
17
19
21

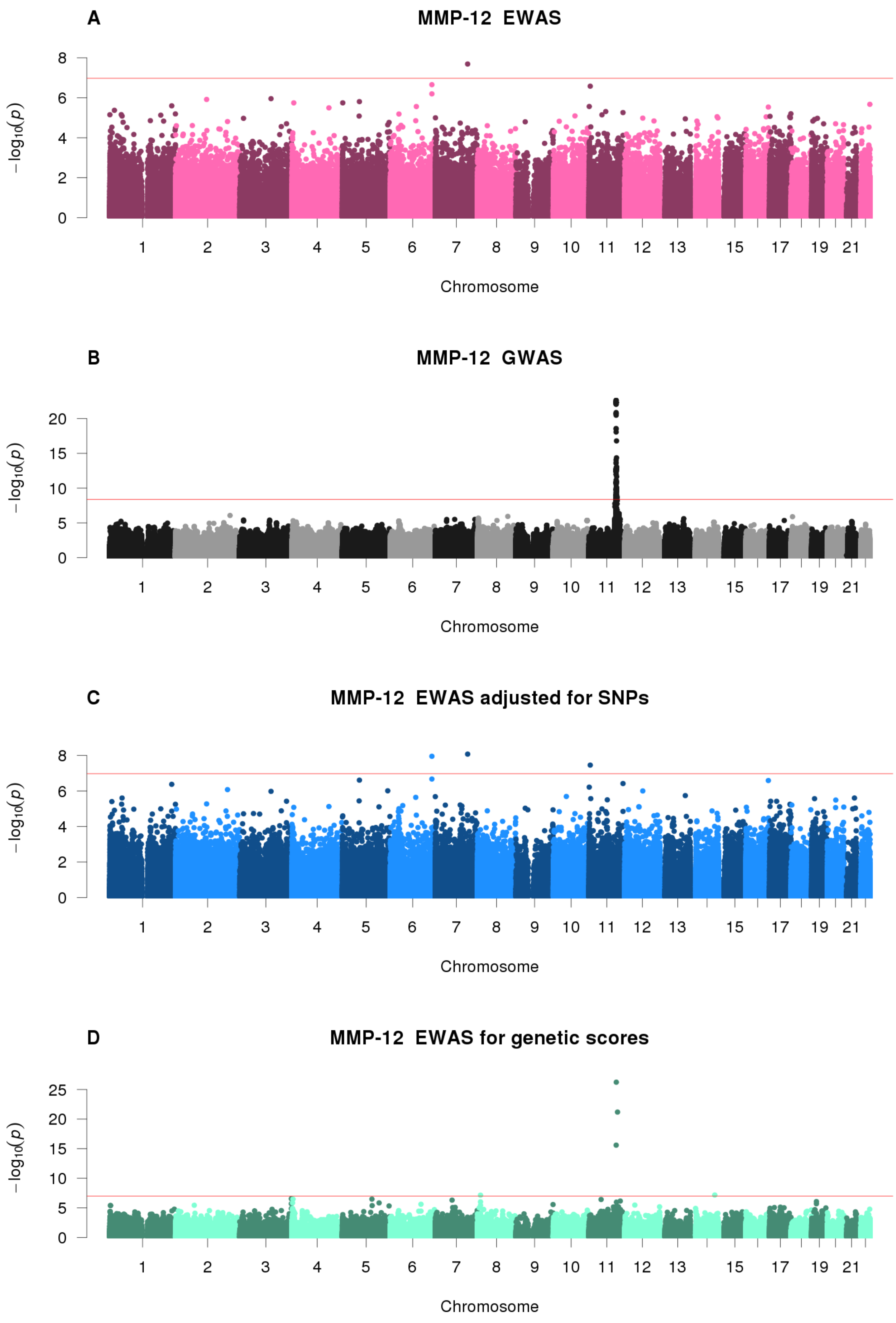
A
MMP-12 EWAS
B
MMP-12 GWAS
C
MMP-12 EWAS adjusted for SNPs
D
MMP-12 EWAS for genetic scores
−log10(p)
Chromosome
1
2
3
4
5
6
7
8
9
10
11
12
13
15
17
19
21

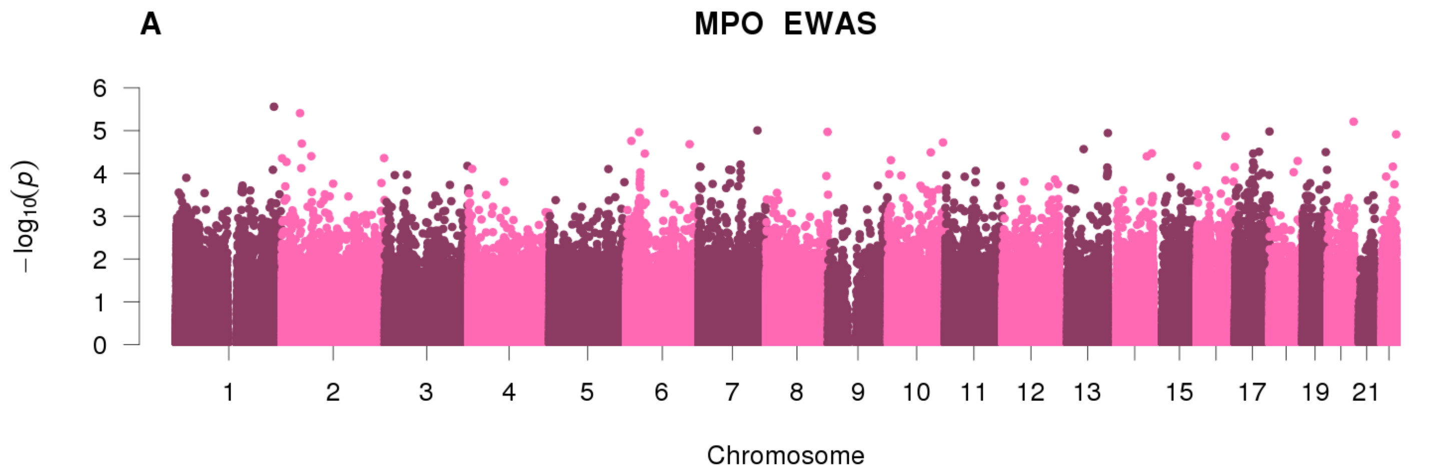
A
MPO EWAS
−log10(p)
Chromosome

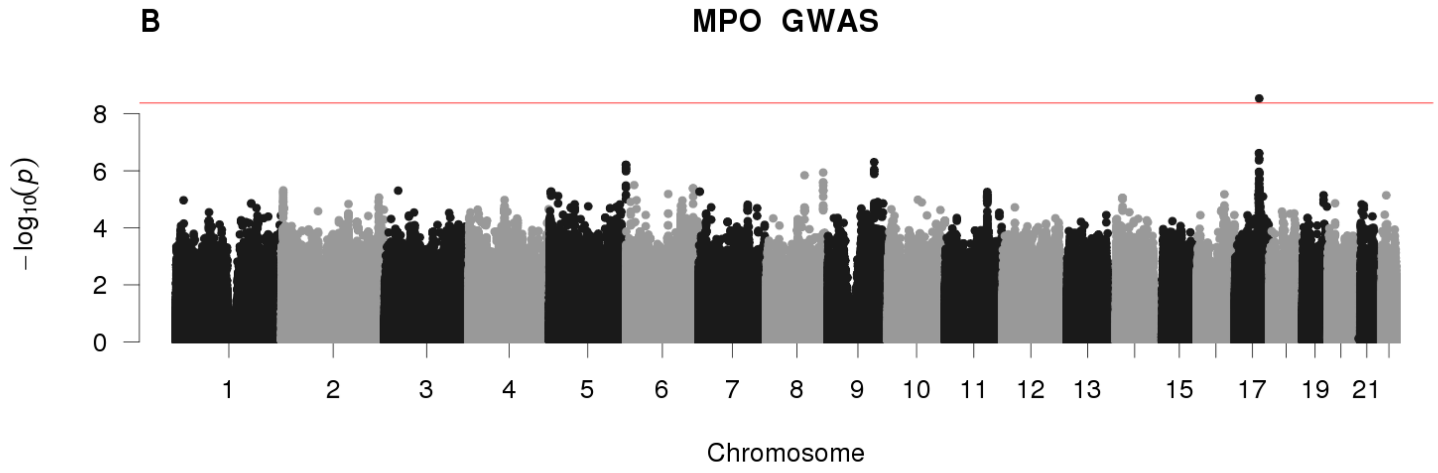
B
MPO GWAS
−log10(p)
Chromosome

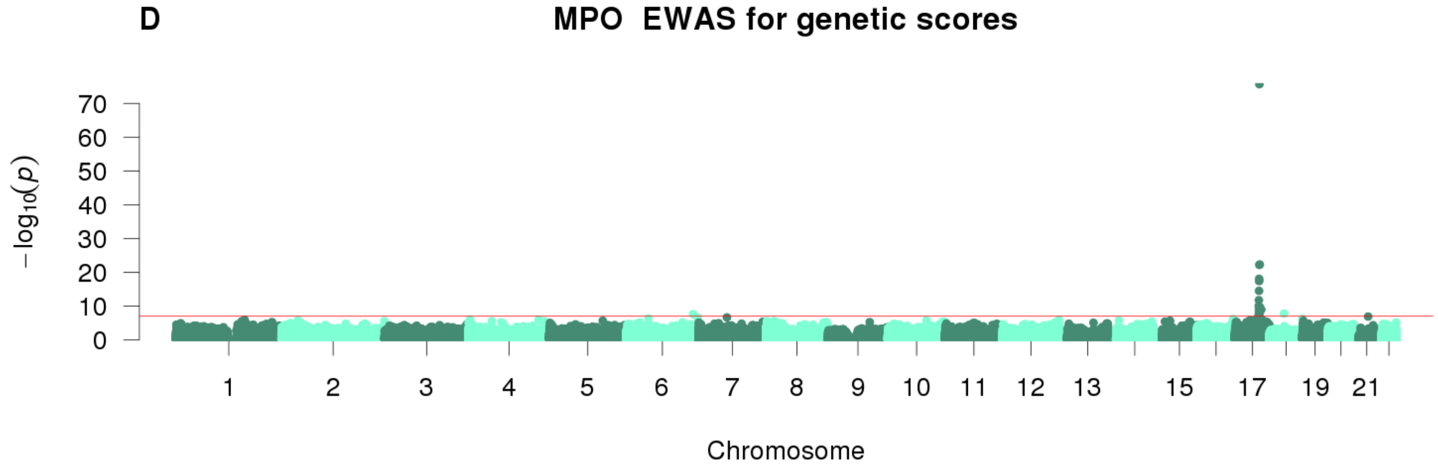
D
MPO EWAS for genetic scores
−log10(p)
Chromosome

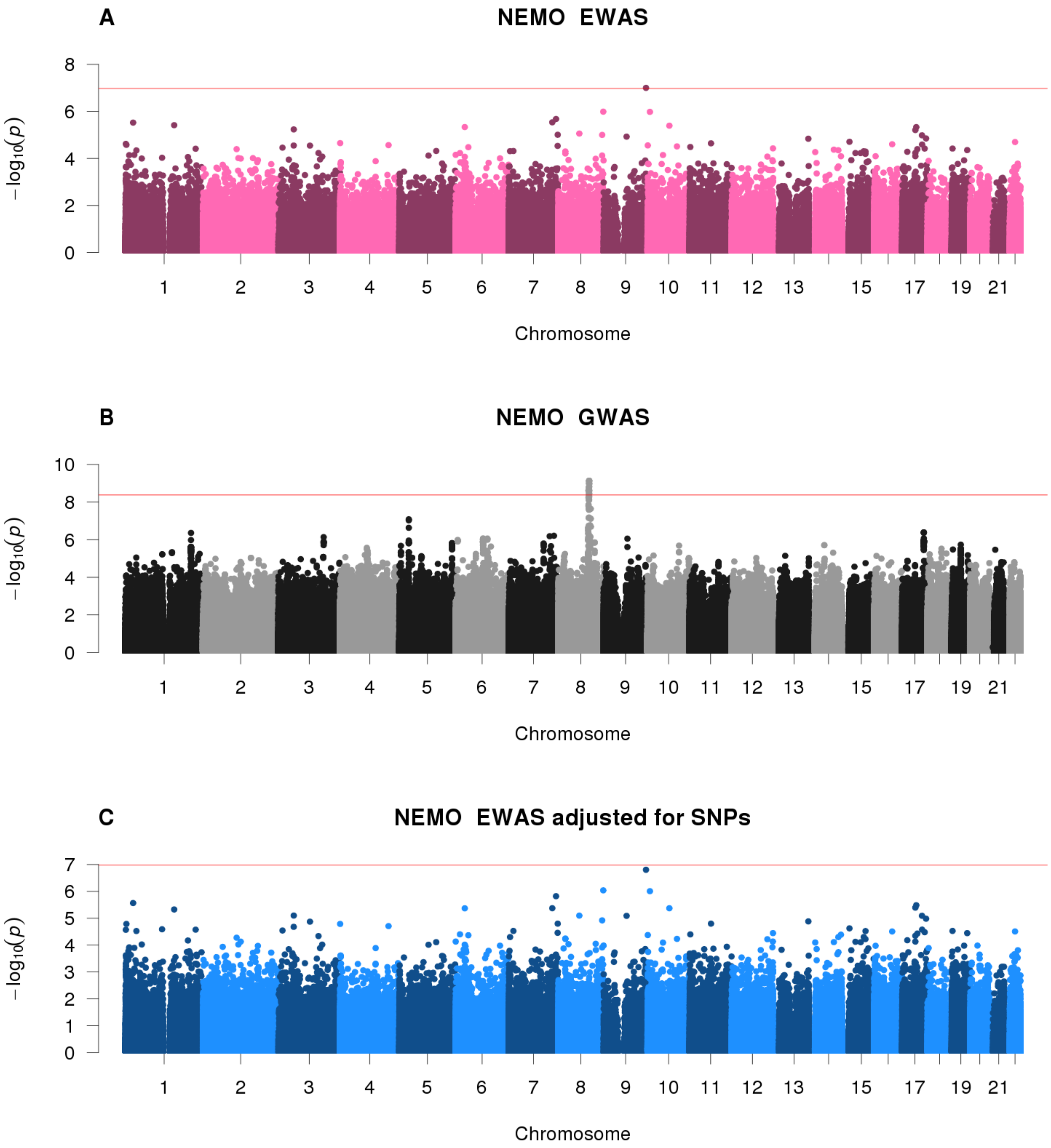

A
NEMO EWAS
8
6
4
2
0
−log10(p)
1
2
3
4
5
6
7
8
9
10
11
12
13
15
17
19
21
Chromosome
B
NEMO GWAS
10
8
6
4
2
0
−log10(p)
1
2
3
4
5
6
7
8
9
10
11
12
13
15
17
19
21
Chromosome
C
NEMO EWAS adjusted for SNPs
7
6
5
4
3
2
1
0
−log10(p)
1
2
3
4
5
6
7
8
9
10
11
12
13
15
17
19
21
Chromosome

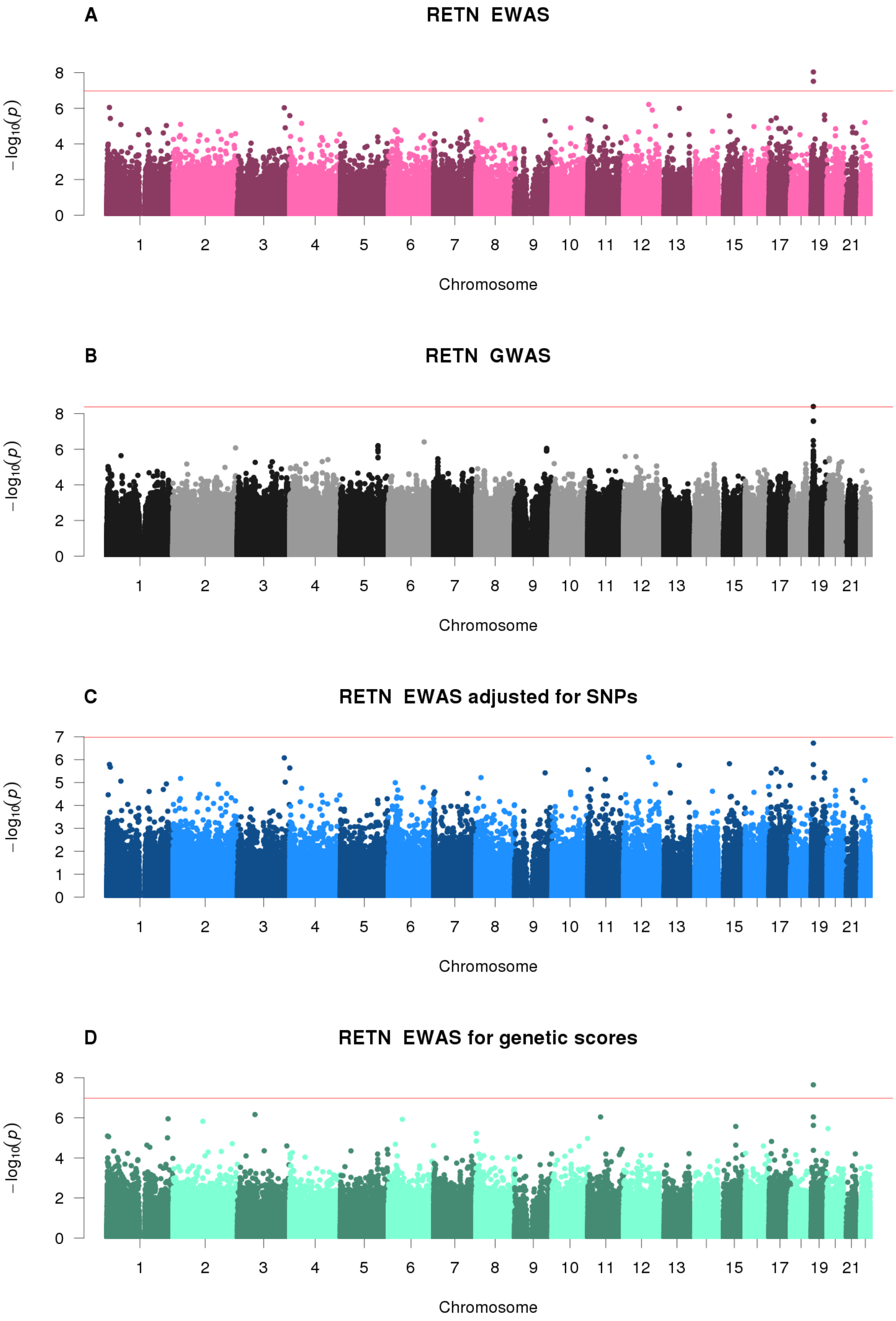
A
RETN EWAS
B
RETN GWAS
C
RETN EWAS adjusted for SNPs
D
RETN EWAS for genetic scores
$-\log_{10}(p)$
Chromosome
1
2
3
4
5
6
7
8
9
10
11
12
13
15
17
19
21

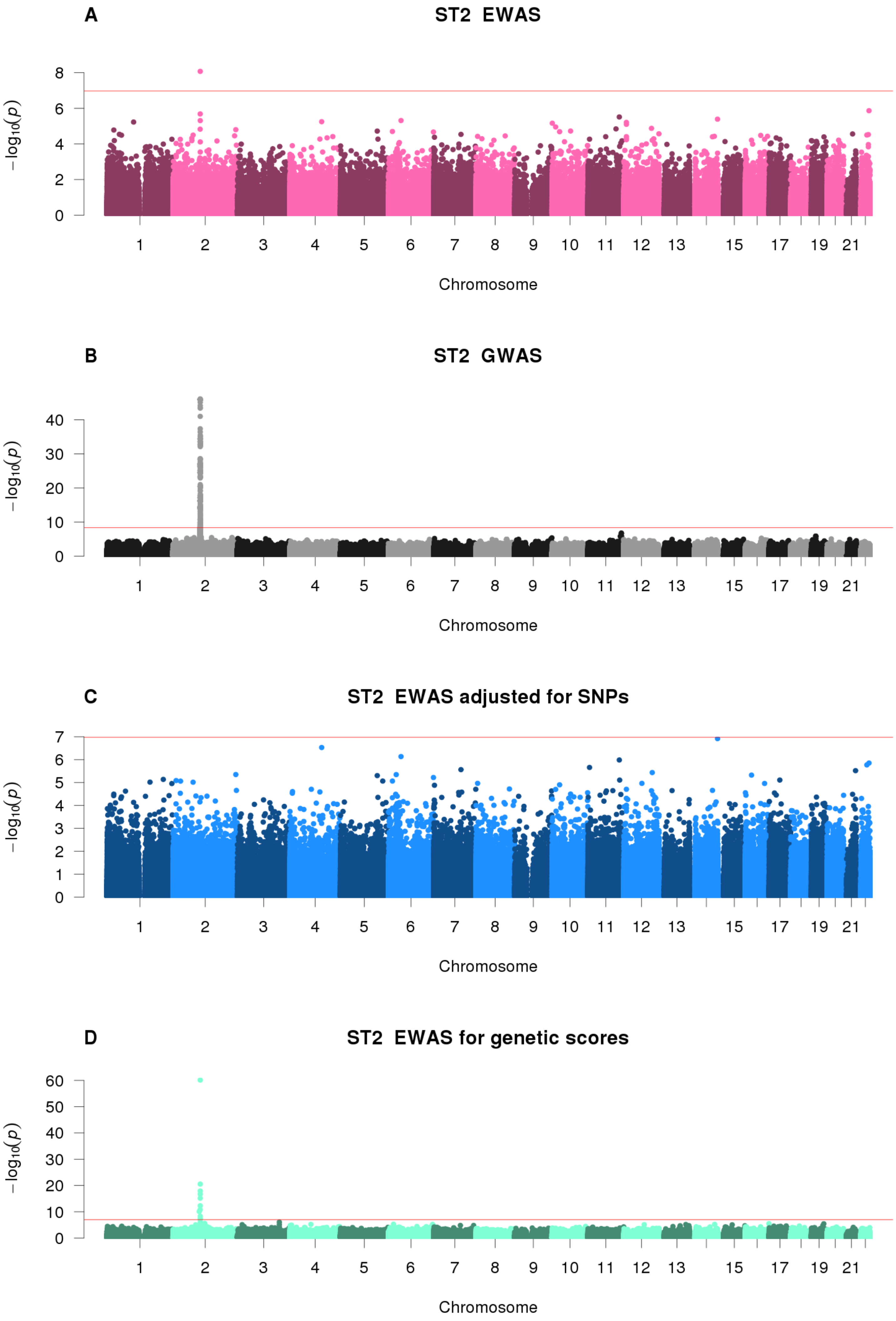
A
ST2 EWAS
−log10(p)
0
2
4
6
8
1 2 3 4 5 6 7 8 9 10 11 12 13 15 17 19 21
Chromosome
B
ST2 GWAS
−log10(p)
0
10
20
30
40
1 2 3 4 5 6 7 8 9 10 11 12 13 15 17 19 21
Chromosome
C
ST2 EWAS adjusted for SNPs
−log10(p)
0
1
2
3
4
5
6
7
1 2 3 4 5 6 7 8 9 10 11 12 13 15 17 19 21
Chromosome
D
ST2 EWAS for genetic scores
−log10(p)
0
10
20
30
40
50
60
1 2 3 4 5 6 7 8 9 10 11 12 13 15 17 19 21
Chromosome

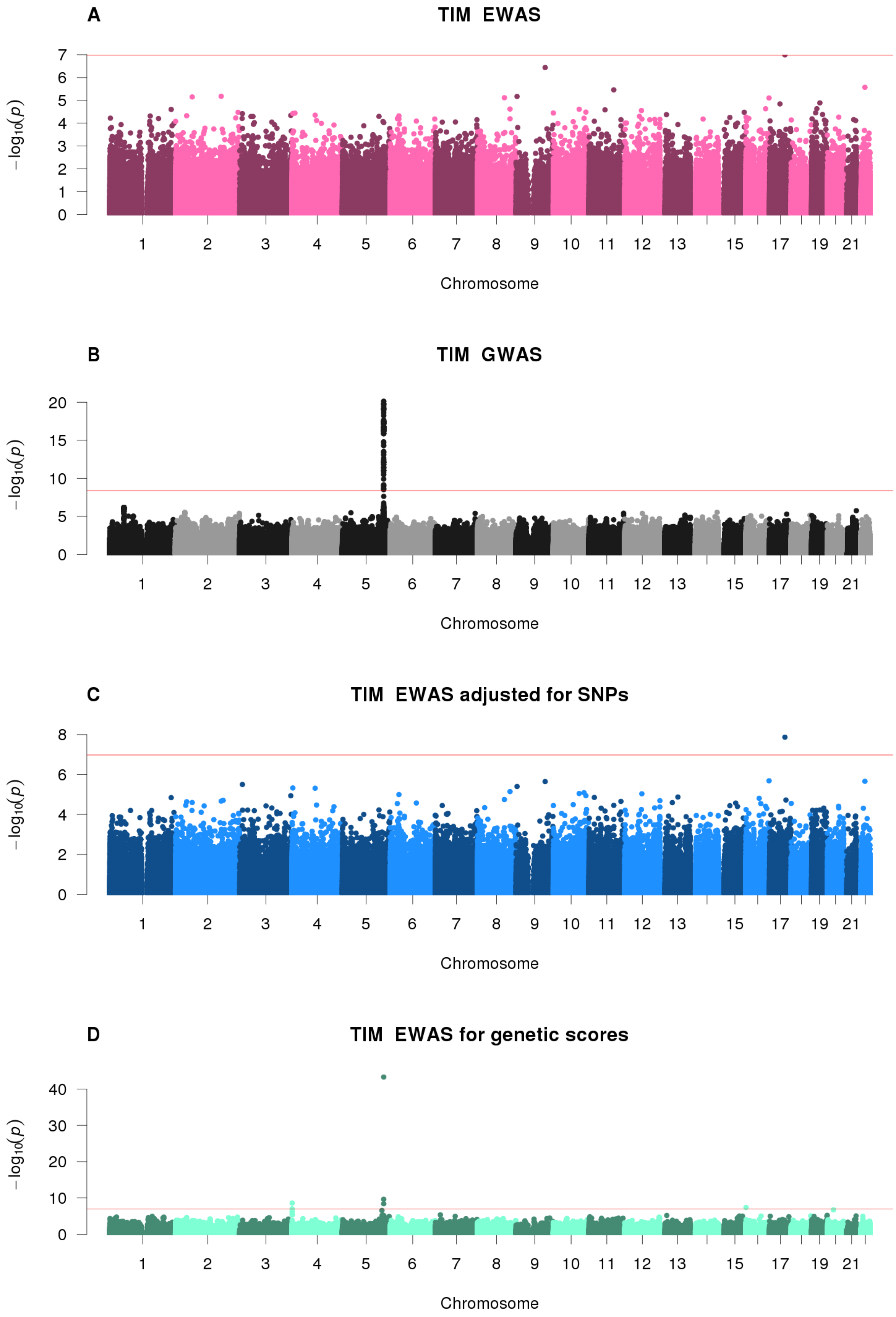
A
TIM EWAS
−log10(p)
Chromosome
B
TIM GWAS
−log10(p)
Chromosome
C
TIM EWAS adjusted for SNPs
−log10(p)
Chromosome
D
TIM EWAS for genetic scores
−log10(p)
Chromosome

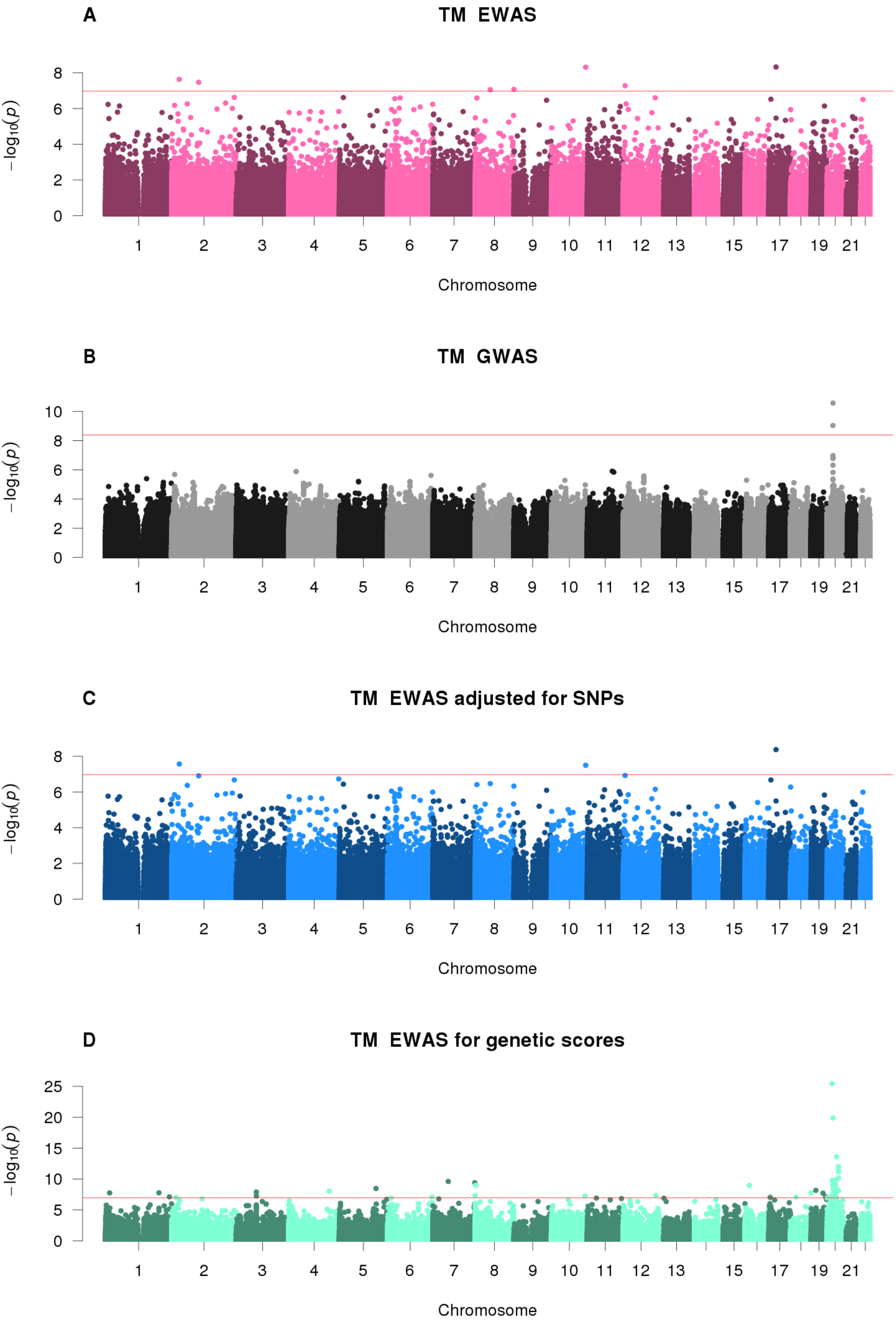
A
TM EWAS
−log10(p)
0 2 4 6 8
1 2 3 4 5 6 7 8 9 10 11 12 13 15 17 19 21
Chromosome
B
TM GWAS
−log10(p)
0 2 4 6 8 10
1 2 3 4 5 6 7 8 9 10 11 12 13 15 17 19 21
Chromosome
C
TM EWAS adjusted for SNPs
−log10(p)
0 2 4 6 8
1 2 3 4 5 6 7 8 9 10 11 12 13 15 17 19 21
Chromosome
D
TM EWAS for genetic scores
−log10(p)
0 5 10 15 20 25
1 2 3 4 5 6 7 8 9 10 11 12 13 15 17 19 21
Chromosome

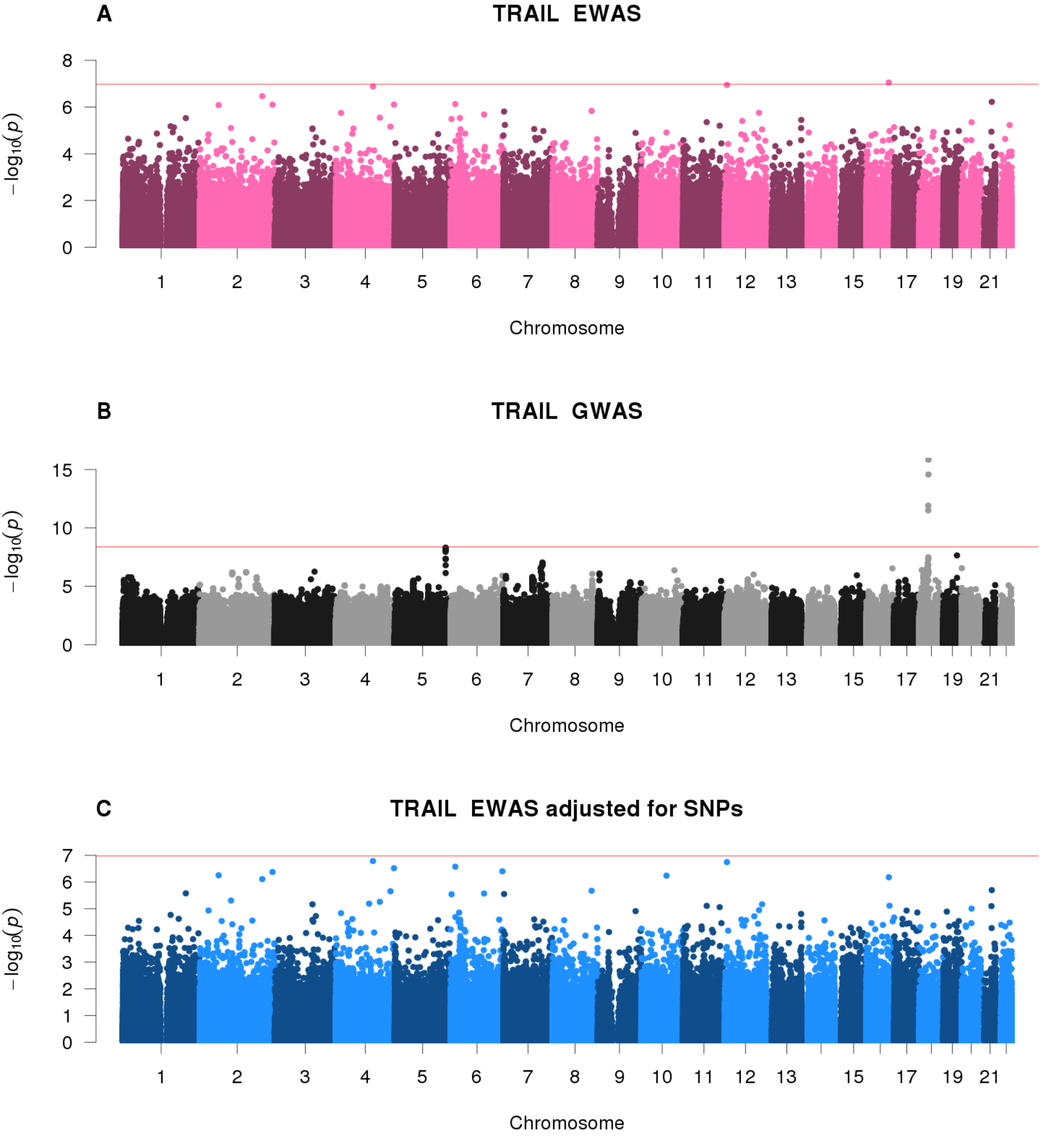
A
TRAIL EWAS
−log10(p)
8
6
4
2
0
1
2
3
4
5
6
7
8
9
10
11
12
13
15
17
19
21
Chromosome
B
TRAIL GWAS
15
10
5
0
C
TRAIL EWAS adjusted for SNPs
7
6
5
4
3
2
1
0

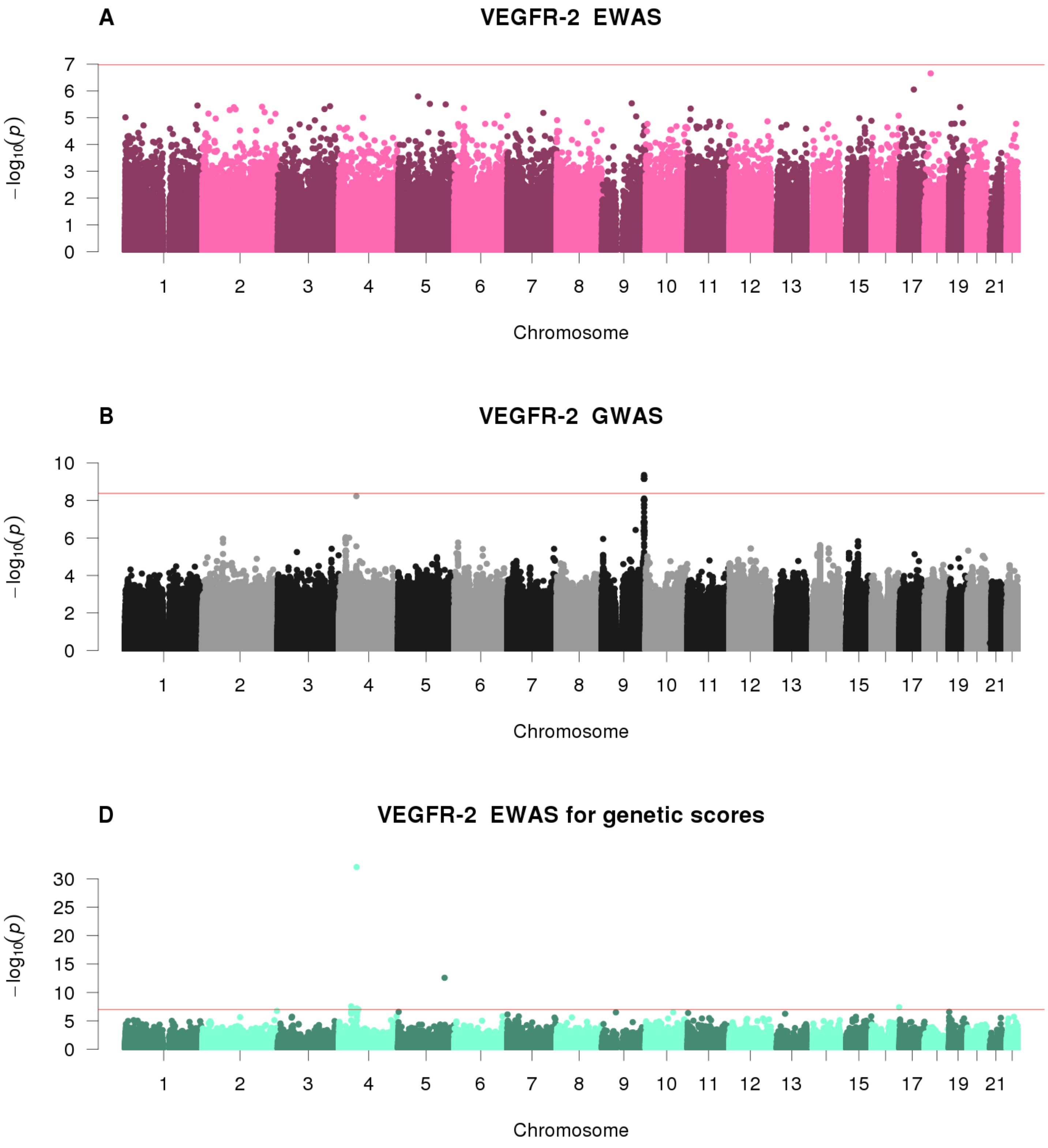
A
VEGFR-2 EWAS
−log10(p)
Chromosome
B
VEGFR-2 GWAS
−log10(p)
Chromosome
D
VEGFR-2 EWAS for genetic scores
−log10(p)
Chromosome
