# Supplementary material for: The relative contribution of DNA methylation and genetic variants on protein biomarkers for human diseases
Source: PLoS Genet. 2017 Sep 15;13(9):e1007005. doi: 10.1371/journal.pgen.1007005 (PMC5617224; doi:10.1371/journal.pgen.1007005)
Supplement: S6 Fig — The X-axis represents the weighted (by the regression coefficients) allelic effects by the top GWAS SNPs and the Y–axis is the measured biomarker level for each individual. For biomarkers with more than one independent SNP, all independent cis-SNPs are included, and N indicates the number of SNPs included. R-squared is the fraction of the variance in biomarker levels explained by all (N) variants. For biomarkers with N = 1, most individuals are in either of three clusters that correspond to individuals being homozygous for the minor or major allele or individuals being heterozygous. Dosage values for the imputed genotypes are used, and therefore, some individuals are located outside the three clusters. For biomarkers with more than one SNP included as IVs; more than three clusters are observed which agrees with the higher number of possible allele-combinations. (PDF) [file pgen.1007005.s006.pdf]

Supplemental Fig S6. *Allelic effects on biomarker levels. The X-axis represents the weighted (by the regression coefficients) allelic effects by the top GWAS SNPs and the Y–axis is the measured biomarker level for each individual. For biomarkers with more than one independent SNP, all independent cis-SNPs are included, and N indicates the number of SNPs included. R-squared is the fraction of the variance in biomarker levels explained by all (N) variants. For biomarkers with N=1, most individuals are in either of three clusters that correspond to individuals being homozygous for the minor or major allele or individuals being heterozygous. Dosage values for the imputed genotypes are used, and therefore, some individuals are located outside the three clusters. For biomarkers with more than one SNP included as IVs; more than three clusters are observed which agrees with the higher number of possible allele-combinations*

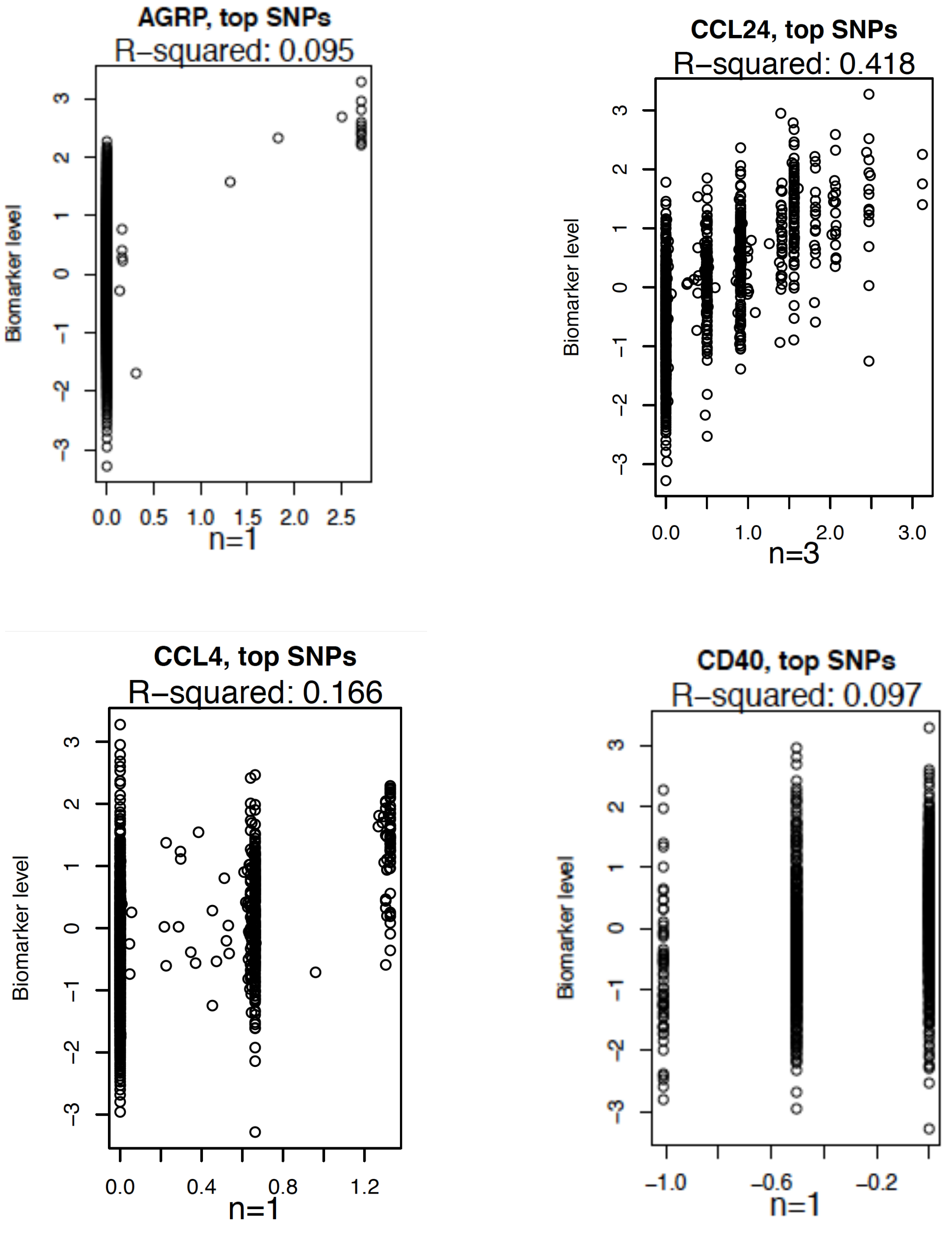

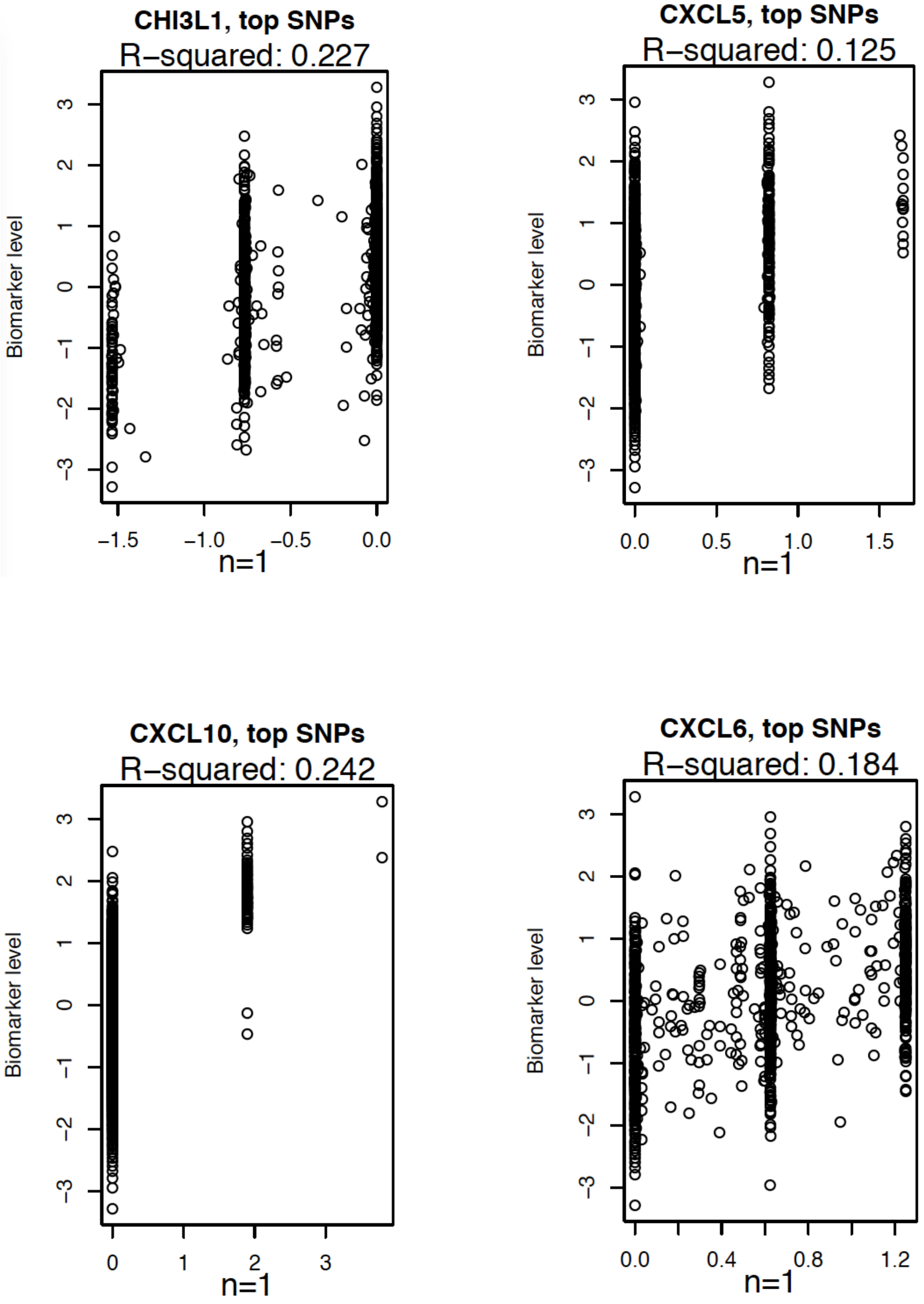
CHI3L1, top SNPs
R−squared: 0.227
Biomarker level
3
2
1
0
−1
−2
−3
−1.5
−1.0
−0.5
0.0
n=1
CXCL5, top SNPs
R−squared: 0.125
Biomarker level
3
2
1
0
−1
−2
−3
0.0
0.5
1.0
1.5
n=1
CXCL10, top SNPs
R−squared: 0.242
Biomarker level
3
2
1
0
−1
−2
−3
0
1
2
3
n=1
CXCL6, top SNPs
R−squared: 0.184
Biomarker level
3
2
1
0
−1
−2
−3
0.0
0.4
0.8
1.2
n=1

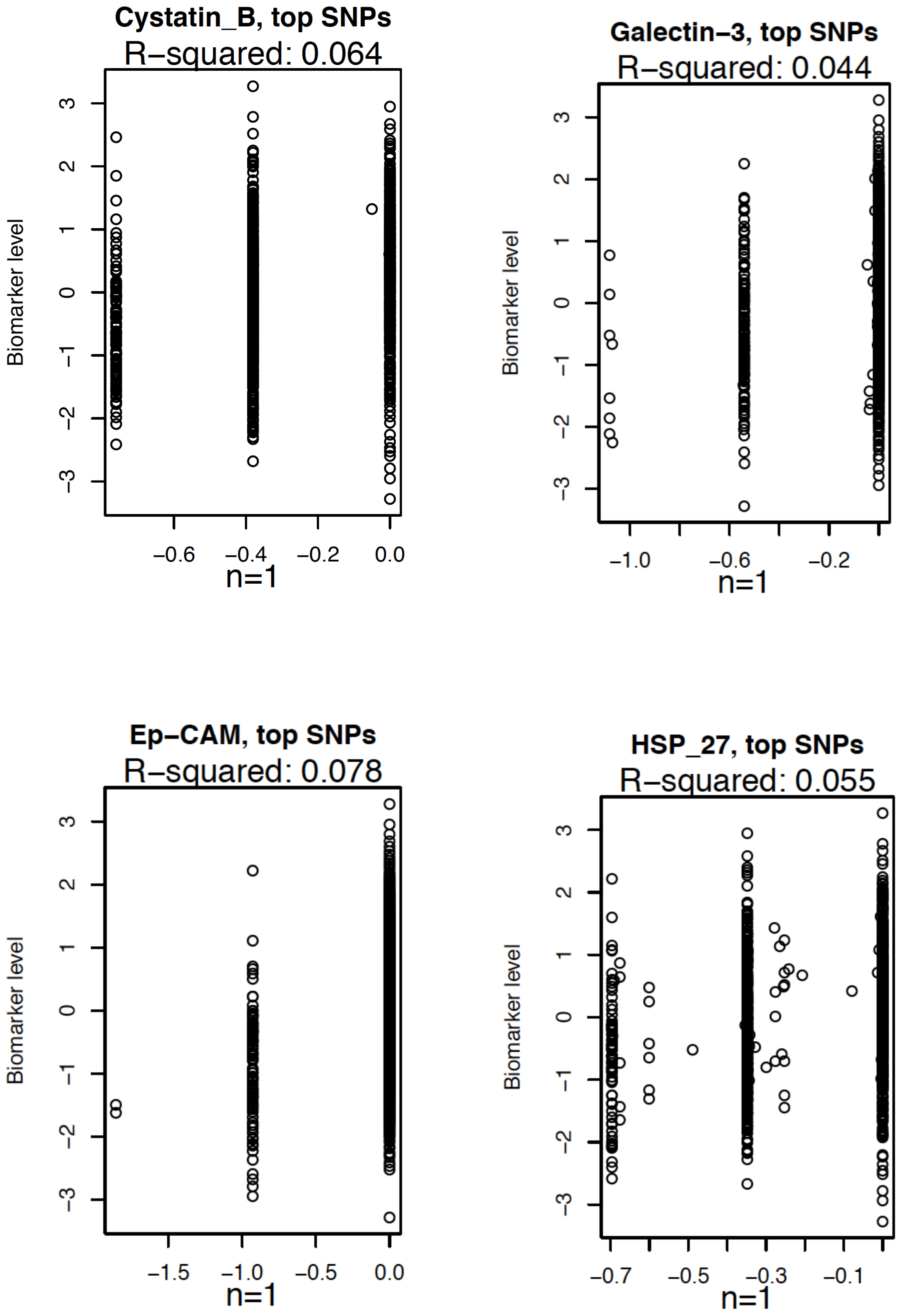
Cystatin_B, top SNPs
R–squared: 0.064
Biomarker level
n=1
Galectin–3, top SNPs
R–squared: 0.044
Biomarker level
n=1
Ep–CAM, top SNPs
R–squared: 0.078
Biomarker level
n=1
HSP_27, top SNPs
R–squared: 0.055
Biomarker level
n=1

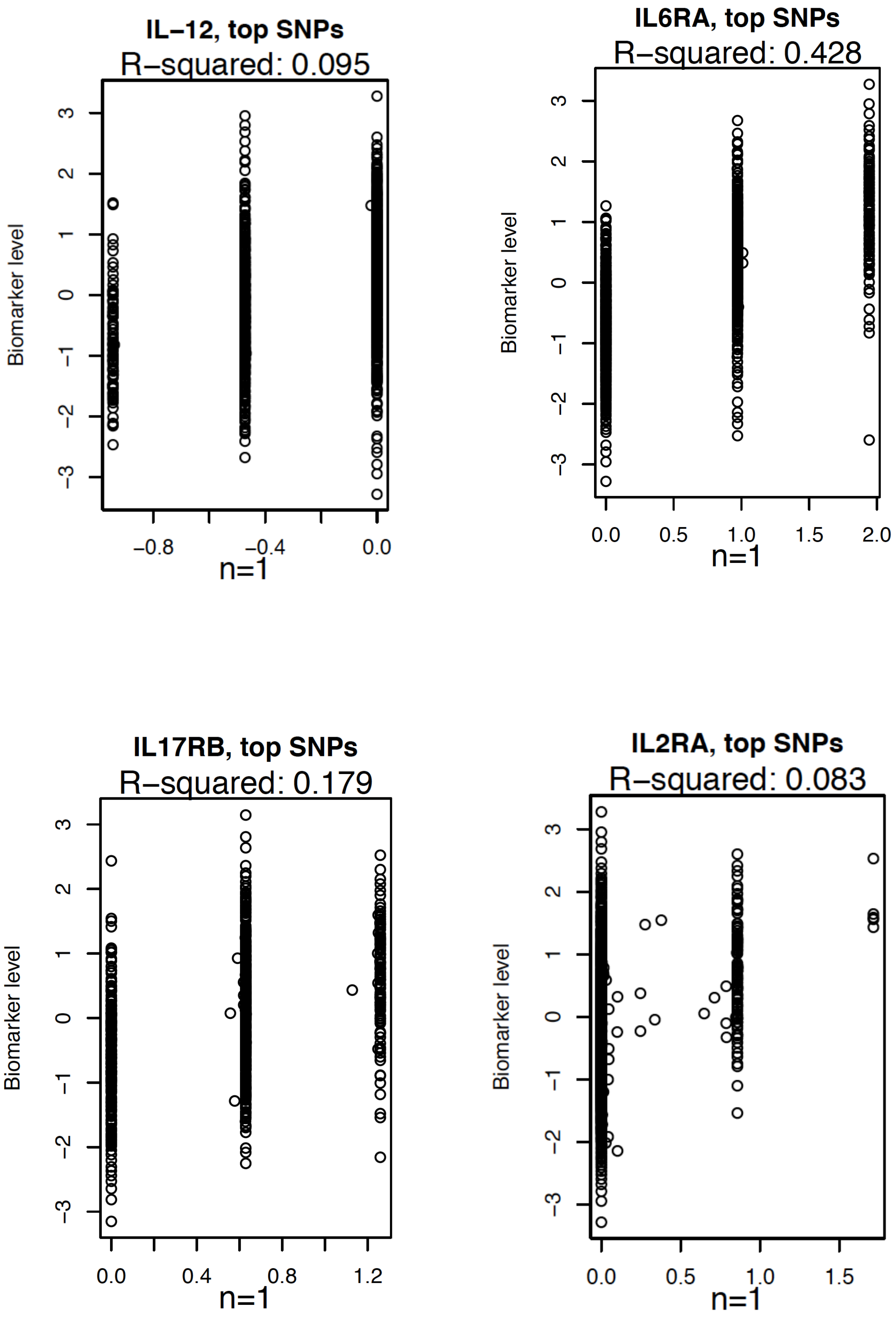
IL−12, top SNPs
R−squared: 0.095
Biomarker level
n=1
IL6RA, top SNPs
R−squared: 0.428
Biomarker level
n=1
IL17RB, top SNPs
R−squared: 0.179
Biomarker level
n=1
IL2RA, top SNPs
R−squared: 0.083
Biomarker level
n=1

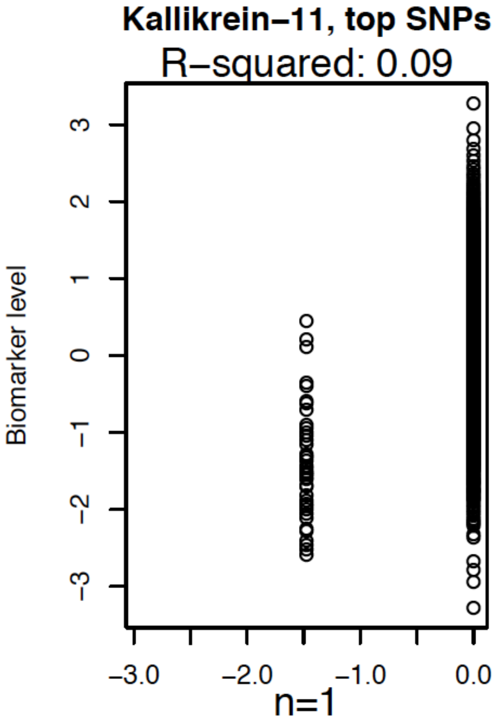
Kallikrein–11, top SNPs
R–squared: 0.09
Biomarker level
n=1
–3.0
–2.0
–1.0
0.0
–3
–2
–1
0
1
2
3

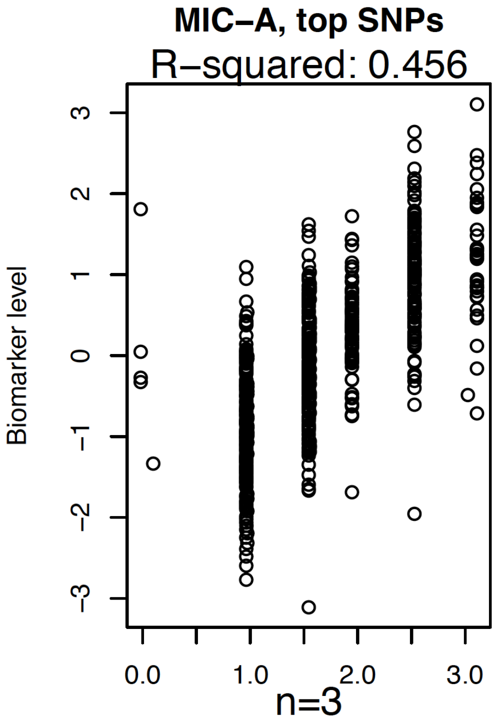
MIC–A, top SNPs
R–squared: 0.456
Biomarker level
n=3
0.0
1.0
2.0
3.0
–3
–2
–1
0
1
2
3

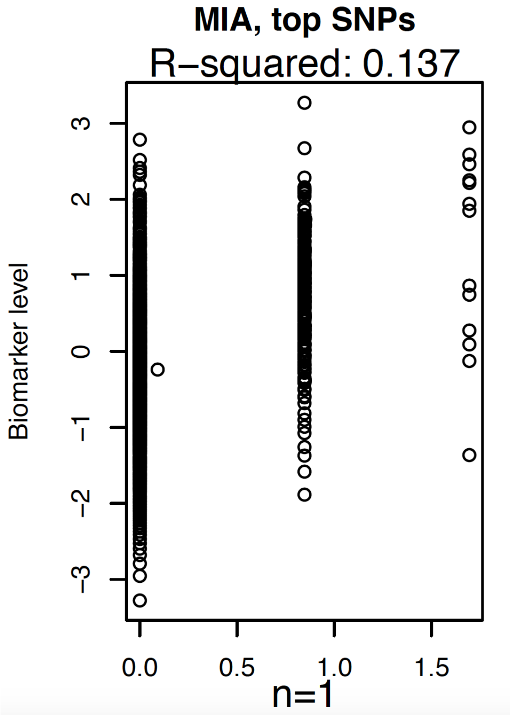
MIA, top SNPs
R–squared: 0.137
Biomarker level
n=1
0.0
0.5
1.0
1.5
–3
–2
–1
0
1
2
3

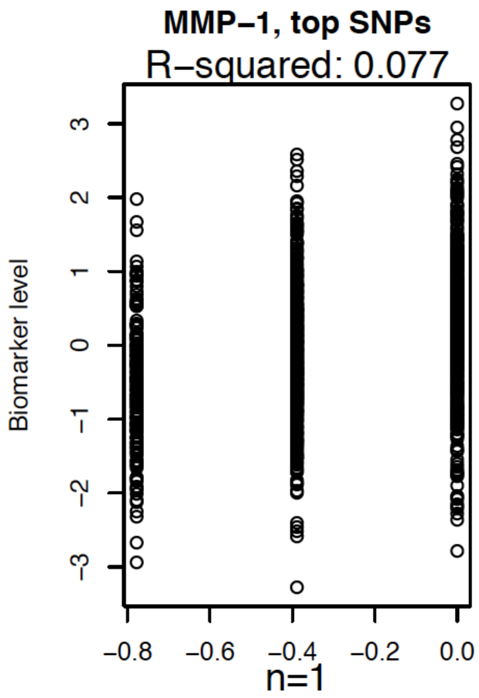
MMP–1, top SNPs
R–squared: 0.077
Biomarker level
n=1
–0.8
–0.6
–0.4
–0.2
0.0
–3
–2
–1
0
1
2
3

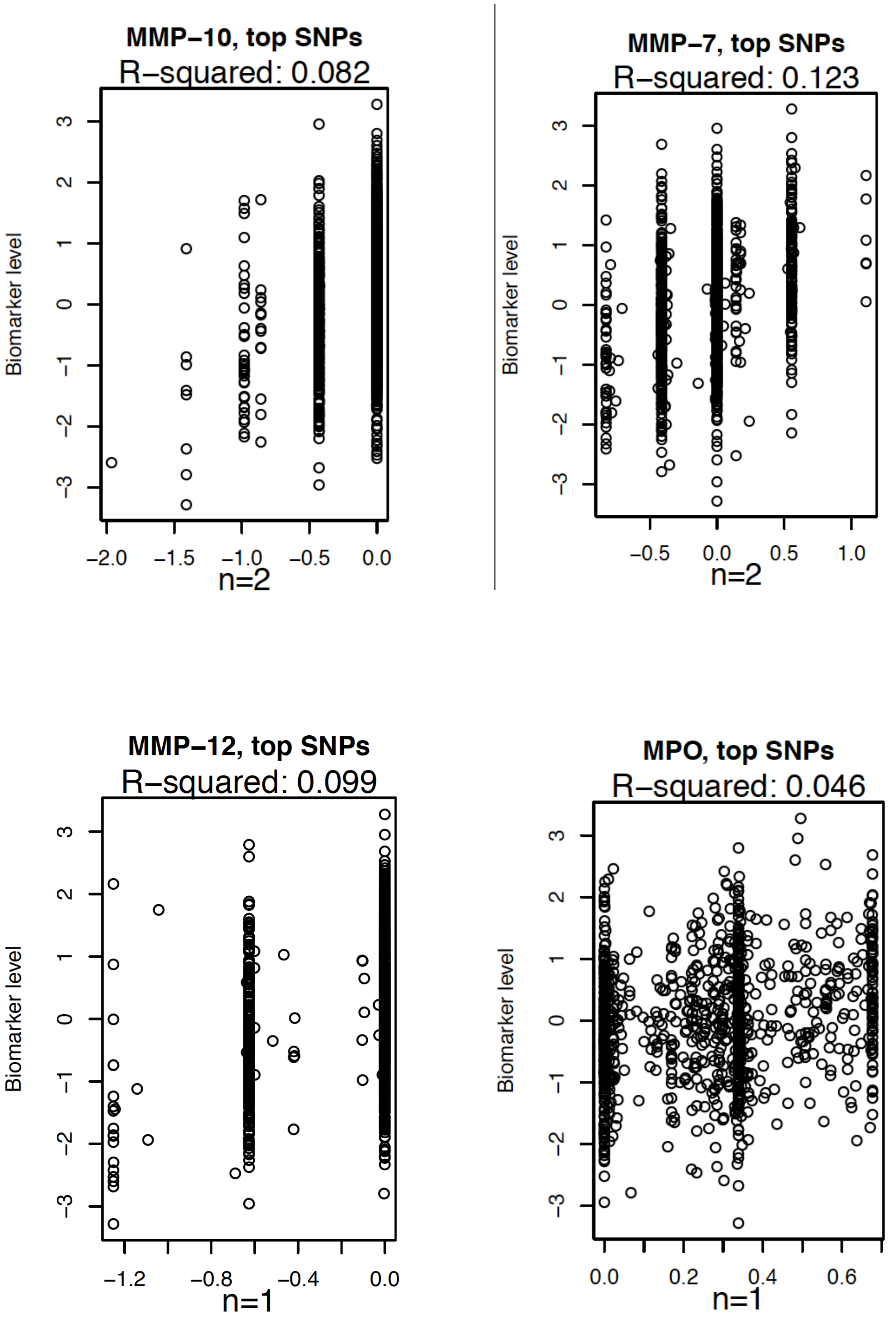
MMP−10, top SNPs
R−squared: 0.082
Biomarker level
n=2
MMP−7, top SNPs
R−squared: 0.123
Biomarker level
n=2
MMP−12, top SNPs
R−squared: 0.099
Biomarker level
n=1
MPO, top SNPs
R−squared: 0.046
Biomarker level
n=1

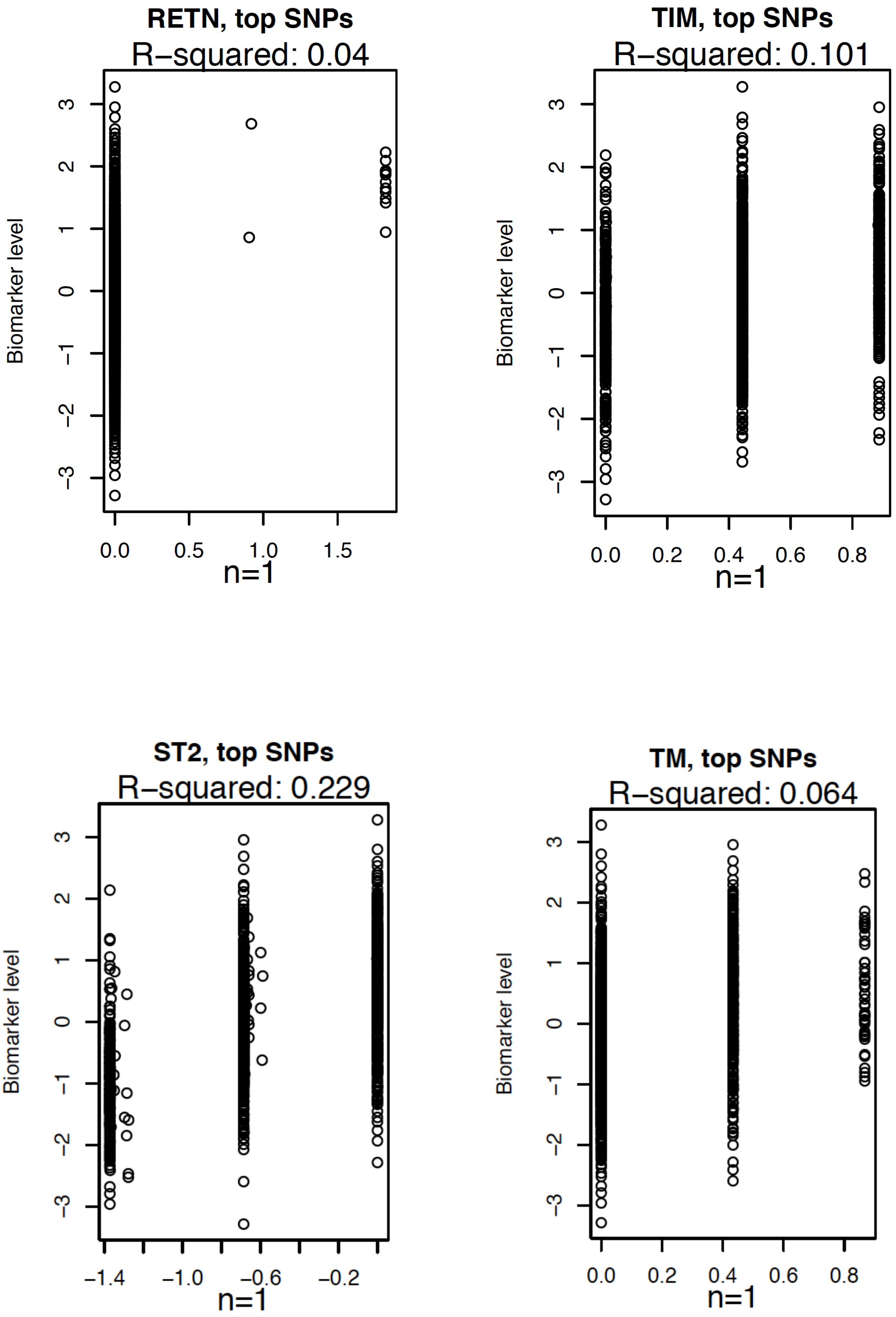
RETN, top SNPs
R–squared: 0.04
TIM, top SNPs
R–squared: 0.101
ST2, top SNPs
R–squared: 0.229
TM, top SNPs
R–squared: 0.064
Biomarker level
n=1

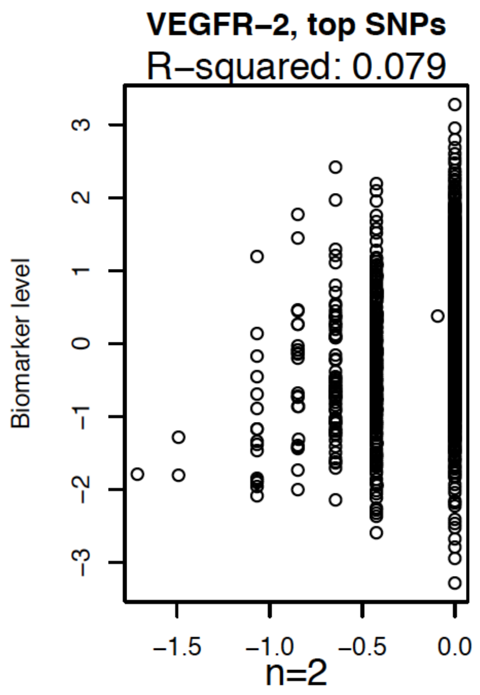
VEGFR−2, top SNPs
R−squared: 0.079
Biomarker level
3
2
1
0
−1
−2
−3
−1.5
−1.0
−0.5
0.0
n=2
