# Supplementary material for: The relative contribution of DNA methylation and genetic variants on protein biomarkers for human diseases
Source: PLoS Genet. 2017 Sep 15;13(9):e1007005. doi: 10.1371/journal.pgen.1007005 (PMC5617224; doi:10.1371/journal.pgen.1007005)
Supplement: S7 Fig — GS are calculated for biomarkers with cis-regulatory SNPs only. (PDF) [file pgen.1007005.s007.pdf]

Supplemental Fig S7. *EWAS for genetic scores (GS).*
GS are calculated for biomarkers with cis-regulatory SNPs only.

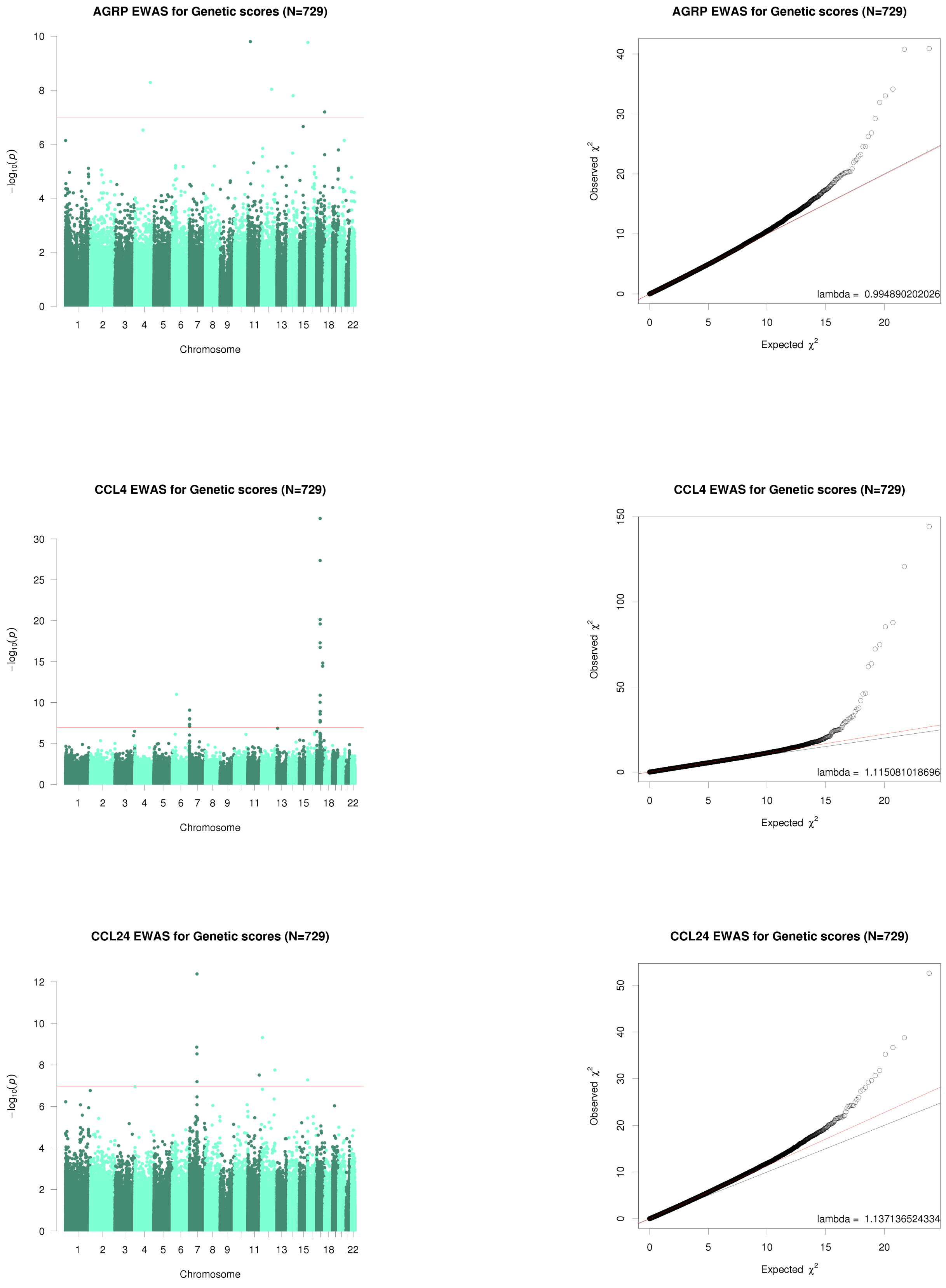

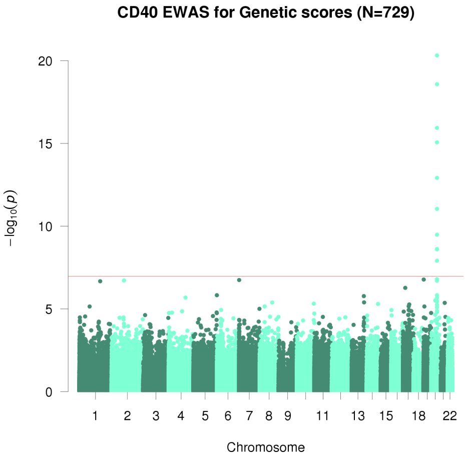
CD40 EWAS for Genetic scores (N=729)
$-\log_{10}(p)$
0
5
10
15
20
1 2 3 4 5 6 7 8 9 11 13 15 18 22
Chromosome

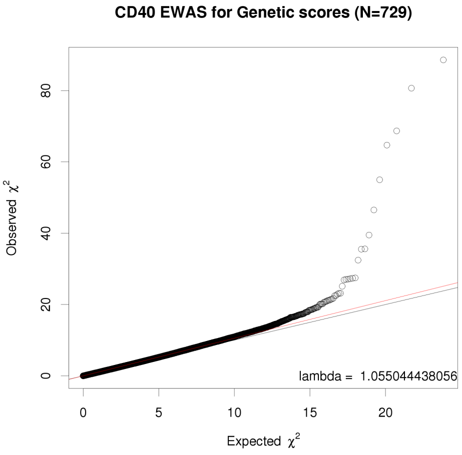
CD40 EWAS for Genetic scores (N=729)
Observed χ^2
0
20
40
60
80
lambda = 1.055044438056
0 5 10 15 20
Expected χ^2

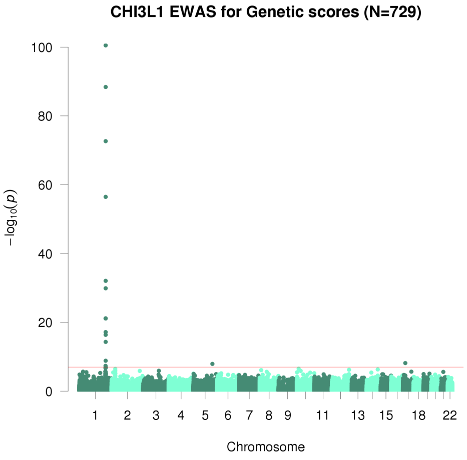
CHI3L1 EWAS for Genetic scores (N=729)
$-\log_{10}(p)$
0
20
40
60
80
100
1 2 3 4 5 6 7 8 9 11 13 15 18 22
Chromosome

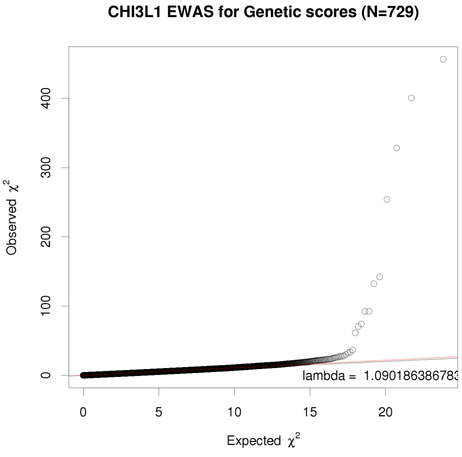
CHI3L1 EWAS for Genetic scores (N=729)
Observed χ^2
0
100
200
300
400
lambda = 1.09018638678
0 5 10 15 20
Expected χ^2

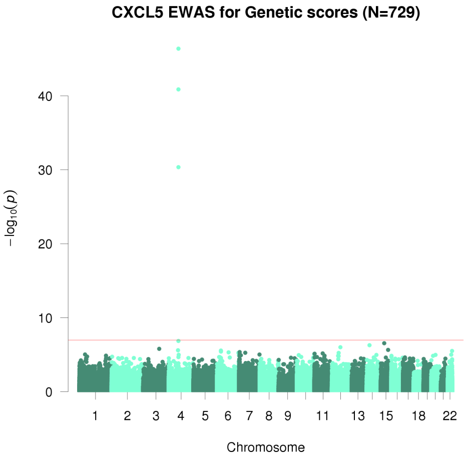
CXCL5 EWAS for Genetic scores (N=729)
$-\log_{10}(p)$
0
10
20
30
40
1 2 3 4 5 6 7 8 9 11 13 15 18 22
Chromosome

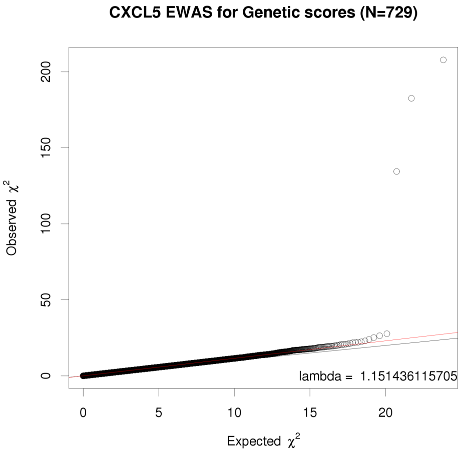
CXCL5 EWAS for Genetic scores (N=729)
Observed χ^2
0
50
100
150
200
lambda = 1.151436115705
0 5 10 15 20
Expected χ^2

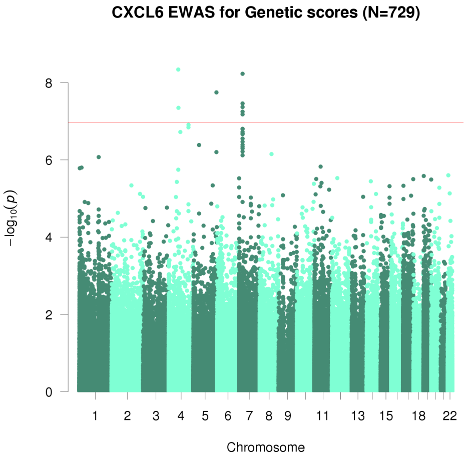
CXCL6 EWAS for Genetic scores (N=729)
$-\log_{10}(p)$
Chromosome
1 2 3 4 5 6 7 8 9 11 13 15 18 22

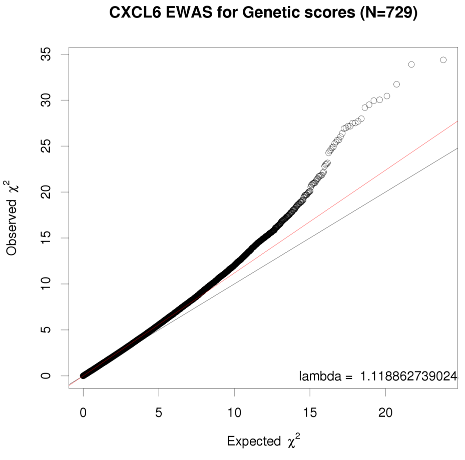
CXCL6 EWAS for Genetic scores (N=729)
Observed χ^2
Expected χ^2
lambda = 1.118862739024

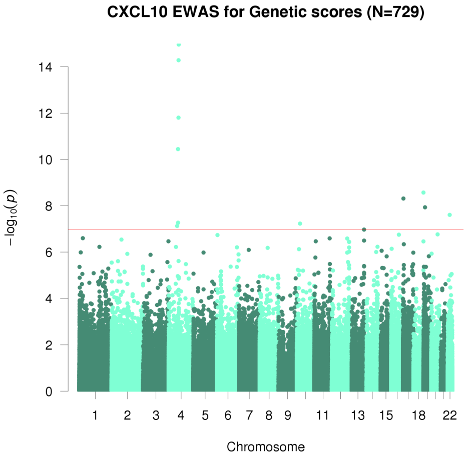
CXCL10 EWAS for Genetic scores (N=729)
$-\log_{10}(p)$
Chromosome
1 2 3 4 5 6 7 8 9 11 13 15 18 22

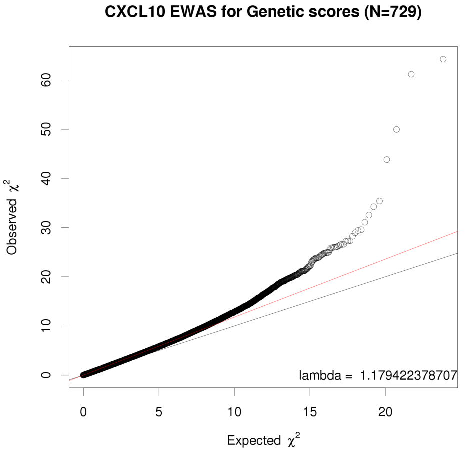
CXCL10 EWAS for Genetic scores (N=729)
Observed χ^2
Expected χ^2
lambda = 1.179422378707

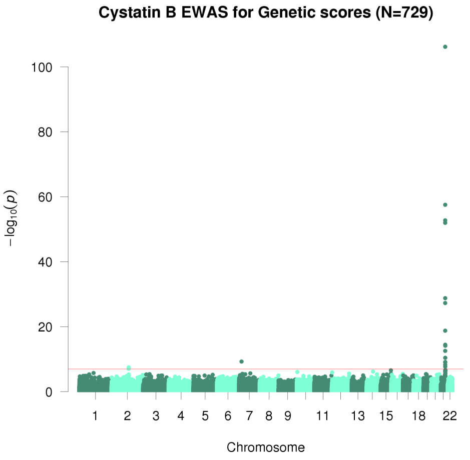
Cystatin B EWAS for Genetic scores (N=729)
$-\log_{10}(p)$
Chromosome
1 2 3 4 5 6 7 8 9 11 13 15 18 22

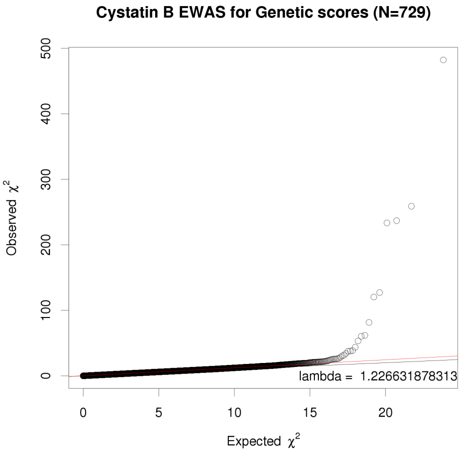
Cystatin B EWAS for Genetic scores (N=729)
Observed χ^2
Expected χ^2
lambda = 1.226631878313

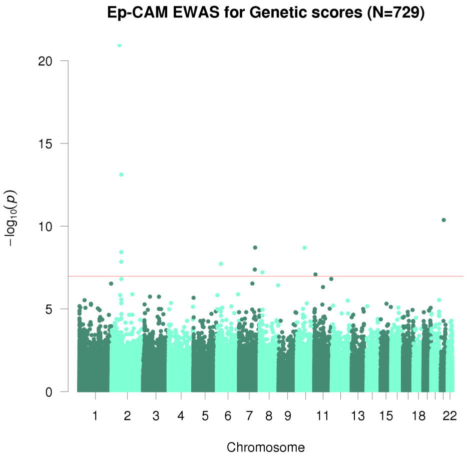
Ep-CAM EWAS for Genetic scores (N=729)
$-\log_{10}(p)$
Chromosome
0
5
10
15
20
1 2 3 4 5 6 7 8 9 11 13 15 18 22

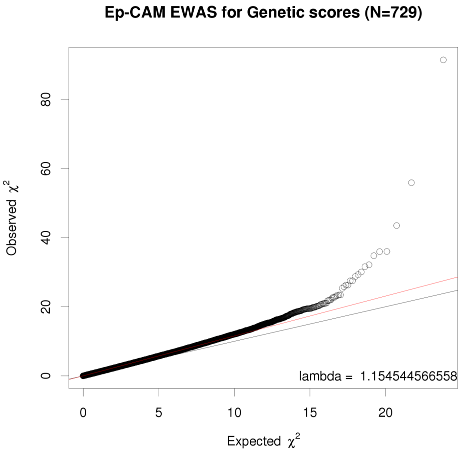
Ep-CAM EWAS for Genetic scores (N=729)
Observed χ^2
Expected χ^2
lambda = 1.154544566558
0 20 40 60 80
0 5 10 15 20

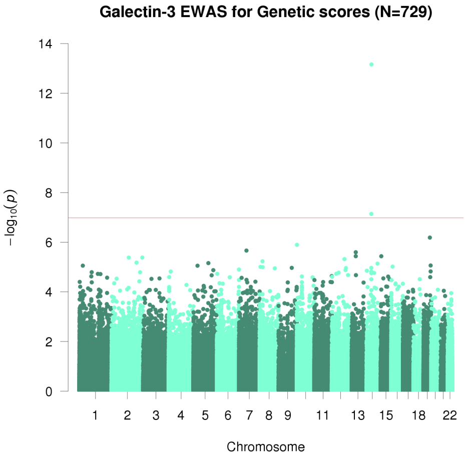
Galectin-3 EWAS for Genetic scores (N=729)
$-\log_{10}(p)$
Chromosome
0 2 4 6 8 10 12 14
1 2 3 4 5 6 7 8 9 11 13 15 18 22

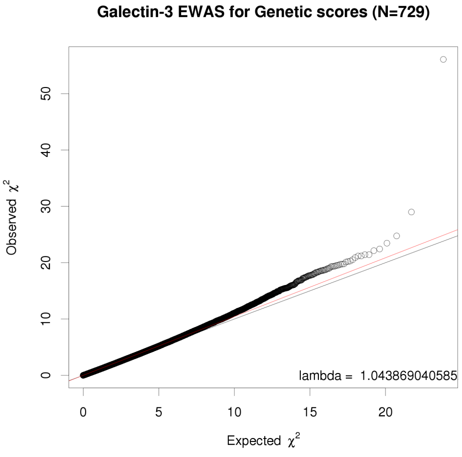
Galectin-3 EWAS for Genetic scores (N=729)
Observed χ^2
Expected χ^2
lambda = 1.043869040585
0 10 20 30 40 50
0 5 10 15 20

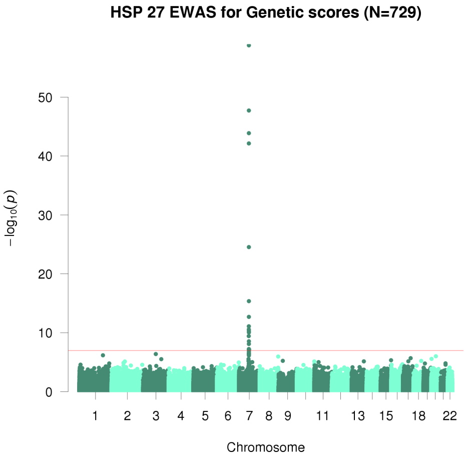
HSP 27 EWAS for Genetic scores (N=729)
$-\log_{10}(p)$
Chromosome
0 10 20 30 40 50
1 2 3 4 5 6 7 8 9 11 13 15 18 22

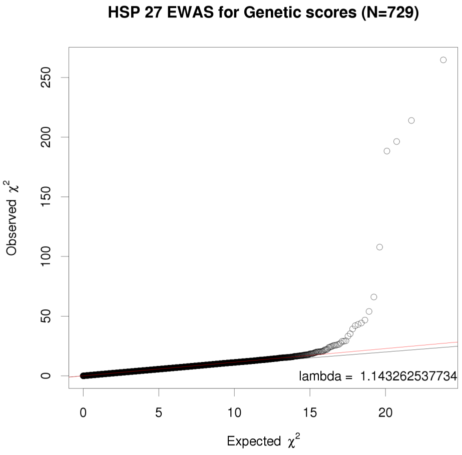
HSP 27 EWAS for Genetic scores (N=729)
Observed χ^2
Expected χ^2
lambda = 1.143262537734
0 50 100 150 200 250
0 5 10 15 20

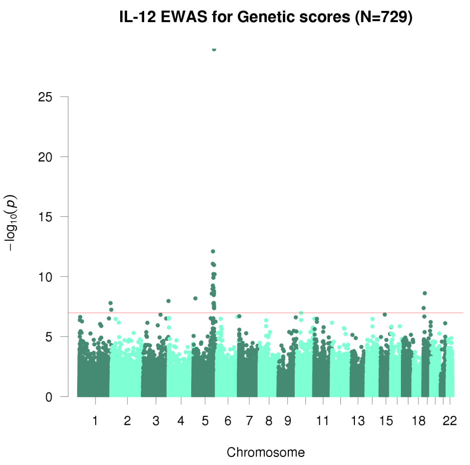
IL-12 EWAS for Genetic scores (N=729)
$-\log_{10}(p)$
Chromosome
1 2 3 4 5 6 7 8 9 11 13 15 18 22
0 5 10 15 20 25

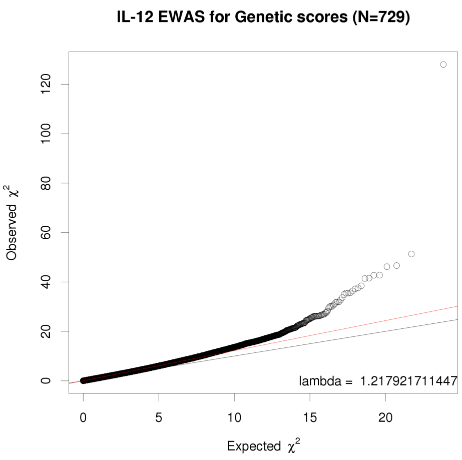
IL-12 EWAS for Genetic scores (N=729)
Observed χ^2
Expected χ^2
lambda = 1.217921711447

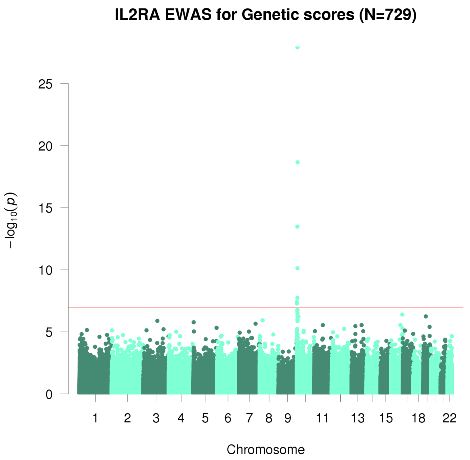
IL2RA EWAS for Genetic scores (N=729)
$-\log_{10}(p)$
Chromosome
1 2 3 4 5 6 7 8 9 11 13 15 18 22
0 5 10 15 20 25

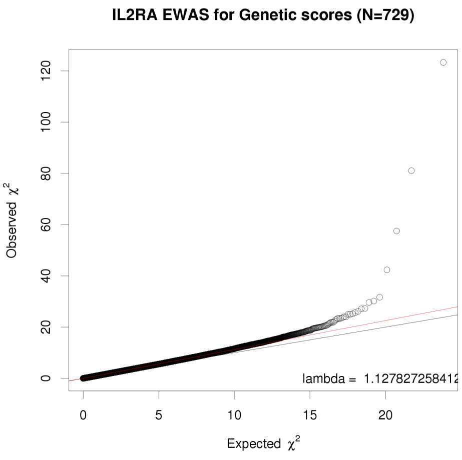
IL2RA EWAS for Genetic scores (N=729)
Observed χ^2
Expected χ^2
lambda = 1.127827258412

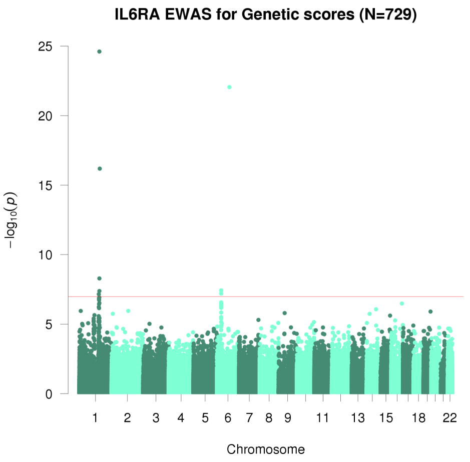
IL6RA EWAS for Genetic scores (N=729)
$-\log_{10}(p)$
Chromosome
1 2 3 4 5 6 7 8 9 11 13 15 18 22
0 5 10 15 20 25

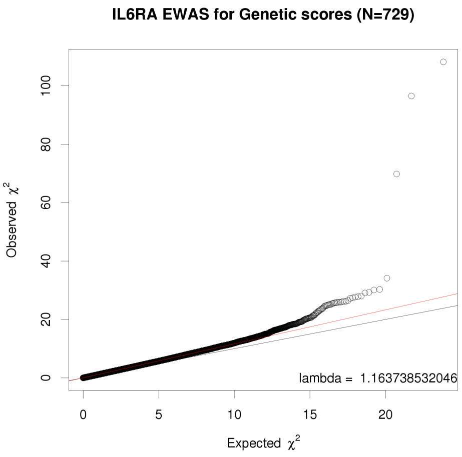
IL6RA EWAS for Genetic scores (N=729)
Observed χ^2
Expected χ^2
lambda = 1.163738532046

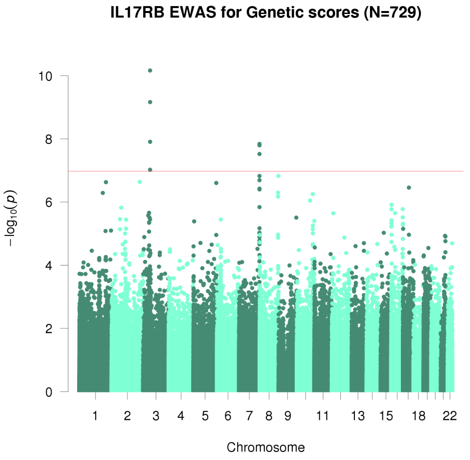
IL17RB EWAS for Genetic scores (N=729)
$-\log_{10}(p)$
Chromosome
0
2
4
6
8
10
1 2 3 4 5 6 7 8 9 11 13 15 18 22

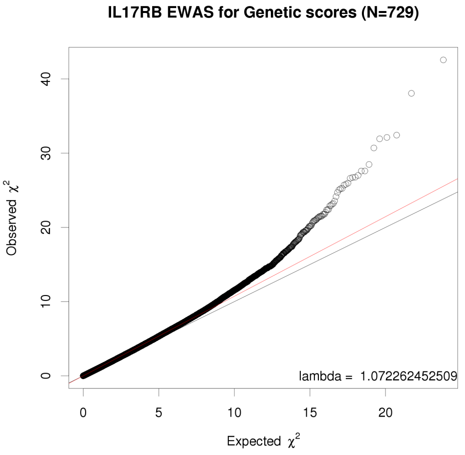
IL17RB EWAS for Genetic scores (N=729)
Observed χ^2
Expected χ^2
lambda = 1.072262452509
0 10 20 30 40
0 5 10 15 20

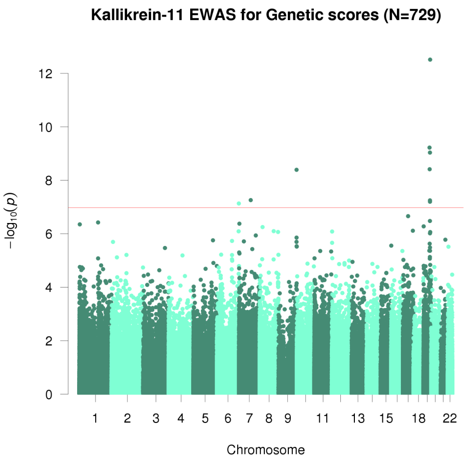
Kallikrein-11 EWAS for Genetic scores (N=729)
$-\log_{10}(p)$
Chromosome
0
2
4
6
8
10
12
1 2 3 4 5 6 7 8 9 11 13 15 18 22

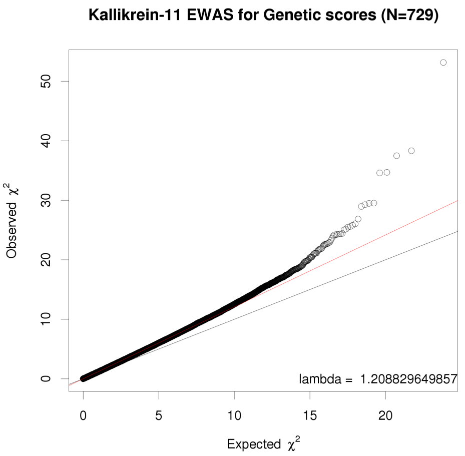
Kallikrein-11 EWAS for Genetic scores (N=729)
Observed χ^2
Expected χ^2
lambda = 1.208829649857
0 10 20 30 40 50
0 5 10 15 20

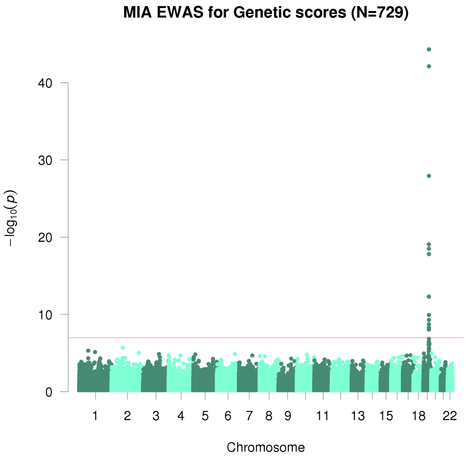
MIA EWAS for Genetic scores (N=729)
$-\log_{10}(p)$
Chromosome
0
10
20
30
40
1 2 3 4 5 6 7 8 9 11 13 15 18 22

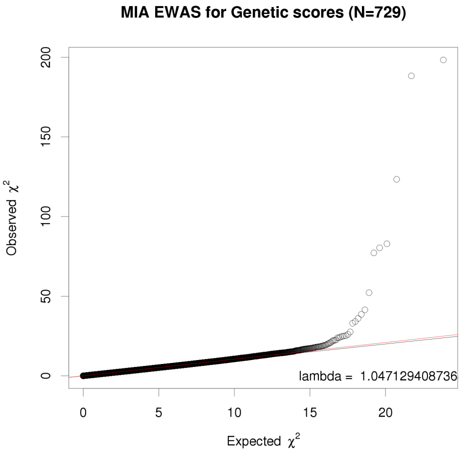
MIA EWAS for Genetic scores (N=729)
Observed χ^2
Expected χ^2
lambda = 1.047129408736
0 50 100 150 200
0 5 10 15 20

MIC-A EWAS for Genetic scores (N=729)

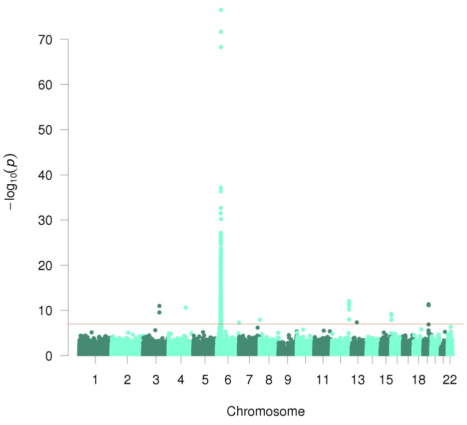

MIC-A EWAS for Genetic scores (N=729)

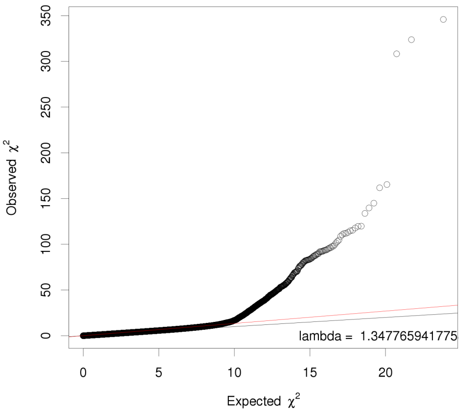

MMP-1 EWAS for Genetic scores (N=729)

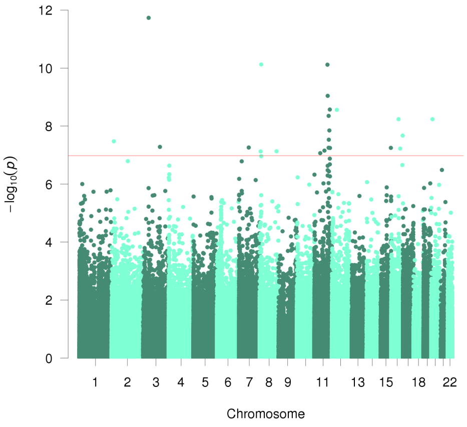

MMP-1 EWAS for Genetic scores (N=729)

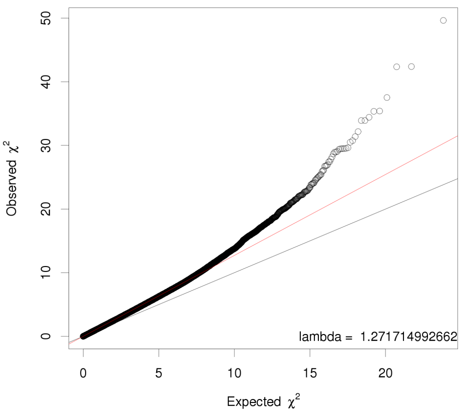

MMP-7 EWAS for Genetic scores (N=729)

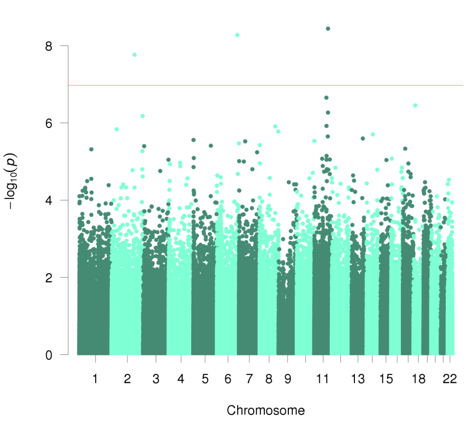

MMP-7 EWAS for Genetic scores (N=729)

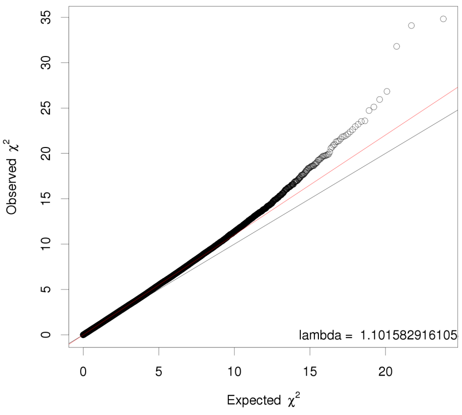

MMP-10 EWAS for Genetic scores (N=729)

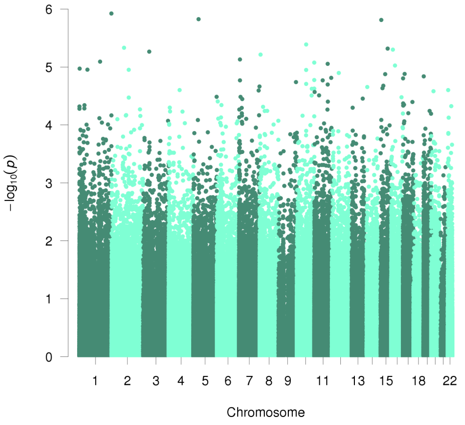

MMP-10 EWAS for Genetic scores (N=729)

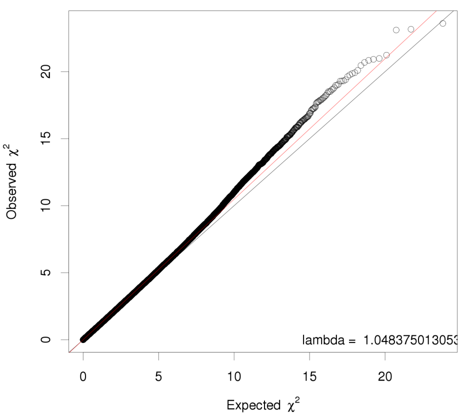

MMP-12 EWAS for Genetic scores (N=729)

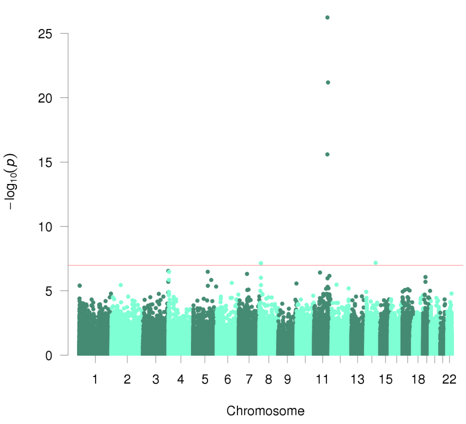

MMP-12 EWAS for Genetic scores (N=729)

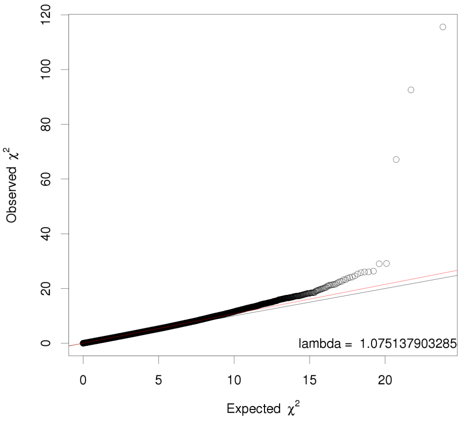

MPO EWAS for Genetic scores (N=729)

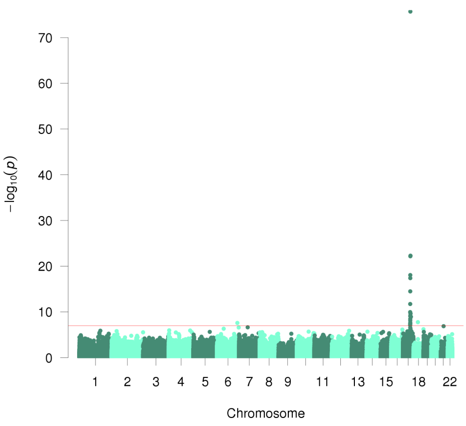

MPO EWAS for Genetic scores (N=729)

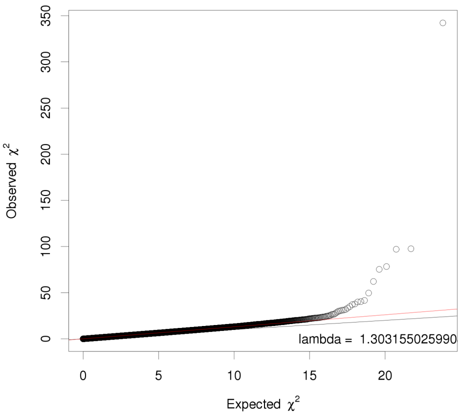

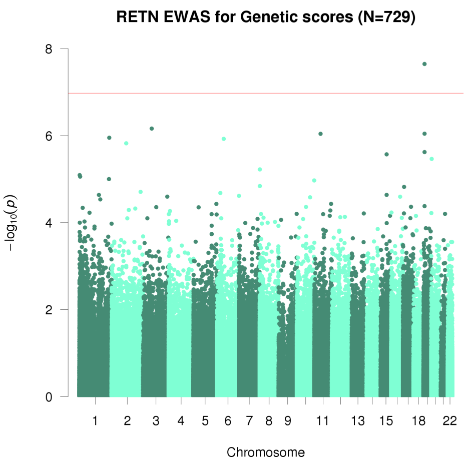
RETN EWAS for Genetic scores (N=729)
$-\log_{10}(p)$
Chromosome

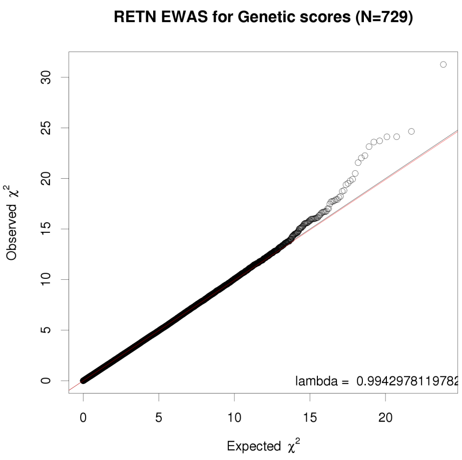
RETN EWAS for Genetic scores (N=729)
Observed χ^2
Expected χ^2
lambda = 0.9942978119782

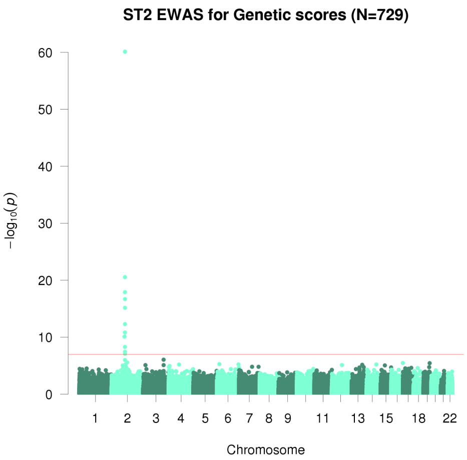
ST2 EWAS for Genetic scores (N=729)
$-\log_{10}(p)$
Chromosome

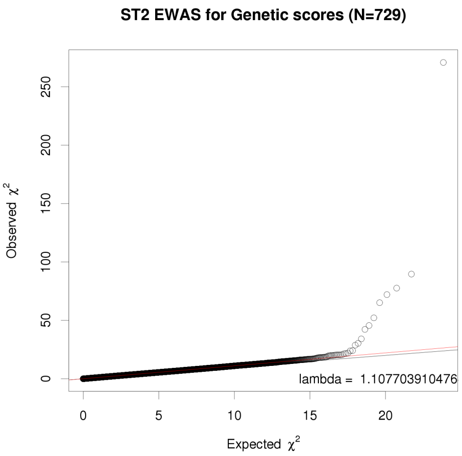
ST2 EWAS for Genetic scores (N=729)
Observed χ^2
Expected χ^2
lambda = 1.107703910476

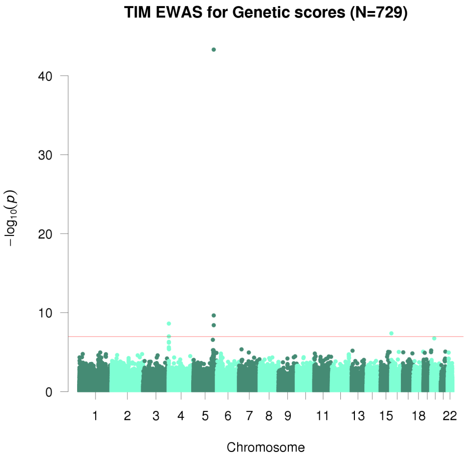
TIM EWAS for Genetic scores (N=729)
$-\log_{10}(p)$
Chromosome

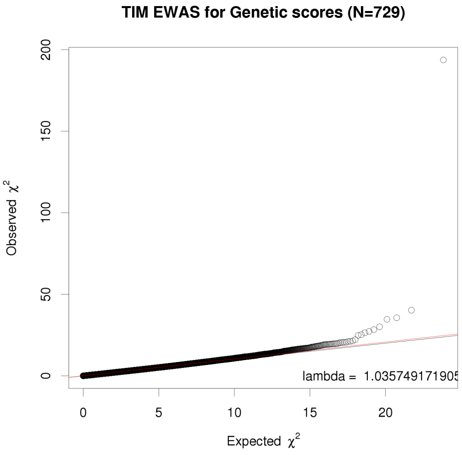
TIM EWAS for Genetic scores (N=729)
Observed χ^2
Expected χ^2
lambda = 1.035749171905

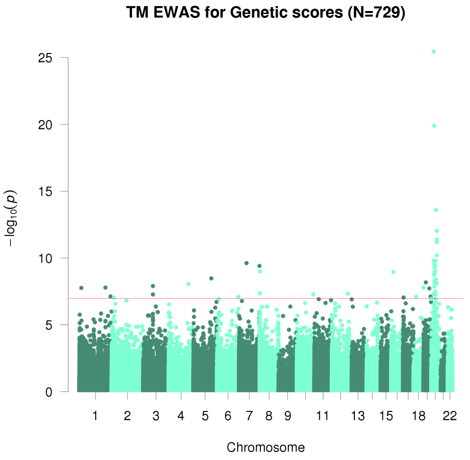
TM EWAS for Genetic scores (N=729)
$-\log_{10}(p)$
Chromosome
0
5
10
15
20
25
1 2 3 4 5 6 7 8 9 11 13 15 18 22

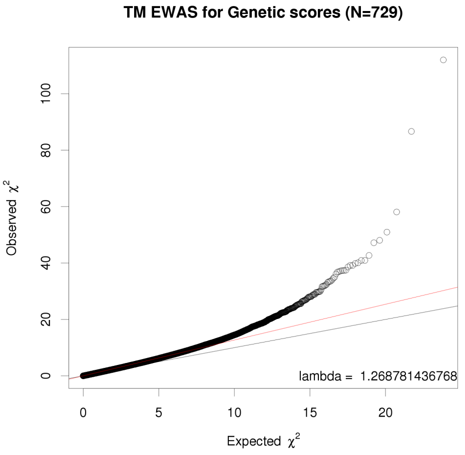
TM EWAS for Genetic scores (N=729)
Observed χ^2
Expected χ^2
lambda = 1.268781436768
0
20
40
60
80
100
0 5 10 15 20

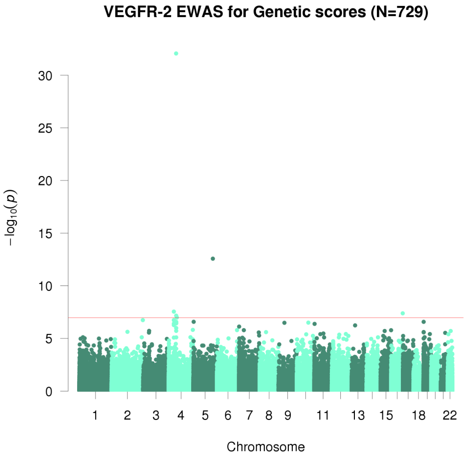
VEGFR-2 EWAS for Genetic scores (N=729)
$-\log_{10}(p)$
Chromosome
0
5
10
15
20
25
30
1 2 3 4 5 6 7 8 9 11 13 15 18 22

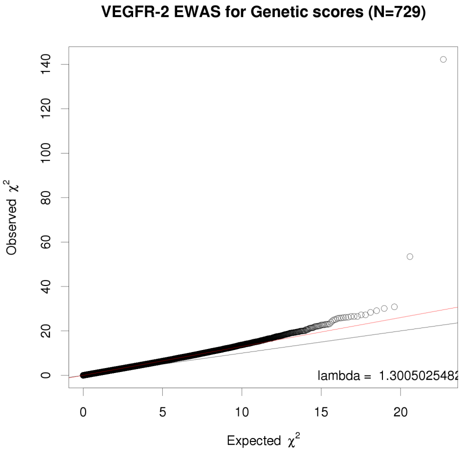
VEGFR-2 EWAS for Genetic scores (N=729)
Observed χ^2
Expected χ^2
lambda = 1.3005025482
0
20
40
60
80
100
120
140
0 5 10 15 20
